# Investigation of Proteome-Tetrazine Reactivity for a Highly Selective Tetrazine Ligation in Live Cells

**DOI:** 10.1021/acscentsci.5c00525

**Published:** 2025-05-06

**Authors:** Junyoung Park, Juhee Hahm, Junhyeong Yim, Hyelim Lee, Hwan Min Hwang, Soyeon Lee, Ju-Young Park, Arun Velladurai, Jagadeesh Kumar Gangasani, Hana Cho, Hankum Park, Minju Lee, Jeehee Lee, Hyunuk Eom, Woon Ju Song, Sanghee Lee, Eunha Kim, Jongmin Park

**Affiliations:** † Department of Chemistry, 34962 Kangwon National University, Chuncheon 24341, Republic of Korea; ‡ Multidimensional Genomics Research Center, 34962 Kangwon National University, Chuncheon 24341, Republic of Korea; § Medicinal Materials Research Center, Biomedical Research Division, 425395 Korea Institute of Science and Technology (KIST), Seoul 02792, Republic of Korea; ∥ Molecular Science and Technology Research Center, 34919 Ajou University, Suwon 16499, Republic of Korea; ⊥ Department of Molecular Science and Technology, 34919 Ajou University, Suwon 16499, Republic of Korea; # Institute for Molecular Science and Fusion Technology, 34962 Kangwon National University, Chuncheon 24341, Republic of Korea; g Department of Dental Sciences, School of Dentistry, 26725 Seoul National University, Seoul 08826, Republic of Korea; h Institute for Data Innovation in Science, 26725 Seoul National University, Seoul 08826, Republic of Korea; i Dental Multiomics Center, School of Dentistry and Dental Research Institute, 26725 Seoul National University, Seoul 08826, Republic of Korea; j Department of HY-KIST Bio-convergence, Hanyang University, Seoul 04763, Republic of Korea; k Department of Chemistry, 26725 Seoul National University, 1 Gwanak-ro, Gwanak-gu, Seoul 08826, Republic of Korea; l KHU-KIST Department of Converging Science and Technology, Kyung Hee University, Seoul 02453, Republic of Korea; m Advanced College of Bio-convergence Engineering, 34919 Ajou University, Suwon 16499, Republic of Korea; n Department of Bio-convergence Engineering, 34919 Ajou University, Suwon 16499, Republic of Korea; o Department of Otolaryngology, Ajou University School of Medicine, Suwon 16499, Republic of Korea

## Abstract

Tetrazine has been widely used in bio-orthogonal click chemistry for diverse biological applications due to its short reaction time and excellent bio-orthogonality. For efficient click reaction, the stability of tetrazine in physiological conditions is one of the key issues in biological applications. However, the reactions between tetrazine and biomolecules have barely been studied. Here, we investigated nonspecific proteome labeling by tetrazine derivatives. Systematic investigation of proteome reactivities of 23 tetrazine derivatives showed their structure-dependent proteome reactivities. We further investigated the relationship between the proteome reactivity of tetrazine derivatives and selectivity of *in situ* tetrazine-*trans*-cyclooctene (**Tz-TCO**) click chemistry-mediated fluorescent labeling of BTK protein in live cells. Intriguingly, a tetrazine derivative **SiR-Tz20** with minimal proteome reactivity showed a highly selective BTK labeling efficiency in live cells and in an *ex vivo* mouse model. Our results demonstrate that the proteome reactivity of tetrazine derivatives is critical for their selectivity in click reaction toward accurate fluorescent protein imaging in live cells.

## Graphical abstract

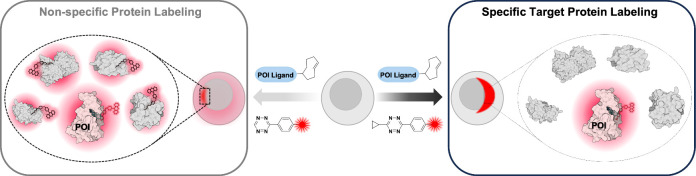

## 1 Introduction

1,2,4,5-Tetrazine derivatives have been widely used for the bio-orthogonal click reaction with cycloalkenes such as cyclopropene and *trans*-cyclooctene (TCO).^1,2^ Compared to copper-mediated alkyne–azide click chemistry, tetrazine–cycloalkene click chemistry offers catalyst-free and remarkably fast reaction kinetics with *k* ∼ 1 × 10^6^ M^–1^ s^–1^.^3^ Considering its bio-orthogonality and fast reaction rate, tetrazine has been used for various biological applications including bioimaging, bioconjugations, and therapeutic applications *in vitro* and *in vivo*.^4–7^ For those successful bioapplications of the tetrazine–cycloalkene click reaction, a number of tetrazine derivatives have been developed to improve their specificity and reactivity in biological systems.^8–10^

During the development of tetrazine derivatives for efficient bio-orthogonal click reactions, structural instability of tetrazine in physiological conditions has been reported. In a number of the reports, steric hindrance and electronic density of the substituent on the 3- and 6-position of 1,2,4,5-tetrazine have been reported to be critical for the bio-orthogonality of tetrazine toward cycloalkene.^10–12^ For instance, Hilderbrand et al. measured the serum stability of tetrazine derivatives and reported the decomposition of tetrazines in the presence of FBS.^13^ Fox et al. reported the reactivity of tetrazine derivatives toward nucleophiles (BuSH and BuNH_2_)^2^ and structure-dependent decomposition of tetrazine derivatives in PBS and cell culture media.^14^ Ting et al. reported the structure-dependent fluorescent background signal of the tetrazine–fluorescein conjugate during the fluorescent protein labeling experiments.^15^ Johnson et al. reported background protein labeling by tetrazine.^5^ However, an in-depth understanding of the reactions between tetrazine and biomolecules is barely reported.

Herein, we discovered the structure-dependent proteome reactivity of tetrazine derivatives and their attribution to bio-orthogonal fluorescent protein imaging. Inspired by previous reports on the reactivity of tetrazines with nucleophiles,^16–19^ we demonstrated that tetrazine can react with amines to generate a formohydrazonamide structure, which could explain the proteome reactivity of tetrazines. To discover a tetrazine derivative with minimal nonspecific proteome reactivity, we synthesized 23 tetrazine derivatives conjugated with a silicon-rhodamine (**SiR-Tz**s). Our study revealed the structure-dependent proteome reactivities of the tetrazine derivatives, which could explain the previously reported nonspecific fluorescent background signal associated with tetrazine-conjugated fluorophores (**FL-Tz**).^15,20^ Among the 23 different **SiR-Tz**s, silicon rhodamine conjugated 3-cyclopropyl-6-phenyl-1,2,4,5-tetrazine, **SiR-Tz20**, showed minimal proteome reactivity. With TCO-modified ibrutinib, a ligand for Bruton’s tyrosine kinase (BTK), **SiR-Tz20** successfully and selectively labeled endogenous BTK in live cells and *ex vivo*. Considering the increasing interest in developing robust bio-orthogonal click chemistry in live cells and *in vivo*,^21^ our discovery could significantly enhance the accuracy and efficiency of the tetrazine-based click reaction, especially in live cells.

## 2 Results and Discussion

### Investigation of Proteome Reactivity of Tetrazine

On our endeavors to label fluorescent protein with the inverse electron demand Diels–Alder (iEDDA) bio-orthogonal click reaction, we serendipitously discovered the proteome reactivity of tetrazine. 1,2,4,5-Tetrazine (**Tz**) derivatives showed the nonspecific labeling of multiple proteins (data not shown). Considering previous reports on the proteome reactivity of heterocycles, electrophiles,^22–26^ molecules previously considered bioorthogonal,^24,27,28^ and nonspecific protein band signals by tetrazine-fluorophore conjugates,^5^ we hypothesized that **Tz** could react with the proteome through a specific reaction mechanism. To confirm that the nonspecific labeling was caused by **Tz** rather than the fluorophore, three different **FL-Tz**s (**TAMRA-Tz**, **Cy5-Tz**, and **SiR-Tz1)** were prepared (Figure 1A) and tested for their proteome reactivity (Figure 1B). The **TAMRA-Tz**, **Cy5-Tz**, and **SiR-Tz1** were incubated with the cell lysates, and resulting mixtures were analyzed by SDS-PAGE, followed by fluorescent gel scanning. The scanning result showed that all of **TAMRA-Tz**, **Cy5-Tz**, and **SiR-Tz1** exhibited nonspecific proteome labeling. However, no proteome labeling patterns was observed with **TAMRA-Bn**, **Cy5-Bn**, and **SiR-Bn**. This result indicates that the structural and charge characteristics of the fluorophores did not result in any significant differences, confirming that proteome labeling by **FL-Tz**s is dependent on the tetrazine moiety. Considering previous reports about the stability of tetrazine derivatives and their reaction with nucleophiles, such as amine, thiol, and water, we investigated the reaction of **Tz** with amines to better understand the proteome labeling event by tetrazine.^2,13,15,16^ We incubated **Boc-Tz1** with diethyl amine for 2 h at room temperature and isolated a conjugated product. A series of NMR and MS studies revealed that the product is *N*-methylformo­hydrazonamide (**MFHA**, Figures 1C and S2–S7). We also monitored the reaction between **Boc-Tz1** and thioethanol, which generated *N*-methylenemethane­hydrazonothioate (Figures S8 and Figure S9). Moreover, **Boc-Tz1** can react with lysine not only in THF but also in PBS (Figure S10 and Figure S11). We believe that these observations suggest the possibility of reaction between **Tz** and the lysine or cystein residue of proteins, which would be one of the reasons for the nonspecific proteome labeling by **FL-Tz** (Figure 1D). To identify the protein labeling event by **Tz**, we first measure the stability of **MFHA** in PBS. LC-MS analysis of **MFHA** showed its stability up to 3 h in PBS (Figure S12), which suggests that protein labeling by **Tz** could be detected in a proteomics study. In the following mass spectrometry analysis, bovine serum albumin (BSA) was incubated with **SiR-Tz1**, followed by tryptic digestion. A tryptic peptide of BSA that contains Cys268 and Cys269 (amino acids 267–280 and 267–285) exhibited distinct proteome labeling by **SiR-Tz1** (Figure S13). These data clearly demonstrated that the nonspecific protein labeling by **FL-Tz** is due to the reaction between **Tz** and nucleophiles of proteins.

**Figure 1:**
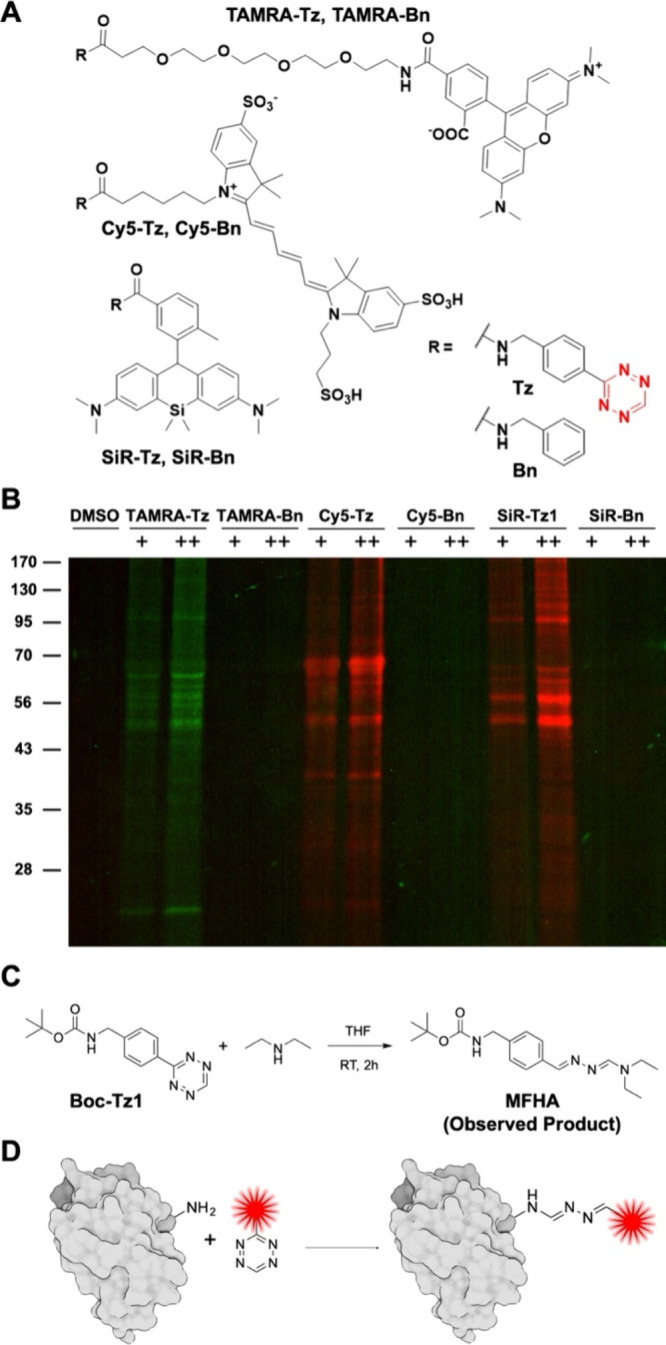
Proteome reactivity of tetrazine. **A)** Chemical structures of fluorescent-conjugated tetrazines (**FL-Tz**) for studying their proteome reactivities. Silicon rhodamine-, TAMRA-, and Cy5-conjugated benzene (**SiR-Bn**, **TAMRA-Bn**, and **Cy5-Bn**) were synthesized as control compounds. **B)** Proteome reactivity of **FL-Tz**. HeLa cell lysates were incubated with **FL-Tz** for 30 min (+: 5 μM, + +: 10 μM) and analyzed by SDS-PAGE, followed by fluorescent gel scanning. **C)** Reaction of tetrazine (**Boc-Tz1**) with a nucleophile, diethyl amine. Nucleophilic attack of tetrazine by diethyl amine resulted in *N*-methylformo­hydrazonamide (**MFHA**) with 40.8% yield. **D)** Schematic presentation of reaction between amine of proteins and **FL-Tz**.

### Proteome Reactivity of 23 Tetrazine Derivatives

Considering the reaction between **Tz** and amine, we envisioned that the proteome reactivity of **Tz** derivatives would be dependent on their structure. Since the reactivities of **Tz** derivatives are dependent on the structure and electronic properties of the substitution group on 3,6-positions, 23 **Tz** derivatives were designed to functionalize with diverse structures (e.g., cyclic ring, aliphatic chain, or highly substituted tertiary carbon group) and electronic properties (e.g., electron-withdrawing or electron-donating group). To find a minimal proteome reactive **Tz**, we further conjugated 23 **Tz** with silicon rhodamine (**SiR-Tz**s) (Figure 2A). To monitor the proteome reactivity of **SiR-Tz**s, we incubated the compounds with HeLa cell lysates for 30 min and analyzed them by SDS-PAGE, followed by fluorescent gel scanning. Intriguingly, most of **SiR-Tz** showed proteome reactivity in a dose-dependent manner (Figure 2B). Compared to **SiR-Tz1**, we found that the substitution on the 3-position of **Tz** reduced the proteome activity of **SiR-Tz**s (**SiR-Tz2**, **SiR-Tz3**, and **SiR-Tz4**). Benzene substitution on the 6-position of **Tz** did not affect the proteome reactivity of Tzs (**SiR-Tz2** vs **SiR-Tz6**, **SiR-Tz4** vs **SiR-Tz8**). When the substitution group on the 3-position of **Tz** became bulkier (**SiR-Tz10** < **SiR-Tz11** < **SiR-Tz12** < **SiR-Tz19** < **SiR-Tz23**), their proteome reactivity was decreased. The most sterically hindered **SiR-Tz23** showed the minimal proteome reactivity among all the **SiR-Tz**s. When the aliphatic chain length on the 3-position of Tz was increased from a methyl to a butyl group, their proteome reactivity was remarkably increased (**SiR-Tz2** < **SiR-Tz10** < **SiR-Tz16** < **SiR-Tz15**). This result indicates that the hydrophobicity of **Tz** could affect its proteome reactivity. Moreover, the cyclic alkyl group on the 3-position of **Tz** was critical for the proteome reactivity of **SiR-Tz**s. **SiR-Tz17** and **SiR-Tz18** showed the highest proteome reactivity. In general, when the size of the cyclic ring was decreased, proteome labeling by **SiR-Tz** was reduced. From the quantification of the fluorescent gel scanning results, we could categorize **SiR-Tz**s into four groups: highest proteome reactive **SiR-Tz**s (**SiR-Tz15**, **SiR-Tz17**, **SiR-Tz18**), moderate proteome reactive **SiR-Tz**s (**SiR-Tz1**, **SiR-Tz7**, **SiR-Tz9**, **SiR-Tz10**, **SiR-Tz16**, **SiR-Tz21**), low proteome reactive **SiR-Tz**s (**SiR-Tz3**, **SiR-Tz4**, **SiR-Tz5**, **SiR-Tz13**, **SiR-Tz14**), and minimal proteome reactive **SiR-Tz**s (**SiR-Tz2**, **SiR-Tz6**, **SiR-Tz8**, **SiR-Tz11**, **SiR-Tz12**, **SiR-Tz19**, **SiR-Tz20**, **SiR-Tz22**, **SiR-Tz23**) (Figure S16). To clarify that the nonspecific labeling of the proteome by **SiR-Tz**s is not attributable to their chemical instability, we measure the stability of **SiR-Tz**s in PBS. LC-MS analysis showed that most **SiR-Tz**s are stable in PBS, which supports that proteome labeling by **Tz**s does not result from the reactive decomposition products of **Tz**s (Figure S18). We also monitored the time-dependent proteome labeling by **SiR-Tz**s. The protein labeling by **SiR-Tz**s was increased with a longer incubation time (Figure S19).

**Figure 2:**
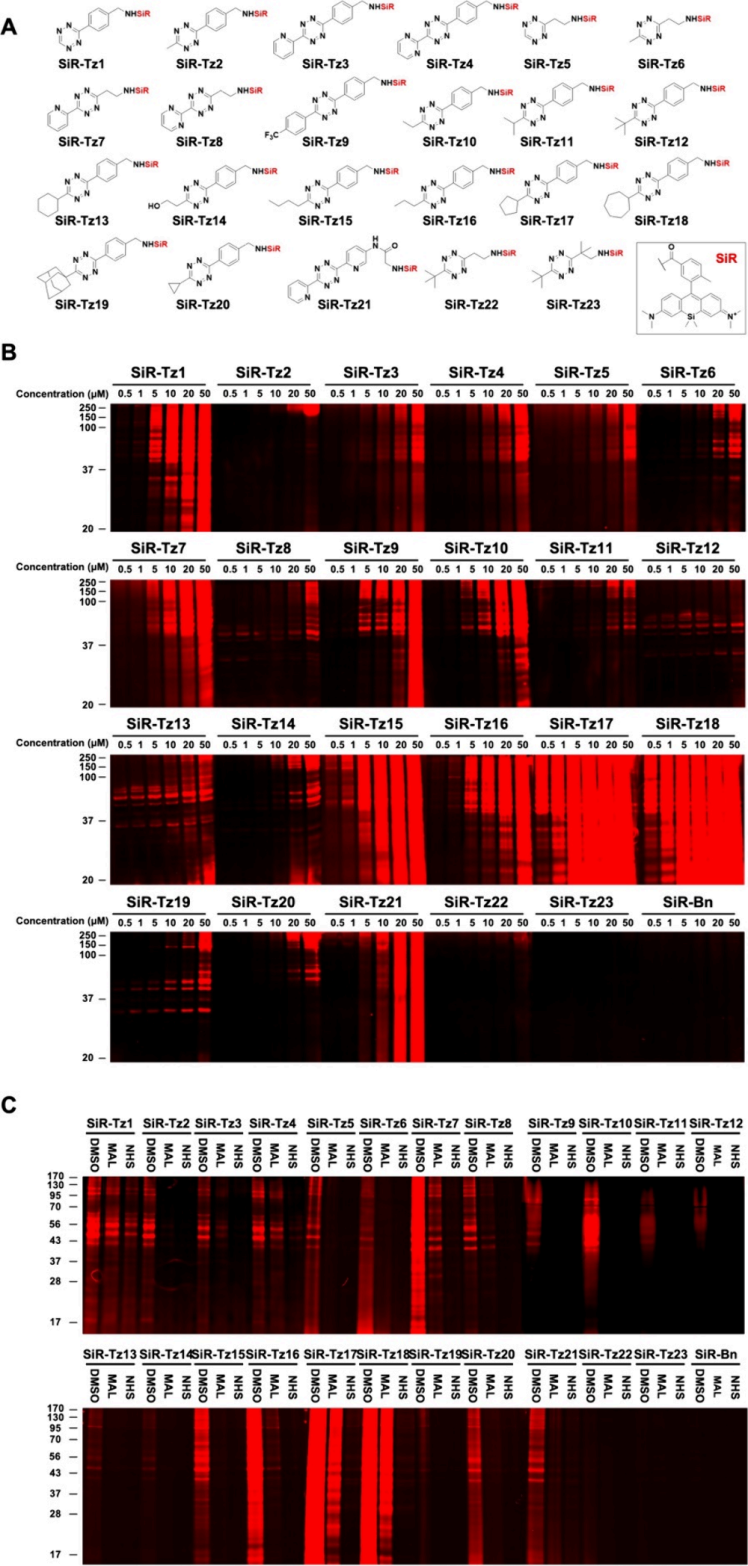
Proteome reactivity of tetrazine derivatives. **A)** Structure of 23 silicon rhodamine conjugated tetrazine derivatives (**SiR-Tz**s). **B)** Proteome reactivity of 23 tetrazine derivatives. HeLa cell lysate was incubated with various concentrations of **SiR-Tz**s and **SiR-Bn** for 30 min. After SDS-PAGE, the proteome reactivity of 23 tetrazine derivatives was analyzed by fluorescent gel scanning. Tetrazine-dependent proteome reactivity was observed. **C)** Perturbation of proteome reactivities of **SiR-Tz**s by 1 mM maleimide (MAL) or 1 mM *N*-hydroxysuccinimide ester (NHS). After protection of nucleophiles (amine and thiol) of the proteome by either MAL or NHS, the resulting cell lysates were incubated with **SiR-Tz**s (50 μM) for 30 min. After SDS-PAGE, the proteome reactivity of **SiR-Tz**s was analyzed by fluorescent gel scanning.

Our next question was whether the proteome labeling by **SiR-Tz**s is attributed to the reaction between **Tz** and nucleophiles of the proteome or not. We first masked the nucleophiles (thiol or amine) of the proteome either by maleimide or *N*-hydroxysuccinimide ester (NHS ester) and then treated **SiR-Tz**s. Subsequent fluorescent gel scanning showed reduced proteome labeling by all **SiR-Tz**s (Figure 2C). In most cases, masking amine functional groups of the proteome by NHS ester significantly perturbed proteome reactivities of **SiR-Tz**s. We also observed that masking thiol functional groups with maleimide mildly reduced the proteome reactivities of **SiR-Tz**s, indicating that both amine and thiol groups in the proteome could react with **Tz**. These results suggested that the reaction between **Tz** and nucleophiles of the proteome is the main reason for the proteome reactivities of **Tz** derivatives.

### Discovery of **SiR-Tz11**, **SiR-Tz13**, **SiR-Tz14**, **SiR-Tz16**, and **SiR-Tz20** as Minimal-Background Reagents for *in Situ* **Tz-TCO** Click Chemistry Mediated Fluorescent Protein Labeling in Live Cells

After monitoring the proteome reactivities of **SiR-Tz**s, we wondered whether the proteome reactivity of **Tz** could affect the click reaction efficiency between **Tz** and TCO in a biological system. Since efficient and accurate bio-orthogonal chemistry in live cells or *in vivo* is critical for the exact monitoring of a protein of interest,^21^ we decided to monitor Bruton’s tyrosine kinase using an *in situ* click reaction for proof-of-concept validation. For this purpose, we functionalized ibrutinib, a BTK ligand, by conjugating TCO (IBR-TCO) for the fluorescent labeling of BTK, which is highly expressed in B cells.

In previous studies, only the overexpressed BTK protein was successfully monitored with **Tz-TCO** click reaction-mediated fluorescent labeling using ibrutinib probes.^29–33^ We hypothesized that endogenous BTK was barely monitored with **Tz-TCO** click reaction-mediated fluorescent labeling due to the nonspecific proteome labeling of **FL-Tz**s. In this context, we envisioned that minimal proteome reactive **SiR-Tz** could be a useful tool for the monitoring of the endogenous level of proteins.

To visualize the endogenous level of BTK, we treated live cells with IBR-TCO and then performed *in situ* **Tz-TCO** click reaction-mediated fluorescent BTK labeling using **SiR-Tz**s in live cells. In this regard, we used BTK-positive HL60 cells and BTK-negative RPMI8226 cells and compared the fluorescent BTK labeling patterns of those cells. Along with fluorescent BTK protein bands (75 kDa, blue dotted rectangle with asterisk in Figure 3B), nonspecific proteome labeling is also observed with most of the **SiR-Tz**s as we previously observed in Figure 2B. **SiR-Tz1**, **SiR-Tz2**, **SiR-Tz15**, **SiR-Tz17**, and **SiR-Tz18** showed intensive nonspecific proteome reactivity in fluorescent gel scanning analysis and failed to label BTK selectively. **SiR-Tz3**, **SiR-Tz4**, **SiR-Tz5**, **SiR-Tz6**, **SiR-Tz7**, **SiR-Tz8**, **SiR-Tz9**, **SiR-Tz10**, and **SiR-Tz21** showed a clear BTK labeling pattern along with additional nonspecific protein band labeling at 60 kDa. **SiR-Tz11**, **SiR-Tz13**, **SiR-Tz14**, **SiR-Tz16**, and **SiR-Tz20** (orange dotted rectangles in Figure 3B) showed a BTK-specific labeling pattern only in BTK­(+) HL60 cells, not in BTK(−) RPMI8226 cells.

**Figure 3:**
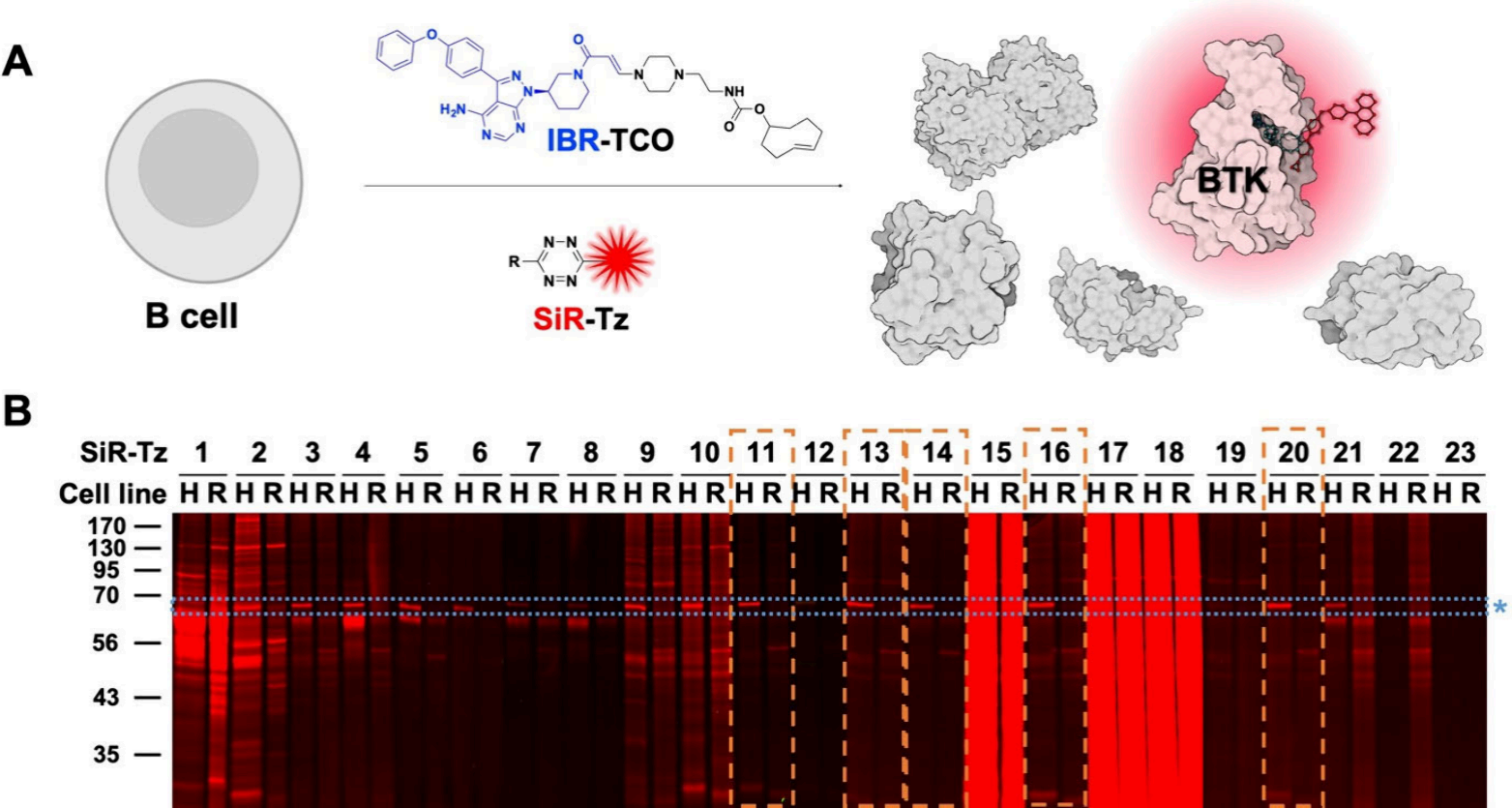
Fluorescent BTK protein labeling with ibrutinib-*trans*-cyclooctene (IBR-TCO) and **SiR-Tz**s in live cells. **A)** Scheme of BTK protein labeling using IBR-TCO and **SiR-Tz**s in live cells. **B)** BTK fluorescent labeling by IBR-TCO and **SiR-Tz**s via an *in situ* click reaction in live BTK­(+) HL60 cells and BTK(−) RPMI8226 cells. Both cell lines were treated with IBR-TCO (10 μM) for 1 h, followed by the treatment of **SiR-Tz**s (10 μM) for 30 min in live cells (H: HL60, R: RPMI8226). Selective BTK labeling in HL60 was observed with **SiR-Tz11**, **SiR-Tz13**, **SiR-Tz14**, **SiR-Tz16**, and **SiR-Tz20** (yellow dotted rectangles). Endogenous BTK bands are marked as a blue dotted rectangle with an asterisk.

In contrast to endogenously expressed BTK, overexpressed BTK protein was readily detected even when IBR-TCO and highly proteome reactive **SiR-Tz**s were used, such as **SiR-Tz1** and **SiR-Tz2** (Figure S26). Considering the intensive fluorescent labeling of overexpressed BTK by IBR-TCO and **SiR-Tz**s—attributable to its high abundance within the cellular proteome—nonspecifically labeled protein bands by **SiR-Tz**s can be neglected in fluorescent gel scanning analysis. However, endogenous proteins are not dominantly expressed compared with other proteins in cells. Therefore, nonspecific protein labeling by **SiR-Tz** can interfere with the fluorescence imaging of endogenously expressed proteins with *in situ* **Tz-TCO** click chemistry. From these results, we found that the endogenous level of BTK can be only specifically and precisely imaged with low or minimal proteome reactive **SiR-Tz**s in live cells.

Our results demonstrated that proteome reactivity of tetrazine derivatives is critical for fluorescent labeling of endogenous proteins with *in situ* **Tz-TCO** click chemistry in live cells. The highest or moderate proteome reactive **Tz**s showed nonspecific protein labeling bands, which interfered with the selective labeling of the protein of interest in *in situ* **Tz**-**TCO** chemistry-mediated fluorescent protein labeling. On the contrary, low or minimal proteome reactive **Tz**s showed desired protein-selective fluorescent labeling patterns, which is very crucial for the imaging of endogenous proteins with low expression levels.

### Discovery of **SiR-Tz20** as the Best Minimal Background Reagent for *in Situ* Tz-TCO Click Reaction Mediated Fluorescent Protein Monitoring in Live Cells

After identifying **SiR-Tz11**, **SiR-Tz13**, **SiR-Tz14**, **SiR-Tz16**, and **SiR-Tz20** as exhibiting low nonspecific proteome reactivity through fluorescent gel scanning analysis, we next evaluated their potential for cellular BTK imaging in live cells. To assess BTK at the cellular level, we used BTK-positive THP-1 monocytes (Figure S27), primed with phorbol 12-myristate 13-acetate (PMA) to induce an adherent state for imaging. The live THP-1 cells were sequentially treated with IBR-TCO and **SiR-Tz**s to image the BTK protein with *in situ* **Tz-TCO** click chemistry-mediated fluorescent protein labeling. In parallel, the BTK protein was visualized by immunofluorescence using a BTK-specific antibody.

The colocalization between BTK immunostaining and *in situ* **Tz-TCO** click chemistry-mediated fluorescent BTK labeling, using IBR-TCO and **SiR-Tz**s, showed intriguing results and identified the best minimal background reagent for *in situ* **Tz-TCO** click reaction-mediated fluorescent protein labeling (Figure 4A and Figure S28). Due to their nonspecific proteome reactivities, *in situ* **Tz-TCO** click chemistry-mediated fluorescent BTK labeling using **SiR-Tz1** and **SiR-Tz2** displayed low colocalization efficiency with BTK immunostaining (*R* = 0.70 and 0.42, respectively). **SiR-Tz11**, **SiR-Tz13**, and **SiR-Tz16** showed moderate colocalization efficiency (*R* = 0.73–0.82), while **SiR-Tz14** demonstrated poor colocalization efficiency (*R* = 0.31). Among the tested **SiR-Tz**s, **SiR-Tz20** exhibited the most specific BTK labeling with the highest colocalization efficiency (*R* = 0.91, Figure 4A). The BTK-specific labeling using **SiR-Tz20** was further confirmed by a fluorescent line intensity profile, which demonstrated an almost complete match between BTK immunostaining and *in situ* **Tz-TCO** click chemistry-mediated fluorescent BTK labeling using IBR-TCO and **SiR-Tz20** (Figure 4B).

**Figure 4:**
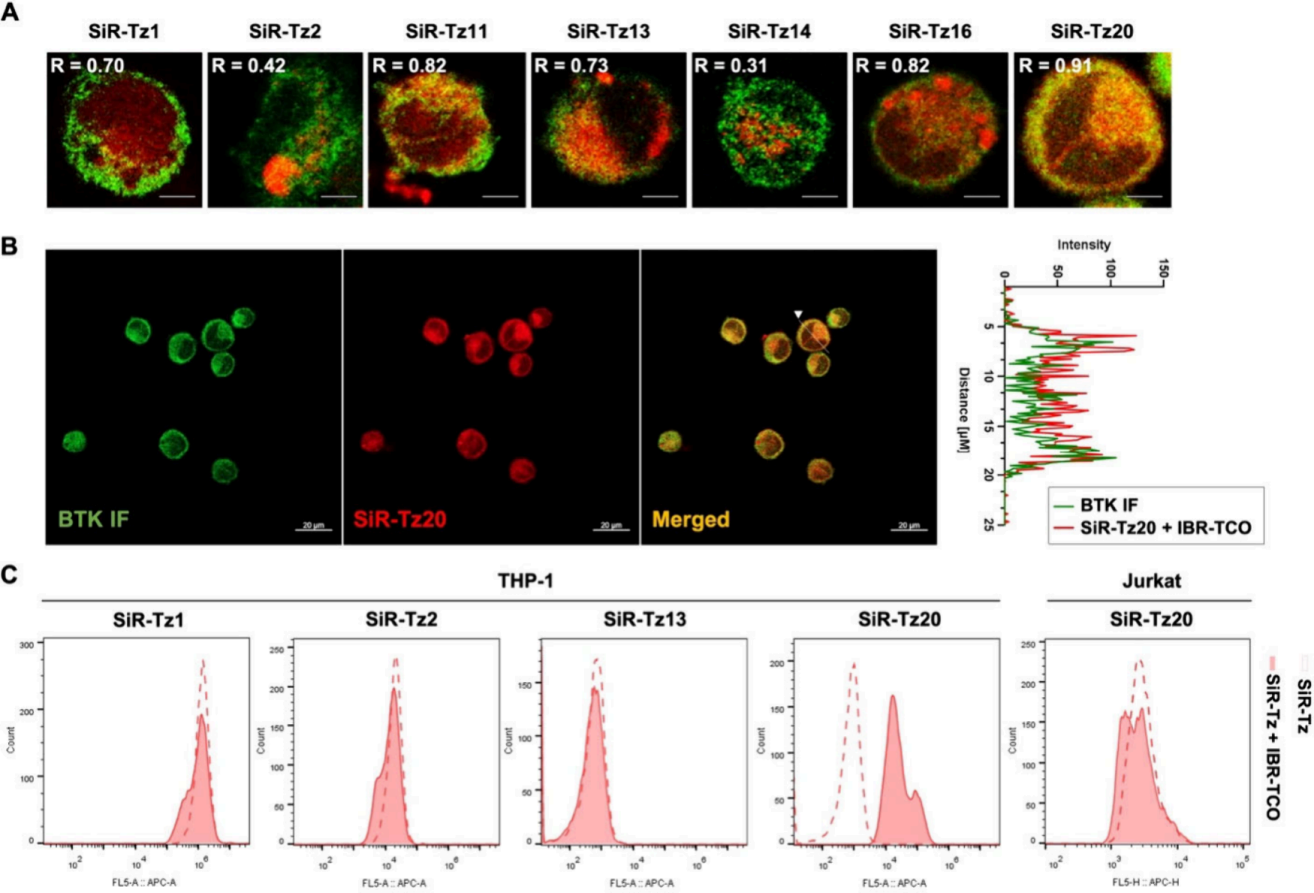
Cellular monitoring of BTK protein using immunostaining and *in situ* **Tz-TCO** click chemistry-mediated fluorescent protein labeling with IBR-TCO and **SiR-Tz**s in live cells. **A)** Merged images of BTK immunostaining (green) and *in situ* **Tz-TCO** click chemistry-mediated fluorescent BTK labeling (red) using IBR-TCO and seven **SiR-Tz**s in PMA-primed THP-1 cells. PMA-primed THP-1 cells were treated with IBR-TCO (10 μM) for 20 min, followed by treatment with **SiR-Tz**s (10 μM) for 20 min in live cells. *R* values denote the colocalization efficiency between BTK immunostaining and *in situ* **Tz-TCO** click chemistry-mediated BTK fluorescent labeling using IBR-TCO and **SiR-Tz**s. Scale bars represent 5 μm. B) Left panels: Scaled-down view of BTK colocalization from **A)** for **SiR-Tz20**. BTK was labeled via immunofluorescence (green), *in situ* **Tz-TCO** click chemistry-mediated fluorescent protein labeling (red, IBR-TCO and **SiR-Tz20**), and a merged image in PMA-primed THP-1 cells (green and red). Scale bars are 20 μm. Right panel: Fluorescent line intensity profile comparing immunofluorescence and *in situ* **Tz-TCO** click chemistry-mediated fluorescent protein labeling using IBR-TCO and **SiR-Tz20** of a cell, as indicated by the white arrowhead in the merged image in the left panel. **C)** Flow cytometry analysis of *in situ* **Tz-TCO** click chemistry-mediated fluorescent BTK labeling using IBR-TCO and **SiR-Tz**s (**SiR-Tz1**, **SiR-Tz2**, **SiR-Tz13**, and **SiR-Tz20**, four left panels) in THP-1 cells and using IBR-TCO and **SiR-Tz20** in Jurkat cells (right panel). Solid-filled histograms represent cells labeled using IBR-TCO and **SiR-Tz**s, while dashed lines represent cells labeled using indicated **SiR-Tz** only. THP-1 cells and Jurkat cells were treated with IBR-TCO (1 μM) for 20 min, followed by **SiR-Tz**s (10 nM) treatment for 20 min in live cells.

To further confirm BTK-specific fluorescent labeling via *in situ* **Tz-TCO** click chemistry under endogenous conditions in a quantitative manner, we conducted flow cytometry using IBR-TCO and **SiR-Tz**s in live THP-1 cells without priming. Consistent with the cellular imaging results, **SiR-Tz20** and IBR-TCO showed the most specific BTK labeling via *in situ* **Tz-TCO** click chemistry in BTK-positive THP-1 cells, but not in BTK-negative Jurkat cells (Figure 4C). These findings suggest that **SiR-Tz20** is a valuable bio-orthogonal tool for *in situ* **Tz-TCO** click chemistry-mediated fluorescent protein labeling in biological systems, offering minimal background noise and high target specificity.

### *Ex Vivo* Fluorescent BTK Monitoring with *in Situ* **Tz-TCO** Click Chemistry Using IBR-TCO and **SiR-Tz20**

After identifying **SiR-Tz20** as a minimally proteome-reactive and highly selective *in situ* **Tz-TCO** click reagent through extensive *in vitro* validation, we next demonstrated its application for staining BTK-positive B cells in an *ex vivo* system (Figure 5A). Mouse splenocytes obtained from Balb/c mice were incubated with either **SiR-Tz20** or **SiR-Tz1**, in the presence or absence of IBR-TCO and subsequently immunostained with the anti-CD19 antibody (for B cell populations) or anti-CD3 antibody (for T cell populations) for the flow cytometry analysis. Compared with **SiR-Tz1**, **SiR-Tz20** clearly exhibited a significant fluorescence intensity in the presence of IBR-TCO in CD19-positive B cells. Co-treatment with IBR-TCO and **SiR-Tz20** resulted in a rightward shift in mean fluorescent intensity (MFI), compared to the **SiR-Tz20** treatment alone. This observation supports the fact that IBR-TCO and **SiR-Tz20** efficiently labeled BTK-positive cells within the CD19-positive B cell population (Figure 5B, left panel).

**Figure 5:**
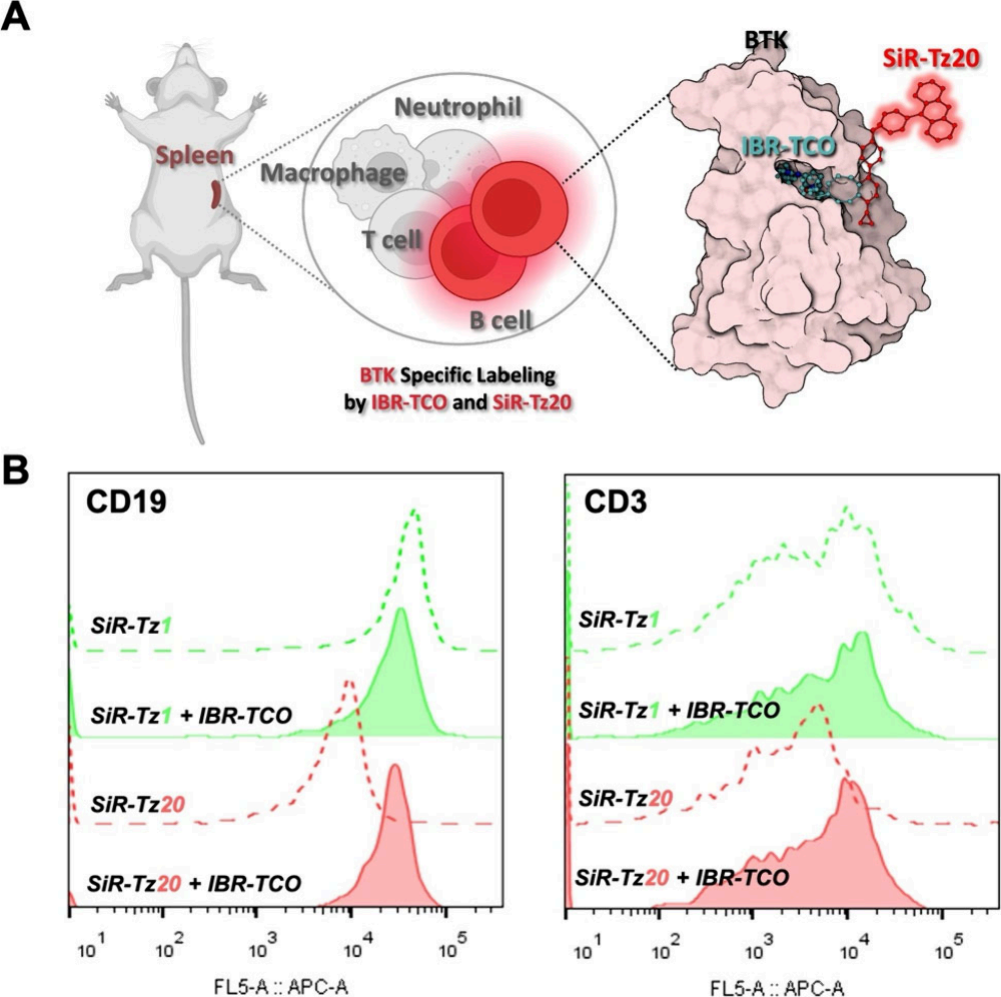
*in situ* **Tz-TCO** click chemistry-mediated fluorescent BTK-specific labeling using IBR-TCO and **SiR-Tz20** in an *ex vivo* system. **A)** Schematic representation of the *ex vivo* study: Mouse spleens were harvested to isolate B and T cells. The cells were treated with IBR-TCO and either **SiR-Tz1** or **SiR-Tz20**, followed by flow cytometry analysis. **B)** Flow cytometry analysis of *in situ* **Tz-TCO** click chemistry-mediated fluorescent BTK labeling using IBR-TCO and either **SiR-Tz1** or **SiR-Tz20**. B cells and T cells were treated with IBR-TCO (1 μM) for 20 min, followed by treatment with **SiR-Tz**s (100 nM) for 20 min in live cells. Solid-filled histograms represent cell populations labeled with both IBR-TCO and **SiR-Tz**s, while dashed lines represent cells labeled only with the respective **SiR-Tz**. CD19-positive splenocytes (B cell populations, left) and CD3-positive cells (T cell populations, right) are shown with the *y*-axis normalized.

To validate the specificity of BTK labeling, we compared the MFI of **SiR-Tz20** in the presence or absence of IBR-TCO in CD3-positive T cells, which are known to be BTK-negative.^34^ As expected, no significant difference in MFI was observed between cotreatment of IBR-TCO and **SiR-Tz20** and treatment of **SiR-Tz20** alone in the CD3-positive T cell population (Figure 5B, right panel). In contrast, **SiR-Tz1** failed to selectively label BTK protein and did not show a significant difference in MFI in either CD19-positive or CD3-positive cell populations, likely due to its nonspecific proteome reactivity. These findings demonstrate that **SiR-Tz20**, with its minimal proteome reactivity, is a valuable tool for selective *in situ* **Tz-TCO** click chemistry-mediated fluorescent protein labeling in both *in vitro* and *ex vivo* mouse models.

### Fluorescent XPO1 Monitoring with *in Situ* **Tz-TCO** Click Chemistry Using SEL-TCO and **SiR-Tz20**

After identifying **SiR-Tz20** as a minimally proteome-reactive and highly selective *in situ* **Tz-TCO** click reagent through extensive *in vitro* and *ex vivo* validation, we next demonstrated its application for the fluorescent labeling of XPO-1 protein (Figure S29A). Based on a previously reported XPO1 probe, Selinexor-yne,^35^ we designed selinexor-*trans*-cyclooctene (SEL-TCO) for the fluorescent imaging of XPO1 with *in situ* **Tz-TCO** click chemistry (Scheme S5). To label the endogenous level of XPO1, we used SEL-TCO for *in situ* **Tz-TCO** click reaction-mediated fluorescent XPO1 labeling with **SiR-Tz**s in live SH-SY5Y cells. In fluorescent gel scanning, the minimal-background reagents (**SiR-Tz11**, **SiR-Tz13**, **SiR-Tz14**, **SiR-Tz16**, and **SiR-Tz20**) exhibited successful XPO1 labeling in SH-SY5Y (Figure S29B, yellow dotted rectangles). Among them, **SiR-Tz20** showed the most selective XPO1 labeling in SH-SY5Y.

We further demonstrated **SiR-Tz20** for the fluorescent cellular imaging of XPO1. In the absence of SEL-TCO, **SiR-Tz1** showed nonspecific fluorescence cellular labeling, while **SiR-Tz20** showed low background fluorescent cellular labeling (Figure S29D). The colocalization between the XPO1 immunostaining image and fluorescent XPO1 labeling using SEL-TCO and **SiR-Tz**s showed again that **SiR-Tz20** is the best minimal background reagent for the *in situ* **Tz-TCO** click reaction (Figure S29E). Compared to **SiR-Tz1**, **SiR-Tz20** and SEL-TCO successfully stained XPO1, which showed a high level of colocalization with the XPO1 immunostaining image.

### Fluorescent Mitochondria Monitoring with *in Situ* **Tz-TCO** Click Chemistry Using TPP-TCO and **SiR**-**Tz20**

Our final validation was the application of **SiR-Tz20** for fluorescent cellular organelle imaging with the *in situ* **Tz-TCO** click reaction (Figure S30A). We designed a mitochondria probe, TPP-TCO, by conjugating the well-known mitochondria-targeting moiety triphenylphosphonium with TCO (Scheme S6).^36^ We treated mitoYFP-overexpressing HeLa cells with TPP-TCO, followed by the treatment of **SiR-Tz**s in live cells. In the absence of TPP-TCO, **SiR-Tz1** showed nonspecific fluorescence cellular labeling, while **SiR-Tz20** showed low background fluorescent cellular labeling (Figure S30B). The colocalization between the mitoYFP image and fluorescent mitochondria labeling using TPP-TCO and **SiR-Tz**s showed again that **SiR-Tz20** is the minimal background reagent for the *in situ* **Tz-TCO** click reaction (Figure S30B and S30C). With this result, we could demonstrate that **SiR-Tz20** is the best minimal background reagent for *in situ* **Tz-TCO** click reaction-mediated fluorescent protein and cellular organelle monitoring in live cells.

## Conclusion

In this study, we reported the structure-dependent proteome reactivity of **Tz** derivatives systematically for the first time. We found that the thiol and amine functional groups of the proteome are critical for the proteome reactivity of **Tz** derivatives. We further investigated whether the proteome reactivity of **Tz** derivatives could affect the specificity of *in situ* **Tz-TCO** click chemistry-mediated fluorescent protein labeling. IBR-TCO was synthesized and applied for *in situ* **Tz-TCO** click chemistry-mediated fluorescent BTK imaging with 23 **SiR-Tz** conjugates. We found that selective *in situ* fluorescent BTK labeling was achieved with IBR-TCO and minimal proteome reactive **SiR-Tz**s in fluorescent gel scanning. With cellular BTK imaging *in vitro* and *ex vivo* experiments, **SiR-Tz20** was discovered as the most minimal proteome reactive **SiR-Tz** for *in situ* **Tz-TCO** click chemistry-mediated fluorescent protein labeling and imaging. We believe that the cyclopropyl group of **SiR-Tz20** introduces greater steric hindrance than a proton or a simple alkyl substituent, thereby restricting the access of protein nucleophiles. For this reason, we suggest that **SiR-Tz20** exhibits lower background reactivity compared to **SiR-Tz1** or **SiR-Tz2**. However, when the 3- or 6-position of the tetrazine is heavily substituted, as in **SiR-Tz22** or **SiR-Tz23**, the resulting substantial steric hindrance limits not only the accessibility of protein nucleophiles but also that of TCO, ultimately leading to failure in the desired bioorthogonal reactions. Therefore, we propose that moderate steric hindrance on the tetrazine is essential for achieving specific and efficient bioorthogonal chemistry.

Overall, our results suggested that proteome reactivity of **Tz** is critical for accurate and selective *in situ* **Tz-TCO** click reactions in live cells. Although we demonstrated that the fluorophore structure is not critical for the proteome reactivities of **Tz**s in the proof-of-concept study, further validation is still needed by conjugating more various fluorophores with **Tz**. We believe this further fluorophore validation would lead to the development of the ideal **FL-Tz** for excellent bio-orthogonal **Tz-TCO** reactions in live cells and *in vivo*. Collectively, we believe our discovery can improve the accuracy and efficiency of the tetrazine-based click reaction especially in live cells, thereby broadening the application of **Tz-TCO** chemistry in various biological contexts and expanding our knowledge of the biological systems more precisely.

## Experimental Section

### Organic Materials and Synthetic Methods

All chemical reagents for synthesis were purchased from Sigma-Aldrich, Alfa Aesar, Acros Organics, and TCI. All reactions were conducted in oven-dried glassware under a dry argon atmosphere, unless otherwise specified. Yields of the synthesized compounds were measured after chromatographic purification. Analytical thin-layer chromatography (TLC) was carried out on silica 60F-254 plates. The spots were visualized by UV light (254 and 365 nm) and/or by potassium permanganate, PMA, and ninhydrin staining. Flash column chromatography was carried out using silica gel (38–75 μm). Reverse-phase column chromatography was performed on C18 silica gel (75 μm) with MPLC (Biotage Selekt System) or C18 columns (YMC-Actus Triart C18, 250 × 20 mm, 5 μm) with HPLC. HPLC semipreparative purifications were conducted using an HPLC system (Waters; 1525 binary HPLC pump and 2489 UV/vis detector) controlled by Empower 3 software. The solvent system consisted of 0.1% trifluoroacetic acid (TFA) in H_2_O as buffer A and acetonitrile (MeCN) as buffer B. ^1^H and ^13^C NMR spectra were recorded on JEOL (400 MHz) and Bruker (400 MHz) instruments. Chemical shifts (δ) are reported relative to TMS and are referenced to the residual proton signal in the deuterated solvents: chloroform-*d* (7.26 ppm), methanol-*d*_4_ (3.31 ppm), acetonitrile-*d*_3_ (1.94 ppm), acetone-*d*_6_ (2.05 ppm) for ^1^H NMR spectra, chloroform-*d* (77.16 ppm), methanol-*d*_4_ (49.00 ppm), acetonitrile-*d*_3_ (1.32 ppm), acetone-*d*_6_ (29.84 ppm) for ^13^C NMR spectra. *J* values are given in hertz, and the splitting patterns are designated as follows: s; singlet; s, br; broad singlet; d, doublet; t, triplet; m, multiplet. Low-resolution mass spectrometry (LRMS) of **MFHA** was confirmed by a QTRAP 4500 LC-MS/MS spectrometer (AB SCIEX) from Central Laboratory at Kangwon National University. High-resolution mass spectrometry (HRMS) of the final compound was confirmed by a Xevo G2-XS QTof high-resolution LC-MS/MS spectrometer (Waters) from Korea Basic Science Institute (KBSI) at Kangwon National University.

### The Rate Constant of the IEDDA Reaction between 5-OH-TCO and Tz Derivatives

The second-order rate constant of the IEDDA reaction between 5-OH-TCO and Tz derivatives was determined by stopped-flow spectrometry under pseudo-second-order conditions. 5-OH-TCO and Tzs were dissolved in PBS:DMSO (1:1) solution. The absorption at 530 nm was measured every second in a time course manner. The data sets were analyzed by exponential fitting (one-phase association) using Prism 10 (GraphPad Software) to calculate the pseudo-first-order rate constants. The second-order rate constants were calculated by dividing the pseudo-first-order rate constants by the concentration of the excess 5-OH-TCO.

### Cell Culture and Reagents

THP-1 (human monocyte cell line) cells were purchased from Invivogen. Jurkat (human T lymphoblast cell lines), HeLa (human epithelial cell lines), HL60 (human promyeloblast cell lines), RPMI8226 (human B lymphocyte cell lines), and SH-SY5Y (human neuroblastoma cell lines) cells were purchased from KCLB. HeLa cells were cultured in RPMI1640 with l-glutamine (Welgene) containing 10% fetal bovine serum (FBS, Gibco) and 1% antibiotic–antimycotic (Gibco) at 37 °C in a humidified CO_2_ incubator. HL60 cells were cultured in IMDM with l-glutamine and 25 mM HEPES (Welgene) containing 10% FBS (Gibco) and 1% antibiotic–antimycotic (Gibco) at 37 °C in a humidified CO_2_ incubator. RPMI8226 cells were cultured in RPMI1640 with l-glutamine and 25 mM HEPES (Welgene) containing 10% FBS (Gibco) and 1% antibiotic–antimycotic (Gibco) at 37 °C in a humidified CO_2_ incubator. SH-SY5Y cells were cultured in DMEM with l-glutamine (Welgene) containing 10% FBS (Gibco) and 1% antibiotic–antimycotic (Gibco) at 37 °C in a humidified CO_2_ incubator. THP-1 cells were cultured in RPMI1640 with 2.05 mM l-glutamine (Hyclone) containing 10% FBS (Hyclone) and 1% penicillin (Corning) at 37 °C in a humidified CO_2_ incubator. Jurkat cells were cultured in RPMI1640 with high glucose and low l-glutamine (Gibco) containing 10% FBS (Hyclone) and 1% penicillin (Corning) at 37 °C in a humidified CO_2_ incubator.

### Proteome Labeling by **FL-Tz**s

HeLa cells (150 ϕ dish × 3) were detached and centrifuged. The supernatant was discarded, and the cell pellet washed with Ca^2+^- and Mg^2+^-free PBS (Welgene) and centrifuged. The supernatant was discarded, and the cell pellet was kept at −80 °C until use. RIPA buffer containing 50 mM Tris-HCl (pH 7.5), 150 mM NaCl, 1% Triton X-100, 1% sodium deoxycholate, 0.1% SDS, and a protease inhibitor cocktail (Thermo) was added to the cell pellets for lysis. The cells were incubated for 15 min on ice. The resulting cell lysate was centrifuged at 4 °C, 13000*g* for 5 min. The protein concentration of the cell lysate was measured with a BCA assay kit (Thermo), and the protein concentration was adjusted to 1 mg/mL. Proteins were treated with Cy5-Tz (Click Chmistry Tools, #1189), TAMRA-Tz (Click Chmistry Tools, #1196), or **SiR-Tz**s or **FL-Bn**s and incubated at room temperature for 30 min. In the case of the excess **SiR-Tz**s quenching experiment in Figure S17, the lysates were further incubated with 50 μM TCO-OH (Sigma) for 10 min. Then, 5× SDS loading buffer (Biosesang) was added and incubated at 90 °C for 5 min. The protein samples were analyzed by 12.5% SDS-PAGE and scanned with a Sapphire biomolecular imager (Azure Biosystems). The fluorescent protein band labeling was quantified using the Azure Spot program (Azure Biosystems). Gels were further stained with Coomassie Blue to confirm protein loading levels.

### Proteome Labeling by SiR-Tzs after Protection of Amine and Thiol

HeLa cells (150 ϕ dish × 3) were detached and centrifuged. The supernatant was discarded, and the cell pellet was washed with Ca^2+^- and Mg^2+^-free PBS and centrifuged. The supernatant was discarded, and the cell pellet was kept at −80 °C until use. RIPA buffer was added to the cells and incubated for 15 min on ice to induce lysis. The resulting cell lysate was centrifuged at 4 °C, 13000*g* for 5 min. The protein concentration of the cell lysate was measured with a BCA assay kit, and the protein concentration was adjusted to 1 mg/mL. The cell lysate was treated with 1 mM NHS-acetate or 1 mM *N*-methyl maleimide or DMSO to the desired concentration and incubated at room temperature for 30 min. For removing excess MAL or NHS, as shown in Figure S22, the lysates were further precipitated with cold acetone at −20 °C for 20 min, followed by centrifugation at 4 °C, 20000*g* for 7 min. The precipitated pellets were washed three times with cold acetone, dried, and resuspended in a RIPA buffer. Finally, 50 μM **SiR-Tz**s was then added to the cell lysate and incubated at room temperature for 30 min. A 5× SDS loading buffer was added and incubated at 90 °C for 5 min. The resulting samples were analyzed by 12.5% SDS-PAGE and scanned with a Sapphire biomolecular imager. The fluorescent protein band labeling was quantified using the Azure Spot program (Azure Biosystems). Gels were further stained with Coomassie Blue to confirm protein loading levels.

### Mass Spectrometry Study for Proteome Labeling by **FL-Tz**s

For proteome labeling between BSA and **FL-Tz**s, 20 μM BSA (Thermo) was incubated with 1 mM (50×) **SiR-Tz1** or **SiR-Tz17** 10% (v/v) DMSO in PBS for 30 min. After incubation, excess **FL-Tz**s was removed by precipitating BSA with cold acetone at −20 °C for 20 min, followed by centrifugation at 4 °C at 20000*g* for 7 min. The precipitated pellets were washed three times with cold acetone, dried, and resuspended in lysis buffer from the EasyPep Mini sample preparation kit (Thermo; A40006). Protein reduction, alkylation, digestion, and peptide cleanup were conducted according to the manufacturer’s instructions. Briefly, 100 μg of the BSA sample in 100 μL of lysis buffer was mixed with 50 μL of reduction solution and 50 μL of alkylation solution, with gentle mixing. Samples were heated at 95 °C for 10 min to reduce and alkylate the protein sample. After incubation, samples were cooled to room temperature. For digestion, 50 μL of the trypsin/Lys-C protease mix solution was added to the reduced and alkylated protein sample solution. The samples were incubated with shaking at 37 °C for 3 h to digest. After incubation, 50 μL of digestion stop solution was added to the sample and gently mixed followed by peptide cleanup. Following the peptide cleanup, the peptides were dried using an IR vacuum concentrator (N-Biotek; NB-503CIR). Dried peptides were desalted by a C18 StageTip (Supelco, #66883-U) and resuspended in 3% acetonitrile (ACN) (Sigma; #34851)/0.1% formic acid (FA) (Thermo Scientific, #85178) for mass spectrometry.

Mass spectrometry was performed by an Orbitrap Exploris 480 instrument coupled with a Vanquish Neo UHPLC system (Thermo Scientific). Peptides were loaded onto an Acclaim PepMap trap column (150 mm × 75 μm, particle size 3 μm, bed length 20 mm, Thermo Scientific, 164535) and separated on a C-18 reversed-phase (RP) μPAC Neo HPLC column (180 μm bed; 110 cm length; 2.5 × 16 μm pillar diameter, Thermo Scientific, COL-NANO110NEOB) with 2–4% (2–5.5 min), 4–20.5% (5.5–55.5 min), 20.5–33% (55.5–85.5 min), 33–90% (85.5–88.5 min), and 90% (88.5–103.5 min) gradient of mobile phase B (ACN/0.1% FA) at a 250 nL/min flow rate. FAIMS Pro Interface was used with a cycle type of 1.25 s/CV with FAIMS CV of – 45/–65 V). MS1 full scans were acquired by Orbitrap (resolution 60,000, 375–1500 *m*/*z*, automatic gain control (AGC) target 1 × 10^6^, maximum injection time 150 ms). Advanced peak determination algorithm was used, and precursor ions were isolated with a 2-Th window in the quadrupole and fragmented by a normalized collision energy (NCE) of 30%. Dynamic exclusion (±10 ppm) was applied for 24 s. MS2 spectra were analyzed with the Orbitrap (resolution 22,500, AGC 7.5 × 10^4^, maximum injection time 150 ms).

RAW files were analyzed by Proteome Discoverer3.1.0.638 (Thermo Scientific). The reference database was constructed from the albumin protein sequence of bovine downloaded from UniProt on February 12, 2025 followed by an appendage with common contaminants and reversed for target-decoy false discovery rate (FDR) estimation. Database searching was done by Sequest HT with a 10 ppm precursor ion tolerance and 0.02 Da fragment ion tolerance. Dynamic modifications were set including oxidation at methionine (+15.995 Da), **SiR-Tz1** at cysteine or lysine (+584.284 Da), **SiR-Tz17** at cysteine or lysine (+652.347 Da), carbamido­methylation at cycteine (+57.021 Da), acetylation at peptide N-termini (+42.011 Da), methionine loss at peptide N-termini (−131.040 Da), **SiR-Tz1** at peptide N-termini, **SiR-Tz17** at peptide N-termini, and acetylation at methionine loss (−89.030 Da). Maximum three missed cleavages were allowed. Peptide-spectrum matches (PSMs) were filtered by a percolator with 1% FDR. Then 1% FDR controls were applied to peptide level.

### *In Situ* Tz-TCO Click Chemistry-Mediated Fluorescent Protein Labeling in Live Cells

For BTK labeling, 1 × 10^6^ of HL60 and RPMI8226 cells (suspension cells) were seeded on a six-well plate. After 24 h, cells were treated with 10 μM IBR-TCO and incubated for 1 h at 37 °C. Cells were further treated with 10 μM **SiR-Tz**s and incubated for 30 min at 37 °C. For the excess **SiR-Tz** quenching experiments in Figure S25, the cells were further incubated with 10 μM TCO-OH (Sigma) for 30 min. Next, the cells were then centrifuged at 3000*g* for 5 min. The supernatant was discarded and cell pellets were washed with PBS (Welgene). RIPA buffer containing 50 mM Tris-HCl (pH 7.5), 150 mM NaCl, 1% NP-40, 0.5% sodium deoxycholate, and protease inhibitor cocktail (Thermo) was added to the cell pellets for lysis. Cells were incubated on ice for 15 min. The cell lysate was centrifuged at 4 °C, 20000 *g* for 5 min. Protein concentration of the cell lysate was measured using the BCA assay kit (Thermo) and adjusted to 1 mg/mL. Then, 5× SDS loading buffer (Biosesang) was added to the lysate, and samples were heated at 90 °C for 5 min. The protein samples were analyzed by 12.5% SDS-PAGE and scanned with Sapphire biomolecular imager (Azure Biosystems). The gels were subsequently stained with Coomassie Blue to confirm protein loading levels.

For XPO1 labeling, 1 × 10^6^ SH-SY5Y cells (adherent cells) were seeded on a six-well plate. After 24 h, cells were treated with 10 μM SEL-TCO and incubated 1 h at 37 °C. Cells were further treated with 10 μM **SiR-Tz**s and incubated for 30 min at 37 °C. Cells were then centrifuged at 3000*g* for 5 min. The supernatant was discarded and cell pellets were washed with PBS (Welgene). RIPA buffer containing 50 mM Tris-HCl (pH 7.5), 150 mM NaCl, 1% NP-40, 0.5% sodium deoxycholate, and protease inhibitor cocktail (Thermo) was added to the cell pellets for lysis. Cells were incubated on ice for 15 min. The cell lysate was centrifuged at 4 °C, 20000*g* for 5 min. Protein concentration of the cell lysate was measured using a BCA assay kit (Thermo) and adjusted to 1 mg/mL. Then, a 5× SDS loading buffer (Biosesang) was added to the lysate, and samples were heated at 90 °C for 5 min. The protein samples were analyzed by 12.5% SDS-PAGE and scanned with Sapphire biomolecular imager (Azure Biosystems). The gels were subsequently stained with Coomassie Blue to confirm protein loading levels.

### *In Situ* SiR-Tz20-TCO Click Chemistry-Mediated Fluorescent Proteome Reactivity in Various Lysis Buffer

A total of 1 × 10^7^ of HL60 (suspension cells) were seeded on a 100 ϕ dish. After 24 h, the cells were treated with 10 μM IBR-TCO and incubated 1 h at 37 °C. Next, the cells were treated with 10 μM **SiR-Tz20** and incubated for 30 min at 37 °C. The cells were then centrifuged at 3000*g* for 5 min, and the supernatant was discarded. The cell pellets were washed with PBS (Welgene) and divided into 12 tubes to access **SiR-Tz20** proteome reactivity in 12 different lysis buffers. The 3 × 4 combinations of buffers included (1) Tris-based buffer (50 mM Tris and 150 mM NaCl), (2) Hepes-based buffer (50 mM Hepes, 150 mM NaCl, and 10 mM EDTA), and (3) PBS-based buffer (Welgene). Each buffer was supplemented with one of the following: (i) NP+DOC (1% NP-40, 0.5% sodium deoxycholate), (ii) NP (1% NP-40), (iii) X-100 (1% Triton X-100), or (iv) urea (8 M urea). A protease inhibitor cocktail (Thermo) was added to each lysis buffer. The cell pellets were then lysed on ice for 15 min. The resulting cell lysates were centrifuged at 4 °C at 20000*g* for 5 min. The protein concentration was measured using a BCA assay kit (Thermo) and adjusted to 1 mg/mL. Next, 5× SDS loading buffer (Biosesang) was added, and the samples were heated at 90 °C for 5 min. The protein samples were analyzed by 12.5% SDS-PAGE and scanned using a Sapphire biomolecular imager (Azure Biosystems). The gels were subsequently stained with Coomassie Blue to confirm protein loading levels.

### Cell Priming with PMA

PMA (Sigma-Aldrich) was used to induce the adherent status of THP-1 cells for fluorescent imaging. PMA was diluted to 0.1 mM in culture conditions of RPMI media. PMA-containing media was mixed with the cell suspension at a ratio of 1:1. THP-1 cells were plated on a poly d-lysine (PDL)-coated confocal dish and incubated for 24 h before compound treatment.

### Immunofluorescence

For BTK monitoring, PMA-primed THP-1 cells were treated with 10 μM IBR-TCO and incubated for 20 min at 37 °C. After that, 10 μM **SiR-Tz**s was added to the cells and incubated for 20 min at 37 °C. In each step, the cell culture media or buffer was gently aspirated, and cells were washed with PBS 3 times. Cells were fixed with 4% paraformaldehyde for 15 min and permeabilizated for 10 min with 0.1% Triton X-100 diluted in PBS. Cells were blocked with 1% BSA in PBST for 1 h. Cells were then incubated with primary antibody solution for 1 h at room temperature. After that, cells were incubated with fluorescence conjugated secondary antibody solution for 30 min at room temperature in the dark. All the antibodies were diluted in TBST as follows: anti-BTK (Cell Signaling/no. 8547, 1:300) and anti-Alexa 488 (abcam/ab150077, 1:600). In each step, buffer solutions were aspirated and washed once with PBS. The cells were observed by LSM-800 (Zeiss). Images were captured using the rhodamine channel (ex/em = 652 nm/668 nm) for **SiR-Tz**s and the Alexa 488 channel (ex/em = 499 nm/520 nm) for Alexa-488 secondary antibody.

For XPO1 monitoring, SH-SY5Y cells were seeded in a confocal dish and incubated overnight. Cells were treated with 10 μM SEL-TCO and incubated for 1 h at 37 °C, followed by treatment with 10 μM **SiR-Tz1** or **SiR-Tz20** for 30 min at 37 °C. Cells were then fixed with 4% paraformaldehyde (PFA) for 20 min, permeabilized with 0.1% Triton X-100 in PBS for 15 min, and blocked with 4% BSA in PBS for 1 h. For immunostaining, cells were incubated with XPO1/CRM1 primary antibody (1:400, CST, 46249S) for 1 h at room temperature (RT), followed by Alexa 488-conjugated secondary antibody (1:800, Invitrogen, A21206) for 1 h at RT. Nuclei were stained with Hoechst 33342 (1:5000 in PBS) for 20 min. Between each step, cells were washed with PBS 3 times. Imaging was performed using a STELLARIS 5 confocal microscope (Leica). Fluorescence signals were captured using the Alexa 405 laser (ex/em = 405 nm/430 nm) for Hoechst, Alexa 488 laser (ex/em = 499 nm/520 nm) for XPO1, and the Alexa 647 laser (ex/em = 653 nm/680 nm) for **SiR-Tz**.

### Mitocondrial Colocalization Imaging

For mitochondria monitoring, HeLa cells were seeded on a confocal dish and transfected with pCAG-mitoYFP (Plasmid #168508) using P3000 according to the manufacturer’s protocol. After 18 h, cells were treated with 10 μM TPP-TCO and incubated for 20 min at 37 °C. Subsequently, 10 μM **SiR-Tz1** or **SiR-Tz20** was added, followed by 20 min of incubation at 37 °C. Cells were then fixed with cold 4% PFA for 5 min at room temperature and blocked with 1% BSA diluted in PBS for 30 min. Nuclei were counterstained with Hoechst 33342 (1:3000 in PBS) for 5 min. Between each step, cells were washed three times with PBS. Imaging was performed using an LSM-800 confocal microscope (Zeiss). Fluorescence signals were captured using the DAPI channel (ex/em = 353 nm/465 nm) for Hoechst, the YFP channel (ex/em = 513 nm/527 nm) for mitochondria, and the SiR channel (ex/em = 645 nm/700 nm) for **SiR-Tz**.

### Flow Cytometry

For BTK monitoring, 1 × 10^6^ THP-1 cells and Jurkat cells were added per tube. Cells were treated with 1 μM IBR-TCO and incubated for 20 min at 37 °C. **SiR-Tz**s (10 nM) were then treated and incubated for 20 min at 37 °C. In each step, the cell culture media or buffer was aspirated and washed with PBS 3 times. The mouse study was approved by the Institutional Animal Care and Use Committee 2946 (IACUC) of the Korea Institute of Science and Technology (KIST-2947 5088-2022-05-073). Eight-week-old BALB/c mice were purchased from DBL (Korea). Balb/c mice were sacrificed, and their spleen tissues were collected. The spleen was digested in RBC lysis buffer for single-cell suspension. The dissociated cells were then filtered through a 70 μm nylon cell strainer and collected for the following analyses. Dissociated splenocytes were treated with 10 μM IBR-TCO and incubated for 20 min in 37 °C. **SiR-Tz**s (0.1 μM) were treated and incubated for 20 min at 37 °C. In each step, the buffer solutions were aspirated and washed with PBS 3 times. After that, cells were resuspended and incubated with primary antibody solution for 1 h on ice in the dark. Cells were then incubated with fluorescence conjugated secondary antibody solution for 30 min on ice in the dark. All the antibodies were diluted in 0.5% BSA in PBS as follows: anti-CD19 (Cell Signaling/no. 8547, 1:300), anti-Alexa 488 (abcam/ab150077, 1:600). The 4-channel CytoFLEX LX (Beckman Coulter, Brea, USA) was used to analyze the samples. A minimum of 10,000 events were acquired from each sample. The data were analyzed using FlowJo 10.1 (LLC, Ashland, OR, USA).

## Supplementary Material



## Notes

Supplementary figures, supplementary table, chemical synthetic procedures, and compound characterization including ^1^H and ^13^C NMR spectra (PDF)
