# Supplementary material for: Investigation of Proteome-Tetrazine Reactivity for a Highly Selective Tetrazine Ligation in Live Cells
Source: ACS Cent Sci. 2025 May 6;11(6):878–89. doi: 10.1021/acscentsci.5c00525 (PMC12203430; doi:10.1021/acscentsci.5c00525)
Supplement: Supplementary file 1 [file oc5c00525_si_001.pdf]

Supplementary Information

Investigation of proteome-tetrazine reactivity for a highly selective tetrazine ligation in live cells.

Junyoung Park[a,†], Juhee Hahm[a,†], Junhyeong Yim[a,b,†], Hyelim Lee[c], Hwan Min Hwang[a], Soyeon Lee[a], Ju-Young Park[d], Velladurai Arun[e], Gangasani Jagadeesh Kumar[a,f], Hana Cho[g,h], Hankum Park[g,h,i], Minju Lee[e], Jeehee Lee[c,j], Hyunuk Eom[k], Woon Ju Song[k], Sanghee Lee[c,l], Eunha Kim[e,m,n,o]* and Jongmin Park[a,b,f]**

[a]Department of Chemistry, Kangwon National University, Chuncheon 24341, Korea

[b]Multidimensional Genomics Research Center, Kangwon National University, Chuncheon 24341, Republic of Korea

[c]Medicinal Materials Research Center, Biomedical Research Division, Korea Institute of Science and Technology (KIST), Seoul 02792, Republic of Korea

[d]Molecular Science and Technology Research Center, Ajou University, Suwon 16499, the Republic of Korea

[e]Department of Molecular Science and Technology, Ajou University, Suwon 16499, Korea

[f]Institute for Molecular Science and Fusion Technology, Kangwon National University, Chuncheon 24341, Republic of Korea

[g]Department of Dental Sciences, School of Dentistry, Seoul National University, Seoul 08826, Korea

[h]Institute for Data Innovation in Science, Seoul National University, Seoul 08826, Republic of Korea

[i]Dental Multiomics Center, School of Dentistry and Dental Research Institute, Seoul National University, Seoul 08826, Republic of Korea

[j]Department of HY-KIST Bio-convergence, Hanyang University, Seoul 04763, Republic of Korea

[k]Department of Chemistry, Seoul National University, 1 Gwanak-ro, Gwanak-gu, Seoul 08826, Republic of Korea

[l]KHU-KIST Department of Converging Science and Technology, Kyung Hee University, Seoul, 02453, Republic of Korea

[m]Advanced college of Bio-convergence Engineering, Ajou University, Suwon 16499, Republic of Korea

[n]Department of Bio-convergence Engineering, Ajou University, Suwon 16499, Republic of Korea

[o]Department of Otolaryngology, Ajou University School of Medicine, Suwon 16499, Republic of Korea

[†]These authors are equally distributed

* Correspondence to Jongmin Park (jpark@kangwon.ac.kr), Eunha kim (ehkim01@ajou.ac.kr), Sanghee Lee (slee19@kist.re.kr)

Contents

Supplementary Figures -- S1

General Information for Chemical Synthesis -- S25

Synthesis and Characterization of Compounds -------------------------------------- S31

^{1}H and ^{13}C NMR Spectra --- S66

References --- S124

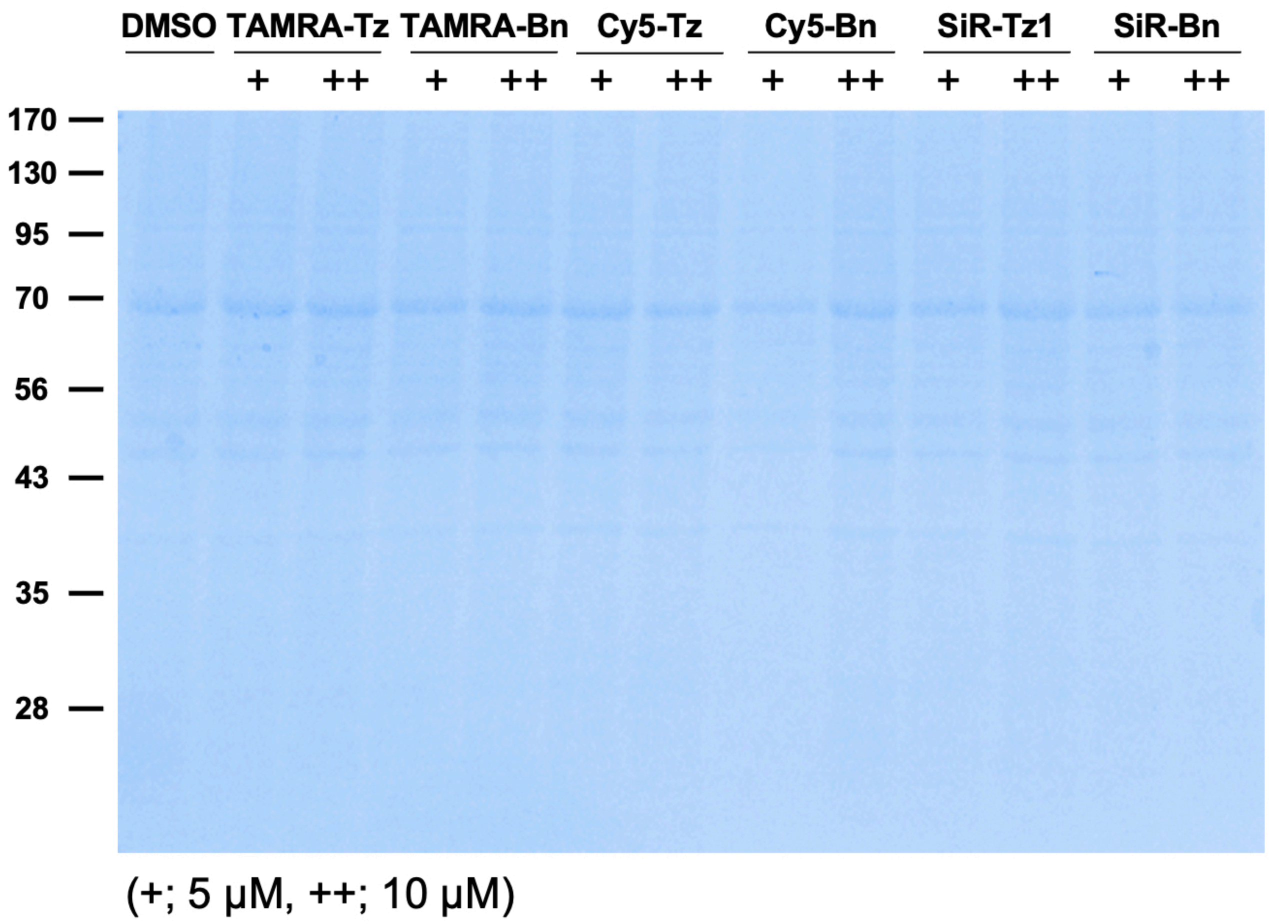

Figure S1. Coomassie Blue staining of Figure 1B.

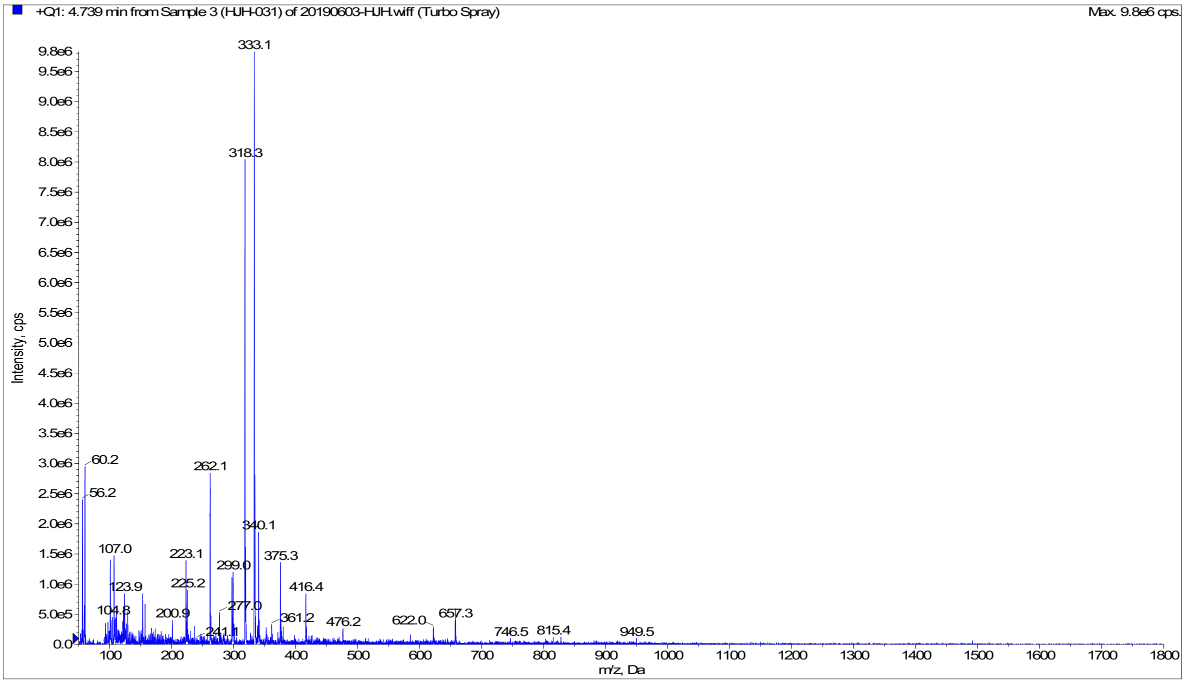

Figure S2. LRMS-ESI spectra of **MFHA**. $[M+H]^+$ calcd for $C_{18}H_{29}N_4O_2^+$, 333.2; found 333.1

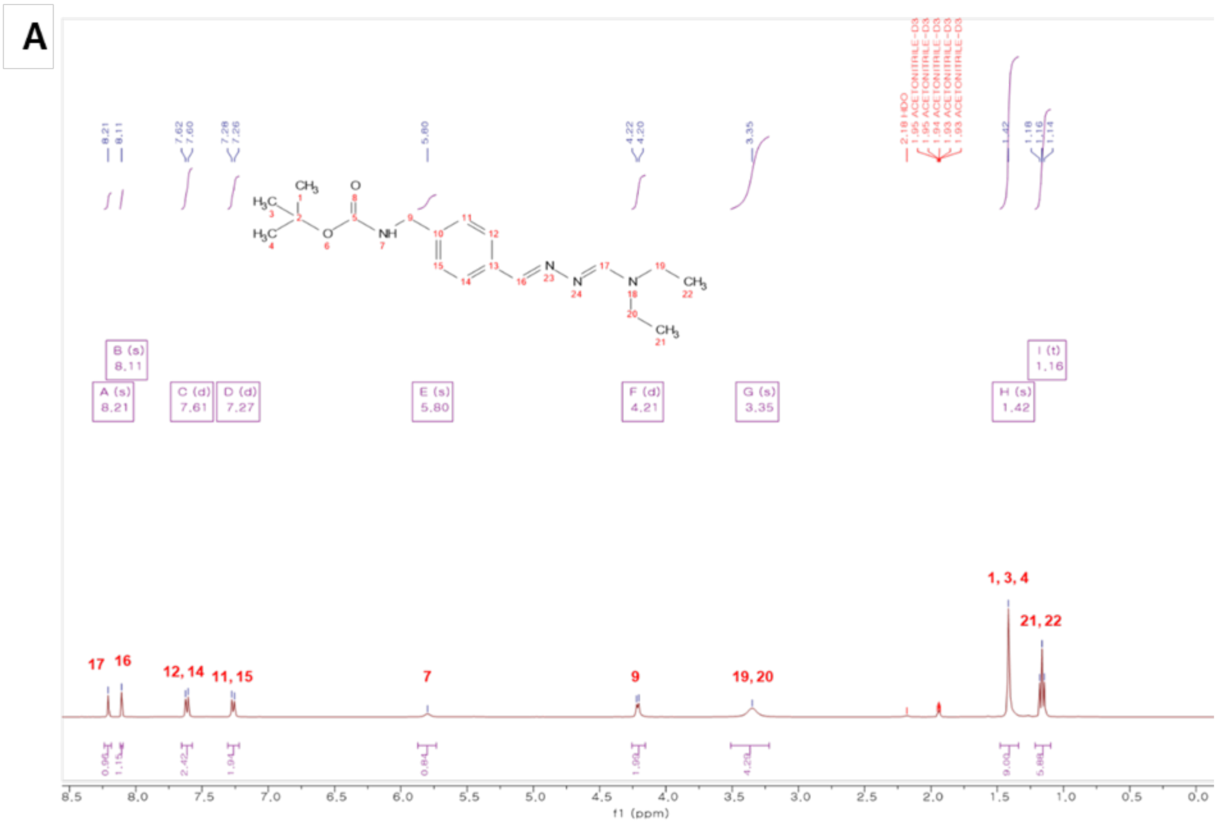

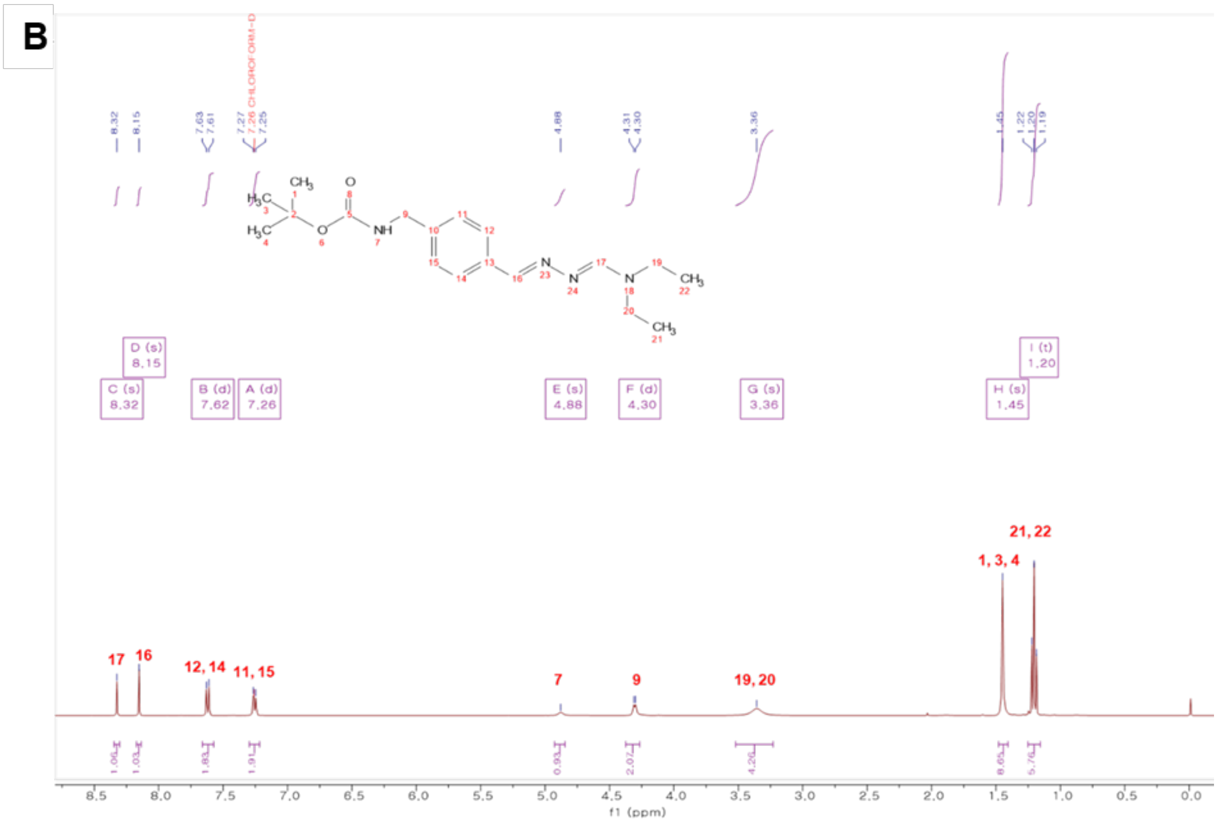

Figure S3. ^{1}H-NMR (400 MHz) spectra of **MFHA**. (A) ^{1}H-NMR with Acetonitrile-*d*3. (B) ^{1}H-NMR with Chloroform-*d*.

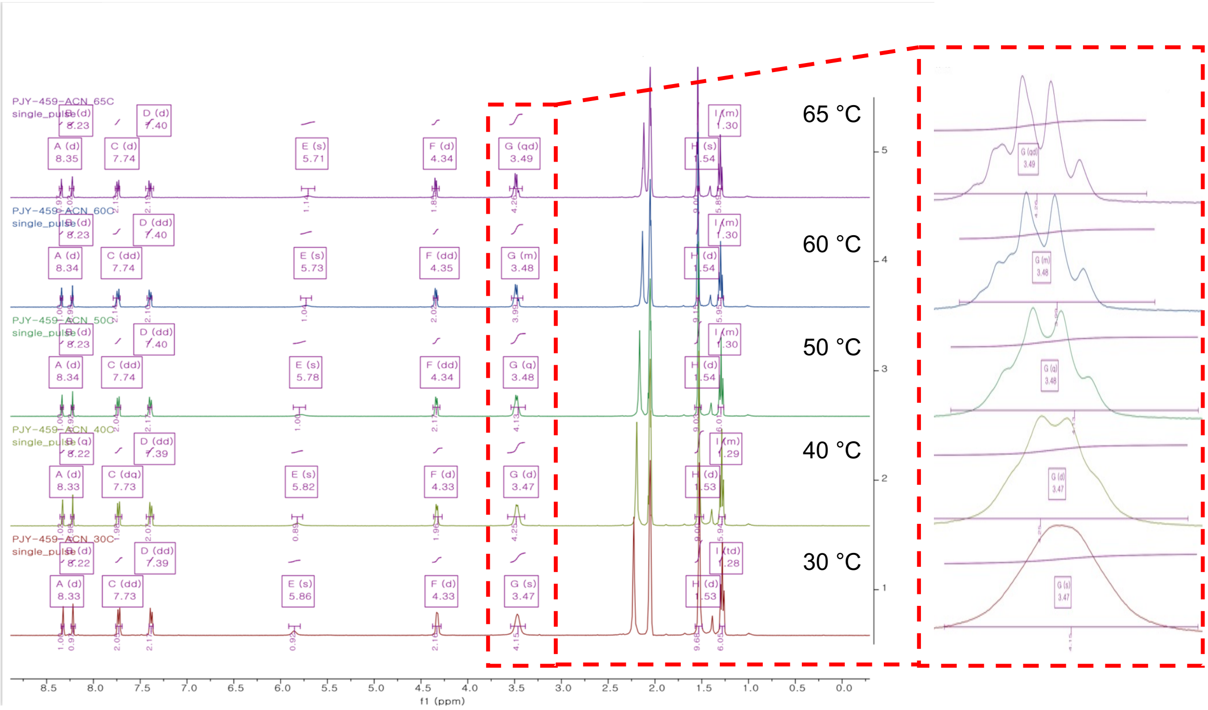

Figure S4. ^{1}H-NMR (400 MHz, Acetonitrile-*d*3) data showing temperature-dependent peak splitting of **MFHA**, identified as a rotamer. The NMR data were obtained at 30°C, 40°C, 50°C, 60°C, and 65°C.

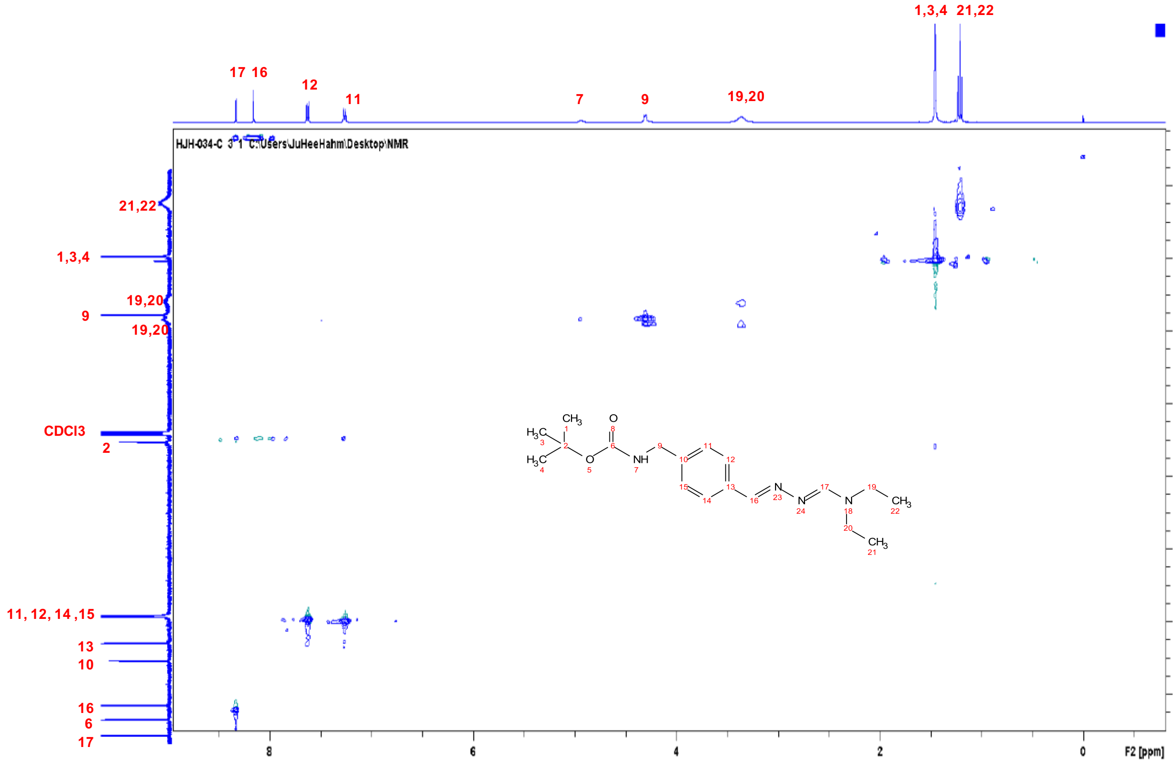

Figure S5. ^{1}H-^{13}C HSQC NMR data of **MFHA** with Chloroform-*d*.

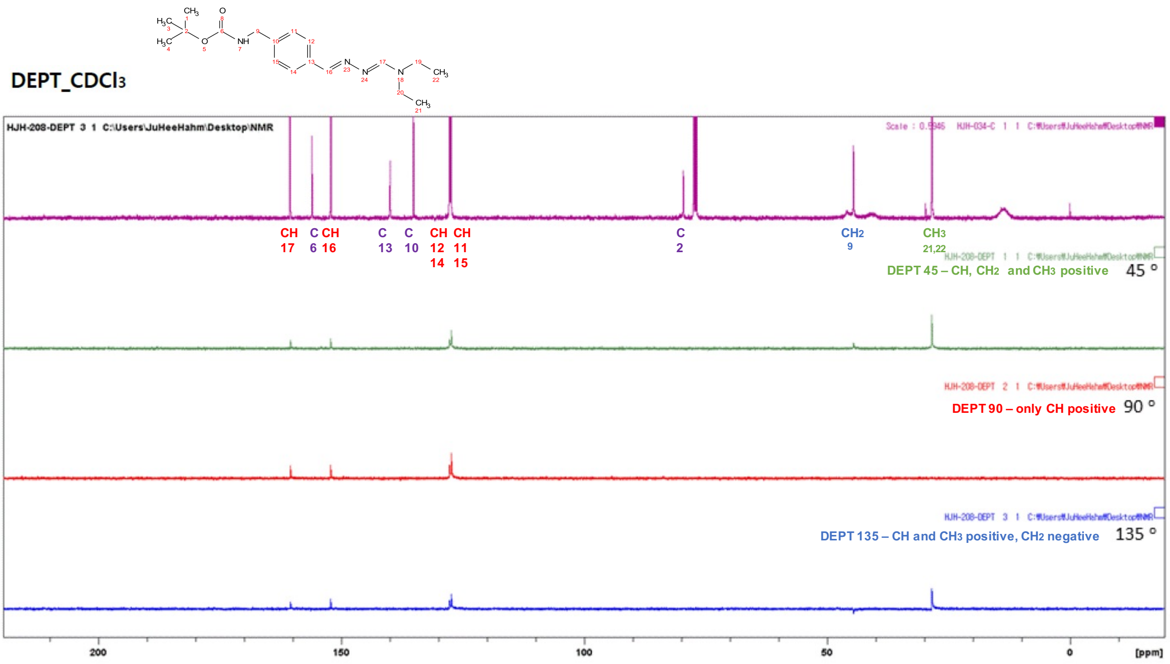

Figure S6. DEPT NMR data of **MFHA** showing the differentiation of CH, CH_2, and CH_3 groups.

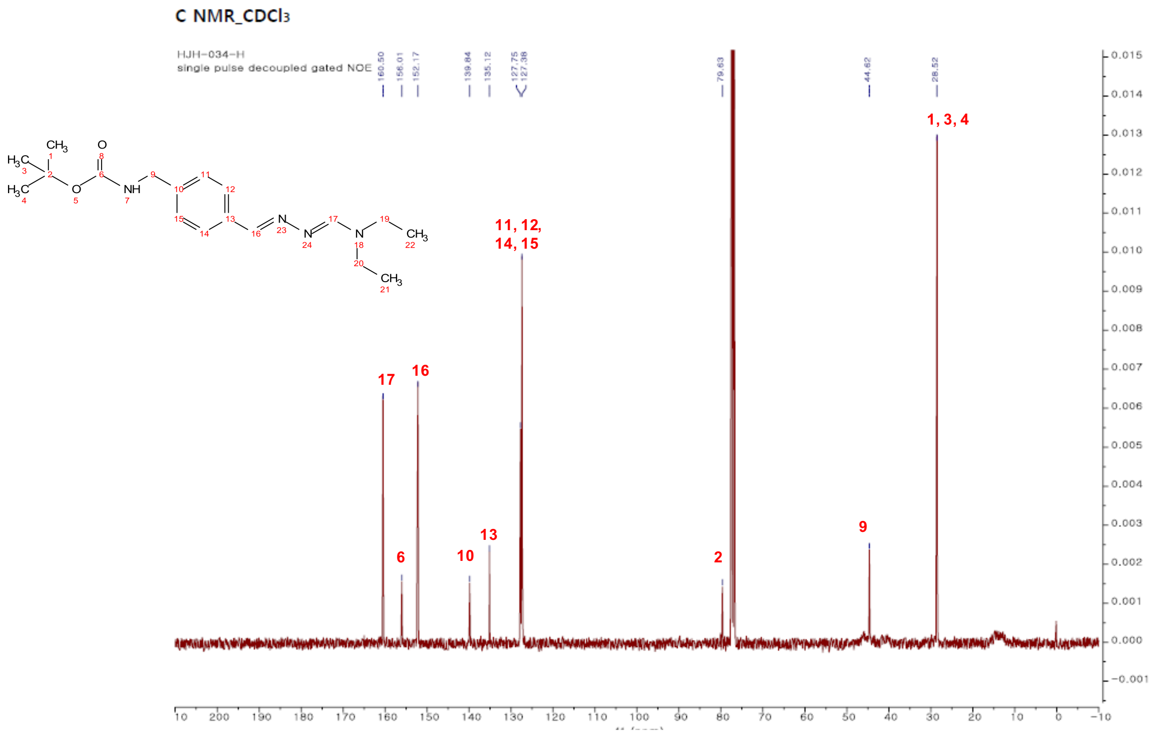

Figure S7. ^{13}C-NMR (101 MHz, Chloroform-*d*) data of **MFHA**.

A

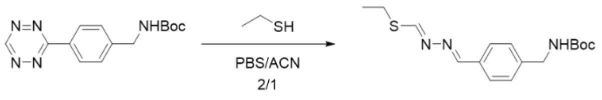

B

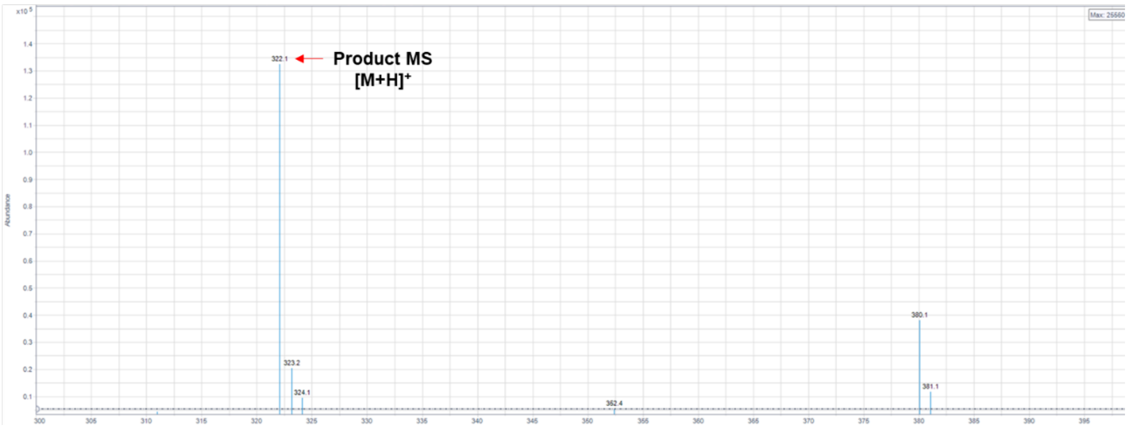

Figure S8. Reaction of **Boc-Tz1** (1 eq) with thioethanol (5 eq) in PBS/ACN (2/1). (A) Reaction scheme of **Boc-Tz1** with thioethanol, which generate N-methylenemethanehydrazonothioate (**MMHT**). (B) Mass spectrum of **MMHT**, showing the expected molecular ion peaks. ESI-MS m/z: calc. for $C_{16}H_{24}N_3O_2S^+$, $[M+H]^+$: 322.16, found 322.1

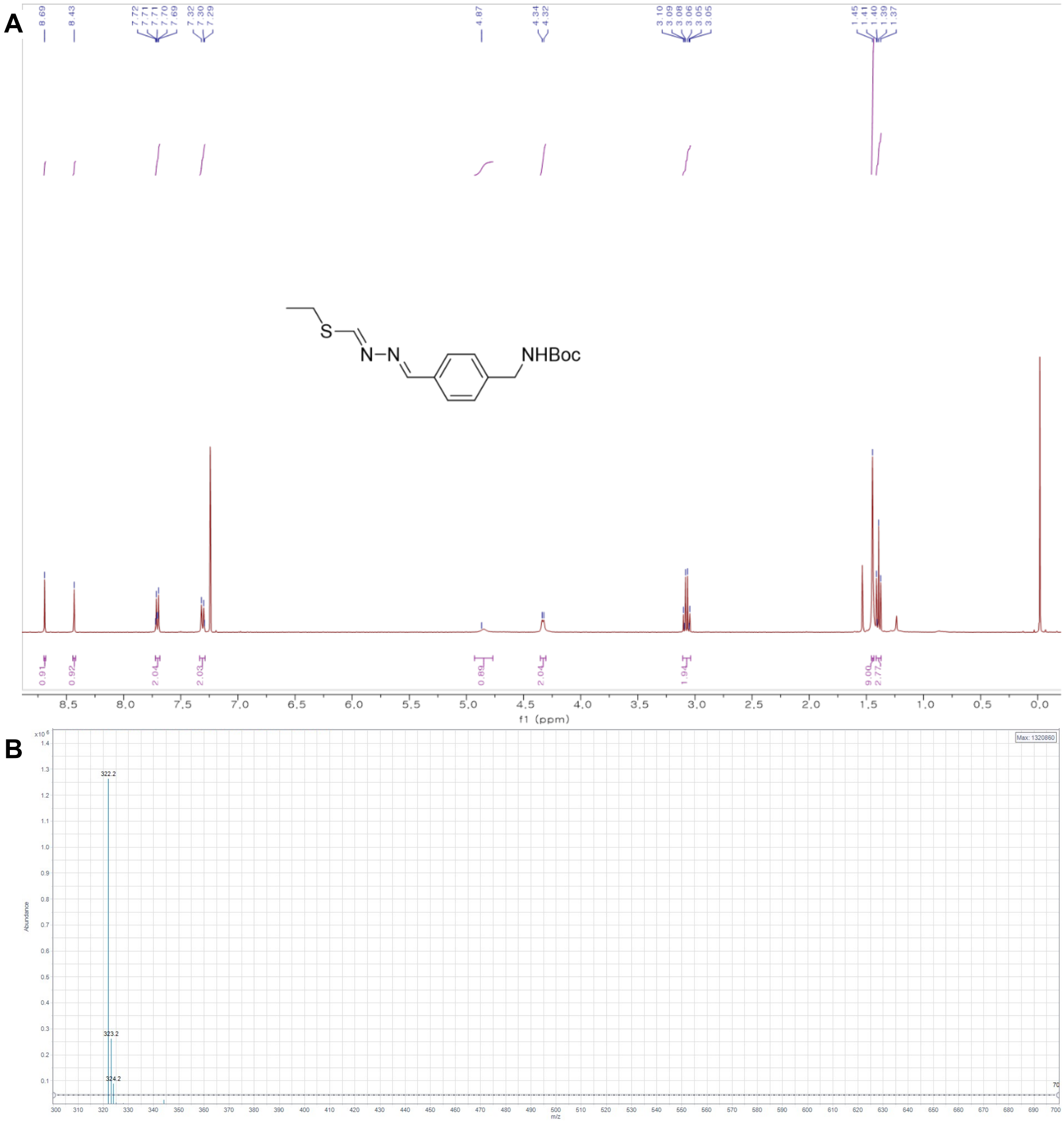

Figure S9. ^{1}H-NMR and mass spectrometry analysis of N-methylenemethanehydrazonothioate (**MMHT**). (A) ^{1}H-NMR spectrum of **MMHT** in ($CDCl_3$, 400 MHz). (B) LRMS-ESI spectra of **MMHT**. ESI-MS m/z: calc. for $C_{16}H_{24}N_3O_2S^+$, $[M+H]^+$: 322.16, found 322.1

A

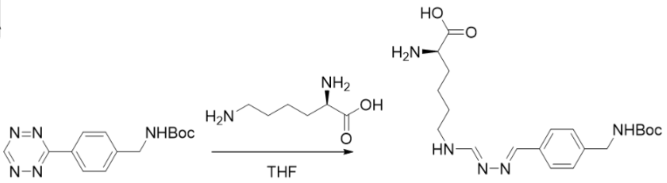

B

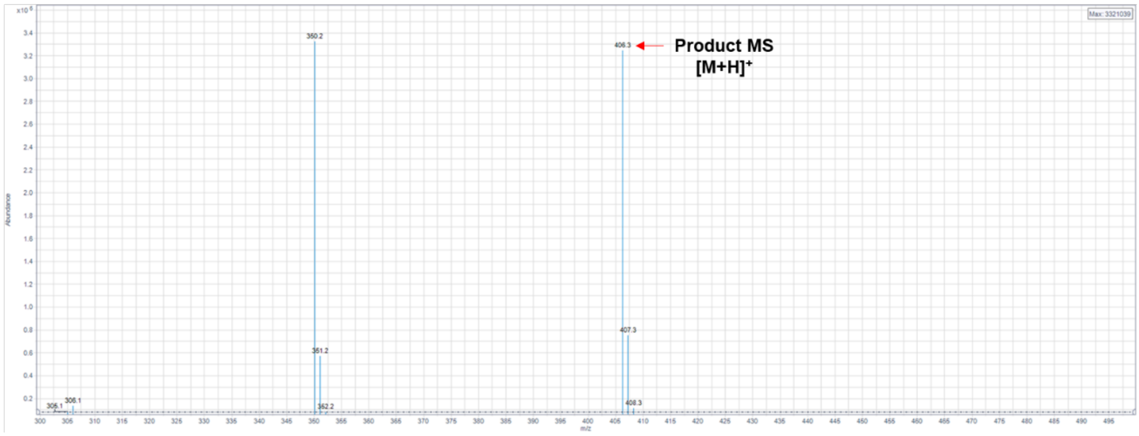

Figure S10. Reaction of **Boc-Tz1** (1 eq) with Lys (5 eq) in THF. (A) Reaction scheme of **Boc-Tz1** with Lys. (B) Mass spectrum of the product, showing the expected molecular ion peaks. ESI-MS m/z: calc. for $C_{20}H_{32}N_5O_4^+$, $[M+H]^+$: 406.24, found 406.3

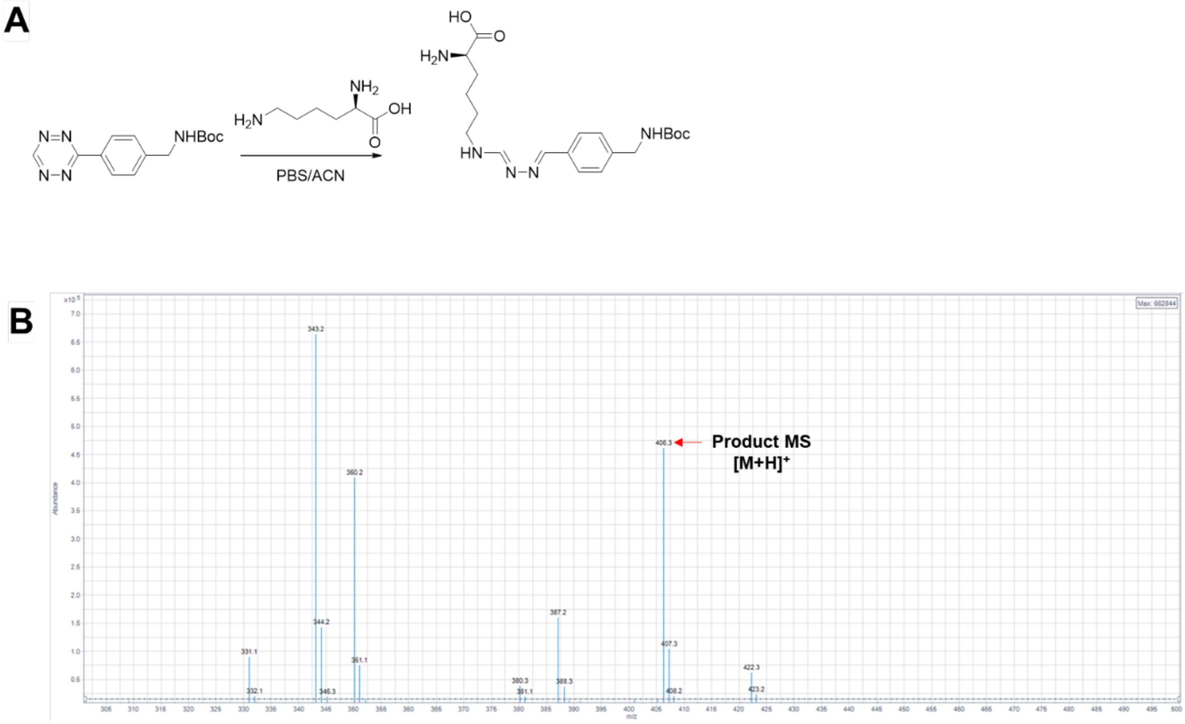

Figure S11. Reaction of **Boc-Tz1** (1 eq) with Lys (5 eq) in PBS/ACN (2/1). (A) Reaction scheme of **Boc-Tz1** with Lys. (B) Mass spectrum of the product, showing the expected molecular ion peaks. ESI-MS m/z: calc. for $C_{20}H_{32}N_5O_4^+$, $[M+H]^+$: 406.24, found 406.3

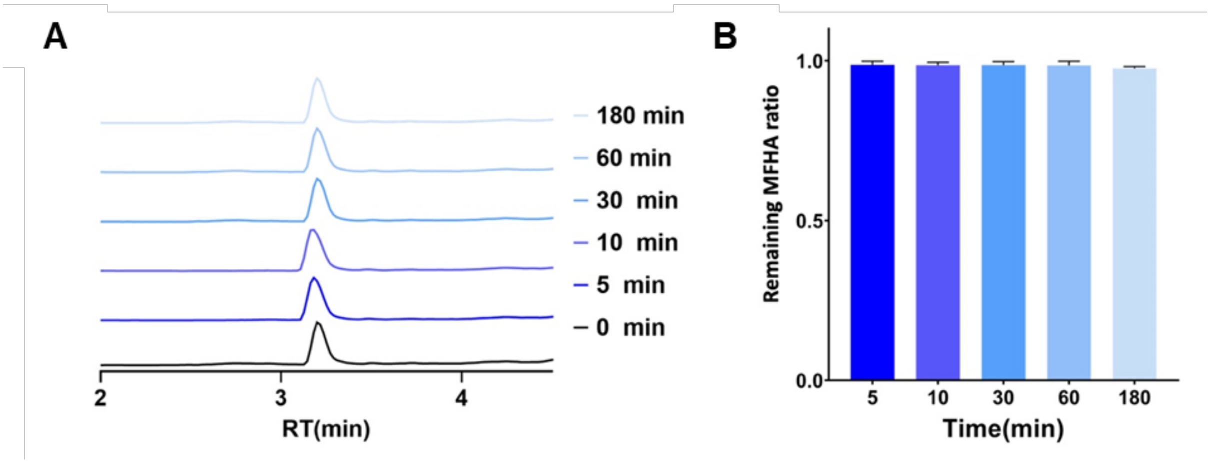

Figure S12. Stability of **MFHA** in PBS. **MFHA** in PBS was analyzed by LC-MS in a time-dependent manner. (A) LC-MS analysis showed that **MFHA** is stable in PBS. (B) The area under curve of (A) was quantified.

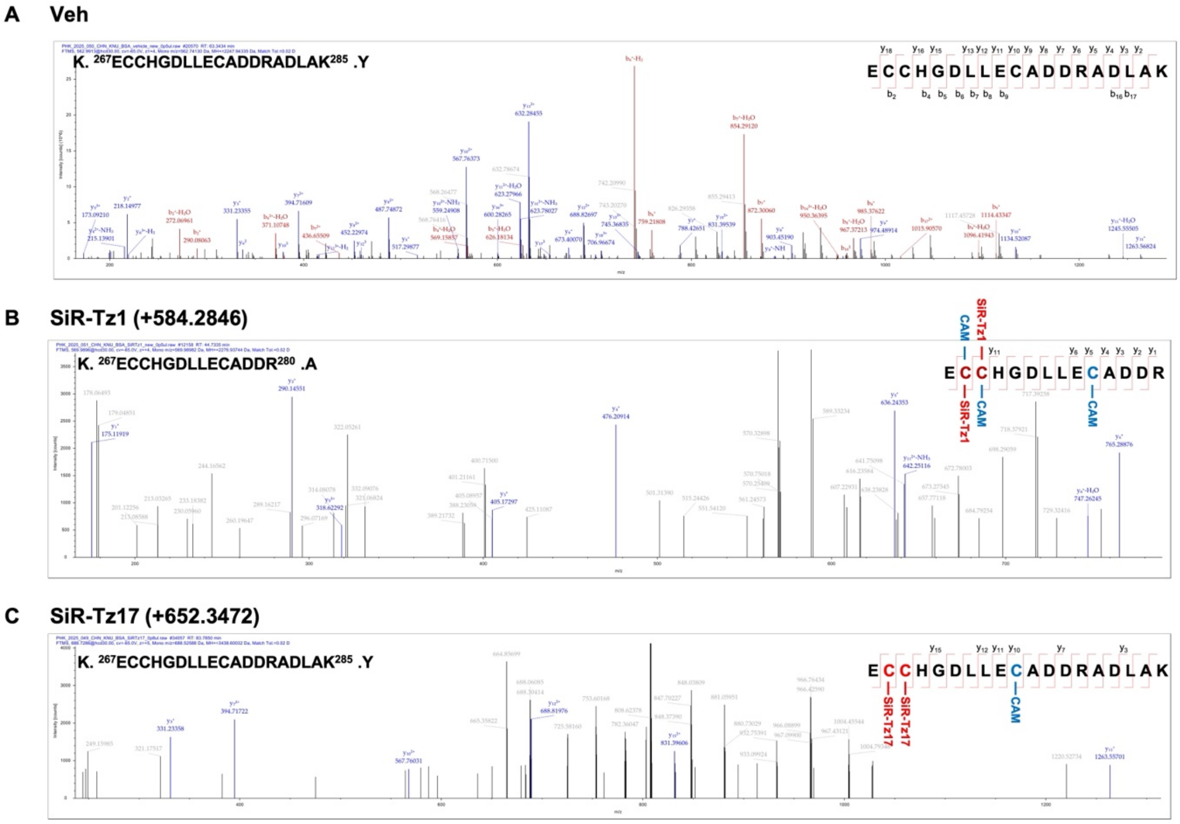

Figure S13. Mass spectrometry analysis for Proteome labeling by (B) **SiR-Tz1** and (C) **SiR-Tz17**. Bovine serum albumin (BSA) was incubated with 1 mM **SiR-Tz**s for 30 min, followed by tryptic digestion. A tryptic peptide of BSA that contains Cys268 and Cys269 (amino acids 267–280 and 267–285) exhibited distinct proteome labeling by **SiR-Tzs**.

Tetrazine		TCO		Solvent	Temp.	Detection wavelength (nm)	k_2 ($M^{-1}s^{-1}$)
Compound	Conc.#	Compound	Conc.#				
**Boc-Tz1**	200 µM	5OH-TCO	2.0 mM	PBS/DMSO (1:1)	37 ºC	530 nm	98750.0
**Boc-Tz2**							3169.0
**Boc-Tz3**							168.9
**Boc-Tz4**							58490.0
**Boc-Tz5**							13360.0
**Boc-Tz6**							791.8
**Boc-Tz7**							3515.0
**Boc-Tz8**							14040.0
**Boc-Tz9**							84.1
**Boc-Tz10**							1357.0
**Boc-Tz11**							597.3
**Boc-Tz12**							9.5
**Boc-Tz13**							N/A
**Boc-Tz14**							1345.0
**Boc-Tz15**							1533.0
**Boc-Tz16**							1619.0
**Boc-Tz17**							1973.0
**Boc-Tz18**							N/A
**Boc-Tz19**							N/A
**Boc-Tz20**							1198.0
**Boc-Tz21**							76020.0
**Boc-Tz22**							1.7
**Boc-Tz23**							801.1

Table S1. Second-order rate constants (k_2) of **Tz**-TCO reactions determined by stopped-flow spectrophotometry. #Concentrations of the **Tz** and TCO solutions, which are mixed 1:1 in the analysis. The final concentration of **Tz** and TCO during the reaction is 100 µM and 1 mM, respectively (N/A : Not Available due to the solubility of the compound).

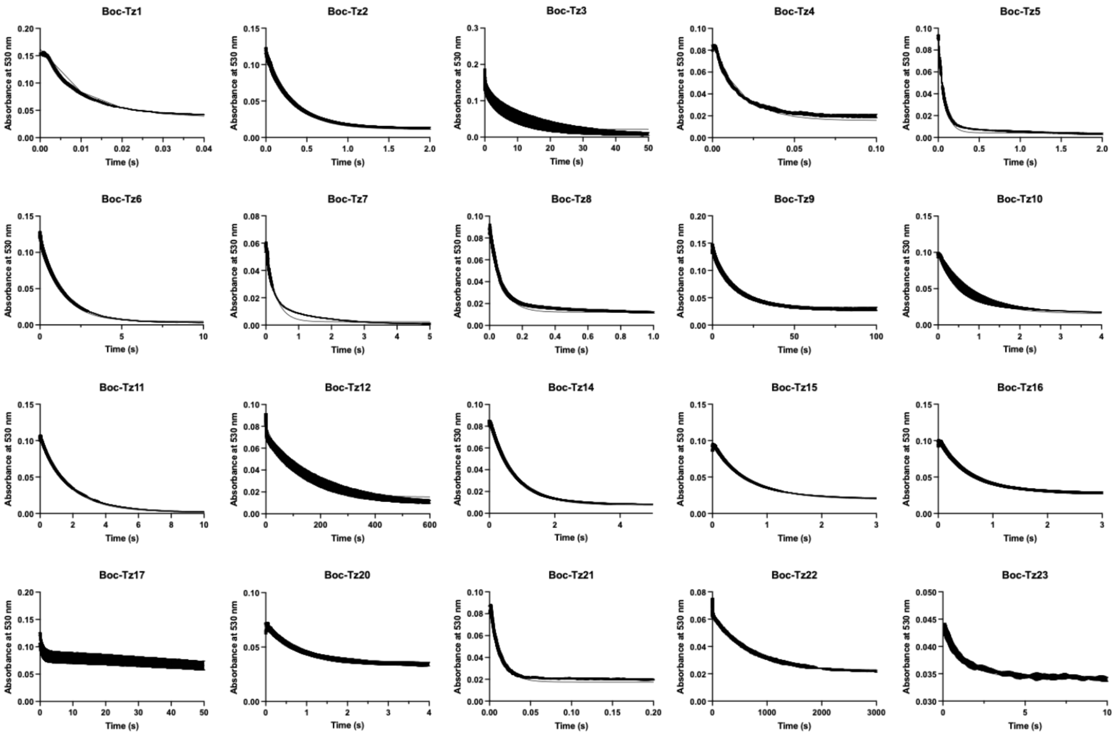

Figure S14. Kinetic plots of reaction of **SiR-Tz** derivatives with 5-OH-TCO in PBS/DMSO (1:1) solution at 37 °C.

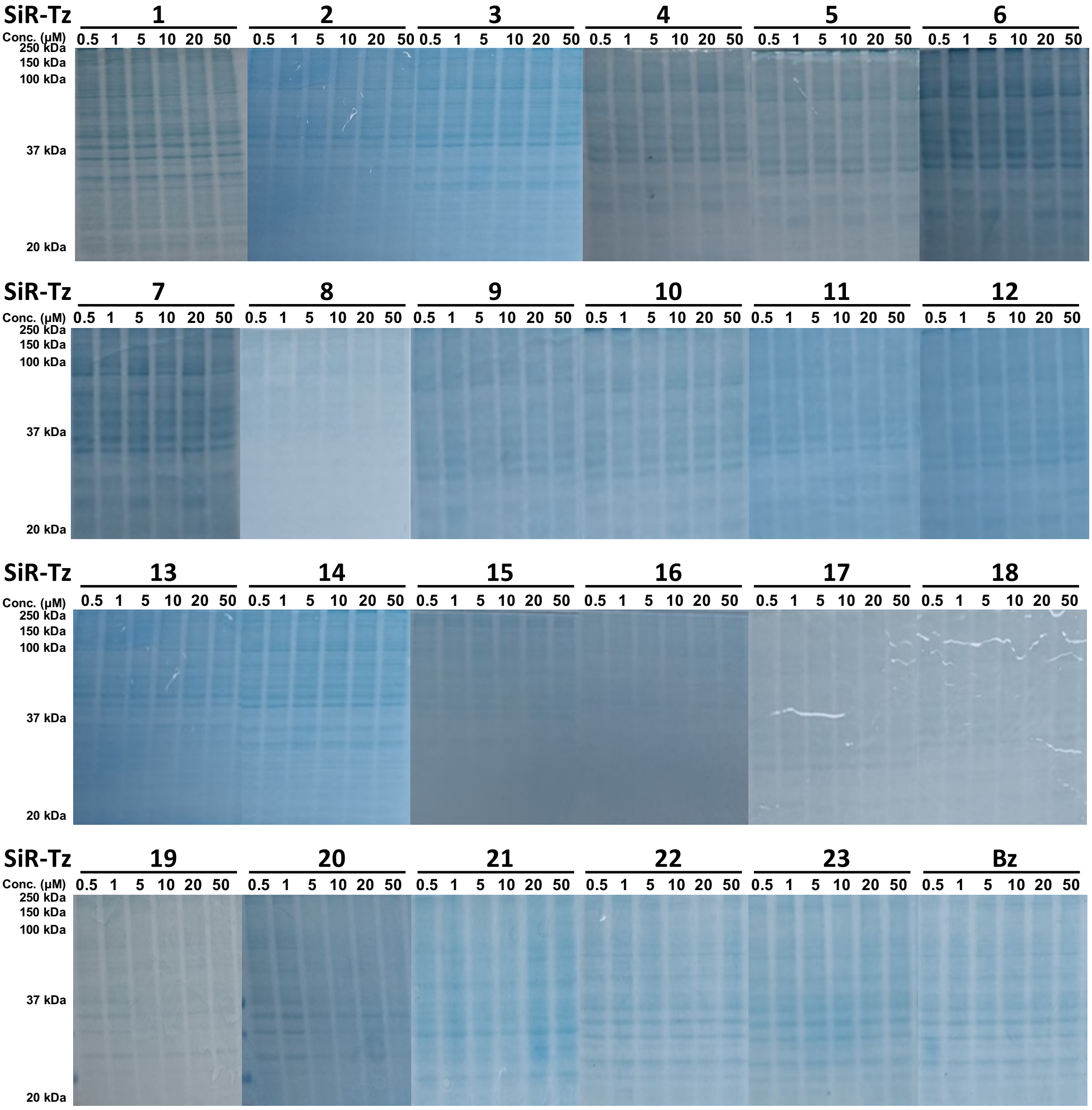

Figure S15. Coomassie Blue staining of Figure 2B.

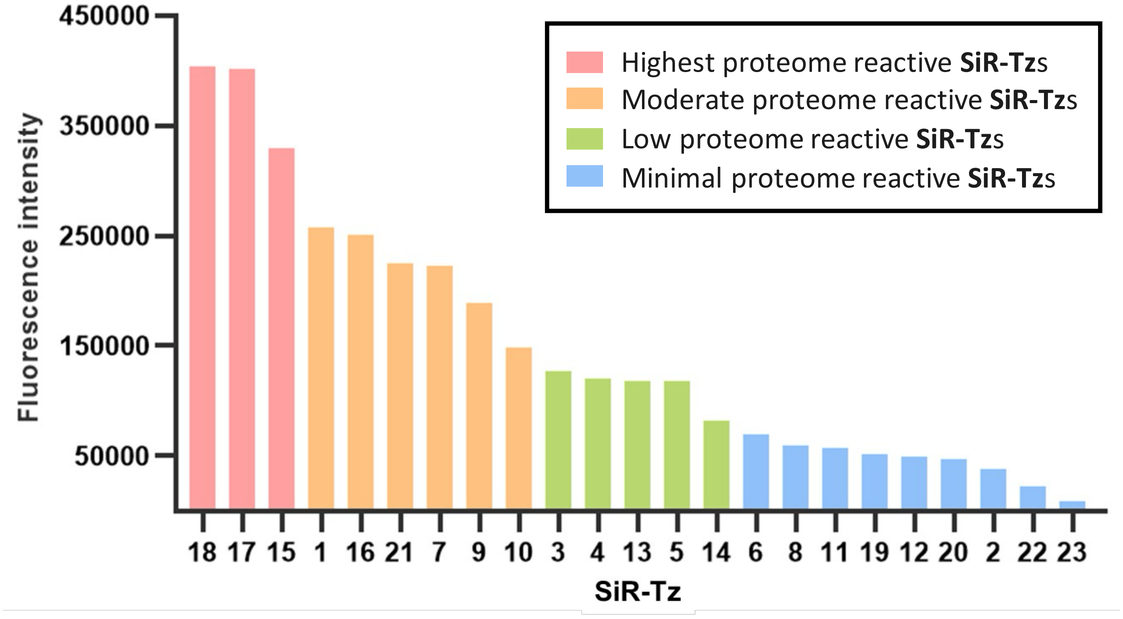

Figure S16. Fluorescent protein band quantification data of Figure 2B. Fluorescent intensities in the lane of the 20 µM **SiR-Tz**s treated proteome were analyzed. The fluorescent intensities of protein bands between 20 kDa and 150 kDa were quantified using *ImageJ* software.

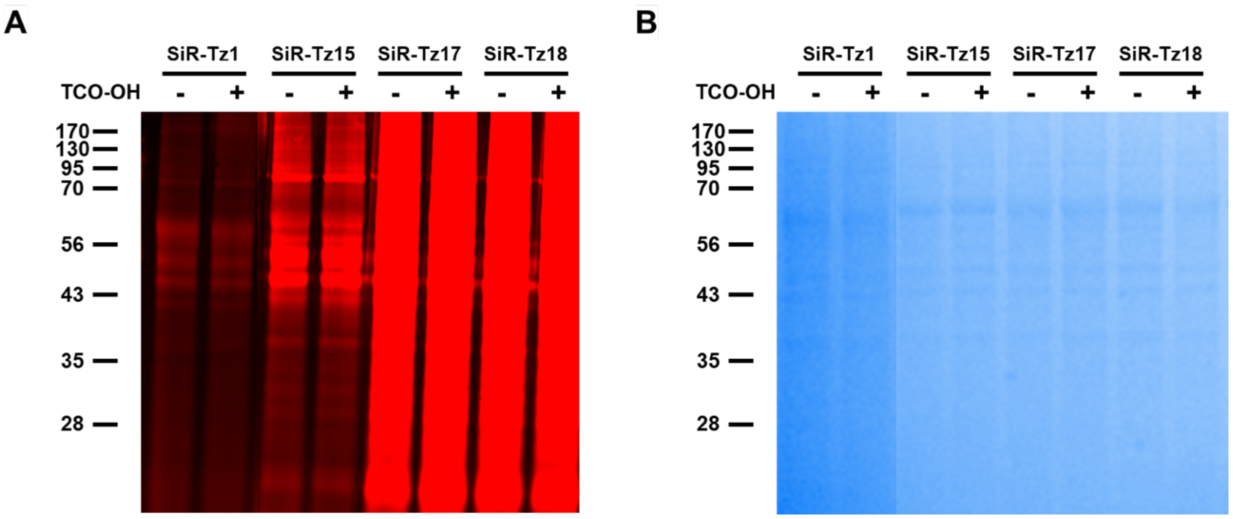

Figure S17. Proteome labeling by **SiR-Tz**s after quenching excess **SiR-Tz**s with TCO-OH in cell lysates. (A) HeLa cell lysates were incubated with 10 µM of **SiR-Tz1**, **SiR-Tz15**, **SiR-Tz17**, and **SiR-Tz18** for 30 min. The lysates were then incubated with 50 µM of TCO-OH for 10 min to quench excess **SiR-Tz**s. After SDS-PAGE, proteome reactivity was analyzed by fluorescent gel scanning, revealing there is no background reaction of excess **SiR-Tz**s due to heating step of SDS-PAGE. (B) Coomassie Blue staining corresponding to (A).

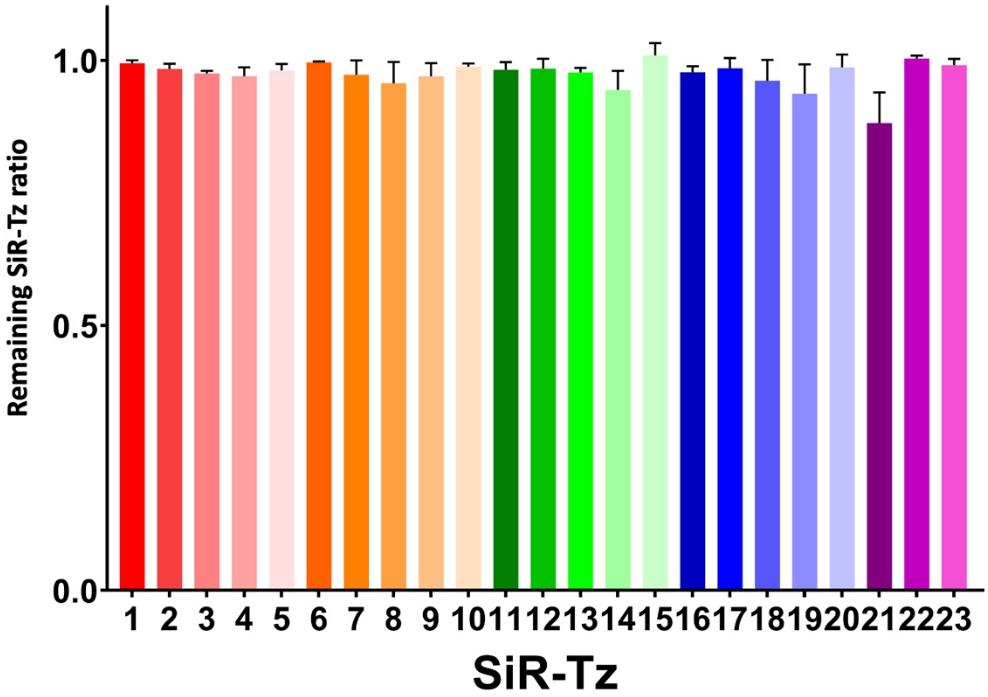

Figure S18. Stability of **SiR-Tz**s in PBS. Residual **SiR-Tz**s were analyzed by LC-MS after 30 min incubation in PBS.

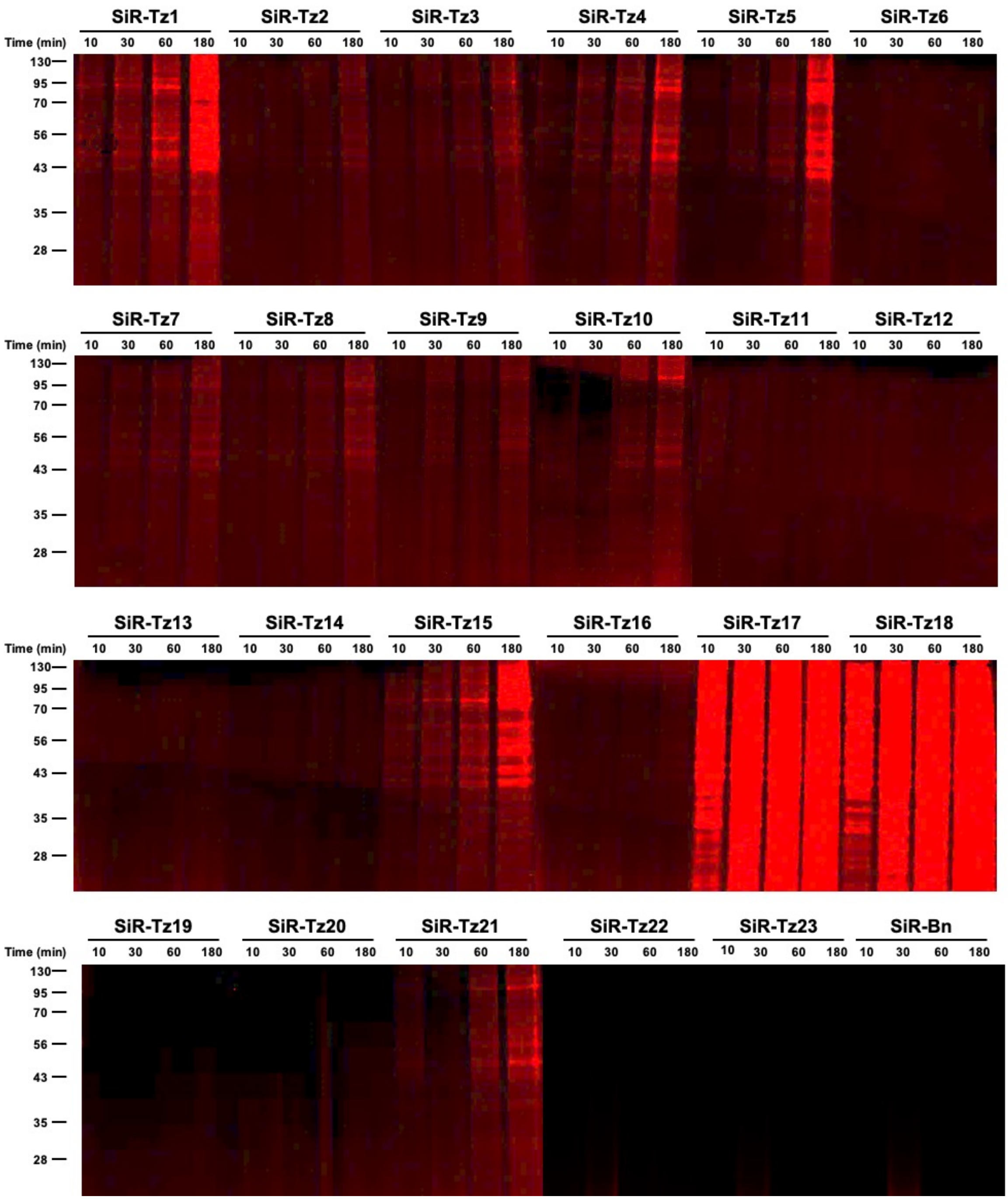

Figure S19. Proteome reactivity of tetrazine derivatives in a time-dependent manner. Proteome reactivity of **SiR-Tz**s and **SiR-Bn**. HeLa cell lysate were incubated with 10 μM **SiR-Tz**s and **SiR-Bn** for 10, 30, 60, 180 min. After SDS-PAGE, proteome reactivities of **SiR-Tz**s were analyzed by fluorescent gel scanning.

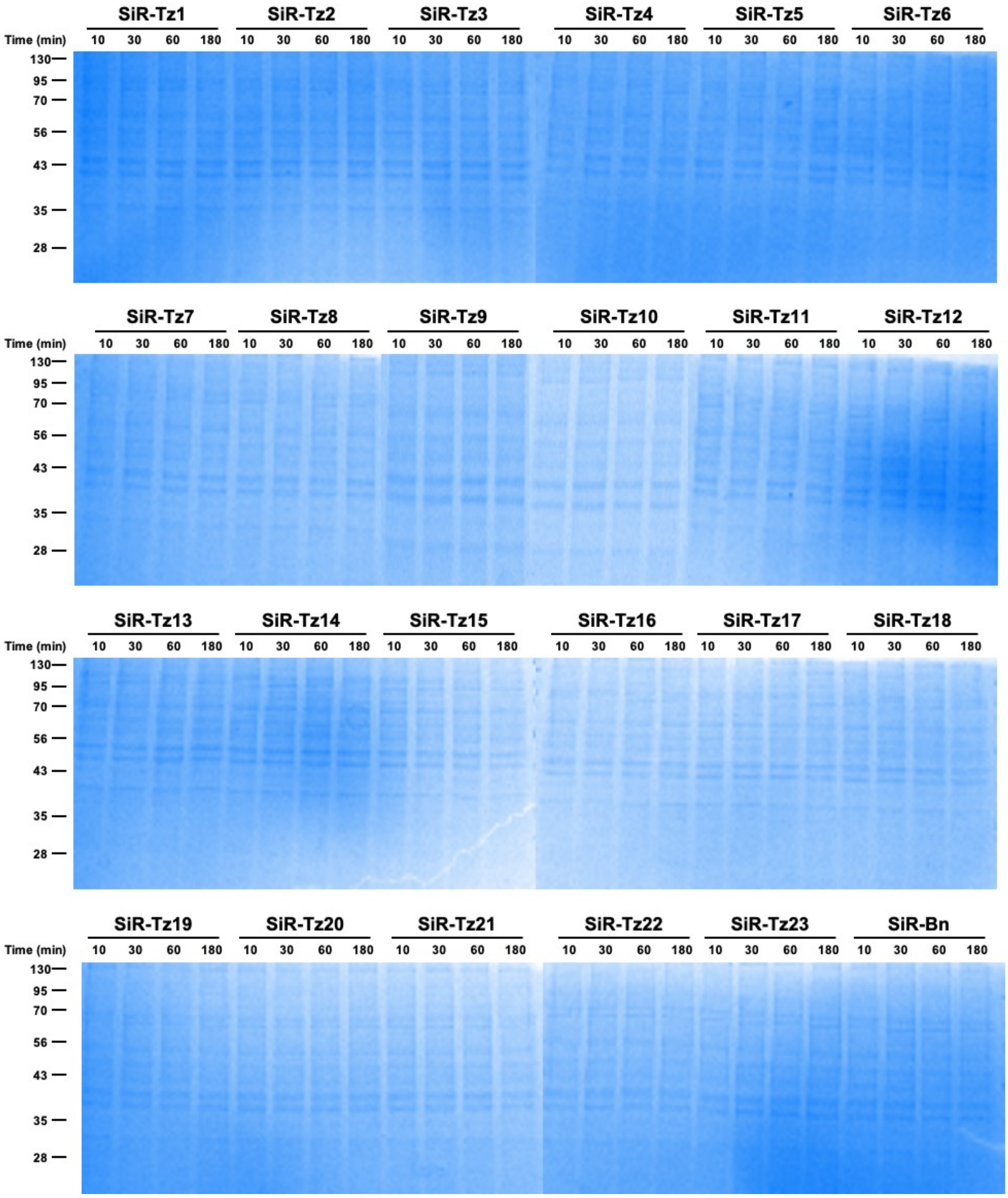

Figure S20. Coomassie Blue staining of Figure S19.

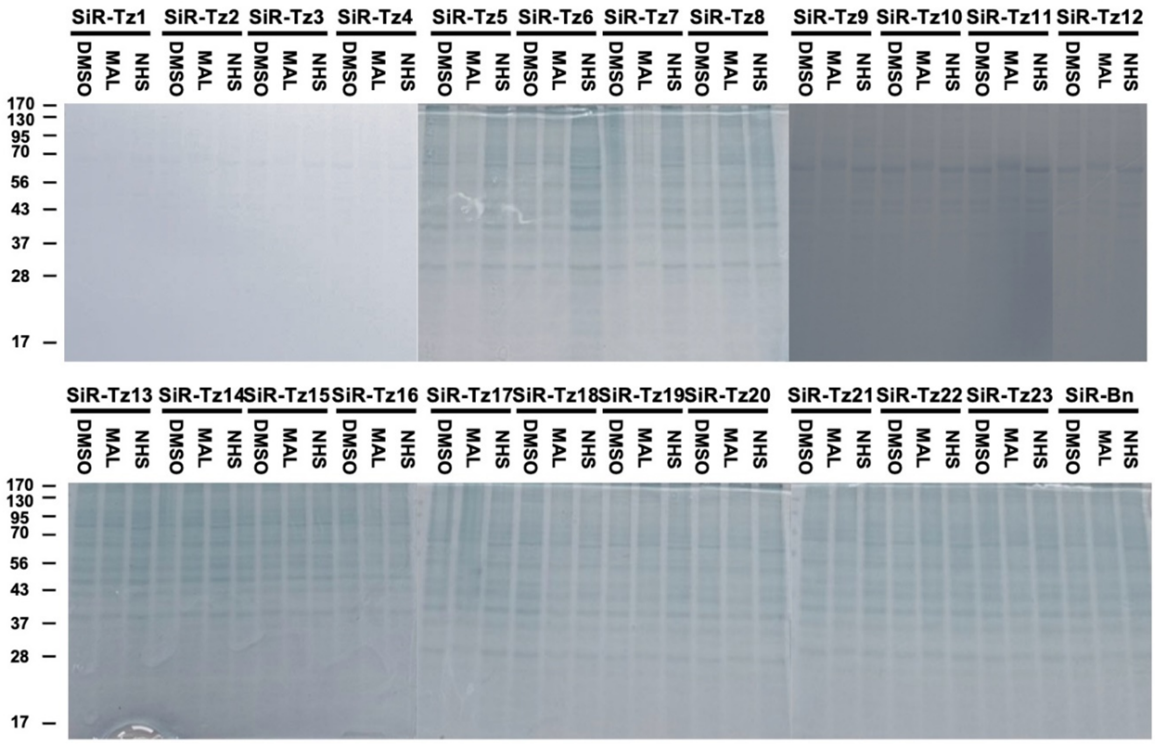

Figure S21. Coomassie Blue staining of Figure 2C.

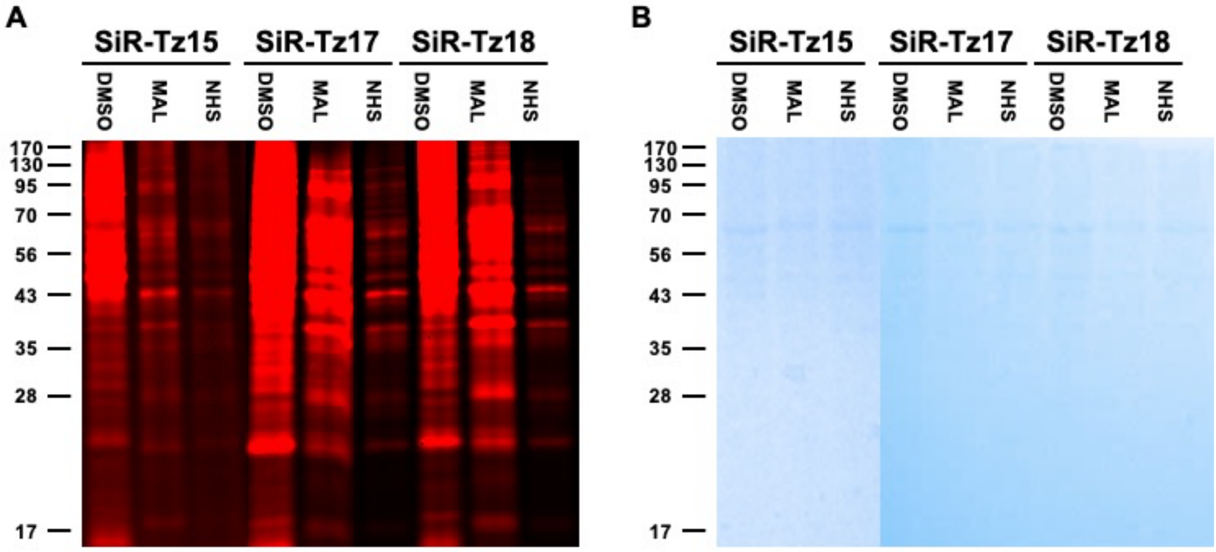

Figure S22. Proteome reactivity of **SiR-Tzs** after removing excess capping reagents by acetone precipitation. (A) HeLa cell lysates were incubated with 1 mM maleimide (MAL) or 1 mM N-hydroxysuccinimide ester (NHS) to perturb **SiR-Tz** proteome reactivity. After capping nucleophiles (thiols and amines) with MAL or NHS, excess reagents were removed by acetone precipitation. The lysates were then incubated with 50 μM **SiR-Tz15**, **SiR-Tz17**, or **SiR-Tz18** for 30 min. Proteome reactivity was analyzed via SDS-PAGE, followed by fluorescent gel scanning. (B) Coomassie Blue staining corresponding to (A).

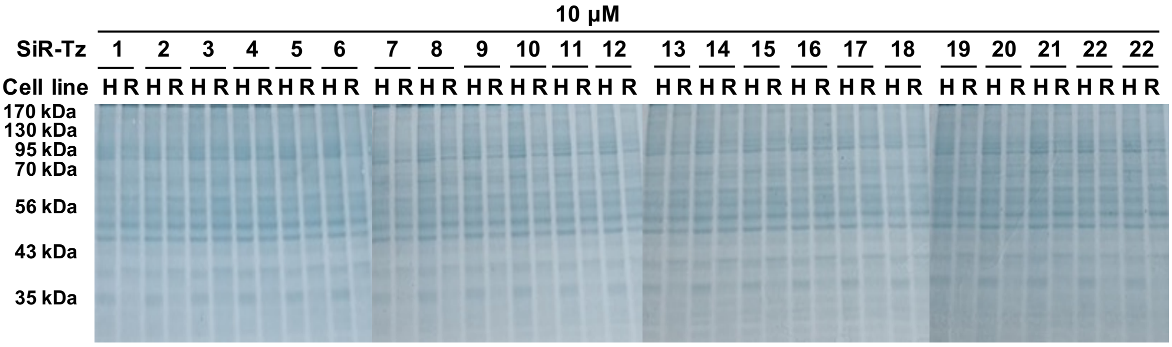

Figure S23. Coomassie Blue staining of Figure 3B.

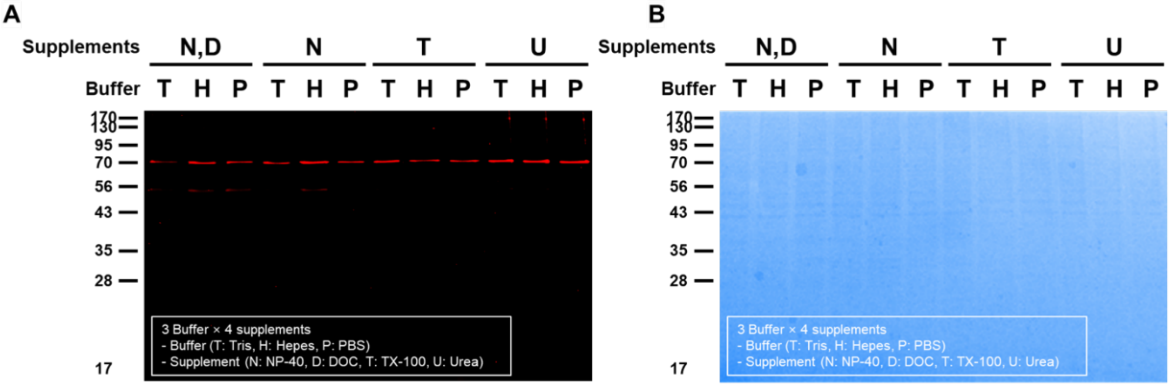

Figure S24. Effect of lysis buffer on fluorescent BTK protein labeling with ibrutinib-*trans*-cyclooctene (IBR-TCO) and **SiR-Tz20** in live cells. (A) Live HL60 cells were treated with IBR-TCO (10 μM) for 1 h, followed by **SiR-Tz20** treatment (10 μM) for 30 min. The cells were lysed using various lysis buffers. Selective BTK labeling was observed with all types of lysis buffer. (B) Coomassie Blue staining corresponding to (A).

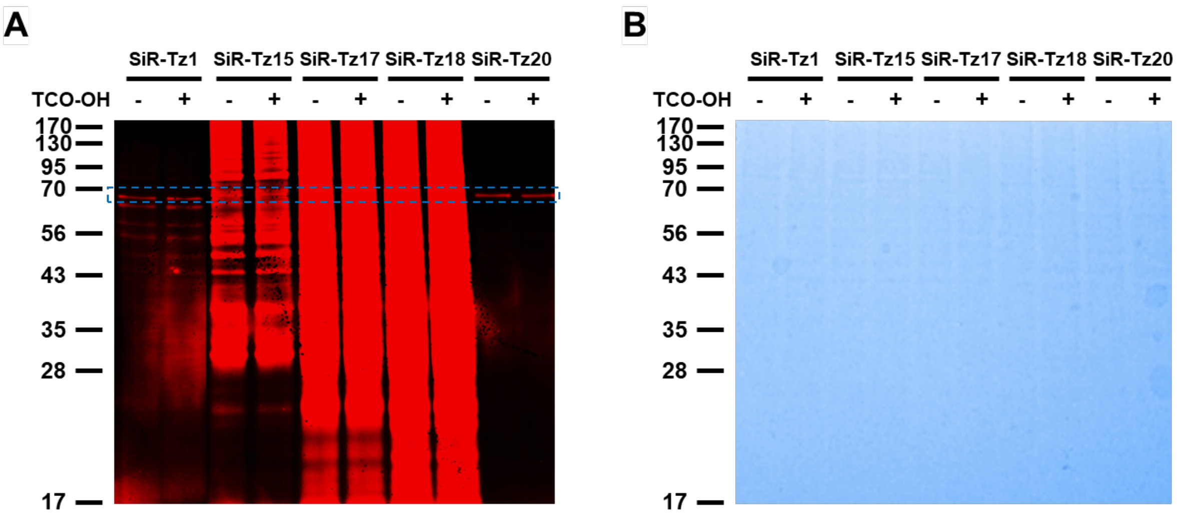

Figure S25. Fluorescent BTK labeling with ibrutinib-*trans*-cyclooctene (IBR-TCO) and **SiR-Tz**s after quenching excess **SiR-Tz**s with TCO-OH in live cells. (A) Live HL60 cells were treated with IBR-TCO (10 µM) for 1 h, followed by treatment with **SiR-Tz1**, **SiR-Tz15**, **SiR-Tz17**, **SiR-Tz18**, and **SiR-Tz20** (10 µM) for 30 min. The cells were then incubated with 10 µM of TCO-OH for 30 min to quench excess **SiR-Tz**s. Selective BTK labeling in HL60 was observed with or without the quenching step, which revealed that there is no background reaction of excess **SiR-Tz**s due to heating step of SDS-PAGE. (B) Coomassie Blue staining corresponding to (A).

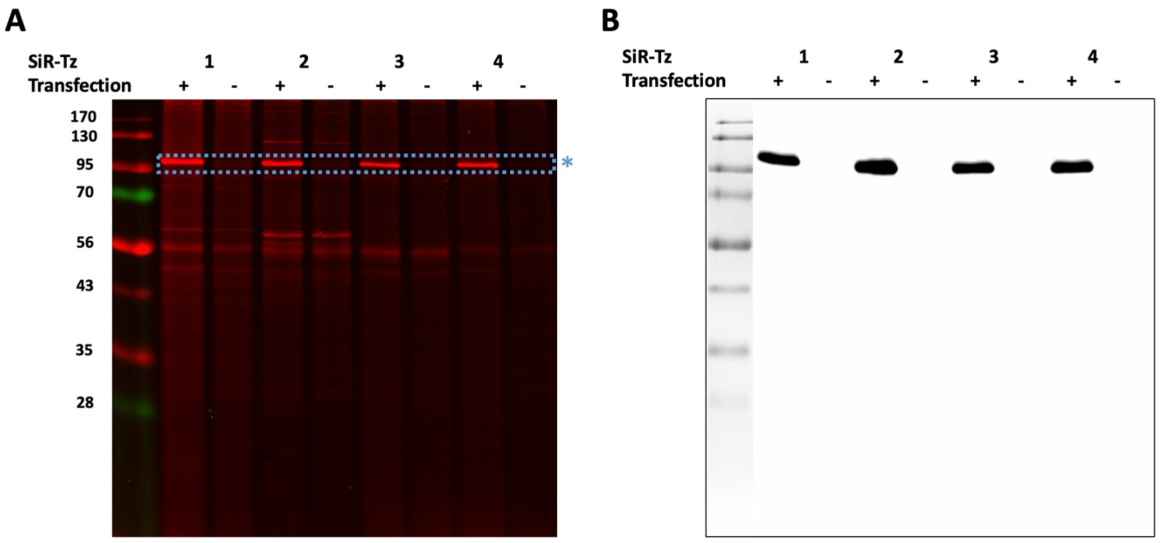

Figure S26. Fluorescent labeling of overexpressed BTK with IBR-TCO and **SiR-Tz**s in live cells. (A) HeLa cells with BTK-GFP were treated with IBR-TCO (10 µM) for 1 h, followed by treatment with **SiR-Tz**s (10 µM) for 30 min. Proteome reactivity was analyzed via SDS-PAGE, followed by fluorescent gel scanning. Overexpressed BTK-GFP bands are marked as blue dotted rectangle with asterisk. (B) Western blot image of (A) using GFP antibody.

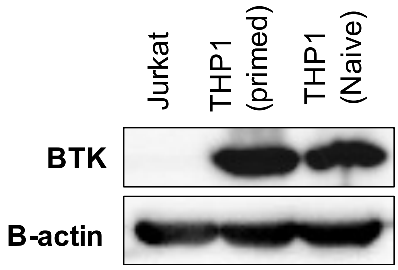

Figure S27. Immunoblot of BTK and β-actin for Jurkat cells, THP1 (primed) cells, and THP1 (native) cells.

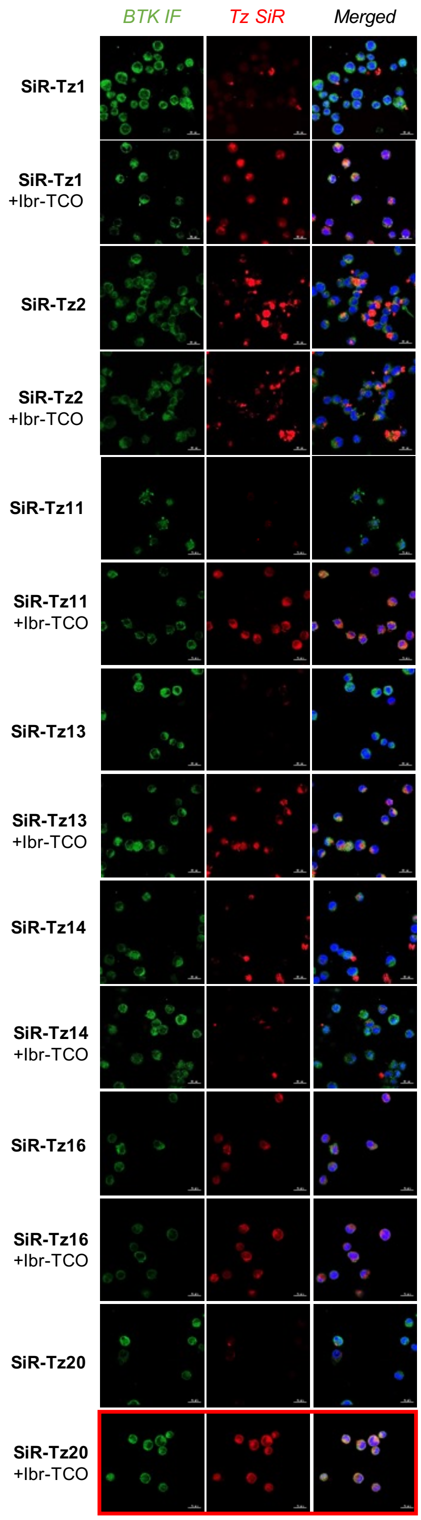

Figure S28. PMA-primed THP-1 dual cells were incubated for 20 min with 10 μM of Ibrutinib-TCO. After cleaning of previous solution, 10 μM of **SiR-Tz**s (1, 2, 11, 13, 14, 16, and 20) were treated for 20 min. After nuclear staining with Hoechst 33342 for 5 min, 40X images were collected by using LSM800 microscope. Scale bar: 20 μm.

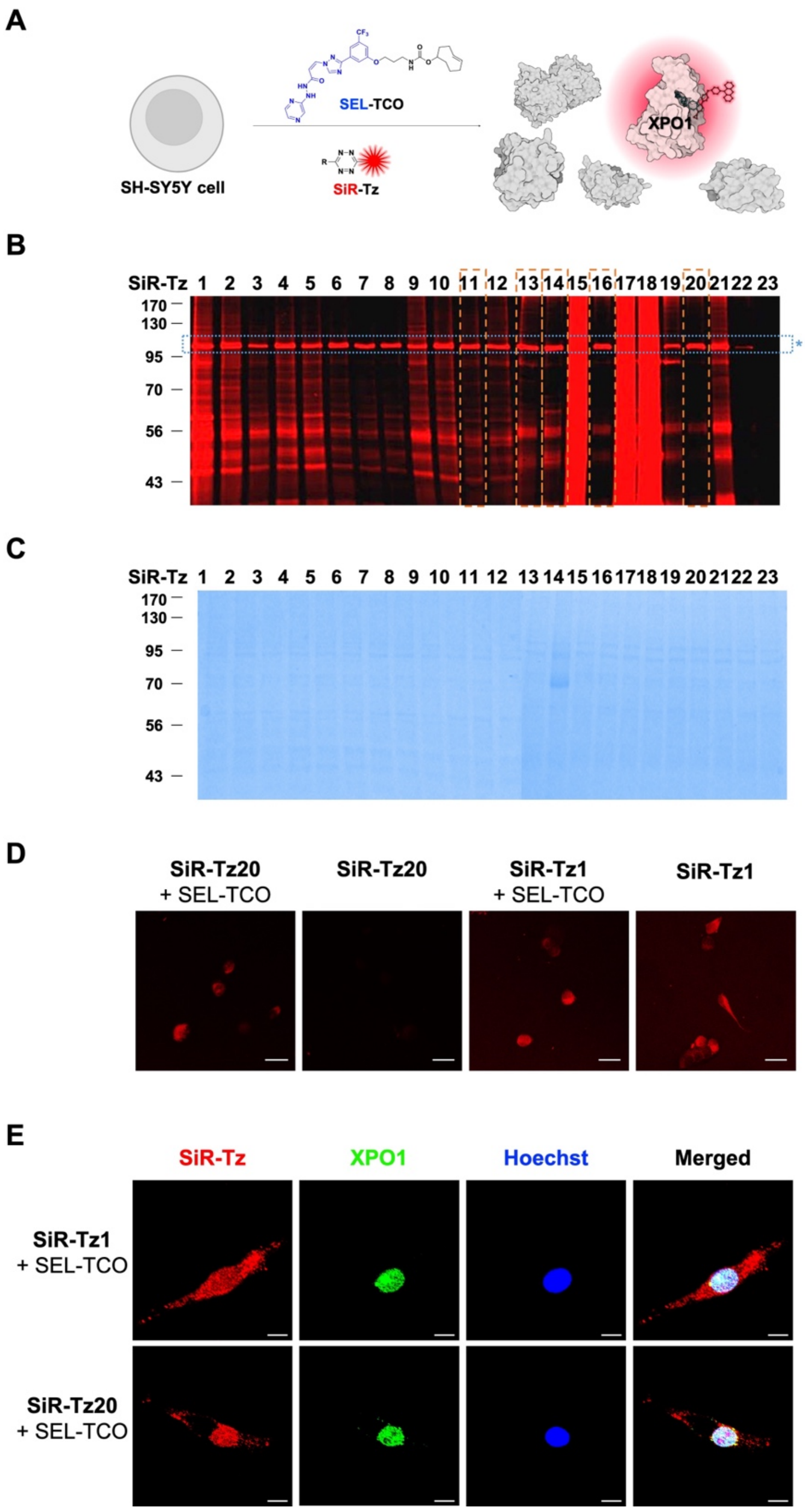
A
SEL-TCO
SiR-Tz
SH-SY5Y cell
XPO1
B
SiR-Tz 1 2 3 4 5 6 7 8 9 10 11 12 13 14 15 16 17 18 19 20 21 22 23
170
130
95
70
56
43
C
SiR-Tz 1 2 3 4 5 6 7 8 9 10 11 12 13 14 15 16 17 18 19 20 21 22 23
170
130
95
70
56
43
D
SiR-Tz20 + SEL-TCO
SiR-Tz20
SiR-Tz1 + SEL-TCO
SiR-Tz1
E
SiR-Tz
XPO1
Hoechst
Merged
SiR-Tz1 + SEL-TCO
SiR-Tz20 + SEL-TCO

Figure S29. Fluorescent XPO1 protein labeling with selinexor-*trans*-cyclooctene (SEL-TCO) and **SiR-Tz**s in live cells. (A) Scheme of XPO1 protein labeling using SEL-TCO and **SiR-Tz**s in live cells. (B) XPO1 imaging by SEL-TCO and **SiR-Tz**s via *in situ* **Tz-TCO** click chemistry-mediated fluorescent protein labeling in live SH-SY5Y cells. SH-SY5Y cells were treated with SEL-TCO (10 μM) for 1 h, followed by **SiR-Tz**s treatment (10 μM) for 30 min in live cells. The minimal-background reagents (**SiR-Tz11**, **SiR-Tz13**, **SiR-Tz14**, **SiR-Tz16**, and **SiR-Tz20**) exhibited successful XPO1 labeling in SH-SY5Y (Yellow dotted rectangles). **SiR-Tz20** showed most selective XPO1 labeling in SH-SY5Y. Endogenous XPO1 bands are marked with blue dotted rectangle with asterisk. (C) Coomassie Blue staining corresponding to (B). (D–E) Fluorescence imaging of SEL-TCO and **SiR-Tz**s for XPO1 imaging. (D) Representative fluorescence images showing the difference in fluorescence signals of **SiR-Tz20** and **SiR-Tz1** in the presence or absence of SEL-TCO in SH-SY5Y cells. Red: SiR dye, Scale bar: 20 μm. (E) Co-localization analysis of XPO1 (green) and **SiR-Tz** (red) fluorescence signals in SH-SY5Y cells. Nucleus was stained by Hoechst33342 (blue). Scale bar: 10 μm.

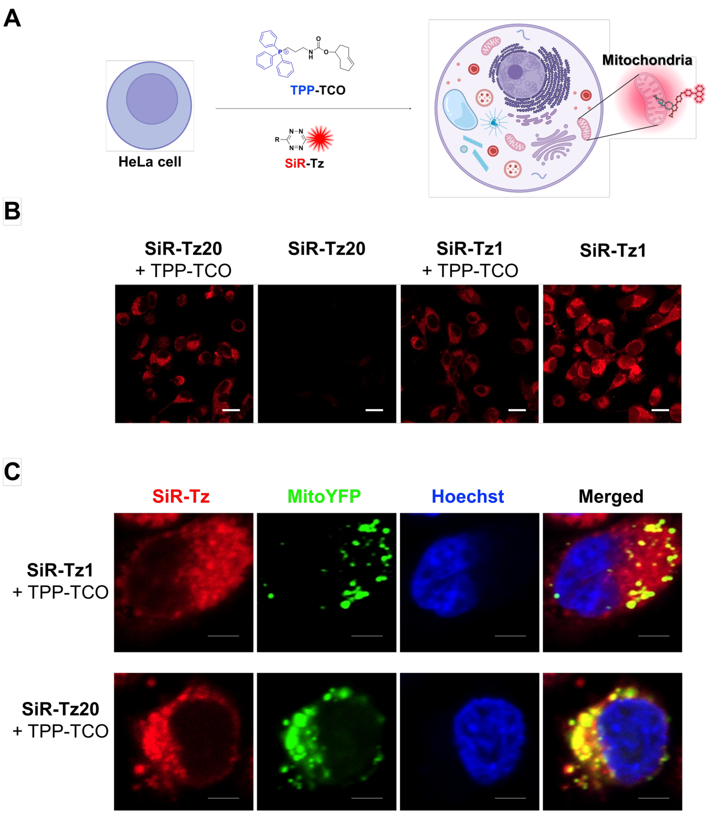

Figure S30. Fluorescent mitochondria labeling with triphenylphosphonium-*trans*-cyclooctene (TPP-TCO) and **SiR-Tz**s in live cells. (A) Scheme of mitochondria labeling using TPP-TCO and **SiR-Tz**s in live cells. (B–C) Fluorescence imaging of TPP-TCO and **SiR-Tz**s for mitochondria imaging. (B) Representative fluorescence images showing the difference in fluorescence signals of **SiR-Tz20** and **SiR-Tz1** in the presence or absence of TPP-TCO in HeLa cells. **SiR-Tz1** labelled the cells non-specifically in the absence of TPP-TCO. Red: SiR dye, Scale bar: 20 μm. (C) Co-localization analysis of mitochondria by mitoYFP (green) and **SiR-Tz** (red) fluorescence signals in HeLa cells. Nucleus was stained by Hoechst33342 (blue). Scale bar: 5 μm.

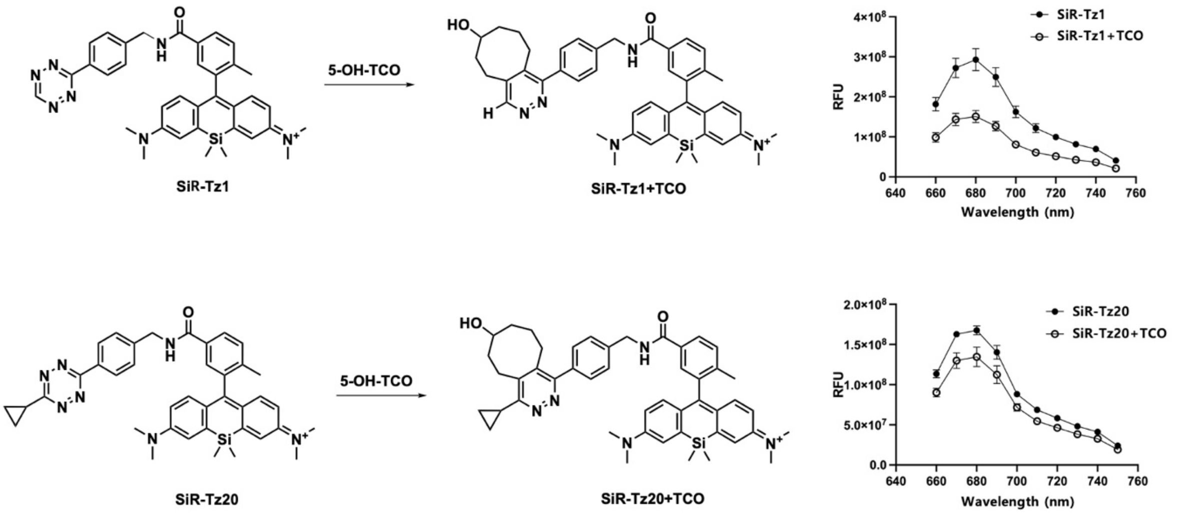

Figure S31. Fluorescent emission spectra of the **SiR-Tz1** and **SiR-Tz20**, and their 5-OH-TCO adducts. The concentrations of **SiR-Tz** derivatives and 5-OH-TCO in the reaction mixture were 10 μM and 100 μM, respectively.

General Information for Chemical Synthesis

Synthesis of TAMRA-Bn

Scheme S1. Synthetic scheme for **TAMRA-Bn**

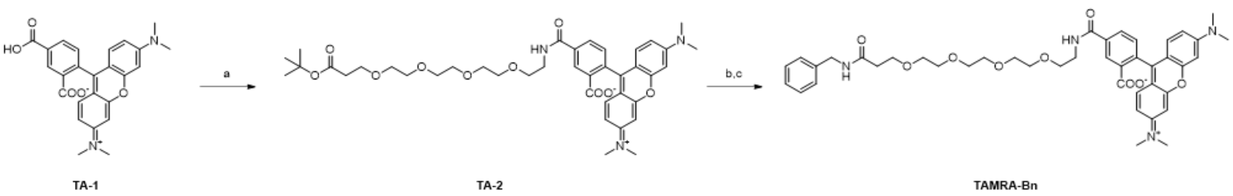

Reagents and conditions: (a) Amino-PEG4-t-butyl ester, HATU, DIPEA, DMF, r.t., 7 h. (b) TFA, DCM, r.t., 4 h. (c) Benzyl amine, HATU, TEA, DMF, r.t., 11 h.

Synthesis of TA-2

5-TAMRA (20 mg, 0.046 mmol, 1 eq) and HATU (35 mg, 0.092 mmol, 2 eq) were dissolved in DMF (500 µL), followed by the addition of TEA (32 µL, 0.23 mmol, 5 eq). The reaction mixture was stirred at room temperature for 10 min. Subsequently, a solution of tert-butyl 1-amino-3,6,9,12-tetraoxapentadecan-15-oate (17 µL, 0.055 mmol, 1.2 eq) in DMF (500 µL) was added, and the reaction mixture was stirred at room temperature for 7 h. The crude product was purified using reverse phase column chromatography (ACN/10 mM Ammonium bicarbonate 0-100%) to afford the title compound as a pink oil (29.4 mg, 86.2%). ^{1}H NMR (400 MHz, Acetonitrile-d_3) δ 8.36 – 8.32 (m, 1H), 8.13 (dt, J = 8.0, 1.1 Hz, 1H), 7.46 (s, 1H), 7.23 (d, J = 8.0 Hz, 1H), 6.58 (d, J = 8.8 Hz, 2H), 6.49 (d, J = 2.5 Hz, 2H), 6.45 (dd, J = 8.8, 2.5 Hz, 2H), 3.68 – 3.44 (m, 18H), 2.96 (d, J = 0.7 Hz, 12H), 2.41 – 2.34 (m, 2H), 1.40 (d, J = 0.8 Hz, 9H); ^{13}C NMR (100 MHz, cd_3cn) δ 171.75, 169.54, 166.50, 154.09, 153.79, 153.51, 139.20, 138.76, 137.32, 134.73, 129.57, 125.25, 124.40, 109.98, 107.14, 98.88, 80.85, 71.03, 71.00, 70.93, 70.89, 70.88, 70.01, 67.40, 40.60, 40.41, 36.89, 28.20.

Synthesis of TAMRA-Bn

TFA/DCM (1/9) and **TA-2** (29.4 mg, 0.04 mmol, 1 eq) were stirred at room temperature for 4 h. Upon completion, the reaction mixture was concentrated in vacuo. The residue and HATU (30.4 mg, 0.08 mmol, 2 eq) were dissolved in dry DMF (1 mL) under an argon atmosphere, followed by the addition of dry TEA (28 µL, 0.2 mmol, 5 eq). The reaction mixture was stirred at room temperature for 10 min, and a solution of benzyl amine (2.57 mg, 0.024 mmol, 0.6 eq)

in dry DMF was added slowly over 11 h using a syringe pump. The crude product was purified using reverse phase column chromatography (ACN/10 mM Ammonium bicarbonate 0-100%) to obtain the title compound as a pink oil (14.8 mg, 48.2%). ^{1}H NMR (400 MHz, Acetonitrile-*d*$_3$) δ 8.36 (dd, *J* = 1.6, 0.8 Hz, 1H), 8.14 (dd, *J* = 8.0, 1.6 Hz, 1H), 7.61 (t, *J* = 5.3 Hz, 1H), 7.33 – 7.16 (m, 6H), 7.13 (s, 1H), 6.58 (d, *J* = 8.8 Hz, 2H), 6.50 (d, *J* = 2.5 Hz, 2H), 6.46 (dd, *J* = 8.9, 2.6 Hz, 2H), 4.32 (d, *J* = 6.1 Hz, 2H), 3.69 – 3.46 (m, 18H), 2.96 (s, 12H), 2.39 (t, *J* = 6.0 Hz, 2H); ^{13}C NMR (100 MHz, Acetonitrile-*d*$_3$) δ 171.08, 168.66, 165.69, 152.84, 152.58, 141.16, 140.97, 139.50, 136.41, 133.95, 128.64, 128.38, 127.85, 127.18, 126.85, 124.28, 123.47, 109.04, 106.14, 98.01, 70.04, 69.92, 69.85, 69.22, 66.92, 42.45, 39.70, 39.50, 36.40. ESI-MS m/z: calc for $C_{43}H_{50}N_4O_9$, $[M]^+$: 766.36, found 766.5

Synthesis of Cy5-Bn

Scheme S2. Synthetic scheme for **Cy5-Bn**

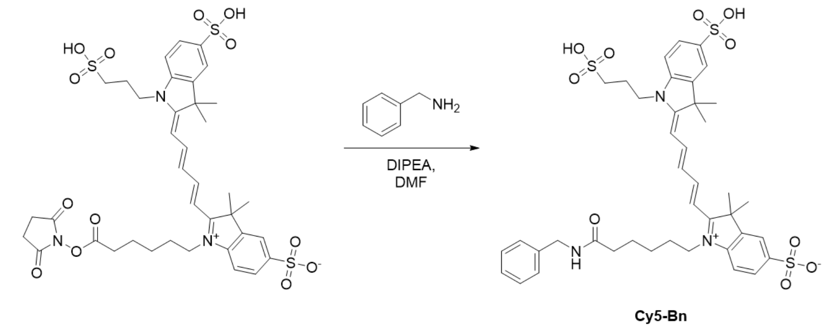

Synthesis of Cy5-Bn

To a solution of Cy5-NHS ester (3 mg, 0.0035 mmol, 1 eq) in DMF(500 μL), 0.1 M benzyl amine (58 μL, 0.0058 mmol, 1.5 eq) solution in DMF and DIPEA (1.8 μL, 0.0105 mmol, 3 eq) were added. The reaction mixture was stirred at room temperature for 13 h. The crude product was purified using reverse phase column chromatography (ACN/10 mM Ammonium bicarbonate 0-100%) to obtain the title compound as a dark blue solid (2.7 mg, 91.8%). ^{1}H NMR (400 MHz, Methanol-*d*$_4$) δ 8.42 (t, *J* = 6.0 Hz, 1H), 8.33 (t, *J* = 13.0 Hz, 2H), 7.92 – 7.84 (m, 4H), 7.47 – 7.40 (m, 2H), 7.34 – 7.18 (m, 5H), 6.72 (t, *J* = 12.4 Hz, 1H), 6.48 (d, *J* = 13.6 Hz, 1H), 6.36 (d, *J* = 13.7 Hz, 1H), 4.34 (dd, *J* = 9.6, 6.0 Hz, 4H), 4.16 – 4.08 (m, 2H), 2.98 (t, *J* = 6.7 Hz, 2H), 2.25 (t, *J* = 7.3 Hz, 2H), 1.82 (dd, *J* = 15.4, 7.5 Hz, 2H), 1.74 (d, *J* = 2.8 Hz,

12H), 1.74 – 1.65 (m, 2H), 1.46 (ddd, J = 15.1, 8.2, 6.6 Hz, 2H), 1.42 – 1.27 (m, 2H). ESI-MS m/z: calc for $C_{41}H_{50}N_3O_{10}S$, $[M+H]^+$: 840.27, found 840.3

Synthesis of SiR-Tzs

Scheme S3. General synthetic scheme for **SiR-Tz**s.

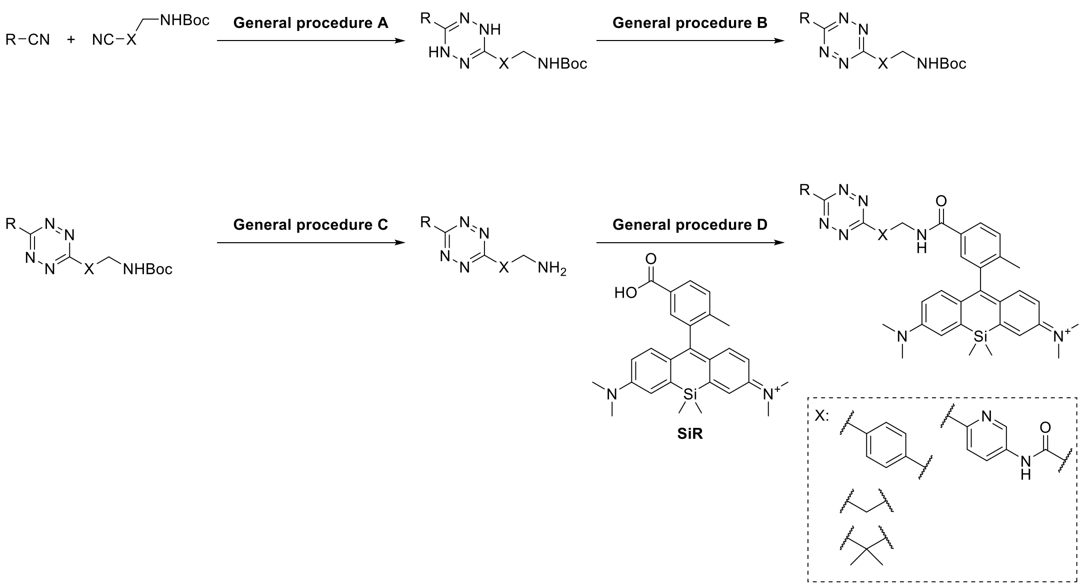

SiR-Tzs were synthesized with four general synthetic procedure (Scheme S3).

1. **General procedure A (Dihydrotetrazine synthesis)**

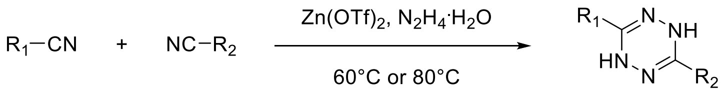

R_1-CN (1 eq), R_2-CN (3–10 eq) and $Zn(OTf)_2$ (0.5 eq) were dissolved in hydrazine monohydrate (50 eq). The reaction mixture was stirred at 60°C or 80°C for 20–24 h. The resulting crude solution containing dihydrotetrazines was used for the next step without further purification.

2. **General procedure B (Dihydrotetrazine oxidation)**

Oxidation a

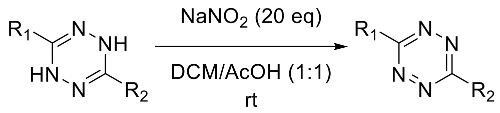

The reaction mixture from **General procedure A** was cooled to room temperature, and a 1:1 (*v*/*v*) DCM/acetic acid solution was added. $NaNO_2$ (20 eq) was added portion-wise in five additions. The reaction mixture was then concentrated under reduced pressure. The residue was dissolved in ethyl acetate (EtOAc) and washed with $NaHCO_3$. The organic layer was dried over $MgSO_4$, and the filtered solution was concentrated under reduced pressure. The desired product was purified by flash column chromatography.

Oxidation b

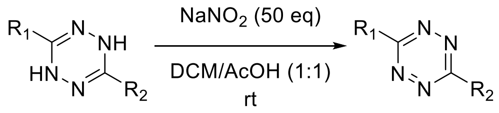

The reaction mixture from **General procedure A** was diluted with EtOAc and allowed to cool to room temperature. The reaction mixture was washed with 1 M HCl, and the aqueous phase was extracted with EtOAc. The organic phase was dried over sodium sulfate and condensed under reduced pressure. The resulting crude residue was dissolved in a DCM/acetic acid (1:1, v/v) solution, followed by the slow addition of $NaNO_2$ (50 eq) in five portions. During the addition, the reaction mixture turned bright red. The reaction mixture was then diluted with DCM, washed with a saturated sodium bicarbonate solution, and the aqueous phase was extracted with DCM. The organic phase was dried over $MgSO_4$ and evaporated under reduced pressure. The desired product was purified by flash column chromatography.

Oxidation c

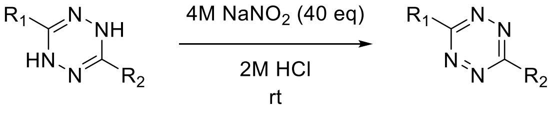

The reaction mixture from **General procedure A** was cooled using ice water, and 4 M $NaNO_2$ (aq.) (40 eq) was added. Then, 2 M HCl (aq.) was added slowly until gas formation ceased (pH 2–3). The reaction mixture was extracted with EtOAc and washed with brine.

The organic layer was dried over Na_2SO_4, and then concentrated under reduced pressure. The desired product was purified by flash column chromatography.

3. General procedure C (Boc deprotection)

Deprotection a

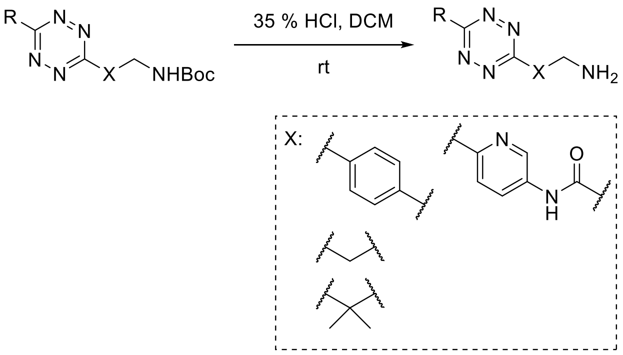

35% hydrogen chloride (HCl) in dioxane was added to the **Boc-Tz**s in DCM and stirred at room temperature. Boc deprotection was confirmed by TLC. The solvent was evaporated under reduced pressure, and the crude mixture was used for the next step without further purification.

Deprotection b

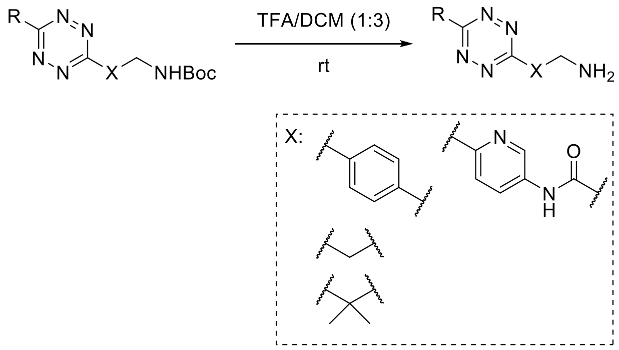

Trifluoroacetic acid (TFA)/DCM (1:3, *v*/*v*) was added to the **Boc-Tz**s in DCM and stirred at room temperature. Boc deprotection was confirmed by TLC. The solvent was evaporated under reduced pressure. The crude mixture was used for the next step without further purification.

4. General procedure D (Amide coupling)

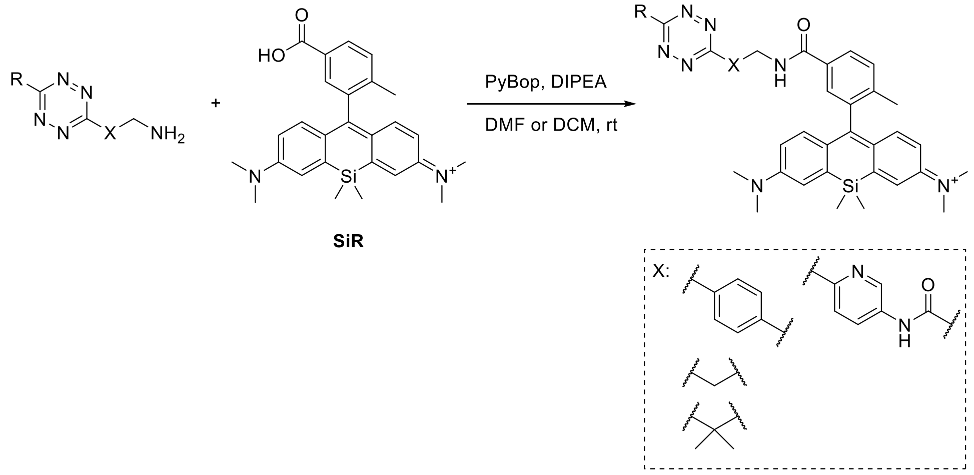

N-(10-(5-carboxy-2-methylphenyl)-7-(dimethylamino)-5,5-dimethyldibenzo[b,e]silin-3(5H)-ylidene)-N-methylmethanaminium (SiR) was prepared as previously reported.[1] SiR and benzotriazol-1-yloxytripyrrolidinophosphonium hexafluorophosphate (PyBOP) were dissolved in either dimethylformamide (DMF) or dichloromethane (DCM) and stirred for 30 minutes. In a separate round-bottom flask, the Boc-deprotected **Tz**s, prepared according to **General procedure C**, were dissolved in either DMF or DCM along with *N*,*N*-diisopropylethylamine (DIPEA). The two mixtures were then combined and stirred overnight.

Synthesis and Characterization of Compounds

Boc-Tz1–8 and **21** were synthesized as previously reported.[2-4]

Synthesis of MFHA

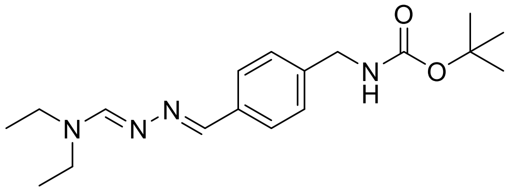

Boc-Tz1 (1 eq) was dissolved in THF, and diethylamine (5 eq) was subsequently added. The reaction mixture was stirred for 2 h at room temperature. The reaction mixture was then evaporated under reduced pressure, and the residue was purified by column chromatography {EtOAc : hexane (Hex) = 0:10 to 1:2}. The product was obtained as a yellow solid (40.8% yield).

^{1}H NMR (400 MHz, Chloroform-*d*) δ 8.32 (s, 1H), 8.15 (s, 1H), 7.62 (d, *J* = 8.2 Hz, 2H), 7.26 (d, *J* = 8.0 Hz, 2H), 4.88 (s, 1H), 4.30 (d, *J* = 5.6 Hz, 2H), 3.36 (s, 4H), 1.45 (s, 9H), 1.20 (t, *J* = 7.2 Hz, 6H). ^{13}C NMR (101 MHz, Chloroform-*d*) δ 160.50, 156.01, 152.17, 139.84, 135.11, 127.75, 127.38, 79.63, 44.62, 28.51. LRMS (ESI) m/z: $[M+H]^+$ calcd for $C_{18}H_{29}N_4O_2$, 333.2; found 333.1

Synthesis of Boc-Tz9

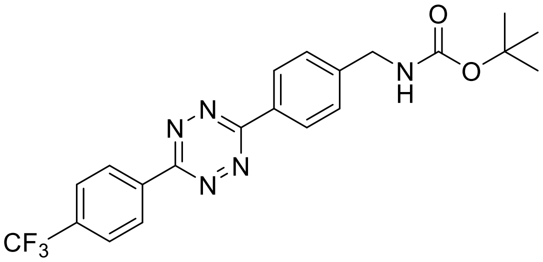

tert-butyl (4-cyanobenzyl)carbamate (2 eq), 4-(trifluoromethyl)benzonitrile (1 eq), $Zn(OTf)_2$ (0.5 eq), and hydrazine monohydrate (50 eq) were used in **General procedure A**. The reaction mixture was stirred at 60°C under Ar for 24 h, and **Oxidation c** was then performed. The resulting mixture was purified by column chromatography (EtOAc : Hex = 1:3 and EtOAc:DCM = 2:98). The product was obtained as a pink solid (11% yield).

NMR data was obtained after deprotection due to its low solubility. ^{1}H NMR (400 MHz, methanol-d_4) δ 8.83 (d, J = 8.2 Hz, 2H), 8.74 (d, J = 8.3 Hz, 2H), 7.98 (d, J = 8.3 Hz, 2H), 7.75 (d, J = 8.2 Hz, 2H), 4.28 (s, 2H); ^{13}C NMR (101 MHz, methanol-d_4) δ 165.24, 164.75, 139.20, 137.22, 134.18, 130.91, 129.80, 129.60, 127.36, 127.31, 127.28, 43.93.

Synthesis of Boc-Tz10

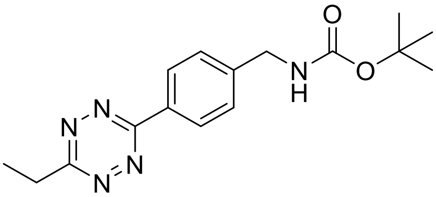

tert-butyl (4-cyanobenzyl)carbamate (1 eq), propionitrile (5 eq), $Zn(OTf)_2$ (0.5 eq), and hydrazine monohydrate (50 eq) were used in **General procedure A**. The reaction mixture was stirred at 80°C for 24 h, and **Oxidation c** was then performed. The resulting mixture was purified by column chromatography (EtOAc:Hex = 1:4). The product was obtained as a pink solid (17 % yield).

^{1}H NMR (400 MHz, Chloroform-d) δ 8.63–8.51 (m, 2H), 7.51 (d, J = 8.2 Hz, 2H), 4.96 (s, 1H), 4.44 (d, J = 6.1 Hz, 2H), 3.40 (q, J = 7.6 Hz, 2H), 1.55 (t, 3H), 1.48 (s, 9H); ^{13}C NMR (101 MHz, Chloroform-d) δ 170.72, 163.99, 155.97, 143.98, 130.78, 128.11, 127.99, 79.72, 44.34, 28.40, 28.33, 12.27.

Synthesis of Boc-Tz 11

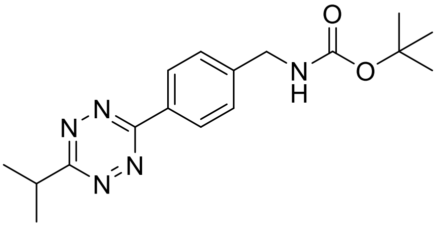

tert-butyl (4-cyanobenzyl)carbamate (1 eq), isobutyronitrile (5 eq), $Zn(OTf)_2$ (0.5 eq), and hydrazine monohydrate (50 eq) were used in **General procedure A**. The reaction mixture was stirred at 80°C for 24 h, and **Oxidation a** was then performed. The resulting mixture was purified by column chromatography (EtOAc:Hex = 3:7). The product was obtained as a pink solid (27 % yield).

^{1}H NMR (400 MHz, Chloroform-*d*) δ 8.63–8.52 (m, 2H), 7.51 (d, *J* = 8.2 Hz, 2H), 4.96 (s, 1H), 4.44 (d, *J* = 6.1 Hz, 2H), 3.69 (p, *J* = 6.9 Hz, 1H), 1.57 (d, *J* = 7.0 Hz, 6H), 1.48 (s, 9H); ^{13}C NMR (101 MHz, Chloroform-*d*) δ 173.61, 164.06, 155.92, 143.87, 130.91, 128.17, 128.05, 79.83, 44.40, 34.24, 28.40, 21.29.

Synthesis of Boc-Tz 12

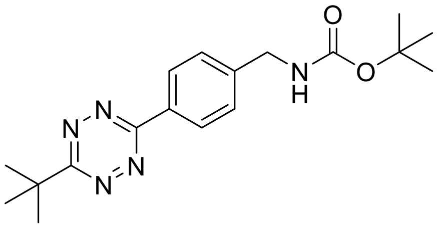

tert-butyl (4-cyanobenzyl)carbamate (1 eq), pivalonitrile (5 eq), $Zn(OTf)_2$ (0.5 eq), and hydrazine monohydrate (50 eq) were used in **General procedure A**. The reaction mixture was stirred at 80°C for 24 h, and **Oxidation a** was then performed. The resulting mixture was purified by column chromatography (EtOAc:Hex = 1:7). The product was obtained as a pink solid (27 % yield).

^{1}H NMR (400 MHz, Chloroform-*d*) δ 8.62–8.53 (m, 2H), 7.51 (d, *J* = 8.1 Hz, 2H), 4.96 (s, 1H), 4.44 (d, *J* = 6.2 Hz, 2H), 1.63 (s, 9H), 1.48 (s, 9H); ^{13}C NMR (101 MHz, Chloroform-*d*) δ 173.61, 164.06, 155.92, 143.87, 130.91, 128.17, 128.05, 79.83, 44.40, 34.24, 28.40, 21.29.

Synthesis of Boc-Tz 13

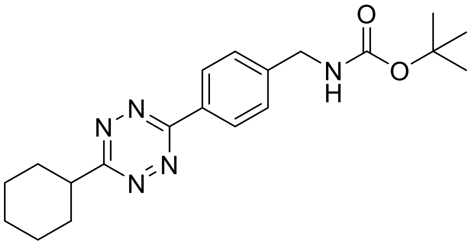

tert-butyl (4-cyanobenzyl)carbamate (1 eq), cyclohexanecarbonitrile (5 eq), $Zn(OTf)_2$ (0.5 eq), and hydrazine monohydrate (50 eq) were used in **General procedure A**. The reaction mixture was stirred at 80°C for 24 h, and **Oxidation a** was then performed. The resulting mixture was purified by column chromatography (EtOAc:Hex = 15:85). The product was obtained as a pink solid (23 % yield).

^{1}H NMR (400 MHz, Chloroform-*d*) δ 8.61–8.51 (m, 2H), 7.56–7.45 (m, 2H), 4.96 (s, 1H), 4.44 (d, *J* = 6.1 Hz, 2H), 3.35 (tt, *J* = 11.7, 3.5 Hz, 1H), 2.25–2.12 (m, 2H), 2.02–1.77 (m, 5H), 1.66–1.26 (m, 12H); ^{13}C NMR (101 MHz, Chloroform-*d*) δ 172.72, 163.99, 155.95, 143.86, 130.92, 128.12, 128.02, 79.77, 44.38, 43.54, 31.41, 28.41, 25.98, 25.66.

Synthesis of Boc-Tz 14

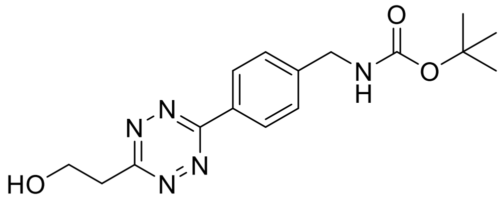

tert-butyl (4-cyanobenzyl)carbamate (1 eq), 3-hydroxypropanenitrile (5 eq), $Zn(OTf)_2$ (0.5 eq), and hydrazine monohydrate (50 eq) were used in **General procedure A**. The reaction was stirred at 60°C for 24 h, and **Oxidation c** was then performed. The resulting mixture was purified by column chromatography (EtOAc:DCM = 3:7). The product was obtained as a pink solid (28 % yield).

^{1}H NMR (400 MHz, Acetone-d_6) δ 8.51 (d, *J* = 8.3 Hz, 2H), 7.59 (d, *J* = 8.1 Hz, 2H), 6.64 (s, 1H), 4.42 (d, *J* = 6.3 Hz, 2H), 4.20 (q, *J* = 6.2 Hz, 2H), 3.94 (t, *J* = 5.8 Hz, 1H), 3.54 (t, *J* = 6.3 Hz, 2H), 1.44 (s, 9H); ^{13}C NMR (101 MHz, Acetone-d_6) δ 169.40, 164.97, 156.91, 146.20, 131.82, 128.83, 128.51, 79.01, 60.81, 60.69, 44.55, 39.18, 28.61.

Synthesis of Boc-Tz 15

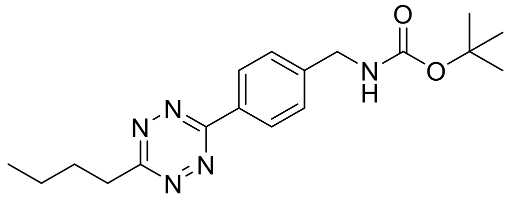

tert-butyl (4-cyanobenzyl)carbamate (1 eq), pentanenitrile (10 eq), $Zn(OTf)_2$ (0.5 eq), and hydrazine monohydrate (50 eq) were used in **General procedure A**. The reaction was stirred at 60°C for 24 h, and **Oxidation b** was then performed. The resulting mixture was purified by column chromatography (EtOAc:Hex = 3:7). The product was obtained as a pink solid (72 % yield).

^{1}H NMR (600 MHz, Chloroform-*d*) δ 8.50 (d, *J* = 8.0 Hz, 2H), 7.46 (d, *J* = 8.1 Hz, 2H), 5.17 (s, 1H), 4.40 (d, *J* = 6.2 Hz, 2H), 3.38–3.29 (m, 2H), 2.00–1.89 (m, 2H), 1.49–1.39 (m, 11H), 0.97 (t, *J* = 7.4 Hz, 3H); ^{13}C NMR (151 MHz, Chloroform-*d*) δ 170.22, 164.00, 156.08, 144.04, 130.87, 128.19, 128.08, 79.57, 44.27, 34.54, 30.38, 28.47, 22.35, 13.82.

Synthesis of Boc-Tz 16

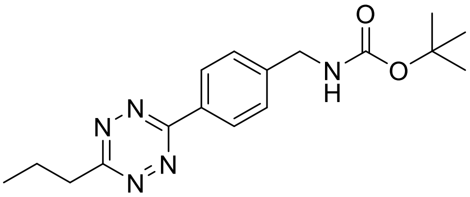

tert-butyl (4-cyanobenzyl)carbamate (1 eq), butyronitrile (10 eq), $Zn(OTf)_2$ (0.5 eq), and hydrazine monohydrate (50 eq) were used in **General procedure A**. The reaction was stirred at 60°C for 24 h, and **Oxidation B** was then performed. The resulting mixture was purified by column chromatography (EtOAc:Hex = 2:8). The product was obtained as a pink solid (70 % yield).

^{1}H NMR (600 MHz, Chloroform-*d*) δ 8.59–8.49 (m, 2H), 7.48 (d, *J* = 8.0 Hz, 2H), 5.08 (s, 1H), 4.42 (d, *J* = 6.2 Hz, 2H), 3.31 (t, *J* = 7.5 Hz, 2H), 2.01 (h, *J* = 7.3 Hz, 2H), 1.46 (s, 9H), 1.07 (t, *J* = 7.4 Hz, 3H); ^{13}C NMR (151 MHz, Chloroform-*d*) δ 170.06, 164.10, 156.07, 144.06, 130.95, 128.27, 128.15, 79.89, 44.48, 36.77, 28.51, 21.80, 13.85.

Synthesis of Boc-Tz 17

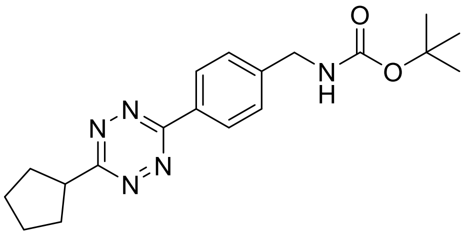

tert-butyl (4-cyanobenzyl)carbamate (1 eq), cyclopentanecarbonitrile (5 eq), $Zn(OTf)_2$ (0.5 eq), and hydrazine monohydrate (50 eq) were used in **General procedure A**. The reaction mixture was stirred at 80°C for 24 h, and **Oxidation B** was then performed. The resulting mixture was purified by column chromatography (EtOAc:Hex = 3:7) and a reverse phase column was performed using a C18 column in HPLC with a gradient increase in the water (containing 0.1%

TFA) to acetonitrile ratio from 0:10 to 10:0. The product was obtained as a pink solid (23 % yield).

^{1}H NMR (400 MHz, Chloroform-*d*) δ 8.56 (d, *J* = 8.4 Hz, 2H), 7.50 (d, *J* = 8.2 Hz, 2H), 4.95 (s, 1H), 4.44 (d, *J* = 6.1 Hz, 2H), 3.55 (tt, *J* = 10.0, 4.2 Hz, 1H), 2.22–2.12 (m, 2H), 2.12–1.99 (m, 2H), 1.99–1.84 (m, 2H), 1.84–1.60 (m, 3H), 1.55 (s, 3H), 1.48 (s, 9H); ^{13}C NMR (101 MHz, Chloroform-*d*) δ 174.26, 164.02, 156.07, 143.92, 131.09, 128.27, 128.19, 79.96, 45.49, 44.52, 33.79, 28.53, 28.38, 26.86.

Synthesis of Boc-Tz 18

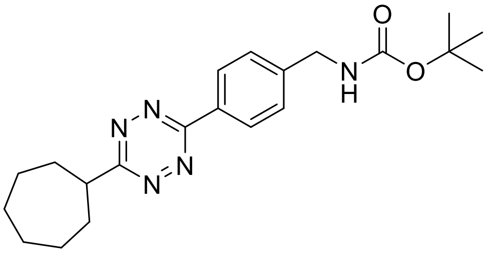

tert-butyl (4-cyanobenzyl)carbamate (1 eq), cycloheptanecarbonitrile (5 eq), Zn(OTf)$_2$ (0.5 eq), and hydrazine monohydrate (50 eq) were used in **General procedure A**. The reaction mixture was stirred at 80°C for 24 h, and **Oxidation A** was then performed. The resulting mixture was purified by column chromatography (EtOAc:Hex = 1: 9). The product was obtained as a pink solid (23 % yield).

^{1}H NMR (400 MHz, Chloroform-*d*) δ 8.62–8.51 (m, 2H), 7.50 (d, *J* = 8.1 Hz, 2H), 4.95 (s, 1H), 4.44 (d, *J* = 6.1 Hz, 2H), 3.55 (tt, *J* = 10.0, 4.2 Hz, 1H), 2.18 (ddd, *J* = 14.7, 7.1, 3.5 Hz, 2H), 2.07–1.98 (m, 2H), 1.93 (ddt, *J* = 13.8, 10.0, 5.2 Hz, 2H), 1.80–1.53 (m, 6H), 1.48 (s, 9H); ^{13}C NMR (101 MHz, Chloroform-*d*) δ 174.26, 164.02, 156.07, 143.92, 131.09, 128.27, 128.19, 79.96, 45.49, 44.52, 33.79, 28.53, 28.38, 26.86.

Synthesis of Boc-Tz 19

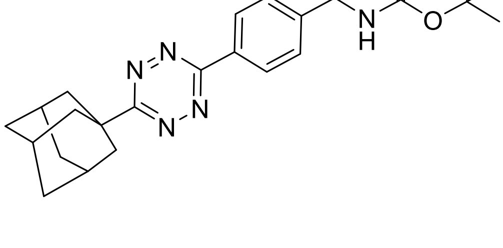

tert-butyl (4-cyanobenzyl)carbamate (1 eq), Cyanoadamantane (2 eq), $Zn(OTf)_2$ (0.5 eq), and hydrazine monohydrate (50 eq) were used in **General procedure A**. The reaction mixture was stirred at 60°C overnight. Oxidation was performed using THF/acetic acid (1:1, *v*:*v*) and $NaNO_2$ (50 eq) in H_2O. The resulting mixture was concentrated under reduced pressure. The residue was redissolved in EtOAc, washed with $NaHCO_3$, dried using $MgSO_4$, and concentrated under reduced pressure. The resulting mixture was purified by column chromatography (EtOAc:Hex = 2:8). The product was obtained as a pink solid (2 % yield).

^{1}H NMR (400 MHz, Chloroform-*d*) δ 8.62–8.52 (m, 2H), 7.50 (d, J = 8.1 Hz, 2H), 4.98 (d, J = 6.3 Hz, 1H), 4.43 (d, J = 6.1 Hz, 2H), 2.26 (d, J = 2.9 Hz, 6H), 2.21 (t, J = 3.1 Hz, 3H), 1.86 (d, J = 3.1 Hz, 6H), 1.48 (s, 9H); ^{13}C NMR (101 MHz, Chloroform-*d*) δ 174.61, 163.45, 155.98, 143.79, 131.03, 128.18, 128.09, 79.86, 44.42, 40.66, 39.54, 36.42, 29.73, 28.43, 28.26.

Synthesis of Boc-Tz 20

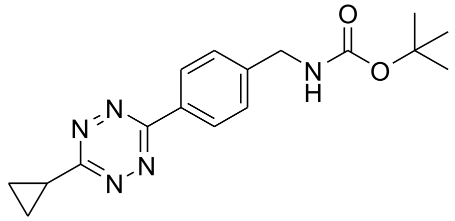

tert-butyl (4-cyanobenzyl)carbamate (1 eq), Cyclopropanecarbonitrile (10 eq), $Zn(OTf)_2$ (0.5 eq), and hydrazine monohydrate (50 eq) were used in **General procedure A**. The reaction was stirred at 60°C for 24 h, and **Oxidation c** was then performed. The resulting mixture was purified by column chromatography (EtOAc:DCM = 3:7). The product was obtained as a pink solid (26 % yield).

^{1}H NMR (400 MHz, Chloroform-*d*) δ 8.51 (d, J = 8.3 Hz, 2H), 7.48 (d, J = 8.2 Hz, 2H), 4.98 (s, 0H), 4.43 (d, J = 6.0 Hz, 2H), 2.70–2.61 (m, 1H), 1.52–1.36 (m, 13H); ^{13}C NMR (101 MHz, Chloroform-*d*) δ 171.24, 164.05, 156.06, 143.71, 131.13, 128.18, 128.03, 79.96, 44.51, 28.53, 15.09, 12.41.

Synthesis of Boc-Tz 22

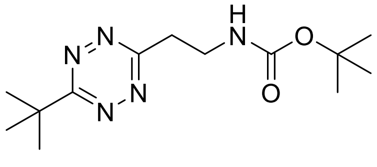

tert-butyl (2-cyanoethyl)carbamate (1 eq), pivalonitrile (5 eq), $Zn(OTf)_2$ (0.5 eq), and hydrazine monohydrate (50 eq) were used in **General procedure A**. The reaction was stirred at 60°C for 24 h, and **Oxidation a** was then performed. The resulting mixture was purified by column chromatography (EtOAc:Hex = 0:100 to 15:85). The product was obtained as a pink solid (5 % yield).

^{1}H NMR (400 MHz, Chloroform-*d*) δ 5.10 (s, 1H), 3.79–3.73 (m, 2H), 3.51 (dd, *J* = 6.8, 5.4 Hz, 2H), 1.59 (s, 9H), 1.40 (s, 9H); ^{13}C NMR (101 MHz, Chloroform-*d*) δ 175.98, 167.79, 155.92, 79.61, 38.29, 37.99, 35.51, 29.28, 28.46.

Synthesis of Boc-Tz 23

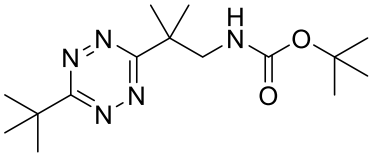

tert-butyl (2-cyano-2-methylpropyl)carbamate (1 eq), pivalonitrile (1 eq), $Zn(OTf)_2$ (0.5 eq), and hydrazine monohydrate (50 eq) were used in **General procedure A**. The reaction was stirred at 60°C for 24 h, and **Oxidation c** was then performed. The resulting mixture was purified by column chromatography (EtOAc:Hex = 8:92 to 1:7). The product was obtained as a pink solid (7 % yield).

^{1}H NMR (400 MHz, Chloroform-*d*) δ 5.17 (s, 1H), 3.60 (d, *J* = 6.7 Hz, 2H), 1.58 (d, *J* = 1.3 Hz, 9H), 1.56 (s, 6H), 1.36 (s, 9H); ^{13}C NMR (101 MHz, Chloroform-*d*) δ 175.94, 167.75, 155.87, 79.57, 38.25, 37.95, 35.47, 29.24, 28.42.

Synthesis of SiR-Tz1

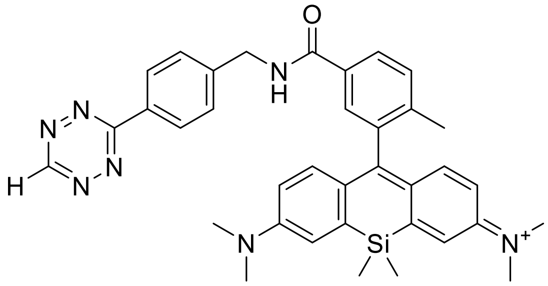

Boc deprotection of **Boc-Tz1** (1 eq) was proceeded according to **Deprotection b**. To a solution of the resulting crude mixture and DIPEA (5 eq) in DMF, HATU (2 eq) was added at room temperature. SiR (1 eq) was then added slowly and the resulting reaction mixture was stirred

for 20 h at room temperature. The reaction mixture was directly purified with reverse phase MPLC using water (containing 0.1% TFA) and acetonitrile, increasing the water : ACN ratio from 0:100 to 50:50. The blue colored fraction was further purified using semi-preparative reverse phase HPLC with a gradient increase in the water (containing 0.1% TFA) to acetonitrile ratio from 0:10 to 10:0 to afford blue solid (28.1 % yield)

^{1}H NMR (400 MHz, methanol-d_4) δ 10.31 (s, 1H), 8.59–8.52 (m, 2H), 8.02 (dd, J = 8.0, 1.9 Hz, 1H), 7.68 (d, J = 1.9 Hz, 1H), 7.61 (d, J = 8.1 Hz, 2H), 7.56 (d, J = 8.1 Hz, 1H), 7.37 (d, J = 2.9 Hz, 2H), 7.06 (d, J = 9.6 Hz, 2H), 6.78 (dd, J = 9.6, 2.8 Hz, 2H), 4.69 (s, 2H), 3.35 (s, 12H), 2.11 (s, 3H), 0.61 (s, 6H); ^{13}C NMR (101 MHz, methanol-d_4) δ 167.72, 166.22, 157.93, 154.45, 148.17, 144.34, 140.75, 140.02, 139.19, 132.11, 131.59, 130.96, 130.44, 128.17, 127.99, 127.82, 127.53, 127.00, 121.02, 114.06, 43.02, 39.62, 18.18, -2.50, -2.58.

Synthesis of SiR-Tz2

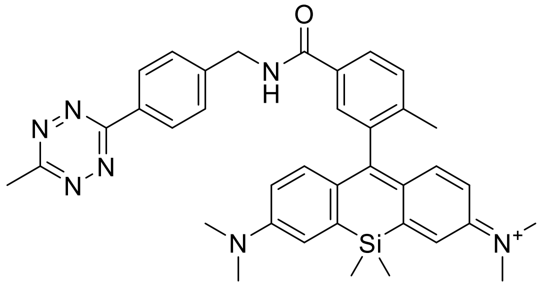

Boc deprotection of **Boc-Tz2** was proceeded according to **Deprotection b**. Boc-deprotected **Tz2** (2 eq) and TEA (6 eq) were dissolved in DMF. SiR-NHS (1 eq) was added to the reaction mixture and stirred at room temperature for 24 h. Without work-up, purification was performed using reverse phase MPLC with a gradient increase in the water (containing 0.1% TFA) to acetonitrile ratio from 0:10 to 1:1 to afford blue solid (92 % yield).

^{1}H NMR (400 MHz, methanol-d_4) δ 8.53–8.49 (m, 2H), 8.02 (dd, J = 8.0, 1.9 Hz, 1H), 7.68 (d, J = 2.0 Hz, 1H), 7.60 (d, J = 8.4 Hz, 2H), 7.56 (d, J = 8.1 Hz, 1H), 7.37 (d, J = 2.8 Hz, 2H), 7.06 (d, J = 9.7 Hz, 2H), 6.79 (dd, J = 9.7, 2.8 Hz, 2H), 4.68 (s, 2H), 3.34 (s, 12H), 3.02 (s, 3H), 2.11 (s, 3H), 0.61 (d, J = 1.5 Hz, 6H); ^{13}C NMR (101 MHz, methanol-d_4) δ 167.74, 167.41, 163.83, 154.45, 148.16, 143.77, 140.76, 140.01, 139.19, 131.59, 131.05, 130.43, 128.07, 127.81, 127.64, 127.51, 126.99, 121.02, 114.06, 43.00, 39.61, 19.73, 18.17, -2.53, -2.58.

Synthesis of SiR-Tz3

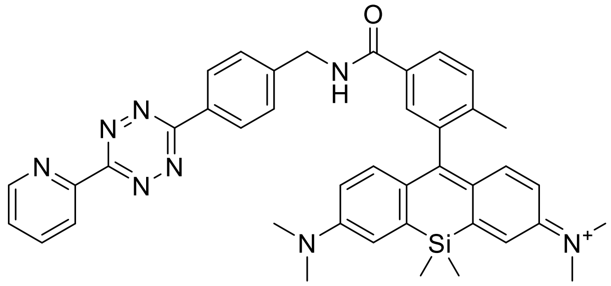

4 M HCl in dioxane and **Boc-Tz3** (1.2 eq) were used in **Deprotection A**. After the deprotection, DIPEA (6 eq), SiR (1 eq), PyBOP (1.3 eq), and DMF were used in **General procedure D** for an overnight reaction. The resulting reaction mixture was directly purified using reverse phase MPLC with a gradient increase in the water (containing 0.1% TFA) to acetonitrile ratio from 0:10 to 1:1 to afford blue solid (25% yield).

^{1}H NMR (400 MHz, methanol-d_4) δ 8.87 (d, J = 4.2 Hz, 1H), 8.74 (d, J = 8.0 Hz, 1H), 8.64 (d, J = 8.5 Hz, 2H), 8.17 (td, J = 7.8, 1.7 Hz, 1H), 8.04 (dd, J = 8.1, 2.0 Hz, 1H), 7.77–7.67 (m, 2H), 7.65 (d, J = 8.5 Hz, 2H), 7.57 (d, J = 8.1 Hz, 1H), 7.38 (d, J = 2.7 Hz, 2H), 7.07 (d, J = 9.6 Hz, 2H), 6.79 (dd, J = 9.7, 2.8 Hz, 2H), 4.72 (s, 2H), 3.35 (s, 12H), 2.12 (s, 3H), 0.61 (s, 6H); ^{13}C NMR (101 MHz, methanol-d_4) δ 169.18, 168.93, 155.68, 149.41, 145.93, 142.04, 141.30, 140.43, 139.83, 132.93, 131.89, 131.78, 129.51, 129.48, 129.23, 128.95, 128.30, 128.26, 122.27, 115.32, 44.33, 40.92, 19.45, -0.88, -1.71.

Synthesis of SiR-Tz4

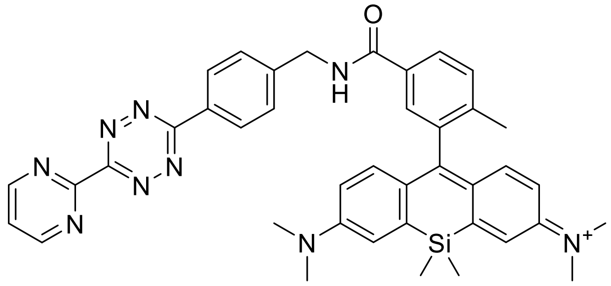

4 M HCl in dioxane and **Boc-Tz4** (1.2 eq) were used in **Deprotection a**. After the deprotection, DIPEA (3 eq), SiR (1 eq), PyBOP (1.3 eq), and DMF were used in **General procedure D** for an overnight reaction. The resulting reaction mixture was directly purified using reverse phase MPLC with a gradient increase in the water (containing 0.1% TFA) to acetonitrile ratio from 0:10 to 1:1 to afford blue solid (14% yield).

^{1}H NMR (400 MHz, Acetone-d_6) δ 9.14 (d, J = 4.9 Hz, 2H), 8.62–8.56 (m, 2H), 8.15 (dd, J = 8.0, 2.0 Hz, 1H), 7.85 (d, J = 1.9 Hz, 1H), 7.78 (t, J = 4.9 Hz, 1H), 7.74–7.68 (m, 2H), 7.61–7.51 (m, 3H), 7.05 (d, J = 9.6 Hz, 2H), 6.84 (dd, J = 9.6, 2.8 Hz, 2H), 4.77 (s, 2H), 3.42 (s, 12H), 2.12 (s, 3H), 0.62 (d, J = 13.0 Hz, 6H); ^{13}C NMR (101 MHz, methanol-d_4) δ 169.17, 168.93, 165.67, 163.69, 160.14, 159.68, 155.68, 149.46, 146.30, 142.06, 141.31, 140.41, 132.94, 131.80, 129.80, 129.62, 129.24, 129.00, 128.27, 124.44, 122.27, 115.31, 44.36, 40.93, 19.46, -0.82, -1.75.

Synthesis of SiR-Tz5

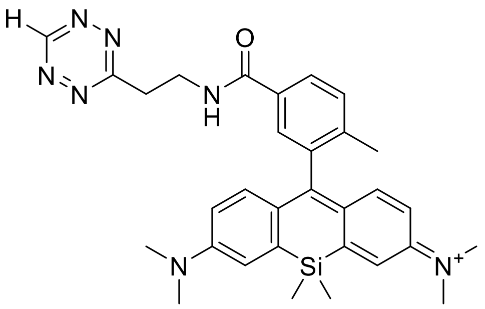

4 M HCl in dioxane and **Boc-Tz5** (2 eq) were used in **Deprotection a**. After the deprotection, DIPEA (3 eq), SiR (1 eq), PyBOP (1.3 eq), and DMF were used in **General procedure D** for an overnight reaction. The reaction mixture was directly purified using reverse phase MPLC using water (containing 0.1% TFA) and acetonitrile (0:10 to 1:1) to afford blue solid (10% yield).

^{1}H NMR (400 MHz, Acetone-d_6) δ 10.38 (s, 1H), 8.20 (t, J = 5.9 Hz, 1H), 7.93 (dd, J = 8.0, 1.9 Hz, 1H), 7.62 (d, J = 1.9 Hz, 1H), 7.57 (d, J = 2.9 Hz, 2H), 7.52 (d, J = 8.0 Hz, 1H), 7.03 (d, J = 9.6 Hz, 2H), 6.87 (dd, J = 9.6, 2.8 Hz, 2H), 4.04–3.95 (m, 2H), 3.61 (t, J = 6.5 Hz, 2H), 3.44 (s, 12H), 2.10 (s, 3H), 0.63 (d, J = 6.7 Hz, 6H); ^{13}C NMR (101 MHz, Acetone-d_6) δ 171.96, 168.66, 166.71, 159.13, 155.30, 148.86, 141.64, 140.03, 139.78, 133.01, 131.19, 128.54, 128.31, 127.82, 122.35, 115.30, 41.02, 38.99, 36.48, 19.38, -1.02, -1.14.

Synthesis of SiR-Tz6

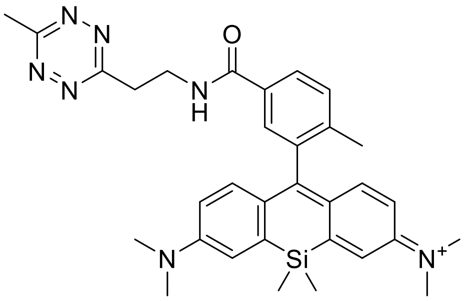

5 M HCl in dioxane and **Boc-Tz6** (1.2 eq) were used in **Deprotection a**. After the deprotection, DIPEA (3 eq), SiR (1 eq), PyBOP (2 eq), and DMF/DCM (1:1, *v*/*v*) were used in **General procedure D** for a 3-hour reaction. The reaction mixture was directly purified using reverse phase MPLC with a gradient increase in the water (containing 0.1% TFA) to acetonitrile ratio from 1:9 to 6:4 to afford blue solid (50% yield).

^{1}H NMR (400 MHz, Acetone-d_6) δ 8.18–8.06 (m, 1H), 7.93 (dd, *J* = 8.0, 1.9 Hz, 1H), 7.63 (d, *J* = 1.8 Hz, 1H), 7.57 (d, *J* = 2.8 Hz, 2H), 7.53 (d, *J* = 8.0 Hz, 1H), 7.04 (d, *J* = 9.6 Hz, 2H), 6.87 (dd, *J* = 9.6, 2.8 Hz, 2H), 4.01–3.94 (m, 2H), 3.56 (t, *J* = 6.6 Hz, 2H), 3.44 (s, 12H), 2.93 (s, 3H), 2.11 (s, 3H), 0.64 (d, *J* = 6.5 Hz, 6H); ^{13}C NMR (101 MHz, Acetone-d_6) δ 168.92, 168.71, 168.30, 155.34, 148.90, 141.69, 140.05, 139.81, 133.09, 131.23, 128.58, 128.33, 127.86, 122.38, 115.33, 41.07, 39.02, 35.77, 21.13, 19.41, -1.00, -1.09.

Synthesis of SiR-Tz7

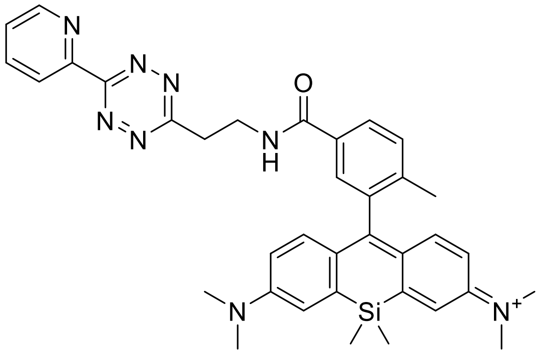

5 M HCl in dioxane and **Boc-Tz7** (1.2 eq) were used in **Deprotection a**. After the deprotection, DIPEA (3 eq), SiR (1 eq), PyBOP (2 eq), and DCM were used in **General procedure D** for a 3-hour reaction. The reaction mixture was then directly purified using reverse phase MPLC with a gradient increase in the water (containing 0.1% TFA) to acetonitrile ratio from 0:10 to 1:1 to afford blue solid (20% yield).

^{1}H NMR (400 MHz, methanol-d_4) δ 8.80 (d, *J* = 4.3 Hz, 1H), 8.62 (d, *J* = 7.9 Hz, 1H), 8.09 (td, *J* = 7.8, 1.7 Hz, 1H), 7.84 (dd, *J* = 8.0, 2.0 Hz, 1H), 7.69 (ddd, *J* = 7.7, 4.9, 1.2 Hz, 1H), 7.51–

7.47 (m, 2H), 7.37 (d, *J* = 2.8 Hz, 2H), 7.01 (d, *J* = 9.6 Hz, 2H), 6.77 (dd, *J* = 9.6, 2.9 Hz, 2H), 3.99 (td, *J* = 6.1, 1.6 Hz, 2H), 3.68 (dd, *J* = 7.0, 5.8 Hz, 2H), 3.35 (s, 12H), 2.08 (s, 3H), 0.60 (d, *J* = 3.8 Hz, 6H); ^{13}C NMR (101 MHz, methanol-d_4) δ 169.38, 169.08, 155.79, 151.35, 149.47, 142.10, 141.22, 140.39, 139.51, 132.88, 131.65, 128.99, 128.69, 128.29, 128.10, 125.20, 122.34, 115.40, 40.94, 39.57, 36.23, 19.46, -1.18, -1.27.

Synthesis of SiR-Tz8

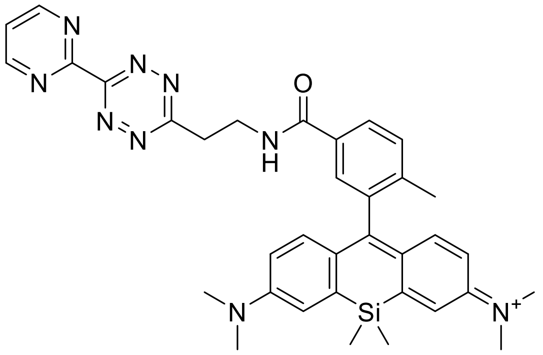

4 M HCl in dioxane and **Boc-Tz8** (2 eq) were used in **Deprotection a**. After the deprotection, DIPEA (6 eq), SiR (1 eq), PyBOP (1.3 eq), and DMF were used in **General procedure D** for 24-hour reaction. The reaction mixture was directly purified using reverse phase MPLC with a gradient increase in the water (containing 0.1% TFA) to acetonitrile ratio from 0:10 to 1:1. The blue fraction was further purified using semi-preparative reverse phase HPLC with a gradient increase in the water (containing 0.1% TFA) to acetonitrile ratio from 0:10 to 10:0 to afford blue solid (4% yield).

^{1}H NMR (400 MHz, methanol-d_4) δ 9.08 (d, *J* = 4.9 Hz, 2H), 7.83 (dd, *J* = 8.0, 2.0 Hz, 1H), 7.75 (t, *J* = 4.8 Hz, 1H), 7.51 (d, *J* = 1.9 Hz, 1H), 7.49 (d, *J* = 8.1 Hz, 1H), 7.36 (d, *J* = 2.8 Hz, 2H), 7.02 (d, *J* = 9.6 Hz, 2H), 6.78 (dd, *J* = 9.7, 2.9 Hz, 2H), 4.01 (t, *J* = 6.3 Hz, 2H), 3.73 (t, *J* = 6.3 Hz, 2H), 3.34 (s, 12H), 2.07 (s, 3H), 0.60 (d, *J* = 0.9 Hz, 6H); ^{13}C NMR (101 MHz, methanol-d_4) δ 169.59, 168.10, 167.75, 162.81, 158.33, 154.45, 148.13, 140.77, 139.88, 139.03, 131.54, 130.31, 127.66, 127.42, 126.97, 123.10, 120.98, 114.06, 39.60, 38.20, 35.03, 18.12, -2.54, -2.59.

Synthesis of SiR-Tz9

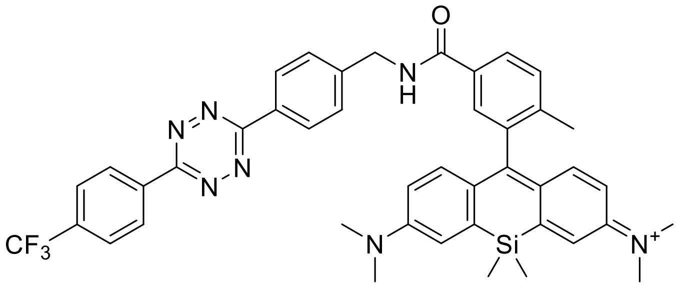

TFA/DCM and **Boc-Tz9** (2 eq) were used in **Deprotection b**. DIPEA (6 eq), SiR (1 eq), PyBOP (2.7 eq), and DMF were used in **General procedure D** for 24-hour reaction. The reaction mixture was then purified using reverse phase MPLC with a gradient increase in the water (containing 0.1% TFA) to acetonitrile ratio from 0:10 to 8:2. The blue fraction was further purified using semi-preparative reverse phase HPLC with a gradient increase in the water (containing 0.1% TFA) to acetonitrile ratio from 0:10 to 10:0 to afford blue solid (25% yield) ^{1}H NMR (400 MHz, methanol-d_4) δ 8.77 (d, J = 8.2 Hz, 2H), 8.65–8.55 (m, 2H), 8.03 (dd, J = 8.1, 1.9 Hz, 1H), 7.95 (d, J = 8.3 Hz, 2H), 7.71 (d, J = 2.0 Hz, 1H), 7.62 (d, J = 8.2 Hz, 2H), 7.56 (d, J = 8.1 Hz, 1H), 7.36 (d, J = 2.9 Hz, 2H), 7.05 (d, J = 9.6 Hz, 2H), 6.77 (dd, J = 9.7, 2.9 Hz, 2H), 4.70 (s, 2H), 3.34 (s, 12H), 2.11 (s, 3H), 0.60 (d, J = 5.5 Hz, 6H); ^{13}C NMR (101 MHz, methanol-d_4) δ 169.07, 165.44, 164.47, 155.79, 149.50, 145.65, 142.09, 141.36, 140.53, 137.26, 134.96, 134.64, 132.94, 132.16, 131.77, 129.52, 129.44, 129.36, 129.18, 128.84, 128.33, 127.27, 127.23, 126.74, 124.03, 122.34, 115.39, 44.38, 40.93, 19.49, -1.15, -1.34.

Synthesis of SiR-Tz10

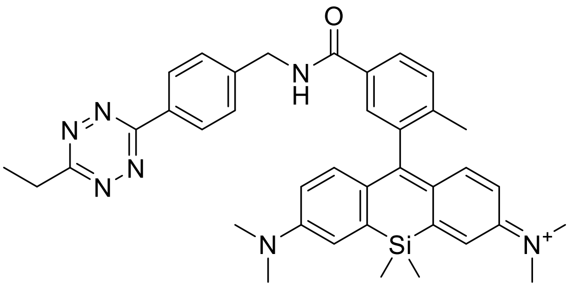

4 M HCl in dioxane and **Boc-Tz10** (1.2 eq) were used in **Deprotection a**. DIPEA (6 eq), amine analog (1.2 eq), SiR (1 eq), PyBOP (2 eq), and DCM were used in **General procedure D** for 1-hour reaction. The reaction mixture was directly purified using reverse phase MPLC with a gradient increase in the water (containing 0.1% TFA) to acetonitrile ratio from 2:8 to 1:1 to afford blue solid (18% yield).

^{1}H NMR (400 MHz, Acetone-d_6) δ 8.56 (t, J = 6.1 Hz, 1H), 8.53–8.48 (m, 2H), 8.12 (dd, J = 8.0, 2.0 Hz, 1H), 7.79 (d, J = 1.9 Hz, 1H), 7.68–7.64 (m, 2H), 7.60–7.56 (m, 3H), 7.08 (d, J = 9.7 Hz, 2H), 6.88 (dd, J = 9.6, 2.9 Hz, 2H), 4.79–4.71 (m, 2H), 3.44 (s, 12H), 3.36 (q, J = 7.6 Hz, 2H), 2.14 (s, 3H), 1.51 (t, J = 7.6 Hz, 3H), 0.64 (d, J = 3.4 Hz, 6H); ^{13}C NMR (101 MHz, Acetone-d_6) δ 171.67, 168.79, 164.89, 155.35, 148.93, 145.45, 141.72, 140.12, 139.88, 133.13, 132.07, 131.30, 129.44, 128.75, 128.58, 128.53, 127.91, 122.35, 115.33, 43.78, 41.04, 19.43, 12.30, -0.98, -1.12.

Synthesis of SiR-Tz11

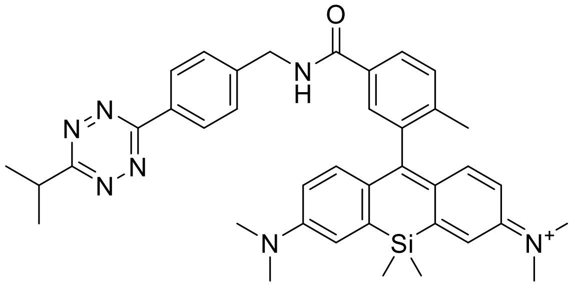

5.5 M HCl in dioxane and **Boc-Tz11** (1.2 eq) were used in **Deprotection a**. DIPEA (3 eq), amine analog (1.2 eq), SiR (1 eq), PyBOP (2 eq), and DCM were used in **General procedure D** for 1-hour reaction. The reaction mixture was directly purified using reverse phase MPLC with a gradient increase in the water (containing 0.1% TFA) to acetonitrile ratio from 2:8 to 4:6 to afford blue solid (55% yield)

^{1}H NMR (400 MHz, Acetone-d_6) δ 8.59 (s, 1H), 8.50 (dd, J = 8.4, 1.7 Hz, 2H), 8.12 (dd, J = 8.0, 1.9 Hz, 1H), 7.80 (d, J = 1.9 Hz, 1H), 7.66 (d, J = 8.1 Hz, 2H), 7.61–7.55 (m, 3H), 7.07 (d, J = 9.6 Hz, 2H), 6.87 (dd, J = 9.6, 2.8 Hz, 2H), 4.74 (s, 2H), 3.72–3.58 (m, 1H), 3.43 (s, 12H), 2.14 (s, 3H), 1.52 (d, J = 7.0 Hz, 6H), 0.64 (d, J = 3.8 Hz, 6H); ^{13}C NMR (101 MHz, Chloroform-d) δ 174.36, 168.73, 166.55, 164.93, 155.30, 148.89, 145.48, 141.69, 140.10, 139.87, 133.08, 132.04, 131.29, 129.44, 128.52, 127.88, 122.36, 115.32, 43.87, 43.75, 41.06, 34.93, 21.47, 19.44, -0.97, -1.12.

Synthesis SiR-Tz12

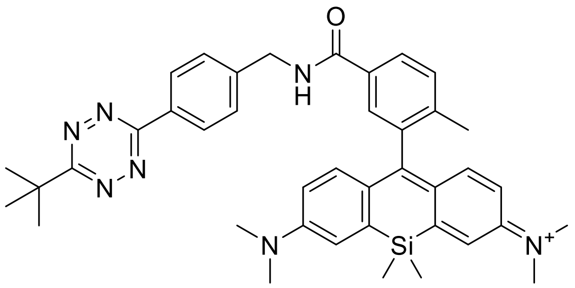

5.5 M HCl in dioxane and **Boc-Tz12** (1.2 eq) were used in **Deprotection a**. DIPEA (3 eq), amine analog (1.2 eq), SiR (1 eq), PyBOP (2 eq), and DCM were used in **General procedure D** for 1-hour reaction. The reaction mixture was directly purified using reverse phase MPLC with a gradient increase in the water (containing 0.1% TFA) to acetonitrile ratio from 2:8 to 4:6 to afford blue solid (32% yield).

^{1}H NMR (400 MHz, Acetone-d_6) δ 8.56 (s, 1H), 8.53–8.49 (m, 2H), 8.12 (dd, J = 8.0, 1.9 Hz, 1H), 7.80 (d, J = 1.9 Hz, 1H), 7.66 (d, J = 8.2 Hz, 2H), 7.60–7.55 (m, 3H), 7.08 (d, J = 9.6 Hz, 2H), 6.88 (dd, J = 9.7, 2.8 Hz, 2H), 4.75 (d, J = 5.2 Hz, 2H), 3.44 (s, 12H), 2.14 (s, 3H), 1.60 (s, 9H), 0.64 (d, J = 3.4 Hz, 6H); ^{13}C NMR (101 MHz, Acetone-d_6) δ 176.19, 168.73, 166.56, 164.31, 155.34, 148.93, 145.41, 141.71, 140.16, 139.89, 133.10, 132.06, 131.32, 129.41, 128.73, 128.58, 127.90, 122.37, 115.33, 43.79, 41.07, 38.39, 29.37, 19.44, -0.96, -1.11.

Synthesis of SiR-Tz13

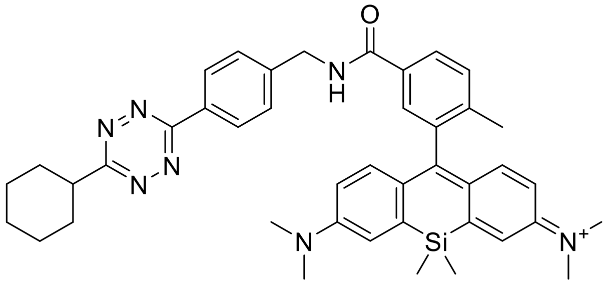

5.5 M HCl in dioxane and **Boc-Tz13** (1.2 eq) were used in **Deprotection a**. DIPEA (3 eq), amine analog (1.2 eq), SiR (1 eq), PyBOP (2 eq), and DCM were used in **General procedure D** for 1-hour reaction. The reaction mixture was directly purified using reverse phase MPLC with a gradient increase in the water (containing 0.1% TFA) to acetonitrile ratio from 2:8 to 6:4 to afford blue solid (21% yield).

^{1}H NMR (400 MHz, methanol-d_4) δ 8.54–8.44 (m, 2H), 8.02 (dd, J = 8.0, 2.0 Hz, 1H), 7.68 (d, J = 1.9 Hz, 1H), 7.61–7.57 (m, 2H), 7.55 (d, J = 8.1 Hz, 1H), 7.37 (d, J = 2.8 Hz, 2H), 7.05 (d,

J = 9.6 Hz, 2H), 6.77 (dd, J = 9.6, 2.9 Hz, 2H), 4.67 (s, 2H), 3.98 (s, 1H), 3.34 (s, 12H), 2.22–2.11 (m, 2H), 2.11 (s, 3H), 1.99–1.89 (m, 2H), 1.88–1.74 (m, 4H), 1.55 (qt, J = 12.7, 3.4 Hz, 2H), 1.46–1.35 (m, 1H), 0.60 (s, 6H); ^{13}C NMR (101 MHz, methanol-d_4) δ 173.96, 169.11, 169.06, 165.44, 155.82, 149.52, 145.15, 142.12, 141.38, 140.56, 132.94, 132.49, 131.80, 129.46, 129.17, 129.05, 128.89, 128.35, 122.39, 115.43, 44.86, 44.37, 40.98, 32.54, 27.13, 26.91, 19.54, -1.18.

Synthesis of SiR-Tz14

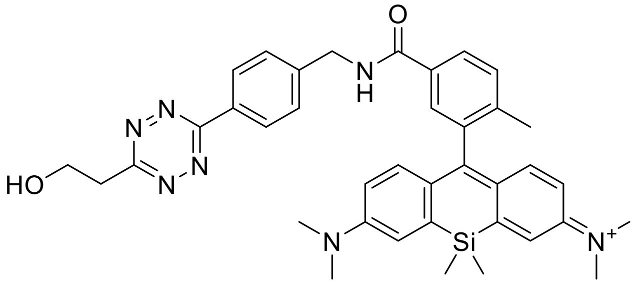

TFA/DCM and **Boc-Tz14** (2 eq) were used in **Deprotection b**. DIPEA (6 eq), SiR (1 eq), PyBOP (2.6 eq), and DMF were used in **General procedure D** for 24-hour reaction. The reaction mixture was then purified using reverse phase MPLC with a gradient increase in the water (containing 0.1% TFA) to acetonitrile ratio from 0:10 to 8:2. The blue fraction was further purified over semi-preparative reverse phase HPLC with a gradient increase in the water (containing 0.1% TFA) to acetonitrile ratio from 0:10 to 10:0 to afford blue solid (30% yield). ^{1}H NMR (400 MHz, methanol-d_4) δ 8.65–8.35 (m, 2H), 8.02 (dd, J = 8.0, 2.0 Hz, 1H), 7.69 (d, J = 1.9 Hz, 1H), 7.64–7.52 (m, 3H), 7.38 (d, J = 2.8 Hz, 2H), 7.06 (d, J = 9.6 Hz, 2H), 6.79 (dd, J = 9.6, 2.9 Hz, 2H), 4.69 (s, 2H), 4.18 (t, J = 6.2 Hz, 2H), 3.53 (t, J = 6.2 Hz, 2H), 3.35 (s, 12H), 2.11 (s, 3H), 0.61 (d, J = 1.2 Hz, 6H); ^{13}C NMR (101 MHz, methanol-d_4) δ 169.61, 169.05, 165.54, 155.83, 149.52, 145.20, 142.12, 141.35, 140.54, 132.94, 132.44, 131.76, 129.42, 129.16, 129.08, 128.84, 122.35, 115.41, 60.99, 49.64, 44.34, 40.92, 39.08, 19.49, -0.05, -1.22.

Synthesis of SiR-Tz15

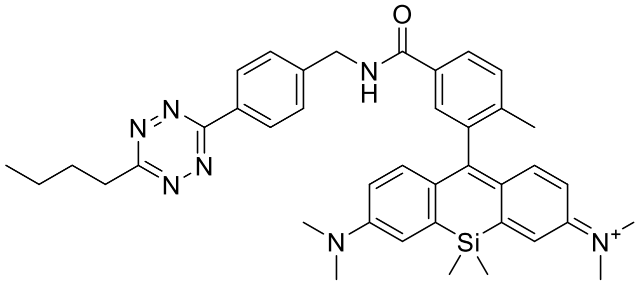

4 M HCl in dioxane and **Boc-Tz15** (2 eq) were used in **Deprotection a**. DIPEA (3 eq), amine analog (1.2 eq), SiR (1 eq), PyBOP (2 eq), and DCM were used in **General procedure D** for an overnight reaction. The reaction mixture was directly purified using reverse phase MPLC with a gradient increase in the water (containing 0.1% TFA) to acetonitrile ratio from 0:10 to 6:4 to afford blue solid (67% yield).

^{1}H NMR (400 MHz, methanol-d_4) δ 9.18 (t, *J* = 5.9 Hz, 1H), 8.52–8.45 (m, 2H), 7.99 (dd, *J* = 8.1, 2.0 Hz, 1H), 7.66 (d, *J* = 1.9 Hz, 1H), 7.60–7.55 (m, 2H), 7.53 (d, *J* = 8.1 Hz, 1H), 7.35 (d, *J* = 2.9 Hz, 2H), 7.03 (d, *J* = 9.6 Hz, 2H), 6.76 (dd, *J* = 9.6, 2.9 Hz, 2H), 4.65 (s, 2H), 3.32 (s, 12H), 2.09 (s, 3H), 1.98–1.87 (m, 2H), 1.47 (h, *J* = 7.4 Hz, 2H), 0.99 (t, *J* = 7.4 Hz, 3H), 0.58 (s, 6H); ^{13}C NMR (101 MHz, methanol-d_4) δ 171.48, 169.12, 169.03, 165.36, 155.82, 149.52, 145.13, 142.11, 141.34, 140.53, 132.94, 132.44, 131.75, 129.41, 129.15, 129.02, 128.84, 128.34, 122.34, 115.40, 44.35, 40.92, 35.34, 31.32, 23.32, 19.49, 14.09, -1.23.

Synthesis of SiR-Tz16

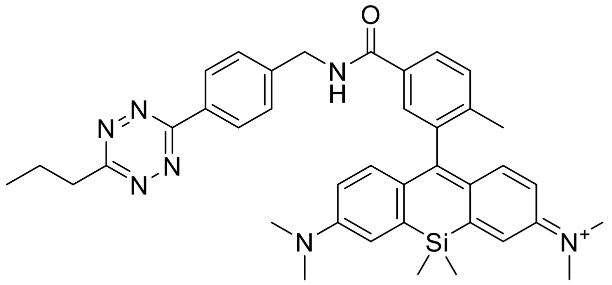

4 M HCl in dioxane and **Boc-Tz16** (2 eq) were used in **Deprotection a**. DIPEA (3 eq), amine analog (1.2 eq), SiR (1 eq), PyBOP (2 eq), and DMF were used in **General procedure D**. It was stirred overnight. Reaction mixture was directly purified using reverse phase MPLC with a gradient increase in the water (containing 0.1% TFA) to acetonitrile ratio from 0:10 to 6:4 to afford blue solid (71% yield).

^{1}H NMR (400 MHz, methanol-d_4) δ 9.18 (s, 1H), 8.55–8.43 (m, 2H), 8.00 (dd, *J* = 8.1, 2.0 Hz, 1H), 7.66 (d, *J* = 1.9 Hz, 1H), 7.60–7.55 (m, 2H), 7.53 (d, *J* = 8.1 Hz, 1H), 7.35 (d, *J* = 2.9 Hz, 2H), 7.04 (d, *J* = 9.6 Hz, 2H), 6.76 (dd, *J* = 9.6, 2.9 Hz, 2H), 4.66 (d, *J* = 4.3 Hz, 2H), 3.32 (s, 12H), 2.09 (s, 3H), 2.04–1.92 (m, 3H), 1.06 (t, *J* = 7.4 Hz, 3H), 0.58 (d, *J* = 1.2 Hz, 6H); ^{13}C NMR (101 MHz, methanol-d_4) δ 171.27, 169.14, 169.04, 165.41, 155.83, 149.52, 145.14, 142.12, 141.34, 140.53, 132.95, 132.46, 131.76, 129.41, 129.16, 129.04, 128.83, 128.35, 122.34, 115.41, 44.34, 40.92, 37.55, 22.57, 19.49, 14.00, -1.22, -1.24.

Synthesis of SiR-Tz17

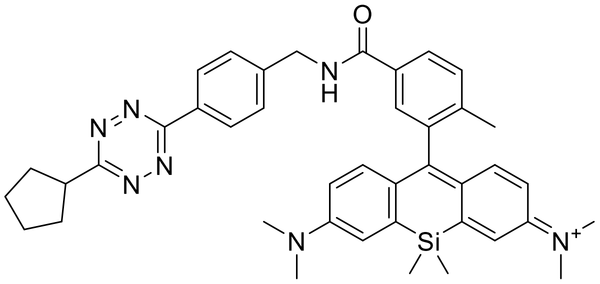

4 M HCl in dioxane and **Boc-Tz17** (2 eq) were used in **Deprotection a**. DIPEA (3 eq), amine analog (1.2 eq), SiR (1 eq), PyBOP (2 eq), and DMF were used in **General procedure D** for an overnight reaction. The reaction mixture was directly purified using reverse phase MPLC with a gradient increase in the water (containing 0.1% TFA) to acetonitrile ratio from 0:10 to 6:4 to afford blue solid (61% yield).

^{1}H NMR (400 MHz, methanol-d_4) δ 8.58 (d, *J* = 8.3 Hz, 1H), 8.52 (d, *J* = 8.3 Hz, 1H), 8.02 (dd, *J* = 8.0, 1.9 Hz, 1H), 7.68 (d, *J* = 1.9 Hz, 1H), 7.67–7.54 (m, 3H), 7.38 (d, *J* = 2.9 Hz, 2H), 7.06 (d, *J* = 9.6 Hz, 2H), 6.79 (dd, *J* = 9.7, 2.8 Hz, 2H), 4.69 (d, *J* = 4.8 Hz, 2H), 3.78 (p, *J* = 8.3 Hz, 1H), 3.35 (s, 12H), 2.94 (s, 1H), 2.80–2.61 (m, 1H), 2.36–2.15 (m, 2H), 2.11 (s, 3H), 2.09–1.80 (m,4H), 0.61 (d, *J* = 1.4 Hz, 6H); ^{13}C NMR (101 MHz, methanol-d_4) δ 155.82, 149.52, 145.07, 142.11, 141.34, 140.54, 132.95, 131.76, 129.49, 129.40, 129.15, 129.01, 128.83, 128.35, 122.34, 115.41, 45.95, 44.35, 41.65, 40.93, 33.77, 27.06, 23.65, 19.49, -1.21, -1.24.

Synthesis of SiR-Tz18

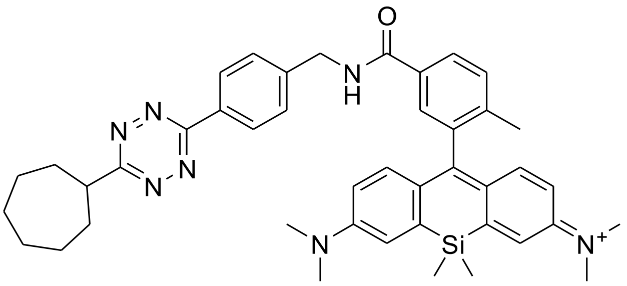

4 M HCl in dioxane and **Boc-Tz18** (1.2 eq) were used in **Deprotection a**. DIPEA (3 eq), amine analog (2 eq), SiR (1 eq), PyBOP (1.3 eq), and DMF were used In **General procedure D** for an overnight reaction. The reaction mixture was directly purified using reverse phase MPLC with a gradient increase in the water (containing 0.1% TFA) to acetonitrile ratio from 0:10 to 6:4 to afford blue solid (53% yield).

^{1}H NMR (400 MHz, methanol-d_4) δ 9.19 (q, *J* = 6.2 Hz, 1H), 8.59–8.53 (m, 1H), 8.49 (dd, *J* = 8.5, 2.0 Hz, 1H), 8.00 (dt, *J* = 8.1, 2.0 Hz, 1H), 7.66 (t, *J* = 1.8 Hz, 1H), 7.63–7.51 (m, 3H), 7.35 (d, *J* = 2.9 Hz, 2H), 7.04 (dd, *J* = 9.6, 0.7 Hz, 2H), 6.76 (ddd, *J* = 9.6, 2.9, 0.6 Hz, 2H), 4.66 (d, *J* = 5.2 Hz, 2H), 3.54–3.41 (m, 1H), 3.32 (s, 12H), 2.97 (ddd, *J* = 15.0, 9.2, 1.6 Hz, 1H), 2.67 (ddd, *J* = 15.0, 9.4, 1.7 Hz, 1H), 2.57–2.50 (m, 1H), 2.25 (t, *J* = 7.4 Hz, 1H), 2.21–2.11 (m, 1H), 2.09 (s, 3H), 2.06–1.41 (m, 6H), 0.58 (d, *J* = 1.4 Hz, 6H); ^{13}C NMR (101 MHz, methanol-d_4) δ 169.14, 169.03, 155.83, 149.52, 142.11, 140.54, 131.76, 129.51, 129.40, 129.16, 129.02, 128.83, 128.34, 122.34, 115.41, 46.70, 44.36, 42.71, 40.92, 34.71, 30.77, 29.73, 29.40, 27.85, 24.06, 19.49, -1.21, -1.24.

Synthesis of SiR-Tz19

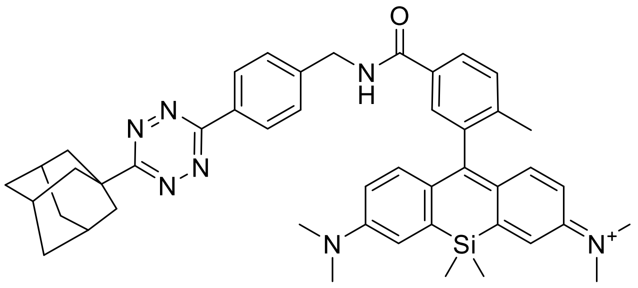

4 M HCl in dioxane and **Boc-Tz19** (1.2 eq) were used in **Deprotection a**. DIPEA (3 eq), amine analog (2 eq), SiR (1 eq), PyBOP (1.3 eq), and DMF were used in **General procedure D** for an overnight reaction. The reaction mixture was directly purified using reverse phase MPLC with a gradient increase in the water (containing 0.1% TFA) to acetonitrile ratio from 0:10 to 6:4 to afford blue solid (40% yield).

^{1}H NMR (400 MHz, methanol-d_4) δ 8.90 (s, 1H), 8.56–8.47 (m, 2H), 8.02 (dd, J = 8.0, 2.0 Hz, 1H), 7.68 (d, J = 1.9 Hz, 1H), 7.59 (d, J = 8.3 Hz, 2H), 7.55 (d, J = 8.1 Hz, 1H), 7.37 (d, J = 2.8 Hz, 2H), 7.06 (d, J = 9.6 Hz, 2H), 6.78 (dd, J = 9.6, 2.8 Hz, 2H), 4.68 (s, 2H), 3.34 (s, 12H), 2.93 (s, 1H), 2.26 (d, J = 2.9 Hz, 7H), 2.18 (s, 4H), 2.11 (s, 3H), 2.02 (d, J = 9.8 Hz, 1H), 1.96–1.84 (m, 2H), 0.60 (d, J = 1.2 Hz, 6H); ^{13}C NMR (101 MHz, methanol-d_4) δ 175.75, 169.13, 169.03, 155.83, 149.52, 145.09, 142.13, 141.35, 140.55, 132.95, 132.53, 131.77, 129.43, 129.16, 129.06, 128.85, 128.34, 122.36, 115.41, 44.35, 41.73, 40.93, 40.64, 37.51, 29.77, 19.49, -1.23.

Synthesis of SiR-Tz20

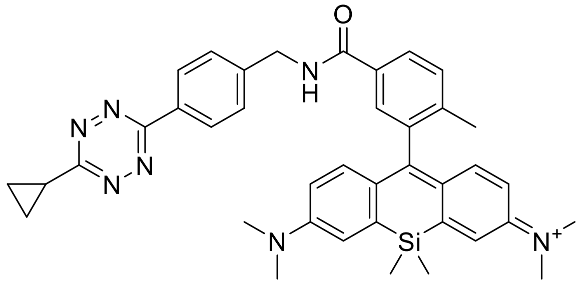

4 M HCl in dioxane and **Boc-Tz20** (1.2 eq) were used in **Deprotection a**. DIPEA (3 eq), amine analog (2 eq), SiR (1 eq), PyBOP (2 eq), and DMF were used in **General procedure D** for an overnight reaction. Reaction mixture was directly purified with reverse phase MPLC with a gradient increase in the water (containing 0.1% TFA) to acetonitrile ratio from 0:10 to 6:4) to afford blue solid (57% yield).

^{1}H NMR (400 MHz, methanol-d_4) δ 8.51–8.36 (m, 2H), 7.99 (dd, J = 8.0, 2.0 Hz, 1H), 7.65 (d, J = 1.9 Hz, 1H), 7.54 (dd, J = 9.5, 8.0 Hz, 3H), 7.35 (d, J = 2.8 Hz, 2H), 7.03 (d, J = 9.6 Hz, 2H), 6.75 (dd, J = 9.7, 2.8 Hz, 2H), 4.64 (s, 2H), 3.32 (s, 12H), 2.67–2.55 (m, 1H), 2.08 (s, 3H), 1.42–1.34 (m, 4H), 0.58 (s, 6H); ^{13}C NMR (101 MHz, methanol-d_4) δ 172.39, 169.12, 169.03, 165.33, 155.82, 149.52, 144.84, 142.11, 141.33, 140.52, 132.95, 132.56, 131.75, 129.38, 129.15, 128.83, 128.77, 128.34, 122.33, 115.40, 44.34, 40.92, 19.48, 15.61, 12.27, -1.23.
HRMS (ESI) m/z: $[M]^+$ calc for $C_{39}H_{42}N_7OSi$, 652.3220; found 652.3265

Synthesis of SiR-Tz21

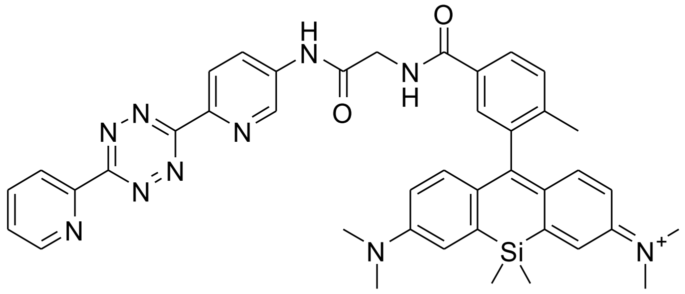

TFA/DCM and **Boc-Tz21** (1.2 eq) were used in **Deprotection b**. DIPEA (6 eq), SiR (1 eq), PyBOP (1.3 eq), and DMF were used in **General procedure D**. It was stirred for 2 h before the addition of boc-deprotected **Boc-Tz21**. It was stirred overnight and purified using reverse phase MPLC with a gradient increase in the water (containing 0.1% TFA) to acetonitrile ratio from 0:10 to 6:4. The blue fraction was further purified using semi-preparative reverse phase HPLC with a gradient increase in the water (containing 0.1% TFA) to acetonitrile ratio from 0:10 to 10:0) to afford blue solid (16% yield).

^{1}H NMR (400 MHz, Acetonitrile-d_3) δ 9.93 (s, 1H), 9.07 (s, 1H), 8.89 (s, 1H), 8.55 (d, J = 21.9 Hz, 2H), 8.38 (d, J = 21.5 Hz, 1H), 8.11–7.93 (m, 2H), 7.75 (s, 1H), 7.64 (s, 1H), 7.49 (d, J = 8.0 Hz, 1H), 7.24 (d, J = 2.8 Hz, 2H), 7.08–6.88 (m, 2H), 6.64 (dt, J = 9.7, 2.6 Hz, 2H), 4.21 (d, J = 5.7 Hz, 2H), 3.26 (s, 12H), 2.04 (s, 3H), 0.56 (d, J = 25.5 Hz, 6H); ^{13}C NMR (101 MHz, Acetonitrile-d_3) δ 170.25, 168.65, 168.09, 164.16, 155.14, 151.94, 151.27, 148.96, 145.24, 141.70, 140.73, 139.93, 139.45, 138.73, 132.40, 131.41, 128.98, 128.62, 127.85, 127.62, 125.63, 125.17, 122.14, 115.16, 45.08, 41.29, 19.52, -0.82, -1.41.

Synthesis of SiR-Tz22

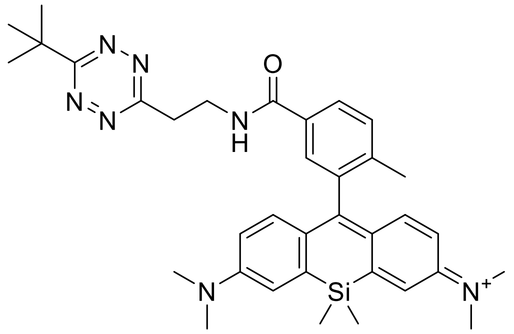

TFA/DCM and **Boc-Tz22** (1.2 eq) were used in **Deprotection b**. DIPEA (6 eq), SiR (1 eq), PyBOP (1.3 eq), and DMF were used in **General procedure D** for 21-hour reaction. The reaction mixture was then purified using reverse phase MPLC with a gradient increase in the water (0.1% TFA) to acetonitrile ratio from 0:10 to 6:4. The blue fraction was further purified using semi-preparative reverse phase HPLC with a gradient increase in the water (0.1% TFA) to acetonitrile ratio from 0:100 to 100:0 to afford blue solid (21% yield).

^{1}H NMR (400 MHz, methanol-d_4) δ 7.82 (dd, J = 8.0, 2.0 Hz, 1H), 7.50 (d, J = 8.1 Hz, 1H), 7.44 (d, J = 2. 0 Hz, 1H), 7.37 (d, J = 2.9 Hz, 2H), 7.02 (d, J = 9.6 Hz, 2H), 6.78 (dd, J = 9.7, 2.8 Hz, 2H), 3.90 (dd, J = 7.0, 5.8 Hz, 2H), 3.55 (dd, J = 7.0, 5.7 Hz, 2H), 3.35 (s, 12H), 2.08 (s, 3H), 1.48 (s, 9H), 0.61 (d, J = 3.7 Hz, 6H); ^{13}C NMR (101 MHz, methanol-d_4) δ 176.78, 169.23, 169.17, 168.74, 155.84, 149.53, 142.17, 141.19, 140.40, 132.92, 131.64, 129.09, 128.61, 128.34, 122.36, 115.43, 40.96, 39.54, 38.68, 36.00, 29.49, 19.48, -1.08, -1.30.

Synthesis of SiR-Tz23

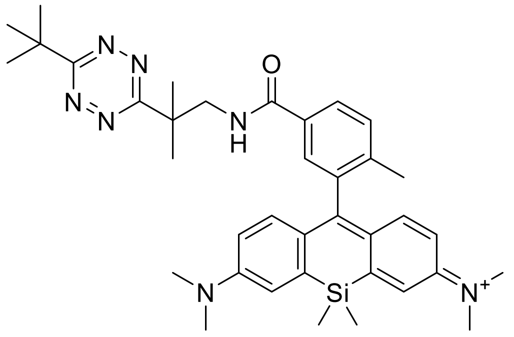

TFA/DCM and **Boc-Tz23** (1 eq) were used in **Deprotection b**. DIPEA (12 eq), SiR (1.5 eq), PyBOP (2 eq), and DCM were used in **General procedure D**. It was stirred for 2 h before the addition of boc-deprotected **Boc-Tz23**. It was stirred overnight and purified using reverse phase MPLC with a gradient increase in the water (0.1% TFA) to acetonitrile ratio from 0:100 to 60:40. The blue fraction was further purified using semi-preparative reverse phase HPLC with

a gradient increase in the water (0.1% TFA) to acetonitrile ratio from 0:100 to 100:0 to afford blue solid (34 % yield).

^{1}H NMR (400 MHz, methanol-d_4) δ 8.69 (s, 1H), 7.76 (d, J = 7.9 Hz, 1H), 7.47 (d, J = 8.0 Hz, 1H), 7.37 (d, J = 2.8 Hz, 3H), 7.00 (d, J = 9.6 Hz, 1H), 6.78 (dd, J = 9.6, 2.8 Hz, 2H), 3.65 (d, J = 5.2 Hz, 2H), 3.34–3.28 (m, 11H), 2.08 (d, J = 2.4 Hz, 3H), 1.67 (d, J = 2.5 Hz, 6H), 1.48 (d, J = 2.3 Hz, 9H), 0.61 (d, J = 3.7 Hz, 6H); ^{13}C NMR (101 MHz, methanol-d_4) δ 171.44, 167.78, 154.46, 148.14, 140.79, 139.76, 139.01, 131.50, 130.18, 127.78, 127.13, 126.94, 120.99, 114.05, 50.09, 42.29, 39.59, 37.18, 28.15, 23.25, 18.10, -2.40, -2.73.

Synthesis of SiR-Bn

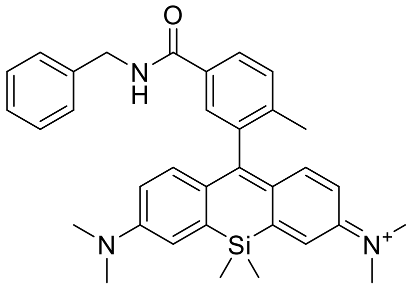

To a solution of SiR (1 eq) and PyBOP (1.1 eq) in DCM, benzylamine (1.2 eq) was added, and the mixture was stirred overnight. The solvent of resulting reaction mixture was then evaporated under reduced pressure, and the residue was purified by reverse phase MPLC with a gradient increase in the water (0.1% TFA) to acetonitrile ratio from 0:100 to 50:50, yielding a blue solid (29% yield).

^{1}H NMR (400 MHz, Methanol-d_4) δ 7.97 (dd, J = 8.0, 2.0 Hz, 1H), 7.64 (d, J = 2.0 Hz, 1H), 7.53 (d, J = 8.1 Hz, 1H), 7.37 (d, J = 2.9 Hz, 2H), 7.36 – 7.28 (m, 4H), 7.26 – 7.21 (m, 1H), 7.05 (d, J = 9.6 Hz, 2H), 6.77 (dd, J = 9.6, 2.9 Hz, 2H), 4.55 (s, 2H), 3.34 (s, 12H), 2.10 (s, 3H), 0.61 (s, 6H); ^{13}C NMR (101 MHz, Methanol-d_4) δ 167.85, 167.57, 154.48, 148.18, 140.79, 139.84, 139.14, 138.80, 131.77, 130.35, 128.20, 127.77, 127.44, 127.30, 127.01, 126.90, 120.99, 114.05, 43.25, 39.59, 18.13, -2.57.

Synthesis of IBR-TCO

Scheme S4. Synthesis of IBR-TCO

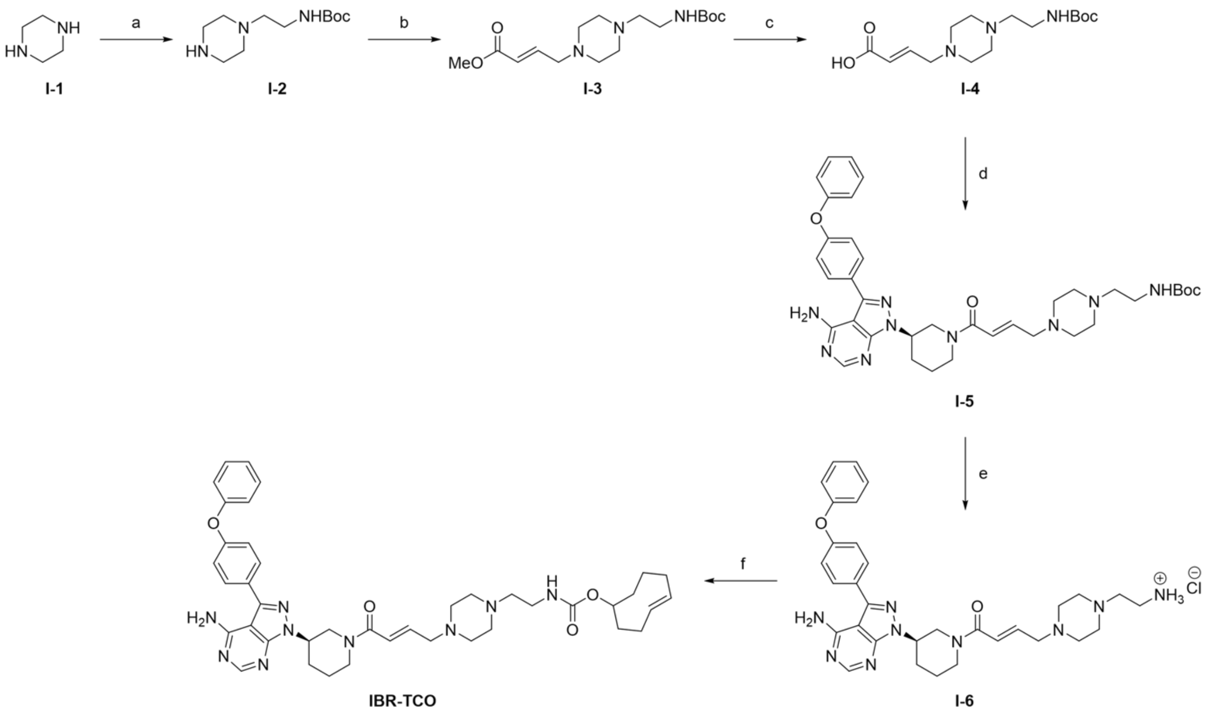

Reagents and conditions: (a) *tert*-butyl (2-bromoethyl)carbamate, NaI, K_2CO_3, acetone, microwave reactor, 100 °C, 3 bar, 10 min; (b) 4-bromobut-2-enoate, TEA, DMSO, r.t., 18 h; (c) 2.5 *N* NaOH solution, THF H_2O, 50 °C, 2 h; (d) (R)-3-(4-phenoxyphenyl)-1-(piperidin-3-yl)-1H-pyrazolo[3,4-d]pyrimidin-4-amin, HATU, DMF, r.t., 18 h; (e) 4 *N* HCl in dioxane, r.t., 18 h; (f) TCO-NHS ester, DIPEA, DCM, r.t., 18 h.

Synthesis of I-2

A solution of piperazine (1.40 g, 16.3 mmol), tert-butyl (2-bromoethyl)carbamate (900 mg, 4.02 mmol), sodium iodide (903 mg, 6.02 mmol) and potassium carbonate (834 mg, 6.03 mmol) in acetone (45 mL) was stirred at 100 °C for 10 min in the microwave reactor (pre-stirring 3 min, 3 bar). The white salt was filtered off and the filtrate was concentrated under reduced pressure. The residue was extracted with dichloromethane and the organic layer was washed with brine, dried over Na_2SO_4 and concentrated *in vacuo*. The residue was purified by column chromatography on silica gel to obtain the title compound as a yellow oil. (1 g, 54.3% yield) ^{1}H NMR (600 MHz, $CDCl_3$) δ 5.06 (s, 1H), 3.29-3.02 (m, 2H), 2.94-2.76 (m, 4H), 2.53-2.07 (m, 6H), 1.45 (s, 9H); ^{13}C NMR (151 MHz, $CDCl_3$) δ 155.92, 79.02, 57.72, 54.25, 45.97, 36.84, 28.36.

Synthesis of I-3

To a solution of *tert*-butyl-(2-(piperazin)-1-yl)ethyl)carbamate (1 g, 4.36 mmol) and TEA (727 μL, 5.23 mmol) in DMSO (20 mL) were added 4-bromobut-2-enoate (624 μL, 5.23 mmol). After the solution was stirred at room temperature for 18 h, the mixture was added ethyl acetate and saturated aqueous solution of $NaHCO_3$. The organic layer was washed with brine, dried over Na_2SO_4 and concentrated *in vacuo*. The residue was purified by column chromatography on silica gel to obtain the title compound as a yellow oil. (600 mg, 42.0% yield) ^{1}H NMR (600 MHz, $CDCl_3$) δ 7.02-6.89 (m, 1H), 6.06-5.93 (d, *J = 15.8 Hz*, 1H), 5.11 (s, 1H), 3.73 (s, 3H), 3.29-3.09 (m, 4H), 2.81-2.27 (m, 10H), 1.45 (s, 9H); ^{13}C NMR (151 MHz, $CDCl_3$) δ 166.30, 155.71, 145.05, 122.73, 78.77, 58.94, 56.92, 52.96, 52.60, 51.28, 36.94, 28.22.

Synthesis I-4

To a solution of ethyl (E)-4-[4-[2-(*tert*-butoxycarbonylamino)ethyl]piperazin-lyl] but-2-enoate (600 mg, 1.83 mmol) in THF/H_2O (1/1, 30 mL) were added 2.5 N NaOH solution (1.10 mL, 2.75 mmol). The solution was stirred at 50 °C for 2 h. The solution was acidified with 1 *N* HCl solution and concentrated *in vacuo*. Methanol was added and the formed solid was filtered off. The filtrate was concentrated *in vacuo* to obtain the title compound as a pale brown oil. (520 mg, 90.6% yield) ^{1}H NMR (600 MHz, CD_3OD) δ 6.92-6.79 (dt, *J = 15.4, 6.5 Hz*, 1H), 6.15-6.04 (d, *J = 15.1 Hz*, 1H), 3.68-2.89 (m, 14H), 1.39 (s, 9H); ^{13}C NMR (151 MHz, CD_3OD) δ 168.63, 158.47, 127.87, 80.77, 58.28, 57.63, 52.33, 50.91, 36.55, 28.72, 28.63.

Synthesis of I-5

To a solution of (E)-4-(4-(2-((*tert*-butoxycarbonyl)amino)ethyl)piperazin-1-yl)but-2-enoic acid (270 mg, 0.862 mmol) and HATU (328 mg, 0.862 mmol) in DMF at room temperature was stirred for 15 min and added (R)-3-(4-phenoxyphenyl)-1-(piperidin-3-yl)-1H-pyrazolo[3,4-d]pyrimidin-4-amine (333 mg, 0.862 mmol). After the reaction mixture was stirred at room temperature for 18 h, ethyl acetate and water added. The organic layers were washed with brine, dried over Na_2SO_4 and concentrated *in vacuo*. The residue was purified by column chromatography on silica gel to obtain the title compound as a pale-yellow solid. (270 mg, 46.0% yield) ^{1}H NMR (600 MHz, CD_3OD) δ 8.31-8.17 (d, *J = 22.7 Hz*, 1H), 7.72-7.60 (d,

J = *8.3 Hz*, 2H), 7.45-7.33 (t, *J* = *7.6 Hz*, 2H), 7.22-7.15 (t, *J* = *7.2 Hz*, 1H), 7.14-7.10 (m, 2H), 7.10-7.02 (d, *J* = *8.3 Hz*, 2H), 6.81-6.21 (m, 2H), 4.84-4.48 (m, 1H), 4.18-3.78 (m, 2H), 3.56-3.35 (m, 1H), 3.27-3.10 (m, 3H), 3.09-2.94 (td, *J* = *14.3, 6.9 Hz*, 1H), 2.81-1.95 (m, 13H), 1.76-1.61 (m, 1H), 1.43 (s, 9H); ^{13}C NMR (151 MHz, CD_3OD) δ 165.98, 158.90, 158.32, 156.74, 156.46, 155.68, 154.13, 153.98, 144.61, 141.72, 130.15, 127.49, 124.29, 123.27, 119.73, 119.19, 98.62, 79.62, 59.56, 57.43, 52.91, 52.75, 49.85, 46.35, 42.56, 37.16, 30.35, 29.98, 28.43, 25.31, 23.89

Synthesis of I-6

To a solution of (R,E)-*tert*-butyl (2-(4-(4-(3-(4-amino-3-(4-phenoxyphenyl)-1H-pyrazolo[3,4-d]pyrimidin-1-yl)piperidin-1-yl)-4-oxobut-2-en-1-yl)piperazin-1-yl)ethyl)carbamate (170 mg, 2.49 mmol) in 1,4-dioxane (20 mL) was stirred at room temperature for 18 h. The formed precipitated solid was filtered and dried in vacuo to obtain the title compound as a pale green solid. (140 mg, 90.9% yield) ^{1}H NMR (600 MHz, DMSO-d_6) δ 9.46 (s, 1H), 8.78-8.49 (m, 4H), 8.14 (s, 1H), 7.72-7.59 (d, *J* = *6.2 Hz*, 2H), 7.50-7.37 (d, *J* = *6.2 Hz*, 2H), 7.26-6.91 (m, 6H), 6.78-6.59 (m, 1H), 4.99-2.95 (m, 19H), 2.37-2.04 (m, 2H), 2.00-1.83 (m, 1H), 1.74-1.52 (m, 1H); ^{13}C NMR (151 MHz, CD_3OD) δ 13C-NMR (151 MHz, METHANOL-D4) δ 166.01, 160.69, 157.69, 153.72, 153.01, 148.71, 147.21, 132.37, 132.06, 131.33, 131.16, 126.98, 125.31, 120.71, 120.10, 98.08, 73.56, 72.44, 68.13, 62.17, 54.51, 50.95, 50.43, 47.32, 43.75, 43.56, 36.07, 30.75, 25.81, 24.30; HR-MS (ESI) m/z: calcd for $C_{32}H_{40}N_9O_2^+$ $[M + H]^+$: 582.3300, found: 582.3292.

Synthesis of I

To a solution of (R,E)-2-(4-(4-(3-(4-amino-3-(4-phenoxyphenyl)-1H-pyrazolo[3,4-d]pyrimidin-1-yl)piperidin-1-yl)-4-oxobut-2-en-1-yl)piperazin-1-yl)ethanaminium chloride (92.5 mg, 0.150 mmol) and DIPEA (78.2 μL, 0.449 mmol) in DCM (10 mL) at room temperature was added (E)-cyclooct-4-en-1-yl (2,5-dioxopyrrolidin-1-yl) carbonate (40 mg, 0.150 mmol) and stirred for 18 h. The reaction mixture was concentrated *in vacuo*. The residue was purified by column chromatography on silica gel to obtain the title compound as a pale-yellow solid. (61 mg, 55.0% yield) ^{1}H NMR (600 MHz, CD_3OD) δ 8.32-8.19 (d, *J* = *17.9 Hz*,

1H), 7.73-7.62 (d, $J = 9.0\ Hz$, 2H), 7.47-7.35 (t, $J = 8.3\ Hz$, 2H), 7.23-7.04 (m, 5H), 6.82-6.42 (m, 2H), 5.71-5.50 (m, 2H), 4.82 (s, 1H), 4.75-4.41 (d, $J = 10.3\ Hz$, 1H), 4.19-3.86 (m, 2H), 3.60-3.33 (m, 2H), 3.30-3.21 (m, 2H), 3.14-3.03 (d, $J = 6.2\ Hz$, 1H), 2.82-1.07 (m, 26H); ^{13}C NMR (151 MHz, CD_3OD) δ 167.46, 167.23, 159.89, 158.73, 157.94, 156.73, 155.21, 145.91, 145.71, 141.16, 136.29, 132.60, 131.23, 131.10, 128.94, 125.23, 125.11, 120.55, 119.98, 99.19, 71.60, 59.85, 58.43, 54.17, 53.47, 53.04, 51.18, 47.30, 47.20, 43.72, 41.95, 38.26, 35.23, 33.71, 31.05, 30.85, 30.33, 29.03, 25.95, 24.24; HR-MS (ESI) m/z: calcd for $C_{41}H_{52}N_9O_4^+$ $[M + H]^+$: 734.4137, found: 734.4136.

Synthesis of SEL-TCO

Scheme S5 Synthesis of SEL-TCO

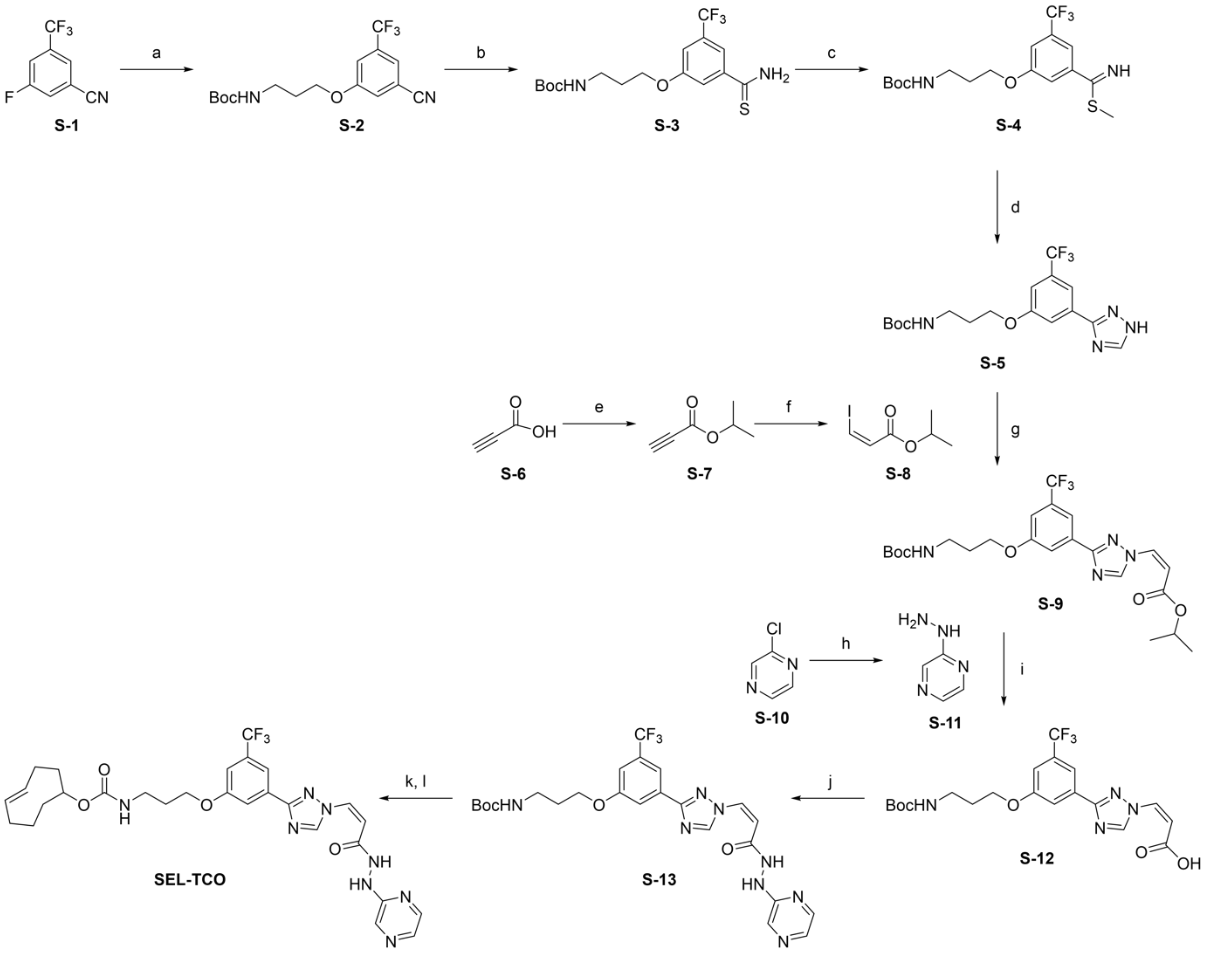

Reagents and conditions: (a) tert-butyl (3-hydroxypropyl)carbamate, NaH, THF, r.t., 16 h. (b) NaSH·XH_2O, $MgCl_2$, DMF, r.t., 6 h.(c) MeI, ether, r.t., 16 h. (d) formohydrazide, DMF, 90°C, 16 h. (e)BF_3OEt_2, IPA, 90°C, 5 h.(f) AcOH, NaI, 70°C, 16 h. (g)DABCO, DMF, r.t., 4 h. (h)

Hydrazine hydrate, EtOH, 60°C, 16 h. (i) LiOH·H_2O, THF, IPA, H_2O, r.t., 2 h. (j) EDCI, DMF, r.t., 4 h (k) TFA, DCM, r.t., 2 h. (l) TCO-NHS, DIPEA, DMF, r.t., 12 h.

Synthesis of S-2

In a 2-neck 100 mL round-bottomed flask, tert-butyl (3-hydroxypropyl)carbamate (2.78 g, 1.58 mmol, 1.2 eq) was dissolved in THF (50 mL), added NaH (2 eq) portion wise in the reaction mixture at -25°C and stirred the reaction mixture at -25°C for 1 hr. after that 3-fluoro-5-(trifluoromethyl)benzonitrile (S-**1**, 2.5 g, 1.32 mmol, 1.0 eq) was added portion wise in the reaction mixture. The reaction mixture was stirred at this temp for 2 hrs. and then at RT overnight. Reaction completion was monitored on TLC using ethyl acetate: hexane (3:7) mobile phase. The reaction mixture was quenched into the ice-water (100 mL) and the compound was extracted in the ethyl acetate (50 mL x 3). The organic layer was washed with brine solution (50 mL x 3) and dried using anhydrous sodium sulphate. The organic layer was concentrated under reduced pressure and the crude product was purified by MPLC (0-100% ethyl acetate in hexane as mobile phase gradient) to afford 2.8 g of desired compound **S-2** (61.5%) as a white solid. ^{1}H NMR (400 MHz, Chloroform-*d*) δ 7.47 (s, 1H), 7.32 (d, J = 14.3 Hz, 2H), 4.70 (s, 1H), 4.08 (t, J = 6.0 Hz, 2H), 3.32 (q, J = 6.3 Hz, 2H), 2.01 (p, J = 6.6 Hz, 2H), 1.42 (s, 9H); ^{13}C NMR (100 MHz, Chloroform-*d*) δ 159.3, 156.03, 133.3, 133.0, 126.7, 124.0, 121.3, 120.7, 118.6, 117.2, 116.3, 114.2, 79.3, 66.6, 37.3, 29.4, 28.2.

Synthesis of S-3

In a 1-neck 100 mL round-bottomed flask, Intermediate **S-2** (2.8 g, 8.13 mmol, 1 eq) was dissolved in DMF (20 mL) added Sodium hydrosulfide hydrate (910 mg, 16.26 mmol, 2.0 eq) and $MgCl_2$ (852 mg, 8.95 mmol, 1.1 eq). The reaction mixture was stirred for 6 h at room temperature. Completion reaction was monitored by TLC using ethyl acetate: hexane (4:6) mobile, then the reaction mixture and quenched into the ice-water (100 mL), and the compound was extracted in the ethyl acetate (100 mL x 3). The organic layer was washed with brine solution (100 mL x 3) and dried over anhydrous sodium sulphate. The organic layer was concentrated under reduced pressure, the crude product was purified by MPLC (0-100% ethyl acetate in hexane as mobile phase gradient) to get the desired compound **S-3** (2.6g, 86.7%) as

a yellow semi-solid. ^{1}H NMR (400 MHz, Chloroform-*d*) δ 8.00 (s, 1H), 7.74 (s, 1H), 7.64 (s, 1H), 7.61 – 7.57 (m, 1H), 7.24 – 7.18 (m, 1H), 4.80 (s, 1H), 4.08 (t, *J* = 5.8 Hz, 2H), 3.30 (q, *J* = 6.5 Hz, 2H), 1.97 (p, *J* = 6.5 Hz, 2H), 1.42 (s, 9H); ^{13}C NMR (100 MHz, Chloroform-*d*) δ 200.8, 158.8, 156.1, 141.3, 132.1, 131.7, 124.7, 122.0, 116.9, 115.6, 114.6, 79.5, 66.3, 37.5, 29.44, 28.3.

Synthesis of S-4

In a 1-neck 100 mL round-bottomed flask, Intermediate **S-3** (2.6 g, 6.88 mmol, 1 eq) was dissolved in diethyl ether (40 mL) and added methyl iodide (2.2 mL, 34.4, mmol, 5 eq) at 0 °C and then the reaction mixture was stirred at room temperature for overnight. Completion of the reaction was monitored by TLC using ethyl acetate: hexane (4:6) mobile phase. Then to the reaction mixture water was added and extracted ethyl acetate (100 mL x 3) The organic layer was washed with brine solution (100 mL x 3) and dried using over anhydrous sodium sulphate, and the obtained crude product was purified by MPLC (0-100% ethyl acetate in hexane as mobile phase gradient) to get the desired compound **S-4** (1.9 g, 67%) as a yellow solid. ^{1}H NMR (400 MHz, Chloroform-*d*) δ 9.46 (s, 1H), 7.63 (s, 1H), 7.50 (s, 1H), 7.21 (s, 1H), 4.74 (s, 1H), 4.09 (t, *J* = 6.0 Hz, 2H), 3.33 (q, *J* = 6.2 Hz, 2H), 2.43 (s, 3H), 2.00 (p, *J* = 6.2 Hz, 2H), 1.43 (s, 9H); ^{13}C NMR (100 MHz, Chloroform-*d*) δ 159.06, 155.98, 140.52, 132.26, 131.93, 124.89, 122.18, 115.63, 113.91, 79.36, 66.23, 37.68, 29.43, 28.37, 12.90.

Synthesis of S-5

In a 2-neck 50 mL round-bottomed flask, intermediate **S-4** (1.9 g, 4.85 mmol, 1 eq) was dissolved DMF (10 mL) and added formic hydrazide (582 mg, 9.69 mmol, 2 eq) and reaction mixture was refluxed to 90 0 °C for 12 h. Completion of the reaction was monitored by TLC using methanol: DCM (1:9) mobile phase. The reaction mixture was brought to room temperature and quenched into the ice water (50 mL) and the compound was extracted in the Ethyl acetate (50 mL x 3). The organic layer was washed with brine solution (50 mL x 3) and dried using anhydrous sodium sulphate, the obtained crude product was purified by MPLC (0-10% Methanol in DCM as mobile phase gradient) to get the desired compound **S-5** (1.2 g, 64%) as a white solid. ^{1}H NMR (400 MHz, Chloroform-*d*) δ 8.26 (s, 1H), 7.88 (s, 1H), 7.70 (s, 1H),

7.08 (s, 1H), 5.08 (s, 1H), 4.03 (t, J = 5.6 Hz, 2H), 3.39 – 3.24 (m, 2H), 1.95 (p, J = 6.0 Hz, 2H), 1.41 (s, 9H); ^{13}C NMR (100 MHz, Chloroform-*d*) δ 159.2, 156.5, 132.4, 132.1, 131.8, 127.7, 124.9, 122.2, 115.6, 115.2, 112.7, 79.7, 65.9, 37.7, 29.3, 28.3.

Synthesis of S-7

In a 2-neck 100 mL round-bottomed flask equipped with a reflux condenser was added propiolic acid (1 g, 14.3 mmol, 1 eq), iPrOH (10 mL) followed by BF_3OEt_2 (3.5 mL, 28.6 mmol, 2 eq) at RT and then refluxed at 90 °C for 3 hrs. Upon completion, monitored by TLC using pentane: diethyl ether (1:9) mobile phase, cooled the reaction mixture to room temperature. After completion of the reaction, water was added (20ml) extracted with DCM (3 × 50 mL), the organic layer was washed with brine solution (20 mL x 3) and dried over anhydrous sodium sulphate, the organic layer was concentrated under reduced pressure, the crude product was purified by MPLC (0-10% diethyl ether in pentane as mobile phase gradient) to get the desired compound **S-7** (700 mg, 86.7%) as light brown oil. ^{1}H NMR (400 MHz, Chloroform-*d*) δ 5.17 – 5.05 (m, 1H), 2.87 (s, 1H), 1.31 (dt, J = 6.3, 0.9 Hz, 6H).

Synthesis of S-8

In a 1-neck 50 mL round-bottomed flask, added compound **S-7** (700 mg, 6.25 mmol,1 eq), sodium iodide (**S-7**, 1.12 g, 7.5 mmol, 1.2 eq), and acetic acid (10 mL) then stirred the reaction mixture at 70 °C for 16 h. After completion of the reaction monitored by TLC (pentane: diethyl ether (1:9) mobile phase), saturated aqueous Na_2HCO_3 was added (20ml) extracted with DCM (3 × 50 mL), the organic layer was washed with brine solution (20 mL x 3) and dried over anhydrous sodium sulphate, the organic layer was concentrated under reduced pressure, the crude product was purified by MPLC (0-10% diethyl ether in pentane as mobile phase gradient to get the desired compound **S-8** (880 mg, 58.7%) as light brown oil. ^{1}H NMR (400 MHz, Chloroform-*d*) δ 7.39 (d, J = 8.9 Hz, 1H), 6.84 (d, J = 8.9 Hz, 1H), 5.12 (p, J = 6.3 Hz, 1H), 1.29 (d, J = 6.3 Hz, 7H).

Synthesis of S-9

In a 1-neck 50 mL round-bottomed flask, added compound **S-5** (386 mg, 1 mmol, 1 eq) in DMF (5 mL) was added DABCO (225 mg, 2 mmol, 2 eq). The mixture was stirred at RT for 30 min and compound **S-8** (480 mg, 2 mmol, 2 eq) was added dropwise. The mixture was stirred at RT for 3 h. After completion of the reaction, monitored by TLC (6:4 Ethyl acetate: Hexane as mobile phase) the mixture was poured into ice water (50 mL) and extracted with ethyl acetate (50 mLx3). All organic layers were combined, washed with saturated sodium chloride solution, (50 mL x2), and dried anhydrous sodium sulphate. The organic layer was concentrated under reduced pressure, and the crude product was purified by MPLC (0-100% ethyl acetate in hexane as mobile phase gradient) to get the desired compound **S-9** (260 mg, 52.2 %) as an off-white solid. ^{1}H NMR (400 MHz, Chloroform-*d*) δ 9.63 (s, 1H), 7.92 (s, 1H), 7.75 (s, 1H), 7.19 (d, *J* = 10.9 Hz, 1H), 7.12 (s, 1H), 5.63 (d, *J* = 10.9 Hz, 1H), 5.06 (p, *J* = 6.3 Hz, 1H), 4.95 (s, 1H), 4.07 (t, *J* = 6.0 Hz, 2H), 3.30 (d, *J* = 6.1 Hz, 2H), 1.97 (p, *J* = 5.6 Hz, 2H), 1.38 (s, 9H), 1.25 (d, *J* = 6.3 Hz, 6H); ^{13}C NMR (100 MHz, Chloroform-*d*) δ 163.8, 160.9, 159.2, 156.0, 147.6, 133.0, 132.3, 132.0, 125.0, 122.3, 115.8, 115.2, 113.1, 108.1, 79.1, 68.8, 66.1, 37.6, 29.4, 28.3, 21.6.

Synthesis of S-10

In a 1-neck 50 mL round-bottomed flask, added compound **S-9** (260 mg, 0.522 mmol, 1eq) in THF: IPA: H_2O (1:1:1, 3ml), followed by the addition of LiOH.H_2O (2 eq) and stirred the reaction mixture at room temperature for 2h, After completion of the reaction monitored by TLC (10% MeOH in DCM as mobile phase), solvents were evaporated and the obtained residue washed with ether and acidified with 1N HCl, extracted with EtOAc (20ml X2) and purified by flash column chromatography (0-10% MeOH, in DCM as mobile phase) to get the desired product **S-10** (170 mg, 71.4 %) as a off white solid. ^{1}H NMR (400 MHz, DMSO-d_6) δ 9.41 (s, 1H), 7.86 (s, 1H), 7.79 (s, 1H), 7.46 (d, *J* = 10.3 Hz, 1H), 7.34 (s, 1H), 6.98 (s, 1H), 5.94 (d, *J* = 10.3 Hz, 1H), 4.15 (t, *J* = 5.6 Hz, 2H), 3.23 – 3.07 (m, 2H), 1.97 – 1.84 (m, 2H), 1.39 (s, 9H); ^{13}C NMR (100 MHz, DMSO-d_6) δ 166.4, 160.2, 159.9, 156.0, 148.0, 132.6, 131.6, 131.3, 131.0, 125.5, 122.8, 116.0, 114.8, 112.7, 112.2, 77.9, 66.5, 37.1, 29.4, 28.6.

Synthesis of S-12

In a 1-neck 50 mL round-bottomed flask, added 2-chloropyrazine **S-11** (1.0 g, 8.73 mmol, 1 eq), in ethanol (10.0 mL) to hydrazine hydrate (4.3 mL, 87.3 mmol 10 eq), then stirred the reaction mixture at 60 °C for 16 h. After completion of the reaction monitored by TLC (90% Ethyl acetate in hexane as mobile phase), the reaction mixture cooled, solvents were evaporated under reduced pressure, and crude residue was extracted with ethyl acetate (50 mL x 5). The combined ethyl acetate layer was dried over MgSO4, filtered, and evaporated under reduced pressure. The crude product was purified by MPLC (0-100% ethyl acetate in hexane as mobile phase gradient) to get the desired compound **S-12** (750 mg, 77.7 %) as a yellow solid. ^{1}H NMR (400 MHz, DMSO-d_6) δ 8.08 (s, 1H), 7.91 (dd, *J* = 2.8, 1.5 Hz, 1H), 7.89 (s, 1H), 7.68 (d, *J* = 2.8 Hz, 1H), 4.20 (s, 2H).

Synthesis of S-13

In a 1-neck 50 mL round-bottomed flask, added compound **S-10** (150 mg, 0.328 mmol, 1 eq) in DMF (2 ml), was added **S-12** (43.5 mg, 0.395 mmol, 1.2 eq), stirred the reaction mixture for 5 mins at room temperature then EDC.HCl (94.6 mg, 0.493 mmol. 1.5 eq) was added further stirred the reaction mixture for 4 h at room temperature, after completion of the reaction monitored by TLC (10% MeOH in DCM as mobile phase), ice water was added, and extracted with EtOAc (30 ml X 3) washed with aq.$NaHCO_3$ and purified by RP-MPLC (0-100% Acetonitrile in water as mobile phase) to afford the desired product **S-13** (120 mg, 66.6%) as pale yellow solid. ^{1}H NMR (400 MHz, Chloroform-*d*) δ 10.37 (s, 1H), 9.58 (s, 1H), 8.55 (s, 1H), 8.17 (s, 1H), 7.97 (d, *J* = 13.2 Hz, 2H), 7.84 (s, 1H), 7.66 (s, 1H), 7.07 (s, 2H), 5.73 (d, *J* = 11.0 Hz, 1H), 5.00 (s, 1H), 3.99 (t, *J* = 5.0 Hz, 2H), 3.39 – 3.18 (m, 2H), 1.92 (s, 2H), 1.41 (s, 9H); ^{13}C NMR (100 MHz, Chloroform-*d*) δ 163.1, 161.1, 159.1, 156.2, 154.2, 148.1, 141.3, 136.0, 132.2, 131.5, 130.6, 124.9, 122.2, 115.8, 115.2, 113.3, 107.7, 79.4, 66.0, 37.6, 29.4, 28.3.

Synthesis of SEL-TCO

To a solution of **S-13** (25 mg, 0.0456 mmol, 1 eq) in DCM (10 ml) was added TFA (0.3 ml) at 0 °C, and stirred the solution at room temperature for 2h, after completion of the reaction monitored by TLC (10% MeOH in DCM as mobile phase), solvents were evaporated under

reduced pressure obtained crude product was further used without purification by dissolving it in DMF (1 ml), to this DIPEA (80 µL, 0.456 mmol, 10 eq) and TCO-NHS ester (12.2 mg, 0.0456 mmol, 1 eq) were added and stirred the reaction mixture for 12 h at room temperature, after completion of the reaction monitored by TLC, ice water was added and extracted with EtOAc (10 ml X 3) washed with aq.$NaHCO_3$, and purified by RP-HPLC (0-100% Acetonitrile in water as mobile phase) to afford the desired product **SEL-TCO** (10.98%) as an off-white solid. ^{1}H NMR (400 MHz, DMSO-d_6) δ 10.50 (d, J = 1.9 Hz, 1H), 9.53 (s, 1H), 9.12 (d, J = 1.9 Hz, 1H), 8.11 (d, J = 1.4 Hz, 1H), 8.06 (dd, J = 2.7, 1.5 Hz, 1H), 7.92 (d, J = 2.7 Hz, 1H), 7.83 (d, J = 4.7 Hz, 1H), 7.80 – 7.73 (m, 1H), 7.48 (d, J = 10.5 Hz, 1H), 7.35 – 7.28 (m, 1H), 7.22 (t, J = 5.7 Hz, 1H), 6.02 (d, J = 10.6 Hz, 1H), 5.65 – 5.44 (m, 2H), 4.69 (dd, J = 11.1, 5.5 Hz, 1H), 4.14 (t, J = 6.3 Hz, 2H), 3.17 (q, J = 8.5, 7.8 Hz, 2H), 2.27 – 2.08 (m, 3H), 2.00 – 1.83 (m, 3H), 1.80 – 1.55 (m, 4H), 1.46 – 1.37 (m, 1H), 1.18 – 1.09 (m, 1H); ^{13}C NMR (100 MHz, DMSO-d_6) δ 163.6, 160.2, 159.9, 156.2, 155.3, 148.2, 142.0, 135.4, 134.9, 132.6, 131.9, 131.8, 131.3, 131.0, 125.5, 115.9, 114.8, 111.0, 79.6, 68.7, 66.4, 40.8, 37.4, 34.3, 32.5, 30.0, 29.4, 27.8. ESI-MS *m/z*: calc for $C_{28}H_{32}F_3N_8O_4$, $[M+H]^+$: 601.25, found 601.3

Synthesis of TPP-TCO

Scheme S6 Synthesis of TPP-TCO

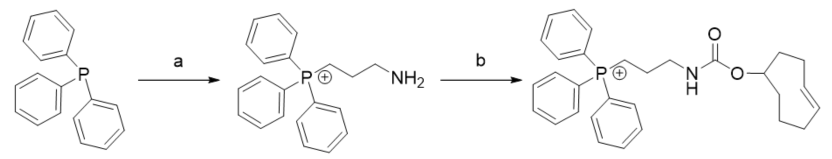

TP-1 TP-2 TP-3

Reagents and conditions: (a) 3-bromopropylamine hydrobromide, acetonitrile, reflux, 24 h. (b) TEA, DMF, r.t., 15 min.

Synthesis of TP-2

A solution of 3-bromopropylamine hydrobromide (1 g, 4.57 mmol, 1.4 eq) and triphenylphosphine (836 mg, 3.19 mmol, 1 eq) in acetonitrile were stirred at reflux temperature for 24 h. The precipitate was filtered and washed with dichloromethane to obtain the title

compound as a white solid. (900 mg, 70.3%) ^{1}H NMR (600 MHz, DMSO-d_6) δ 7.99-7.88 (t, *J = 6.5 Hz*, 3H), 7.86-7.78 (m, 12H), 3.81-3.68 (dt, *J = 15.1, 7.2 Hz*, 2H), 3.04-2.93 (t, *J = 7.2 Hz*, 2H), 1.90-1.77 (m, 2H); ESI-MS *m/z*: calc. for $C_{21}H_{23}NP^{+}$, $[M]^{+}$: 320.16, found 320.2.

Synthesis of TP-3

A suspension of **TP-2** (37.4 mg, 0.094 mmol, 1 eq), TCO-NHS ester (50 mg, 0.187 mmol, 2 eq), and triethylamine (130 μL, 0.935 mmol, 9.9 eq) in DMF (400 μL) was stirred at room temperature for 15 min. The crude product was purified by reverse-phase preparative HPLC using a linear gradient of acetonitrile (5−100%) for 25 min in water with 0.1% TFA to afford title compound as transparent oil. (4 mg, 7.7%) ^{1}H NMR (600 MHz, CD_3OD) δ 7.96-7.70 (m, 15H), 5.69-5.48 (m, 2H), 4.87-4.75 (q, *J = 5.3 Hz*, 1H), 3.48-3.35 (m, 2H), 2.74-1.06 (m, 14H); ESI-MS *m/z*: calc for $C_{30}H_{35}NO_2P^{+}$, $[M]^{+}$: 472.24, found 472.4.

^{1}H and ^{13}C NMR Spectra

^{1}H NMR of **TA-2**

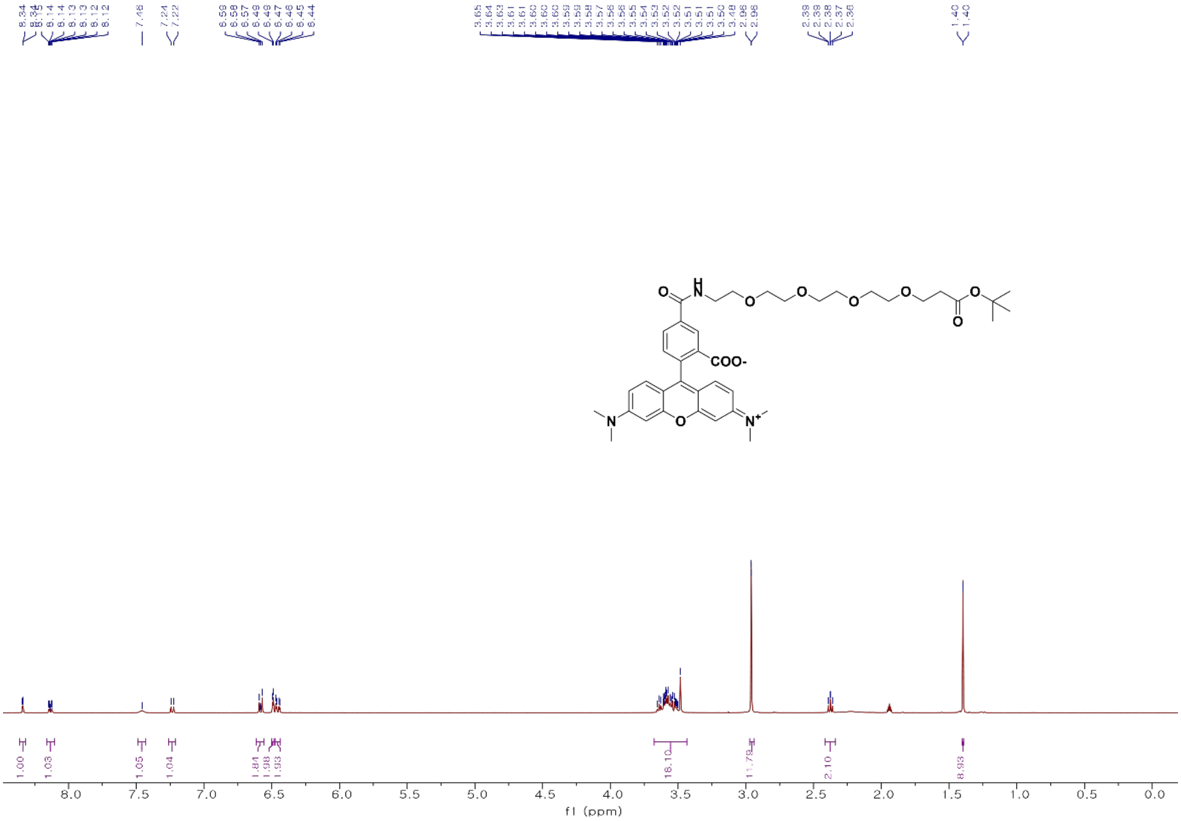

^{13}C NMR of **TA-2**

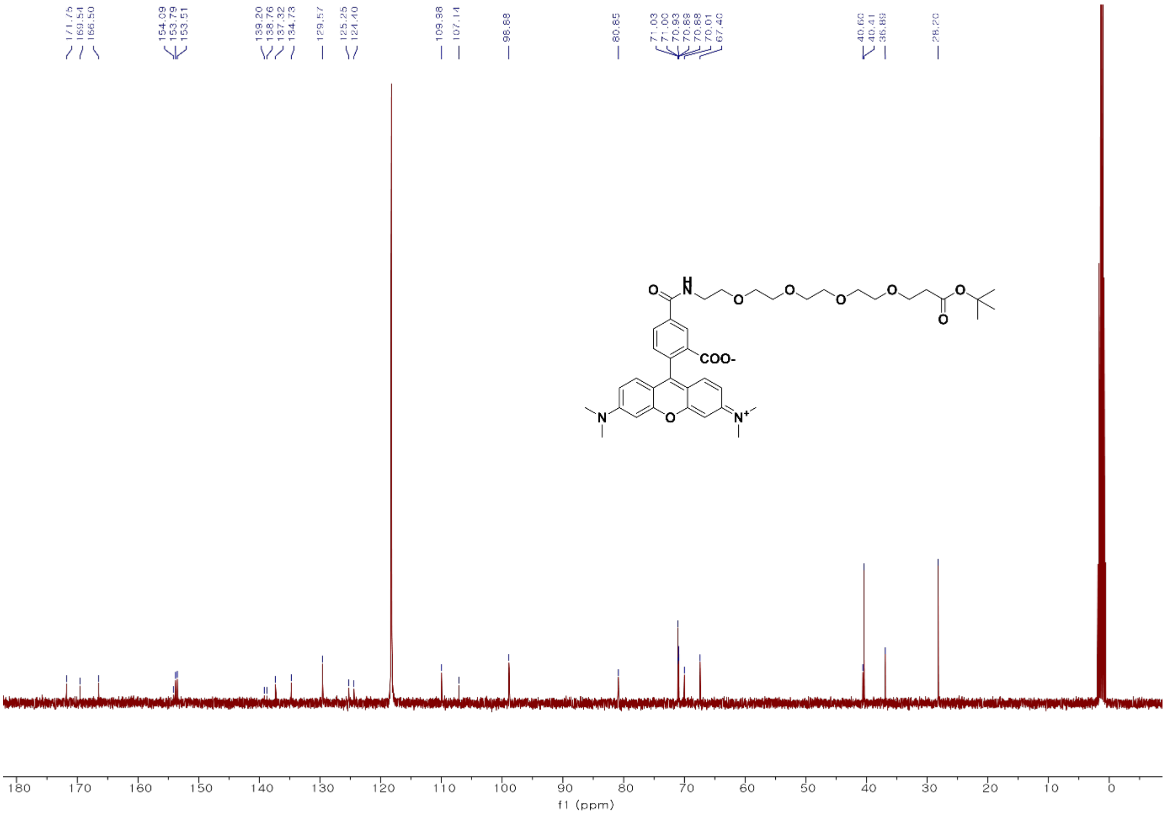

^{1}H NMR of **TAMRA-Bn**

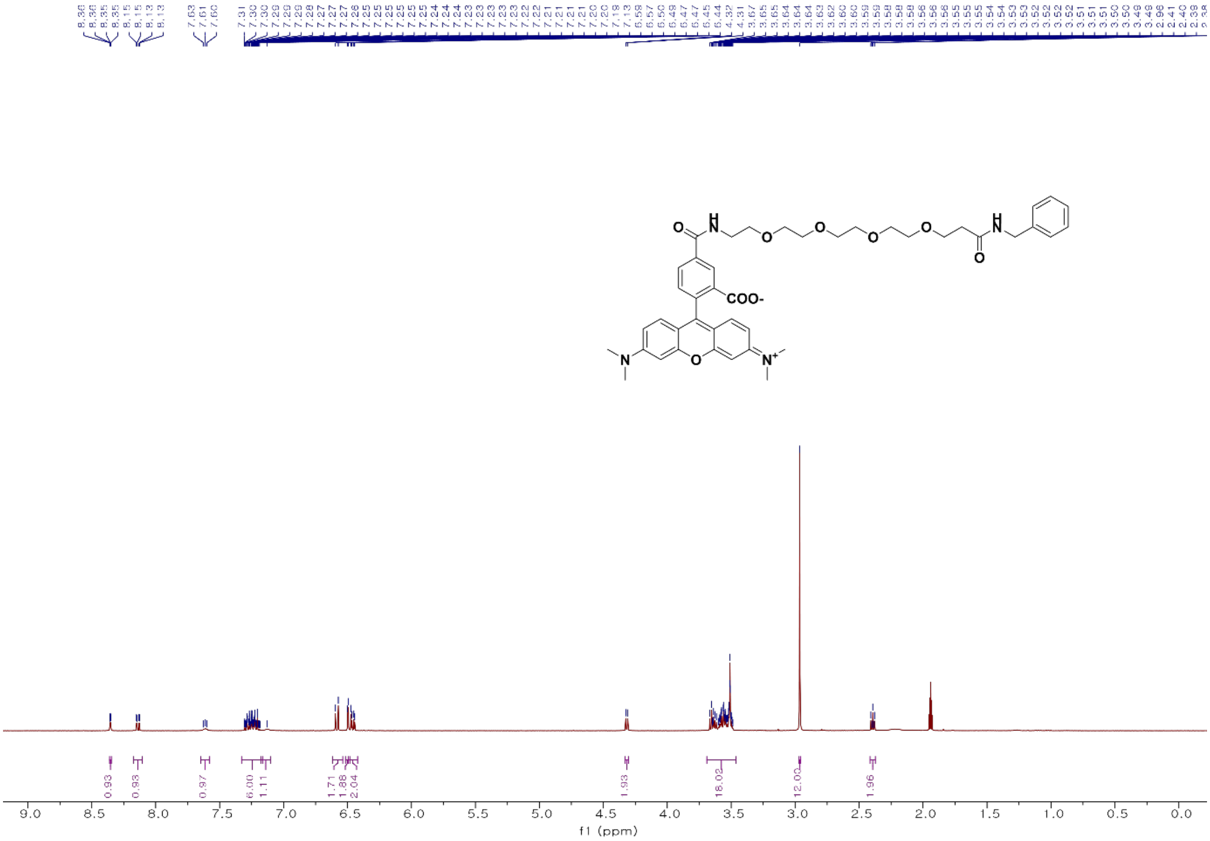

^{13}C NMR of **TAMRA-Bn**

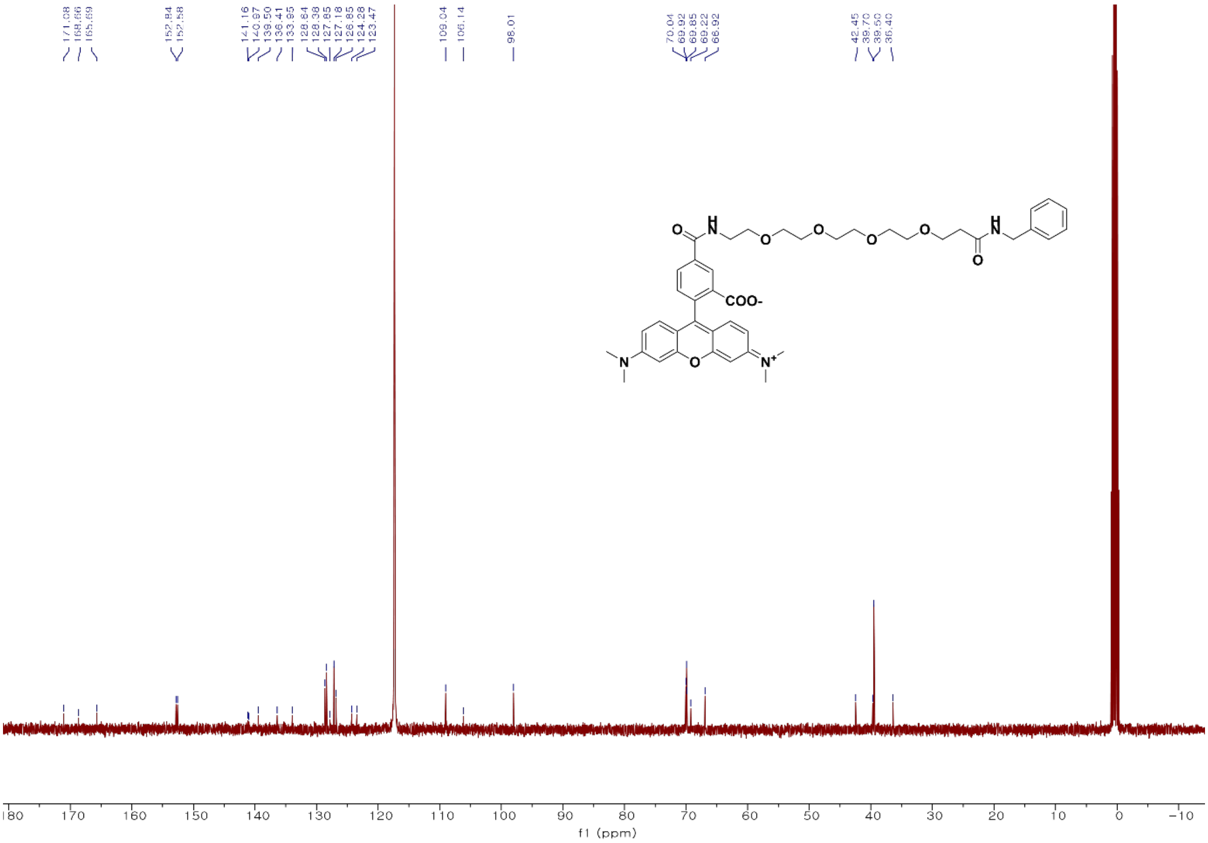

ESI-MS of **TAMRA-Bn**

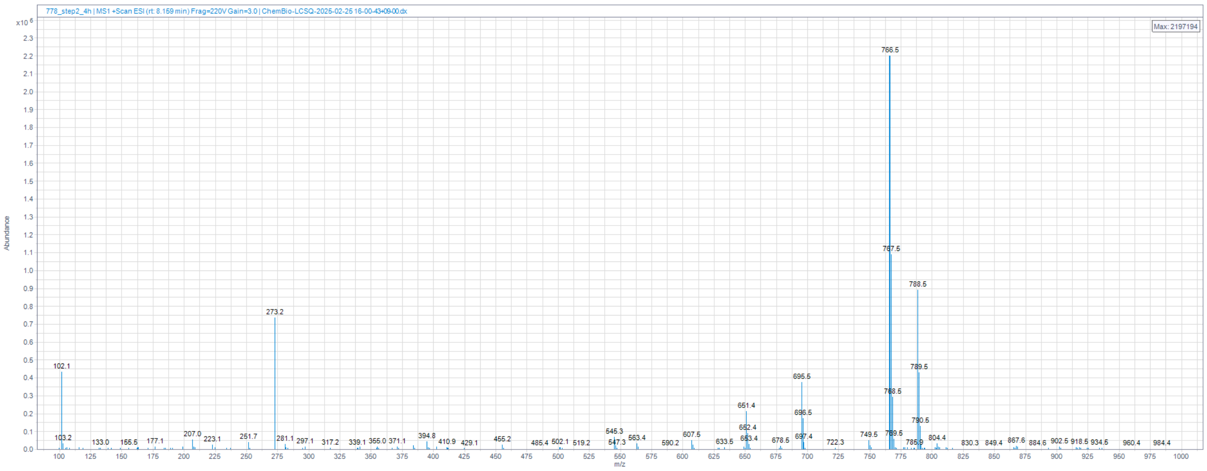

ESI-MS of **Cy5-Bn**

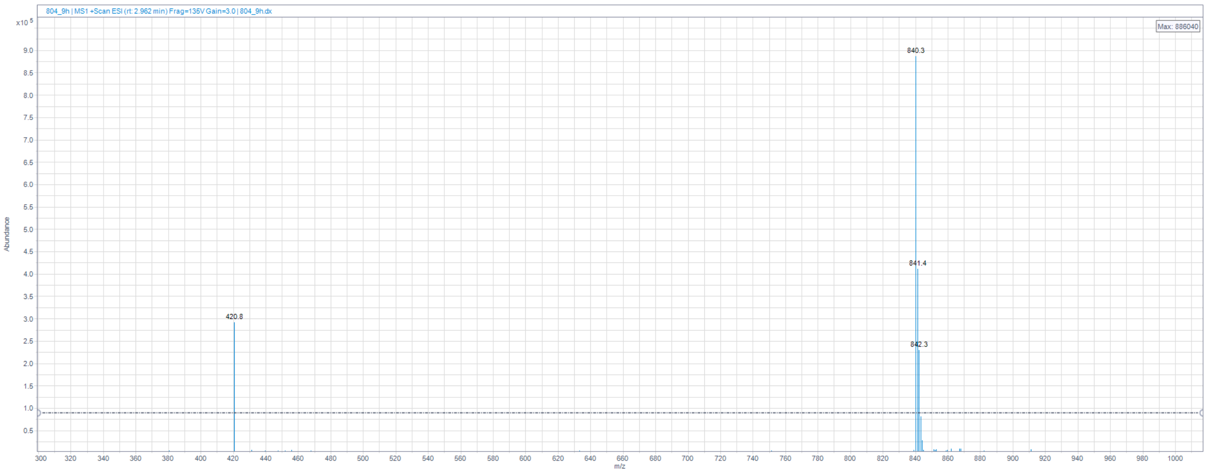

^{1}H NMR of deprotected **Boc-Tz9**

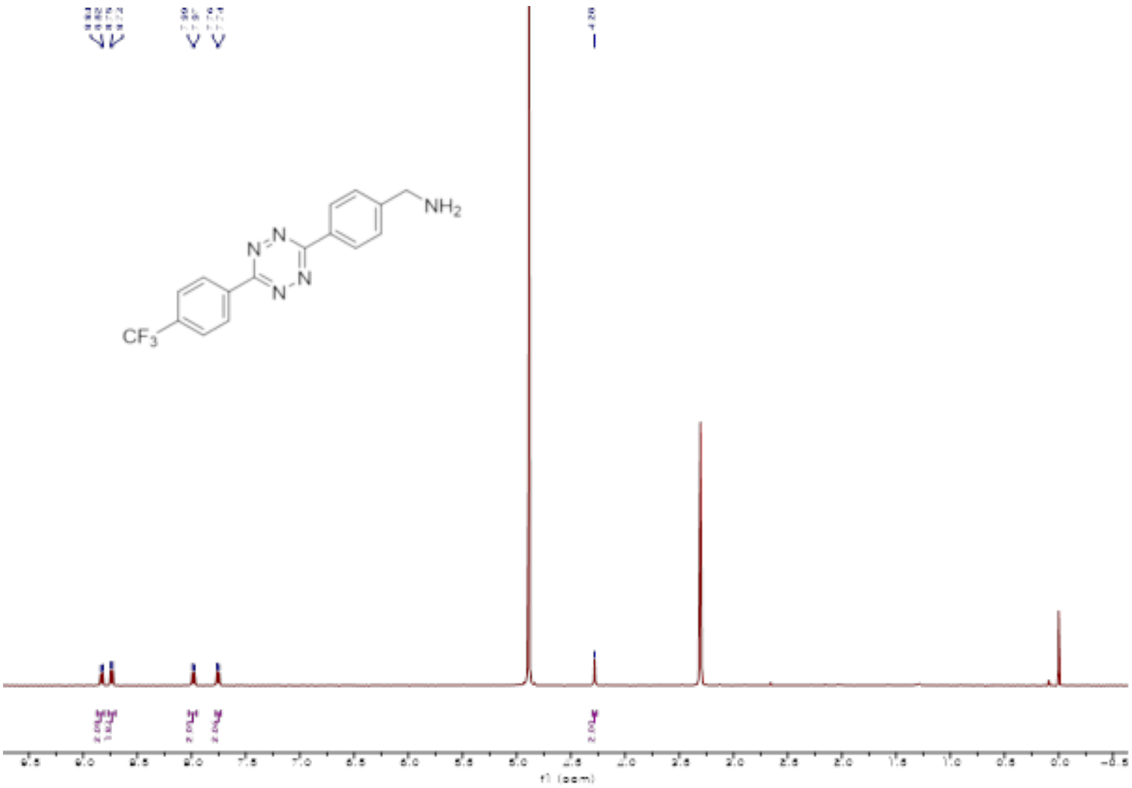

^{13}C NMR of deprotected **Boc-Tz9**

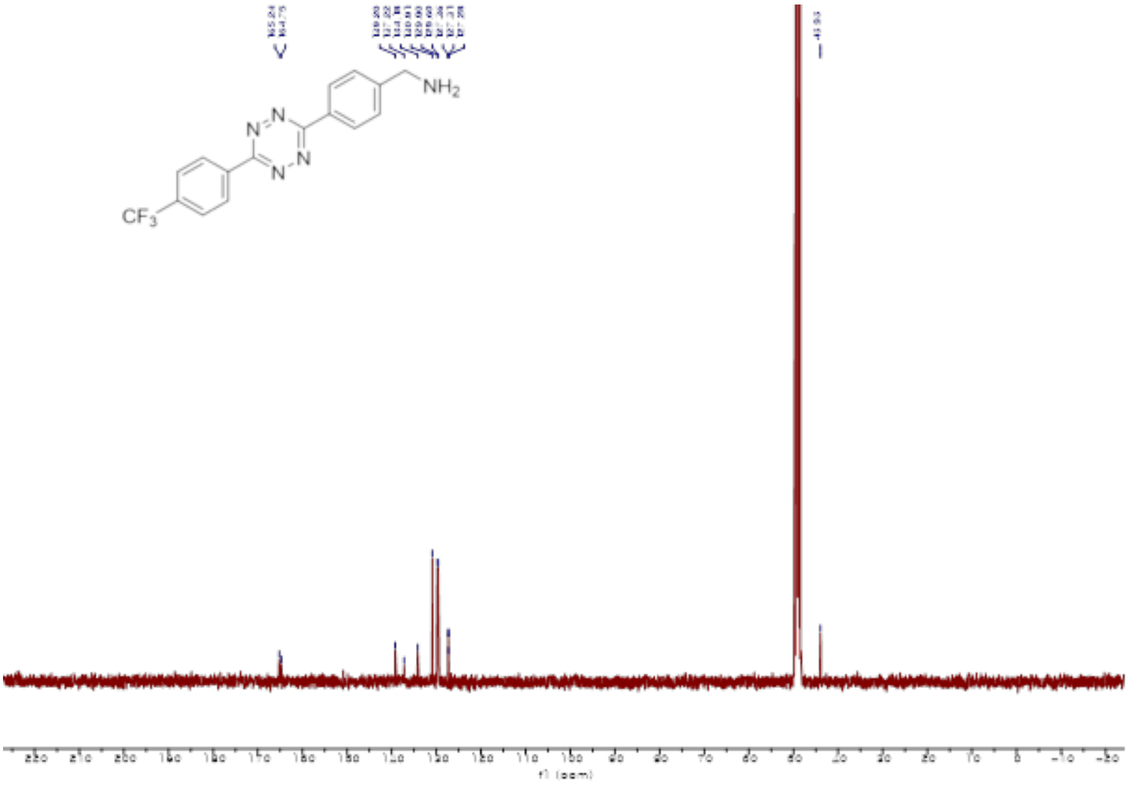

^{1}H NMR of B**oc-Tz10**

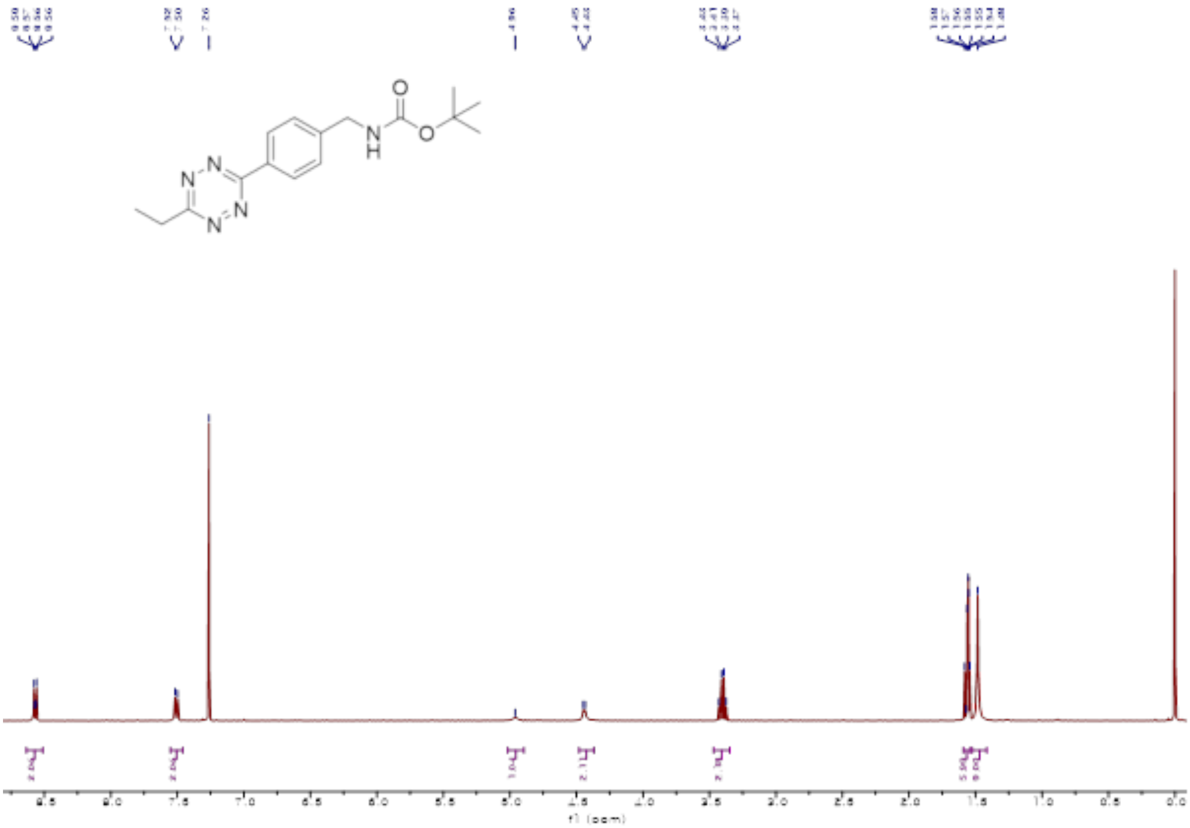

^{13}C NMR of **Boc-Tz10**

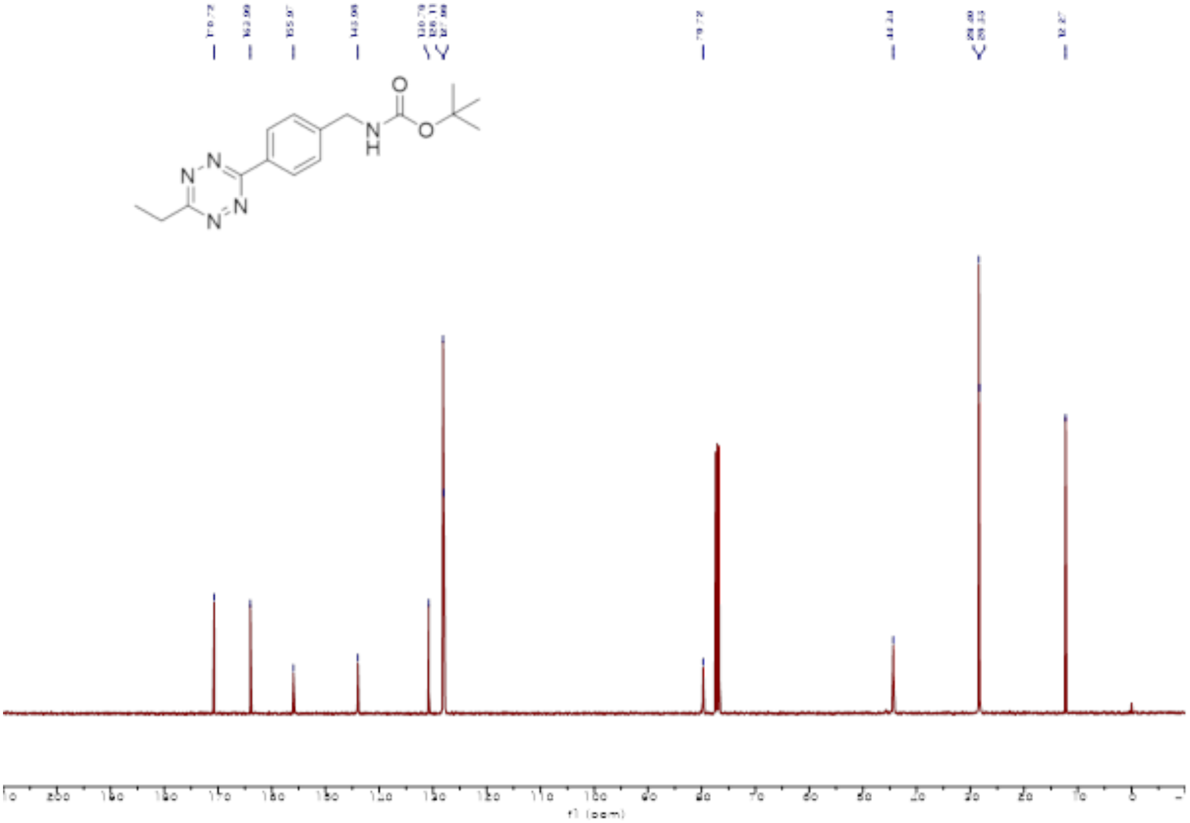

^{1}H NMR of B**oc-Tz11**

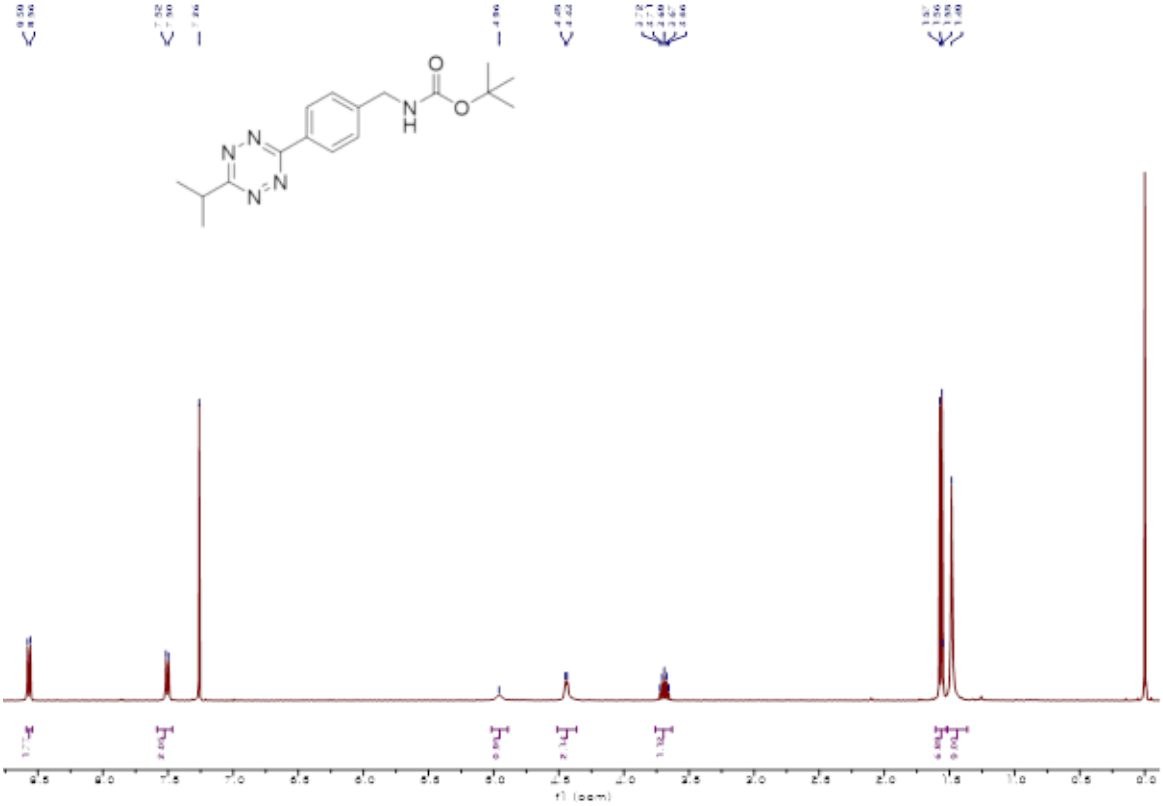

^{13}C NMR of **Boc-Tz11**

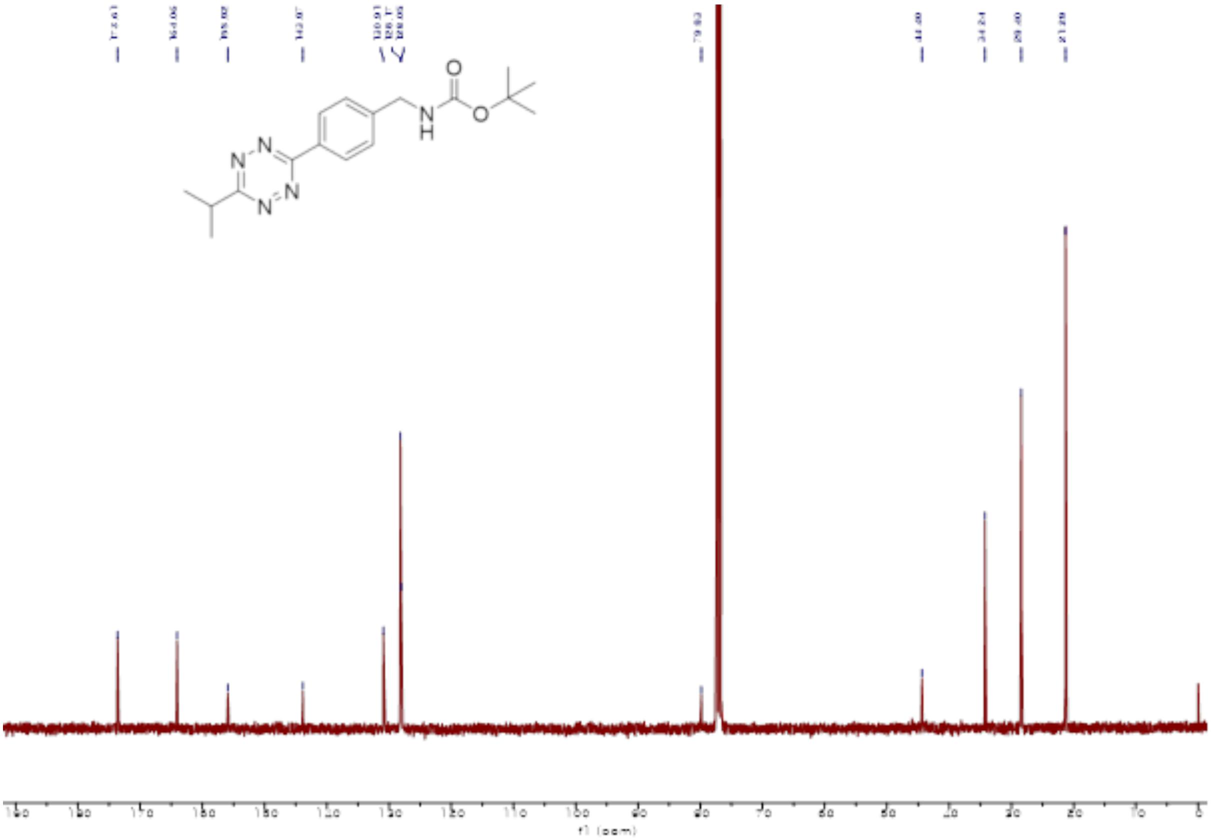

^{1}H NMR of Boc-Tz12

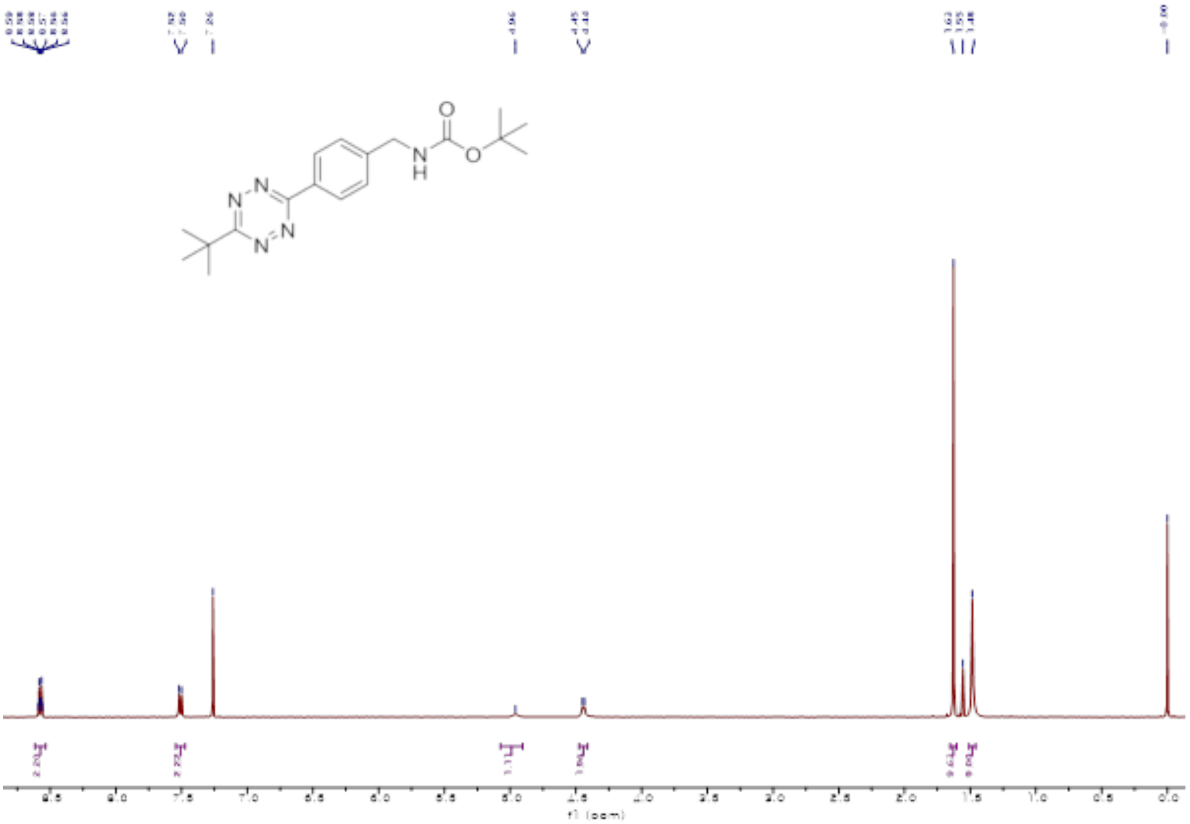

^{13}C NMR of Boc-Tz12

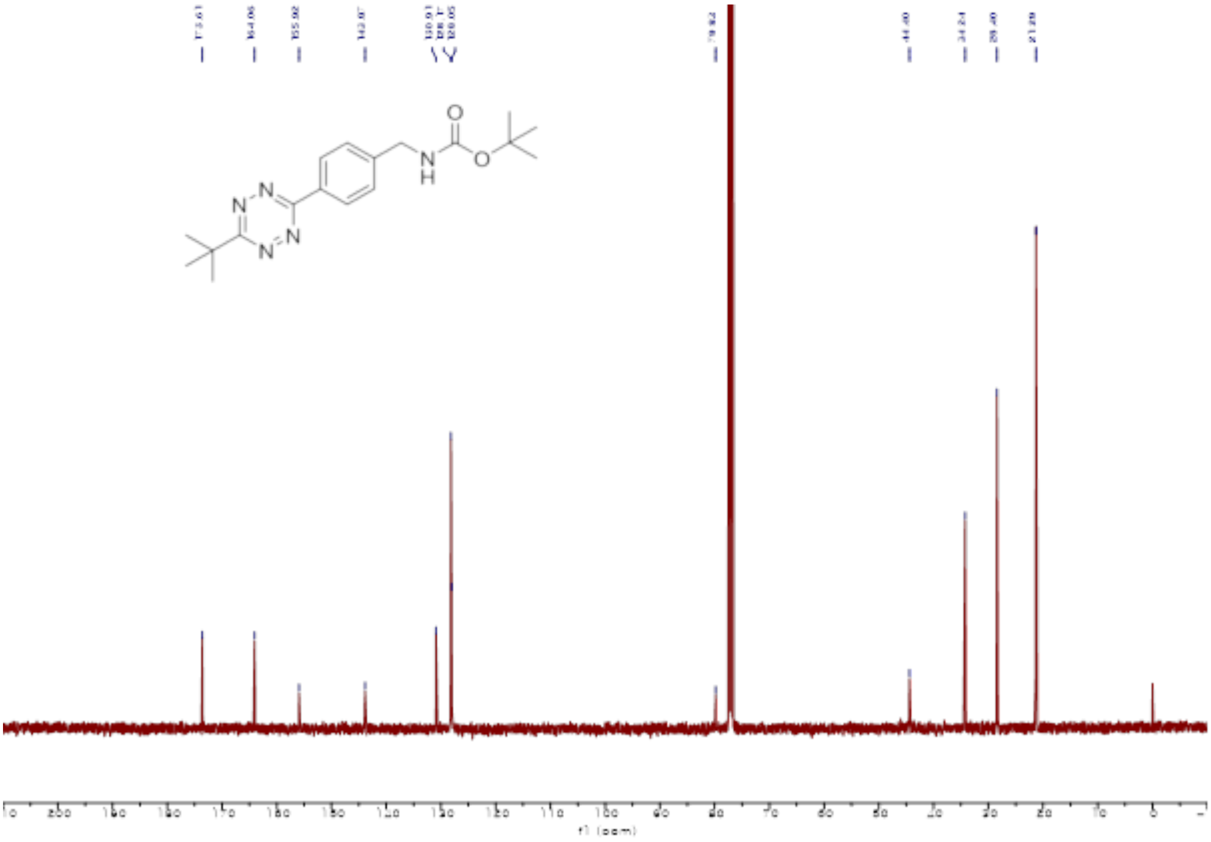

^{1}H NMR of B**oc-Tz13**

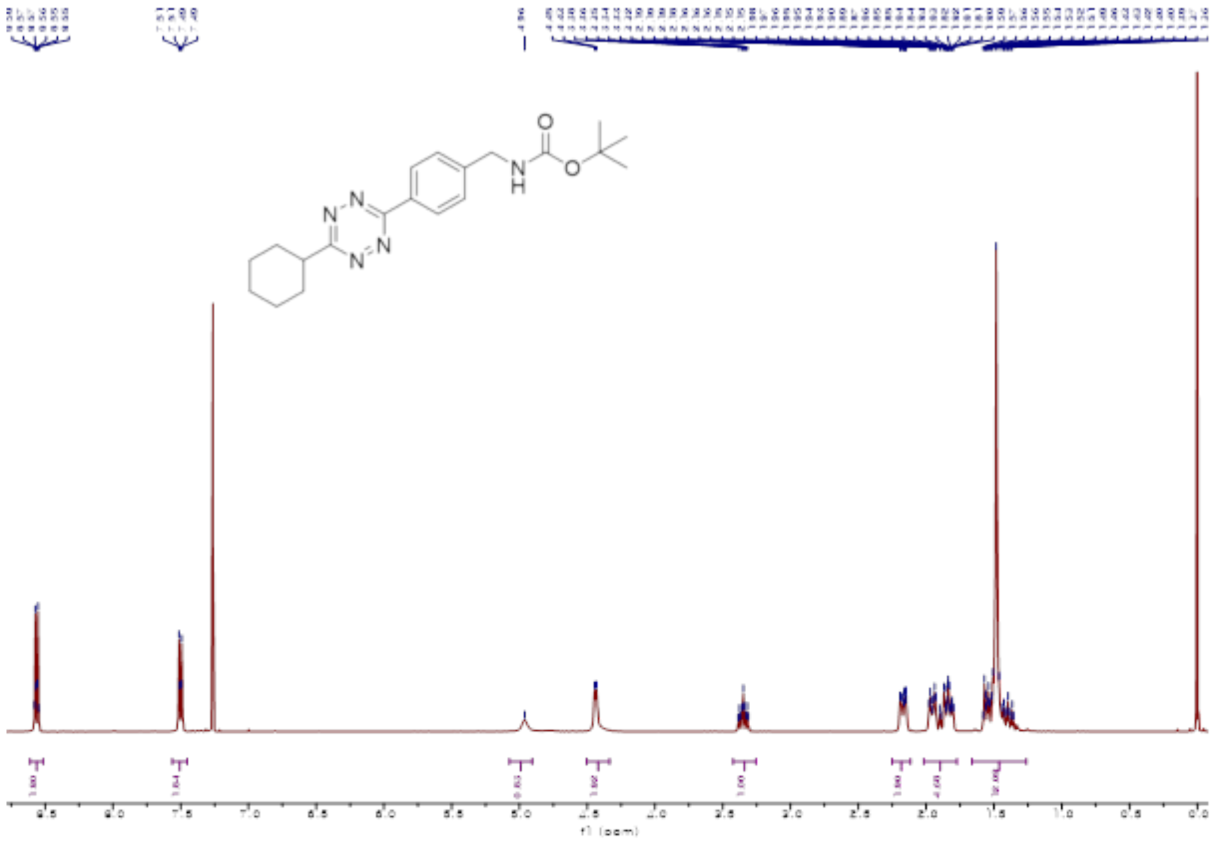

^{13}C NMR of **Boc-Tz13**

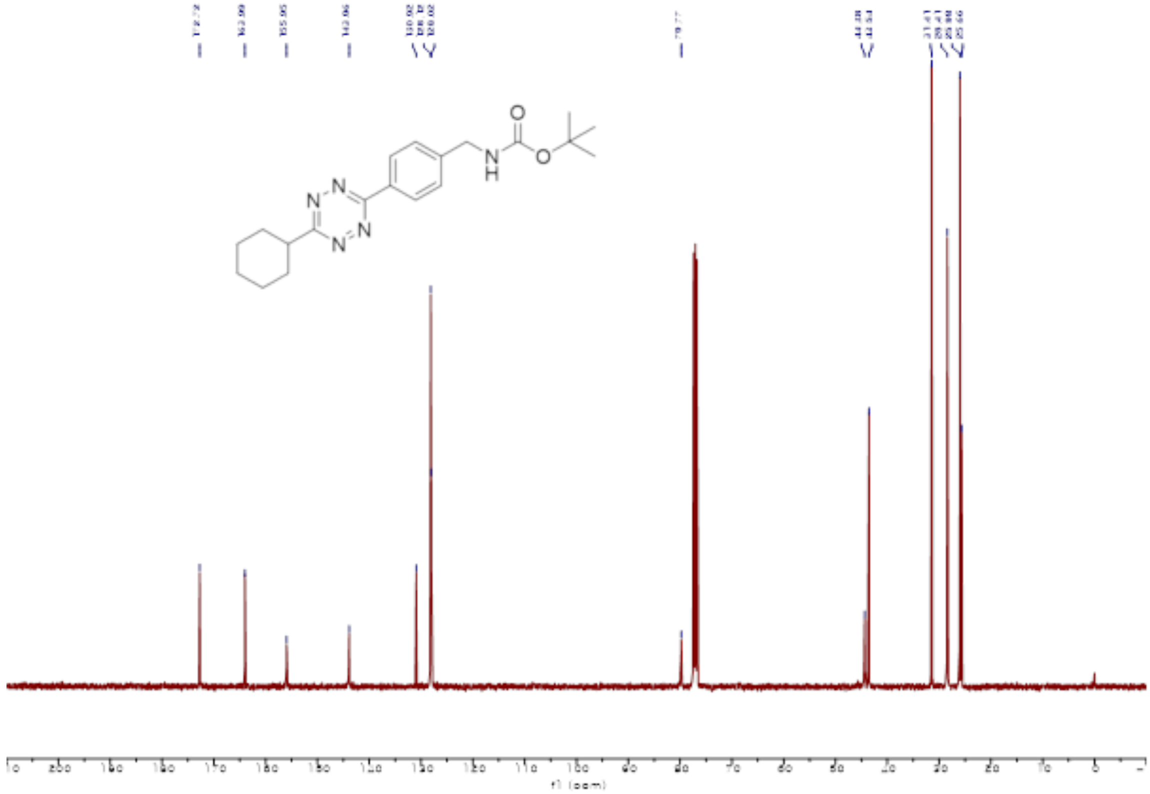

^{1}H NMR of B**oc-Tz14**

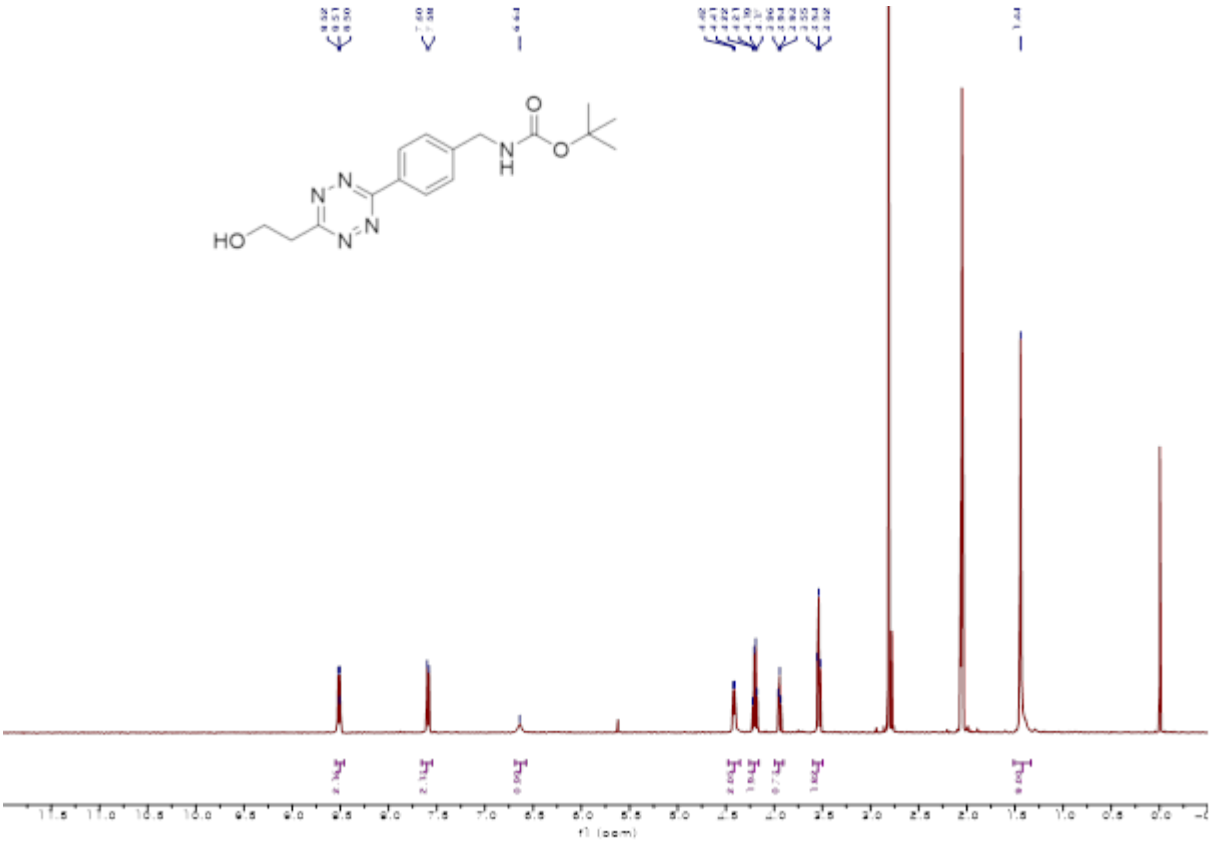

^{13}C NMR of **Boc-Tz14**

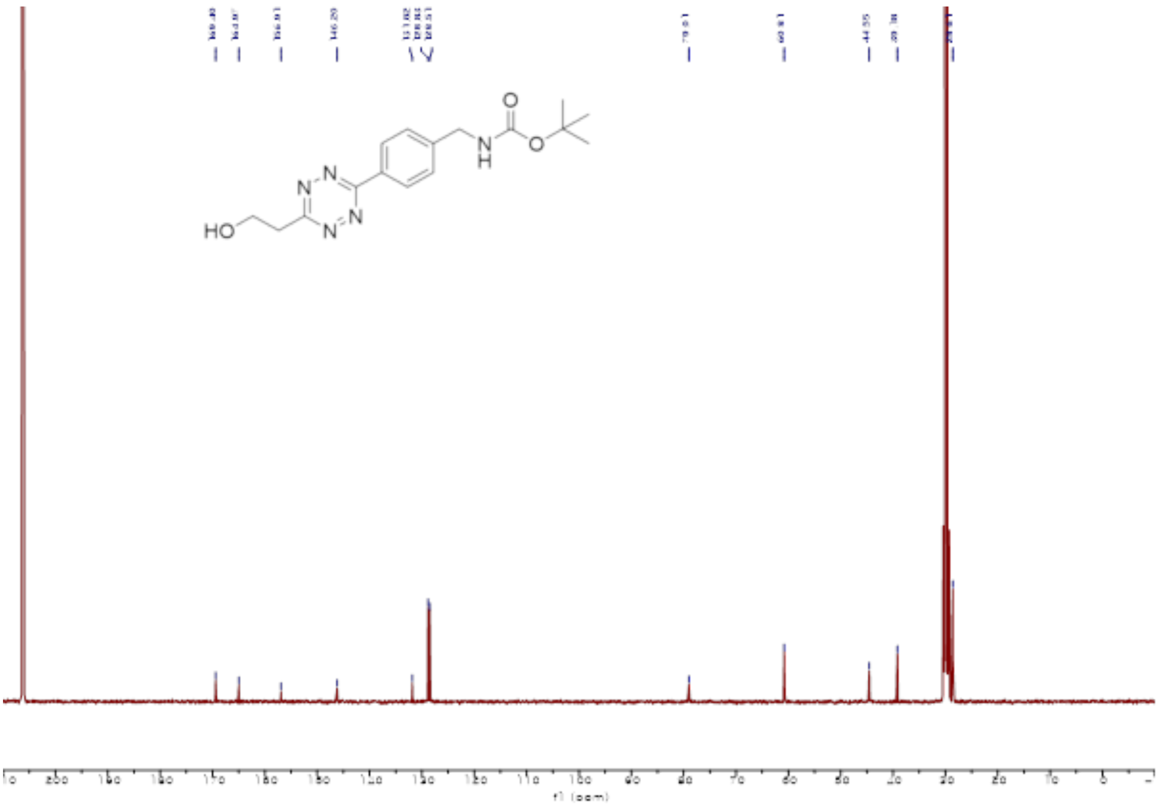

^{1}H NMR of B**oc-Tz15**

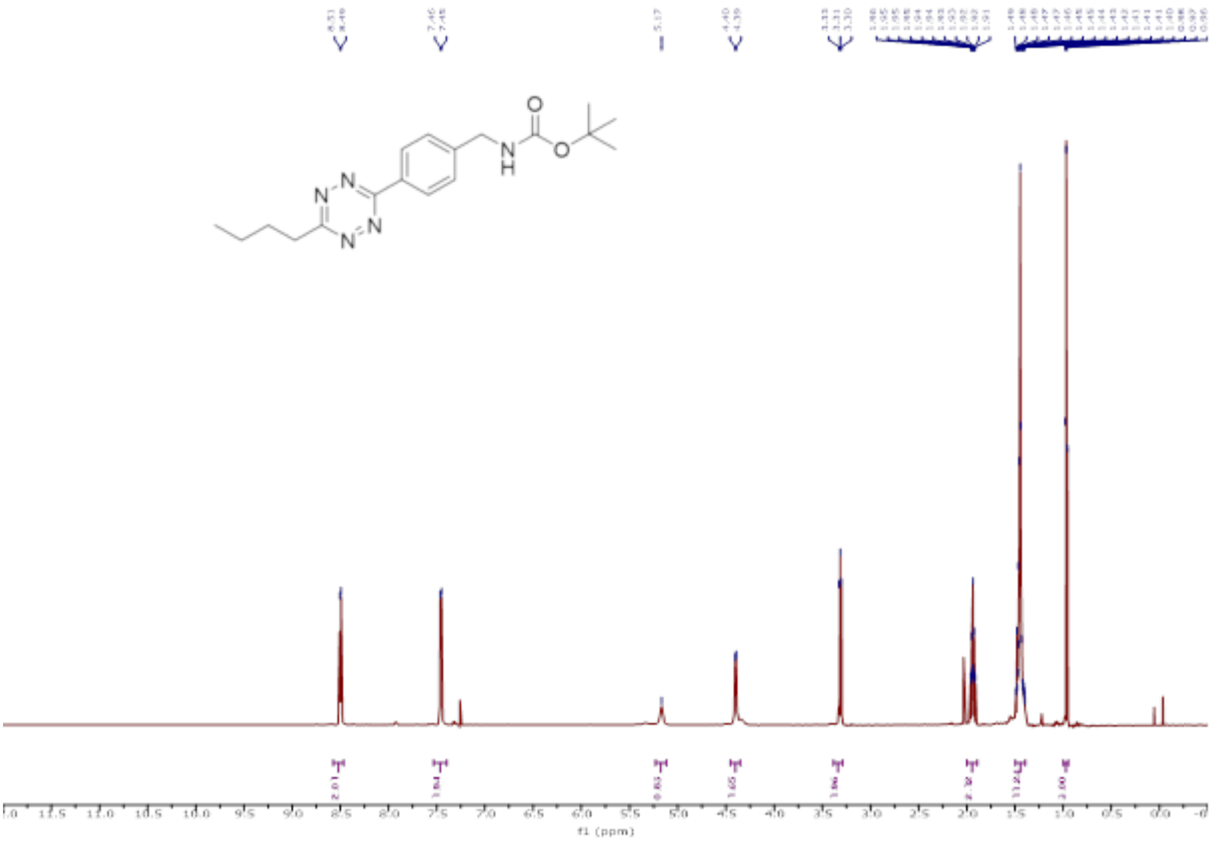

^{13}C NMR of **Boc-Tz15**

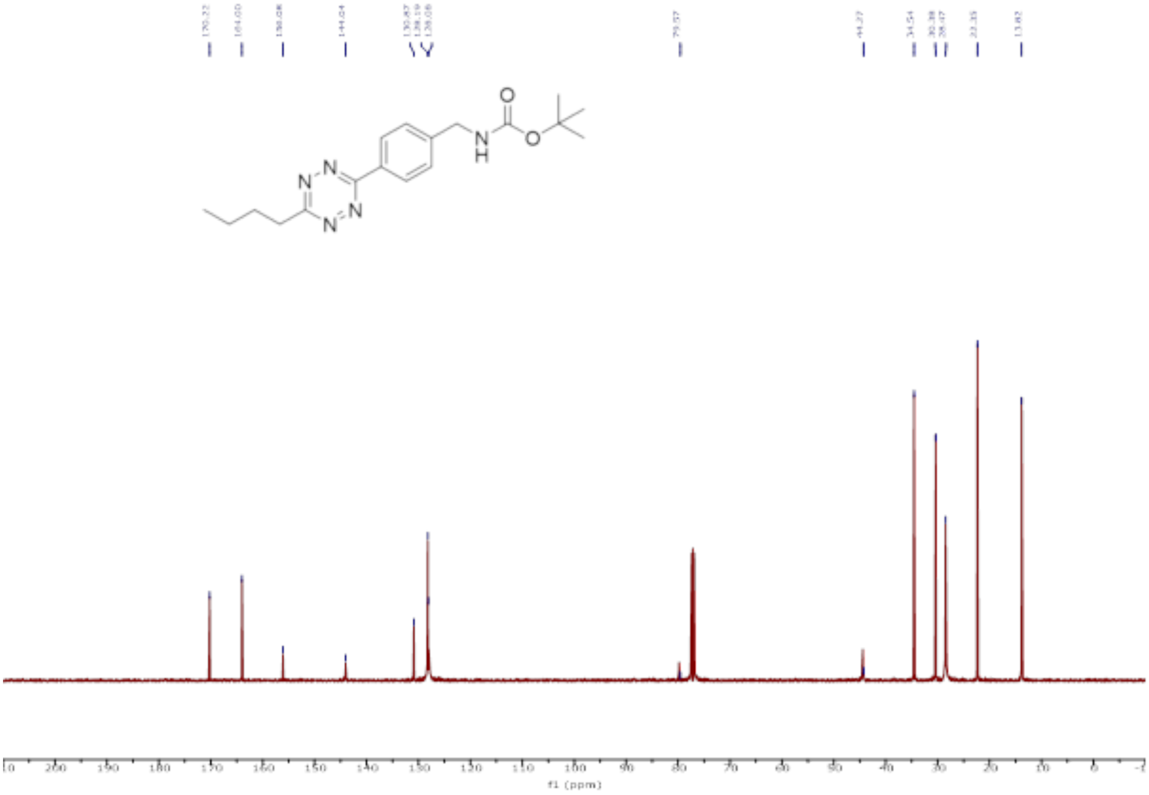

^{1}H NMR of Boc-Tz16

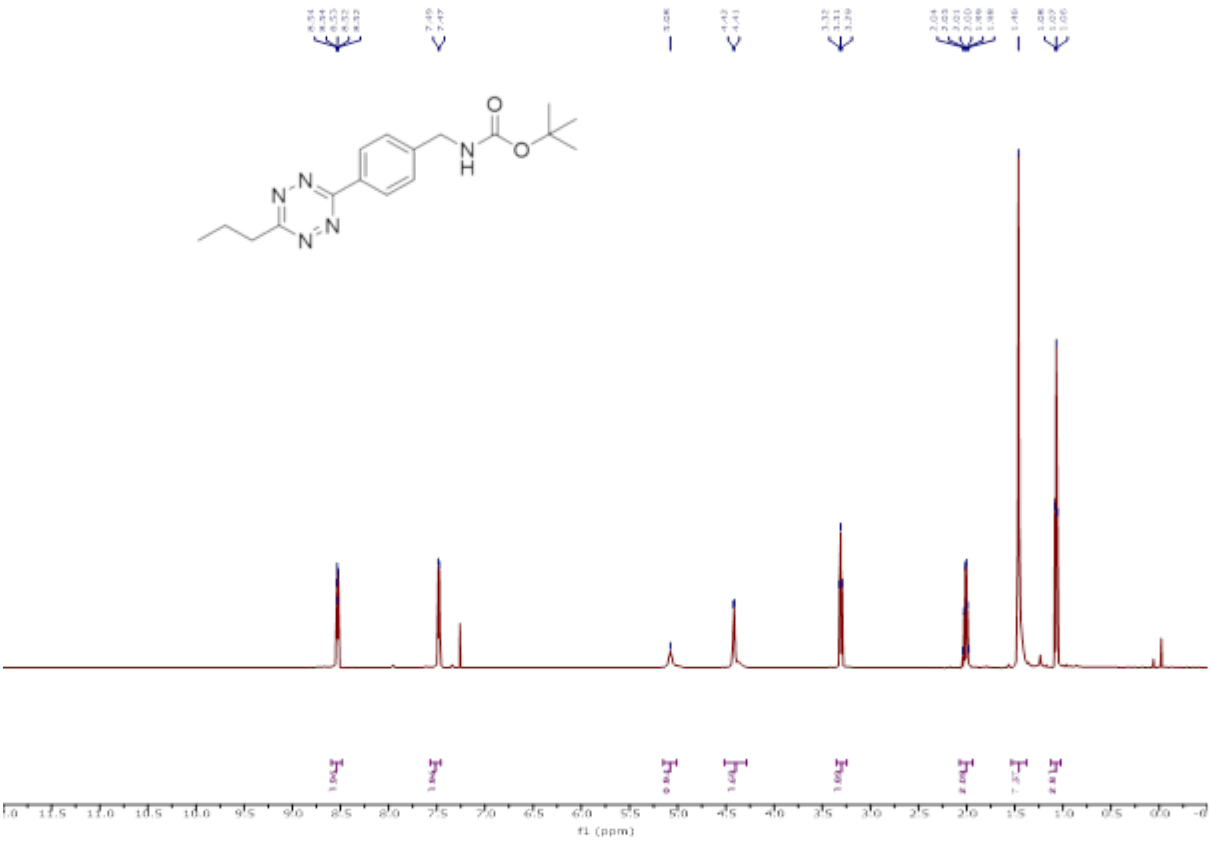

^{13}C NMR of **Boc-Tz16**

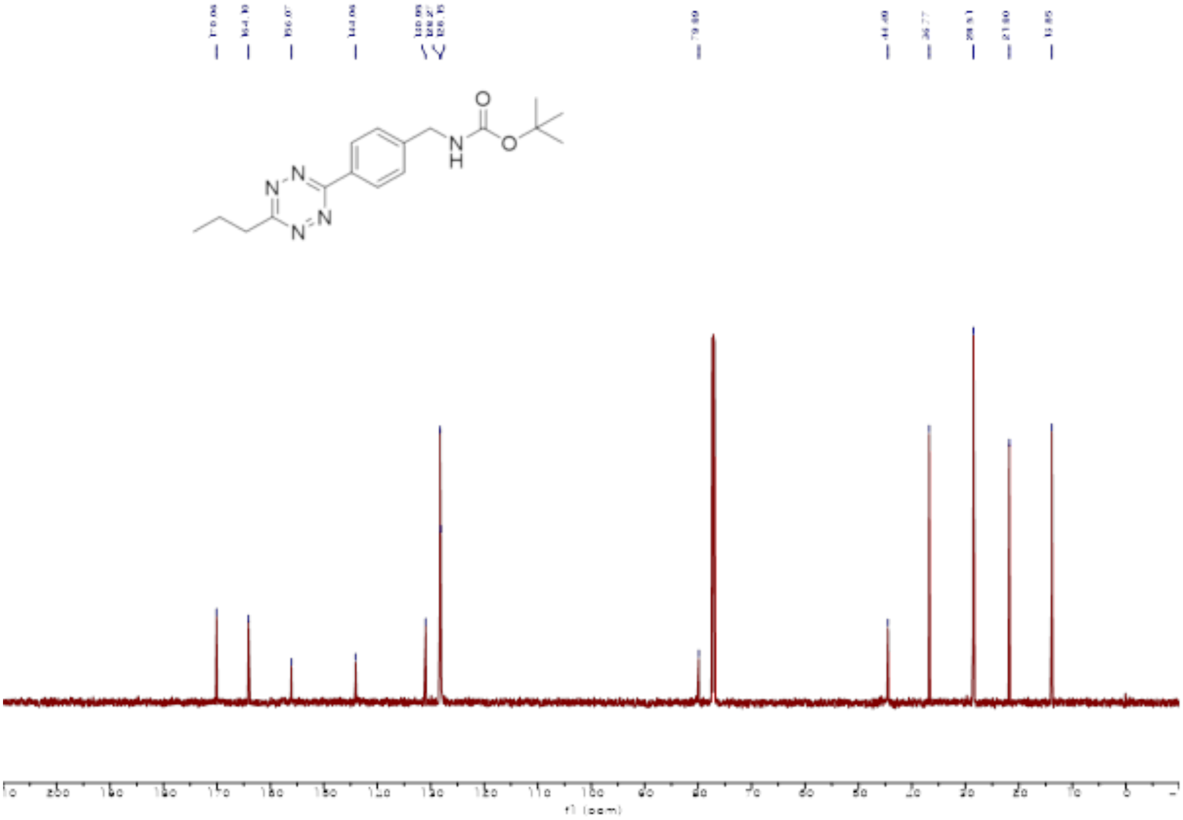

^{1}H NMR of B**oc-Tz17**

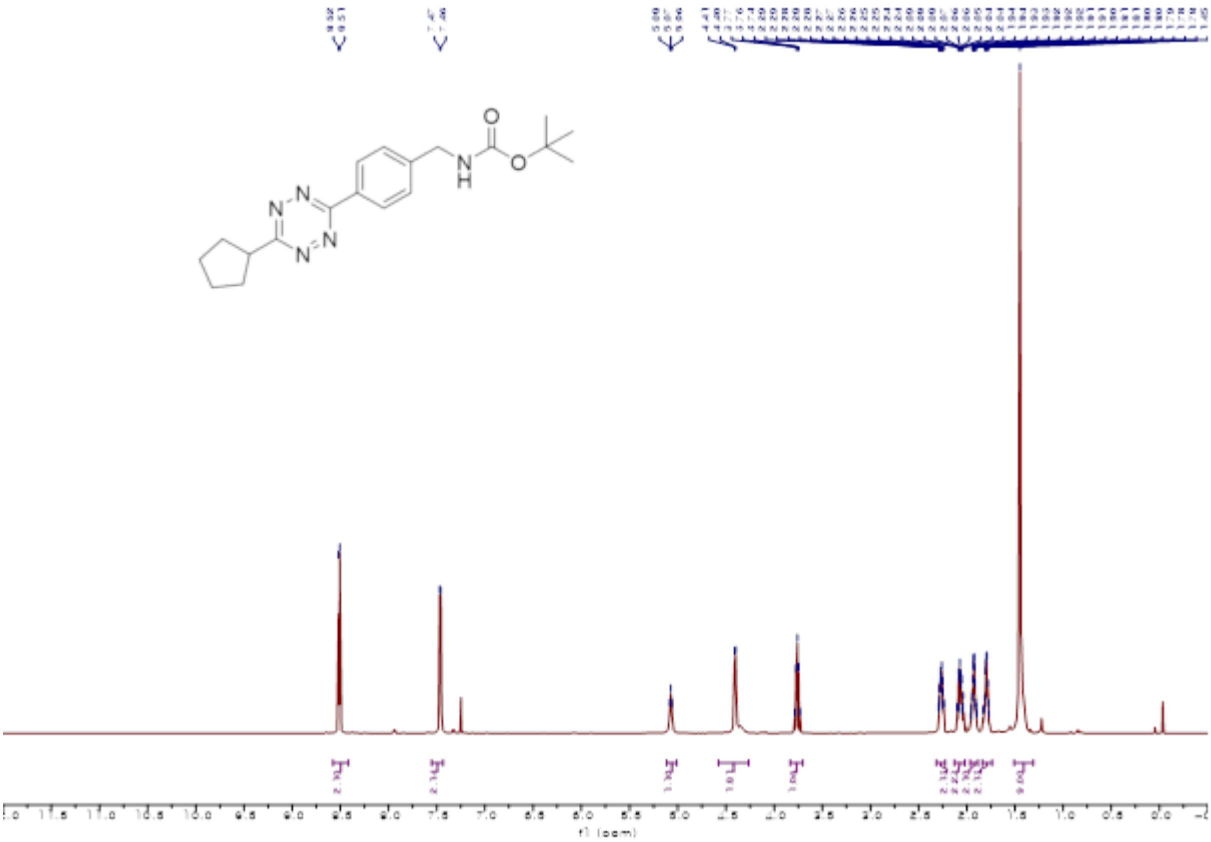

^{13}C NMR of **Boc-Tz17**

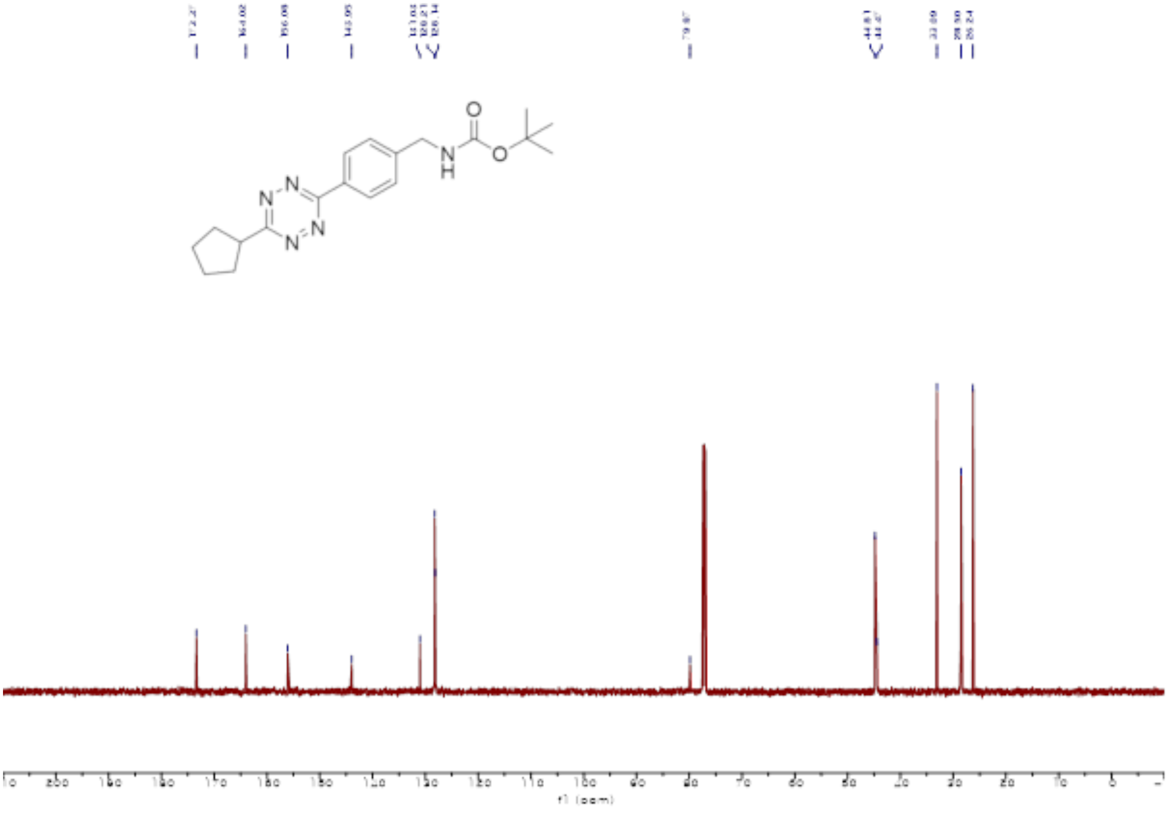

^{1}H NMR of B**oc-Tz18**

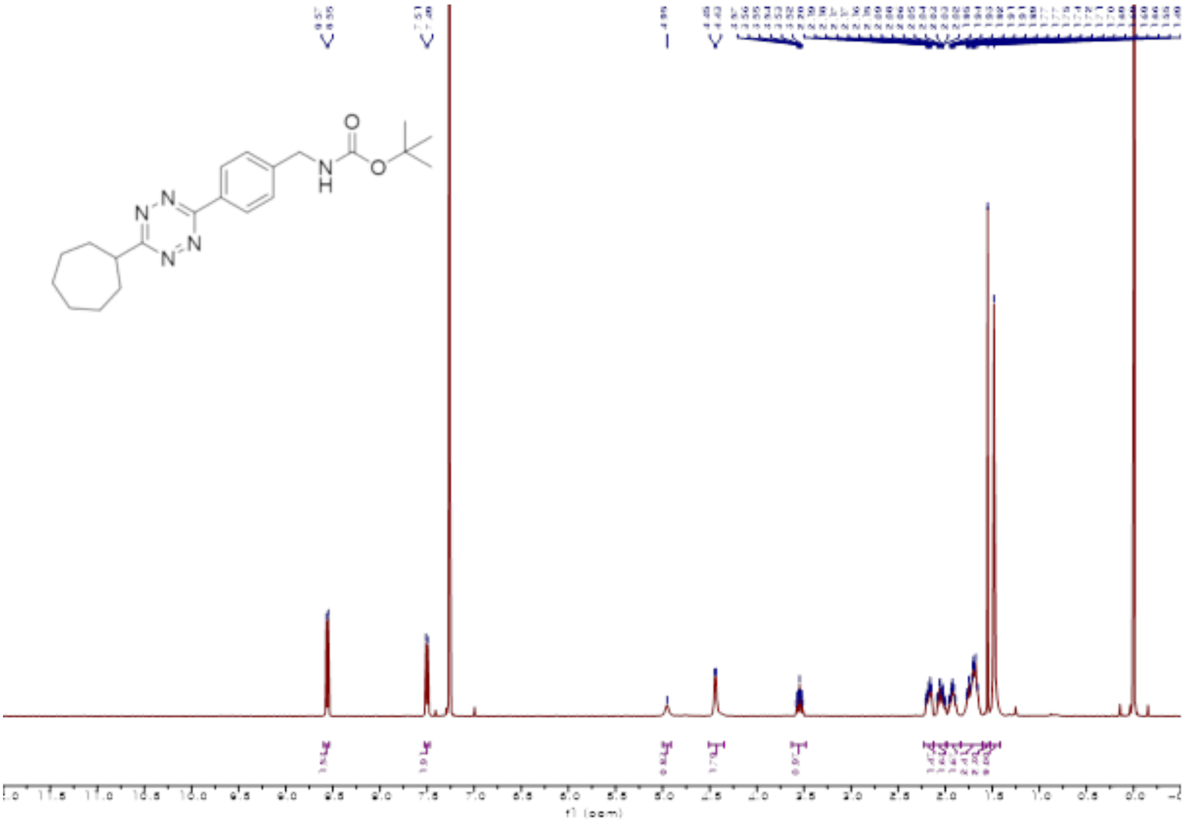

^{13}C NMR of **Boc-Tz18**

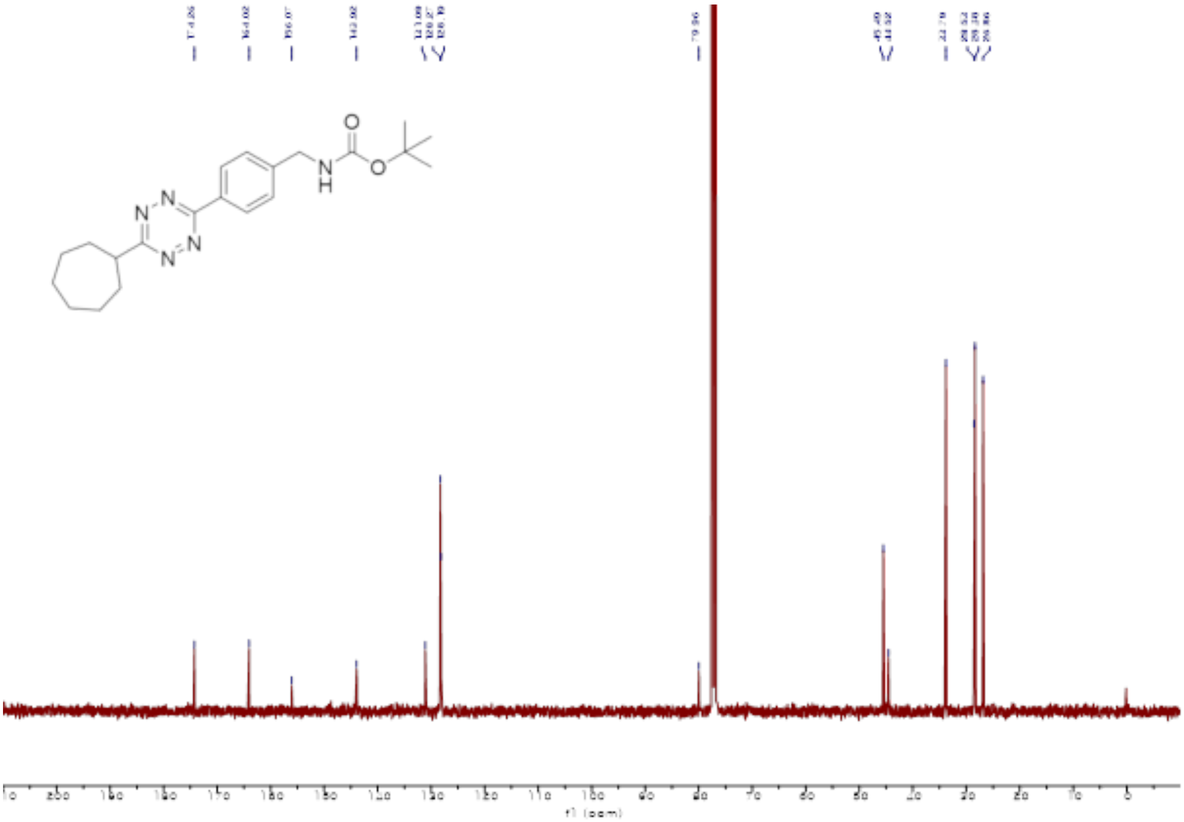

^{1}H NMR of B**oc-Tz19**

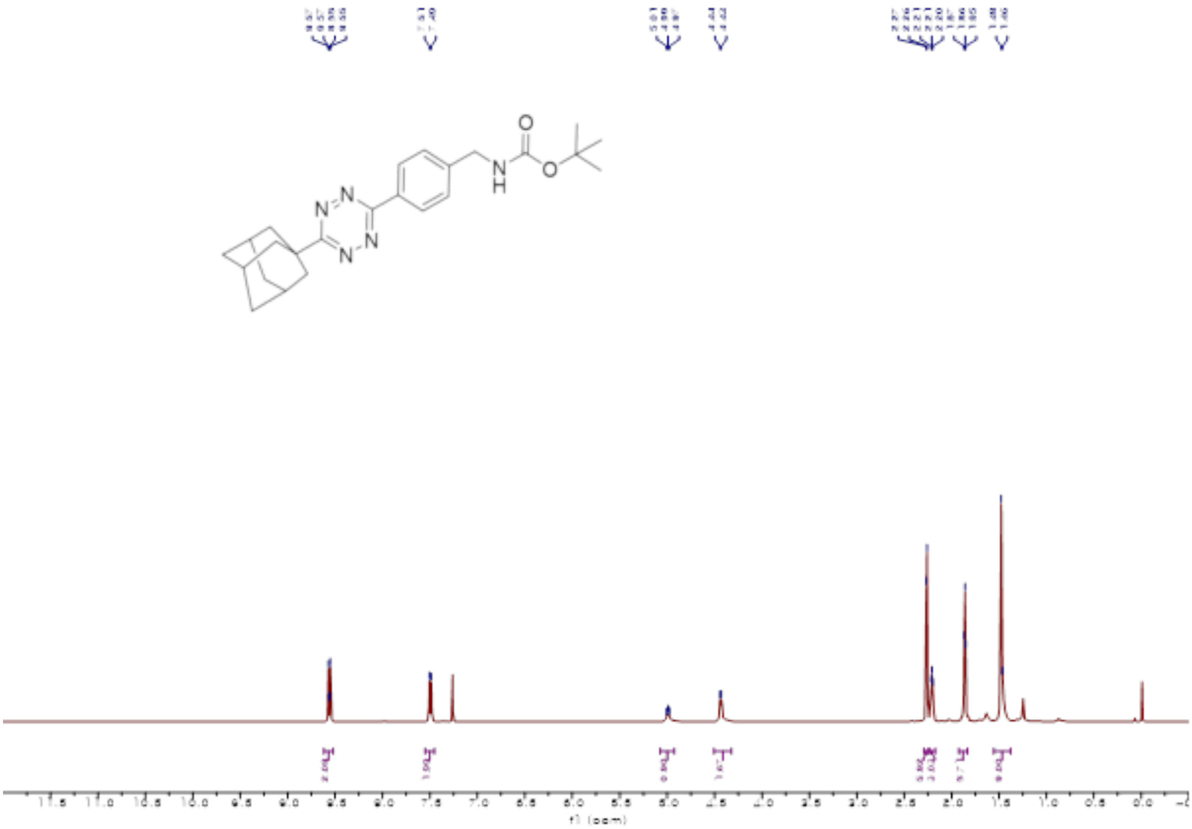

^{13}C NMR of **Boc-Tz19**

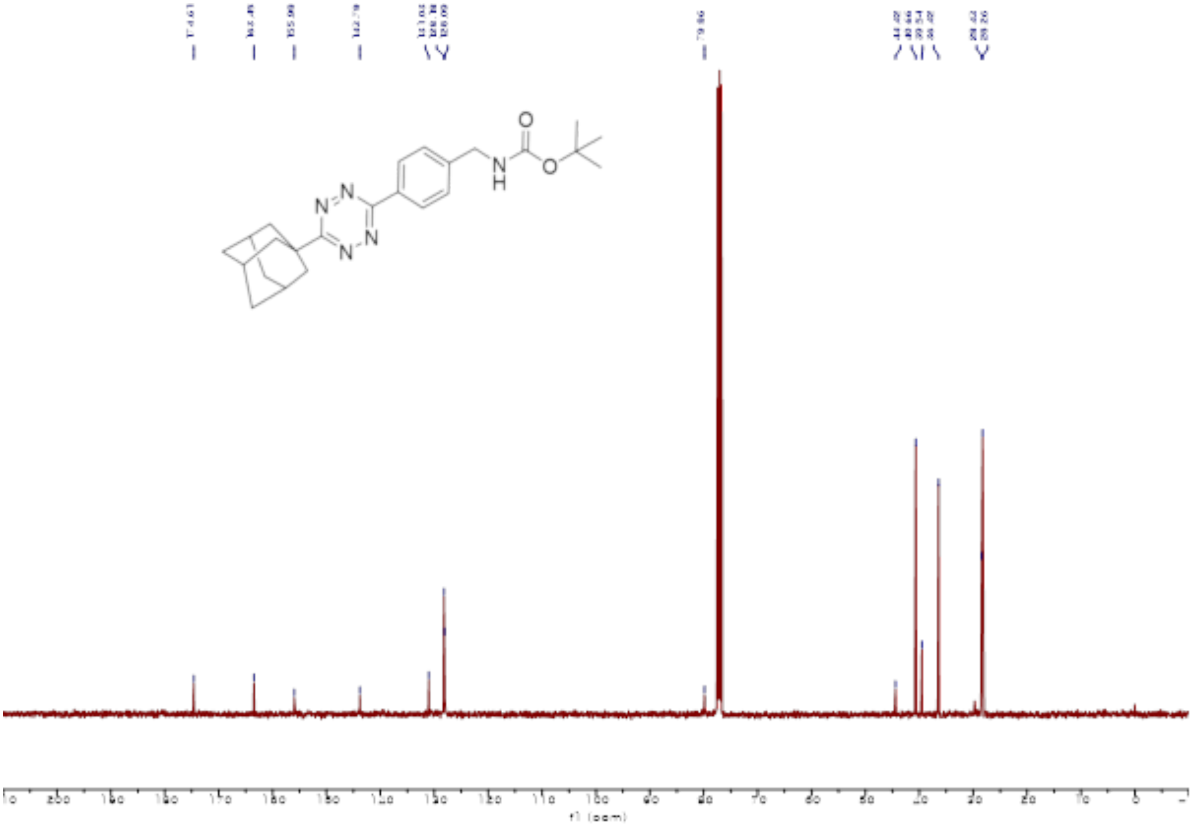

^{1}H NMR of B**oc-Tz20**

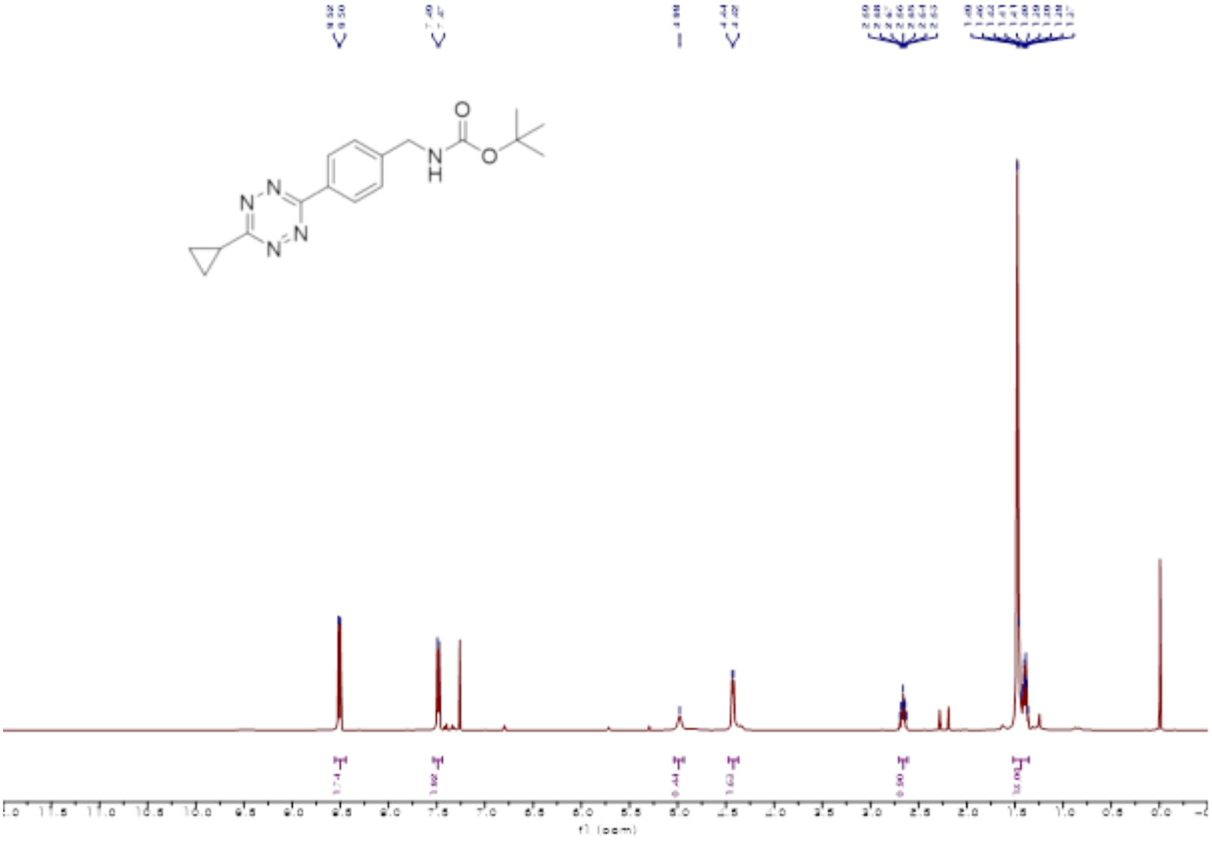

^{13}C NMR of **Boc-Tz20**

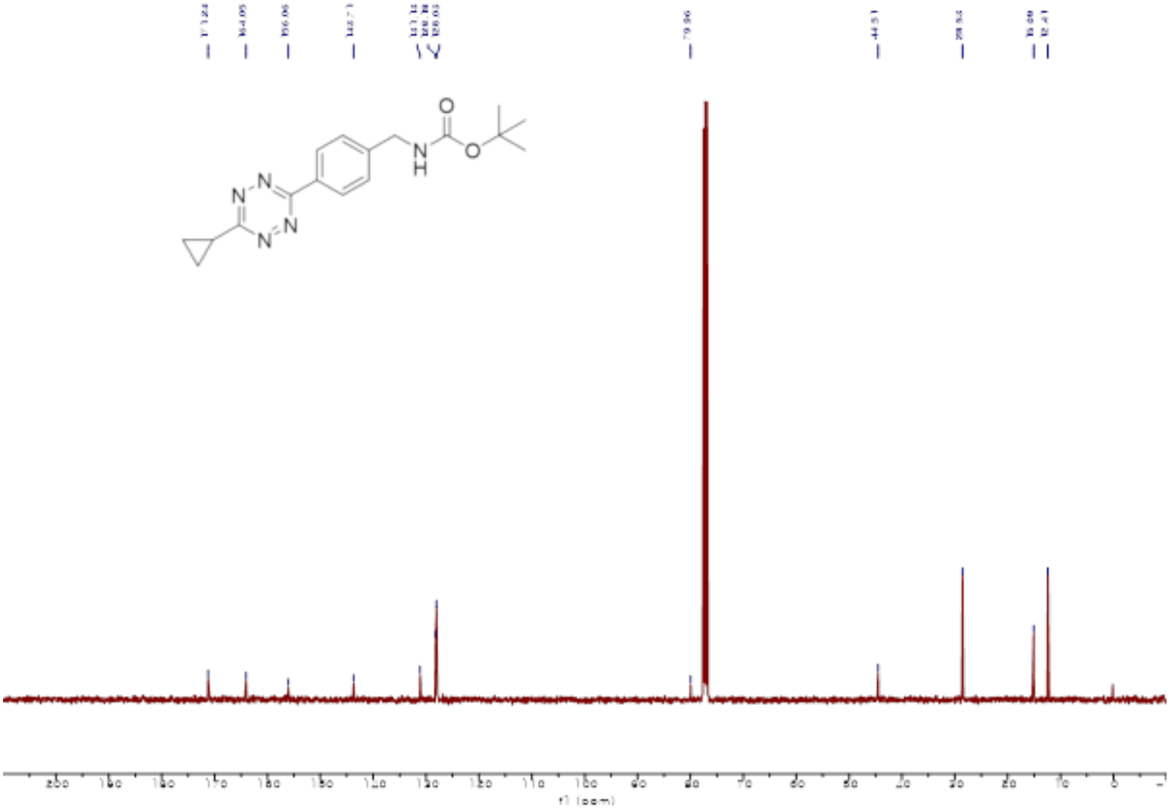

^{1}H NMR of B**oc-Tz22**

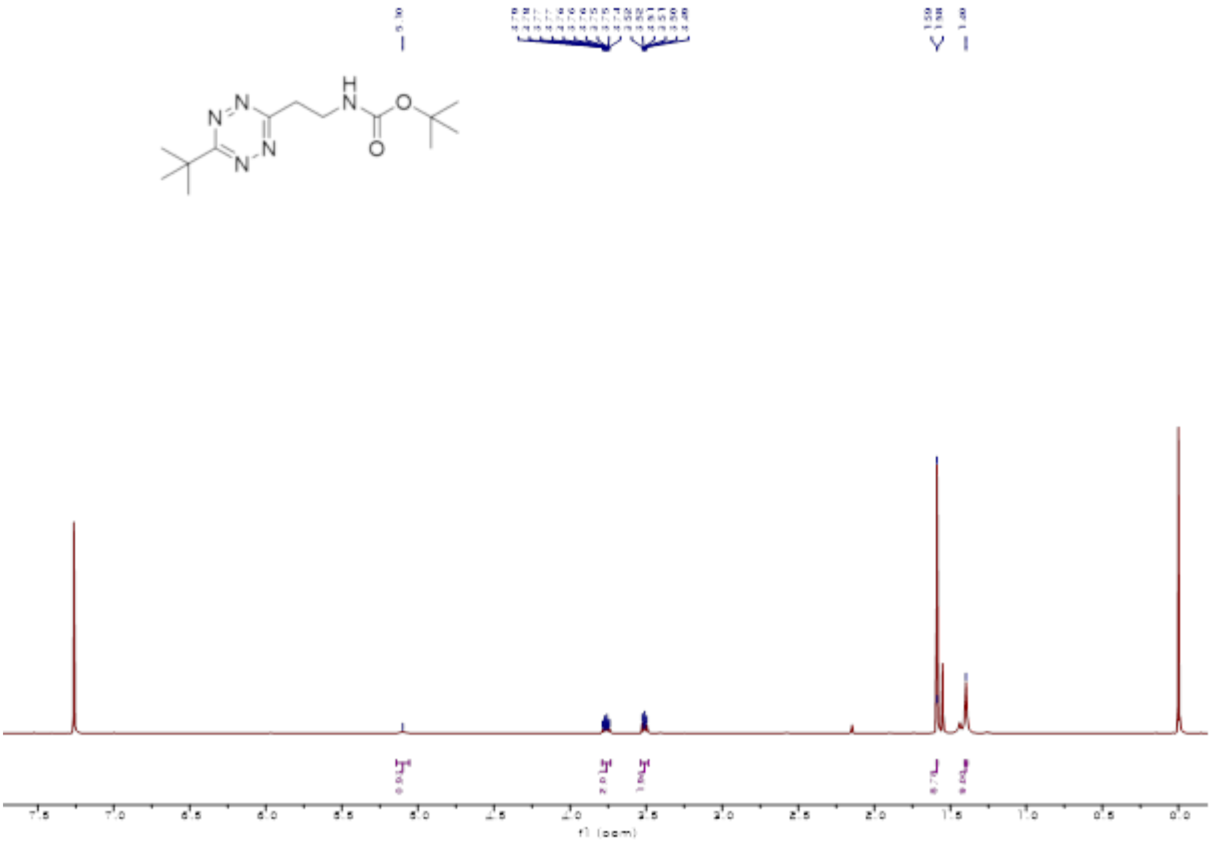

^{13}C NMR of **Boc-Tz22**

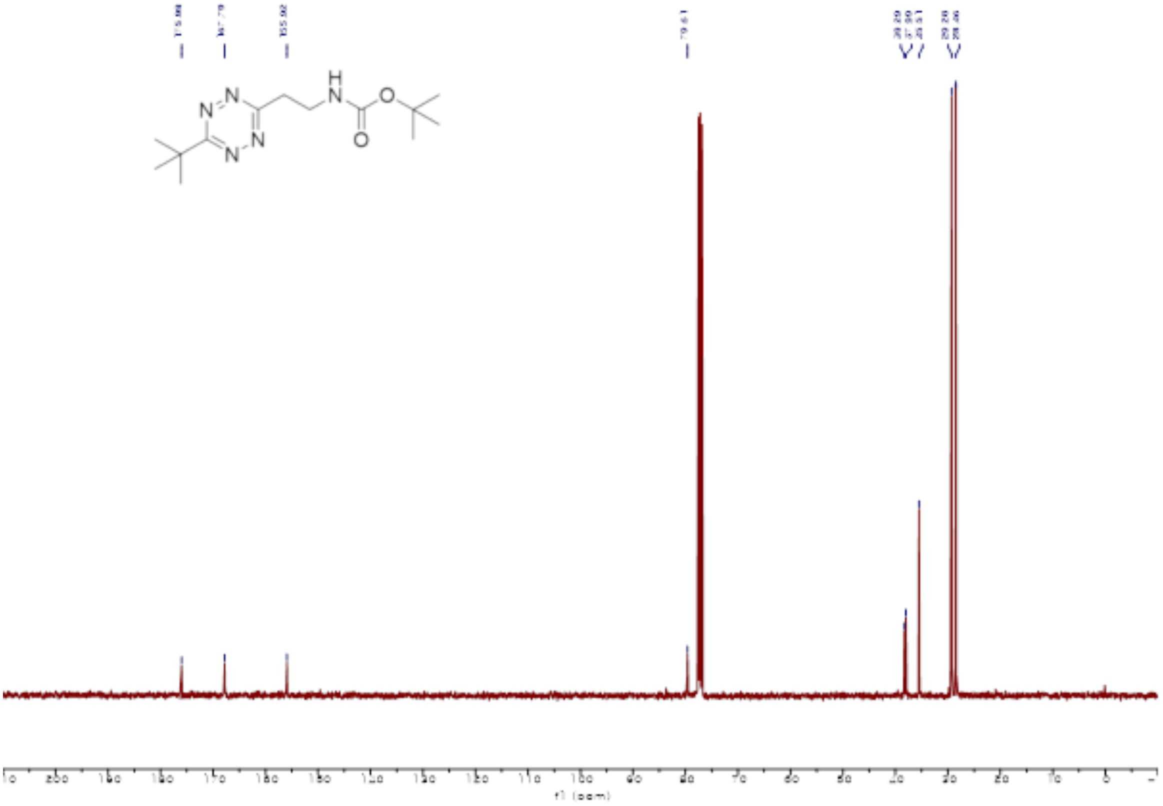

^{1}H NMR of B**oc-Tz23**

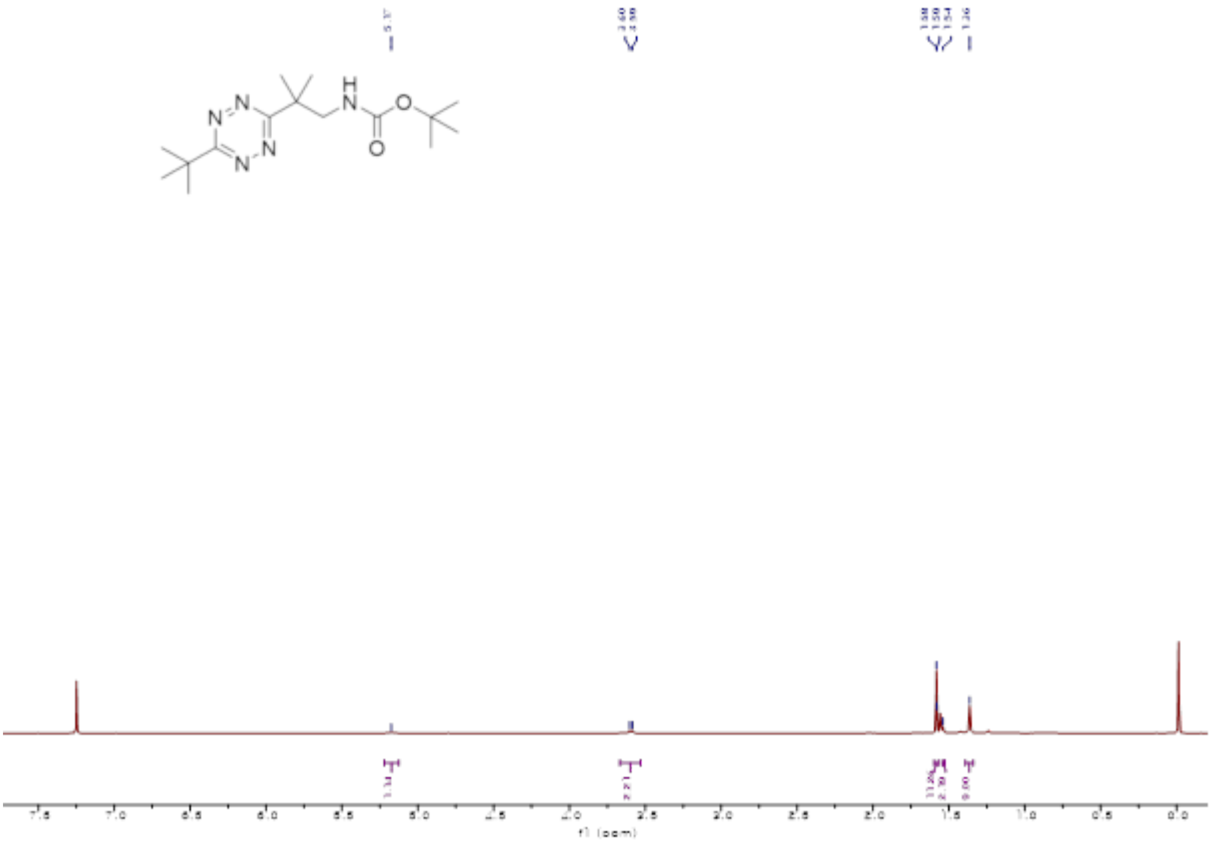

^{13}C NMR of **Boc-Tz23**

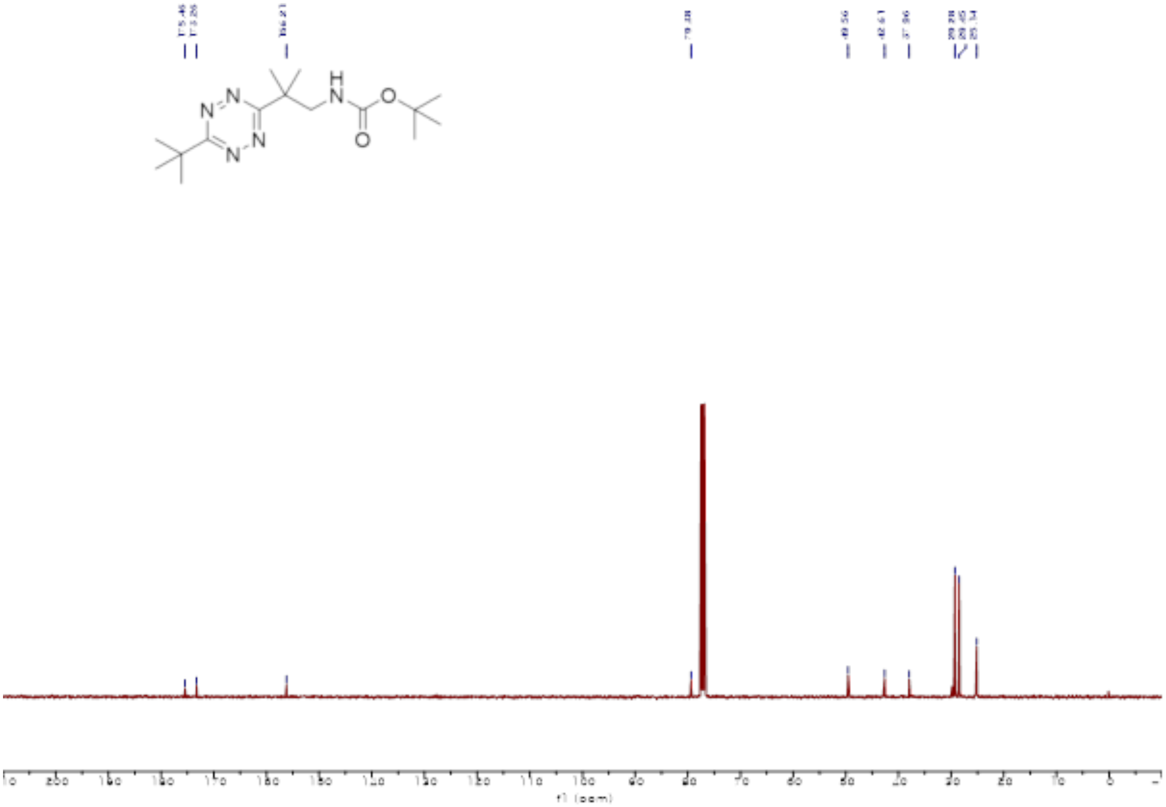

^{1}H NMR of **SiR-Tz1**

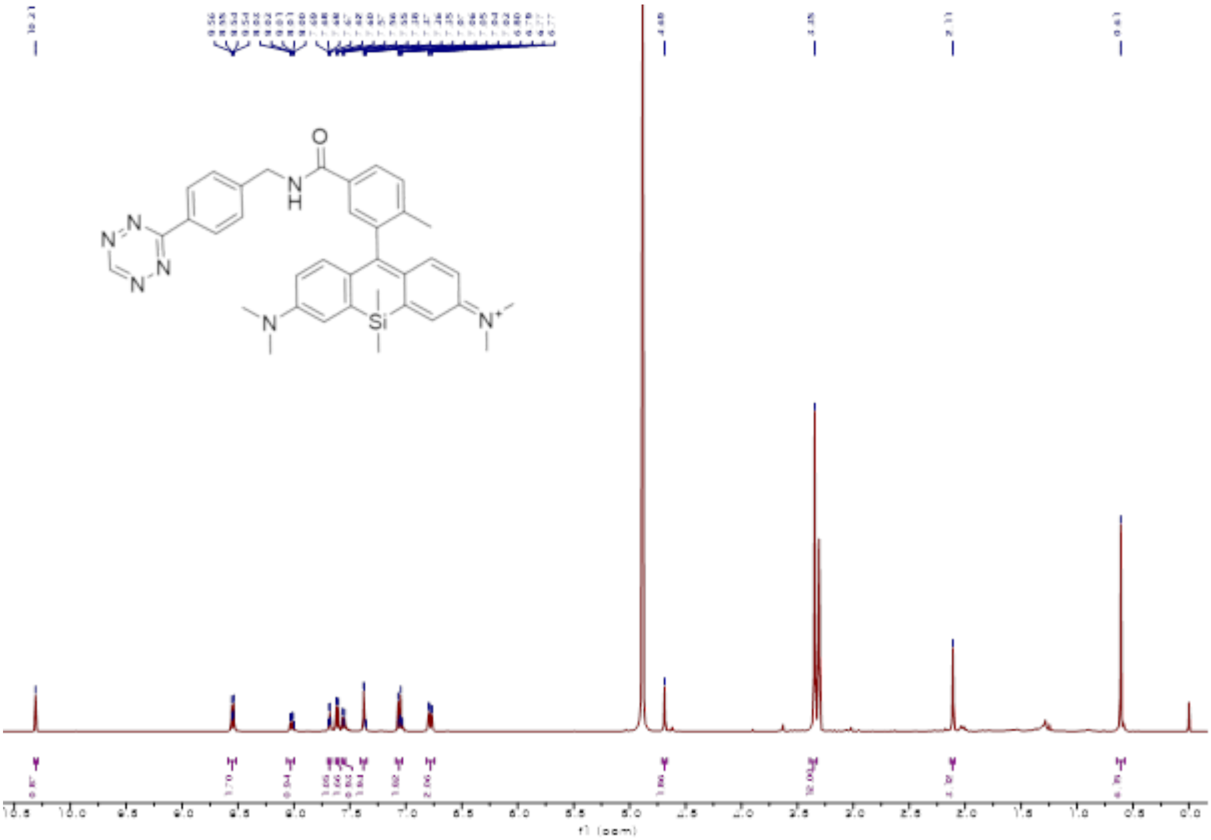

^{13}C NMR of **SiR-Tz1**

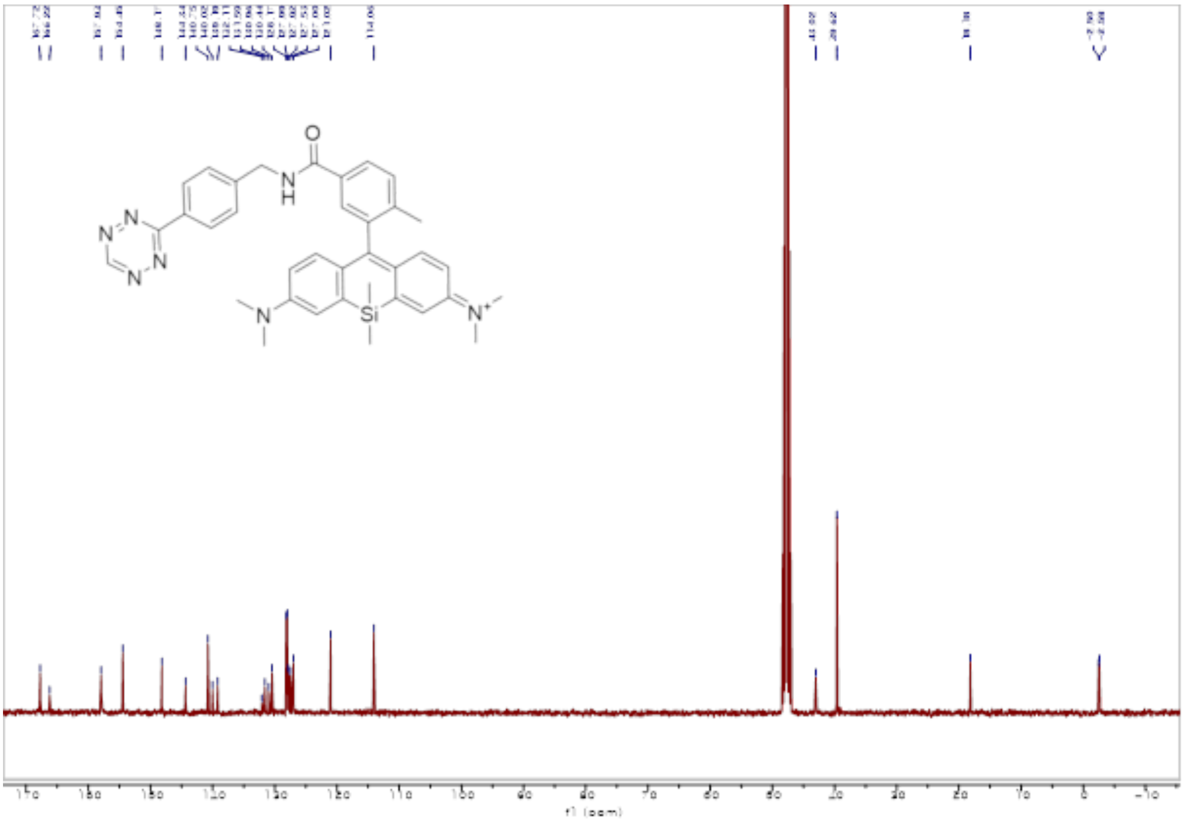

^{1}H NMR of **SiR-Tz2**

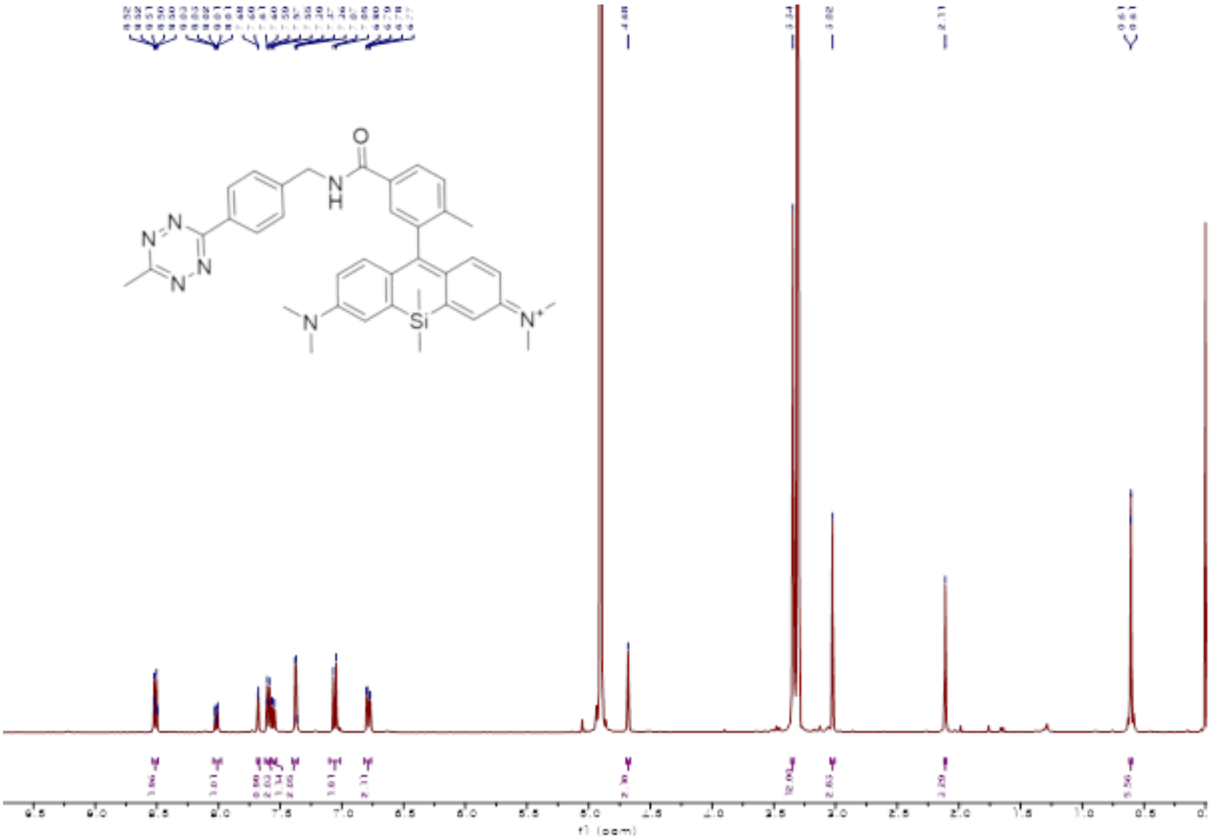

^{13}C NMR of **SiR-Tz2**

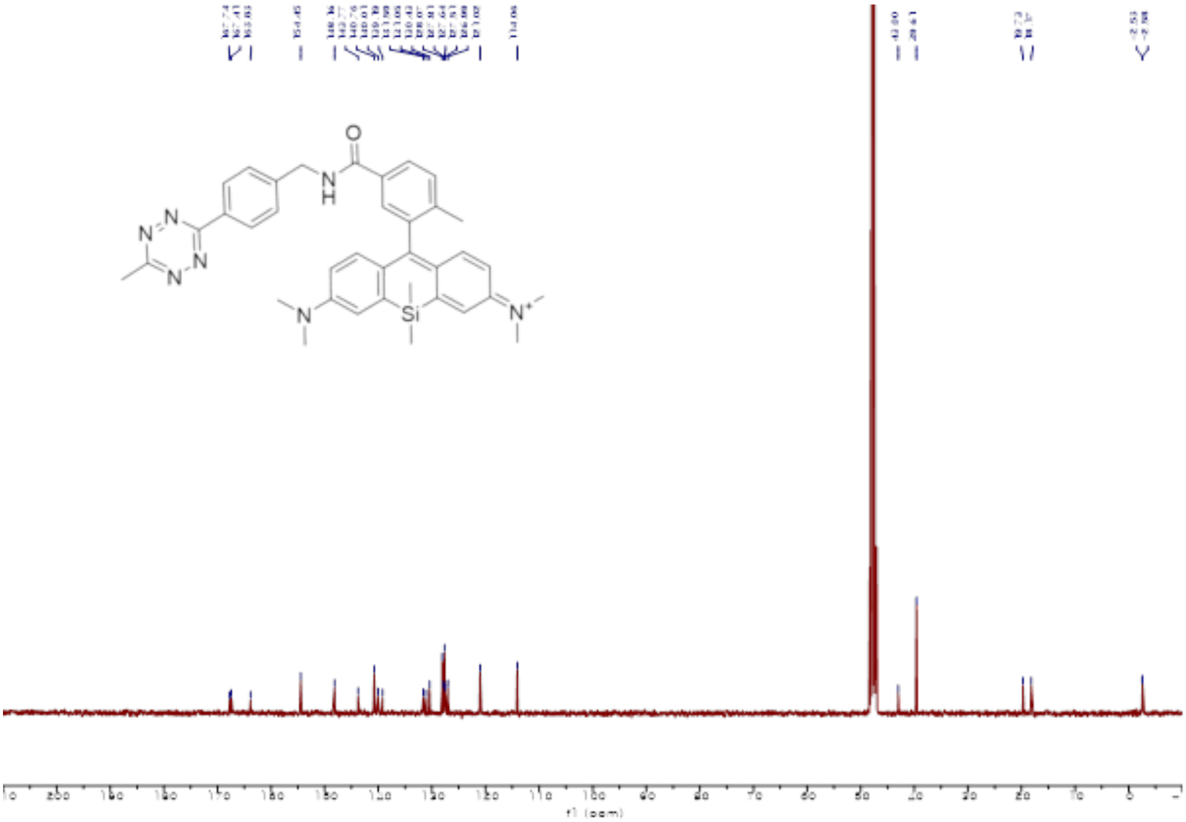

^{1}H NMR of **SiR-Tz3**

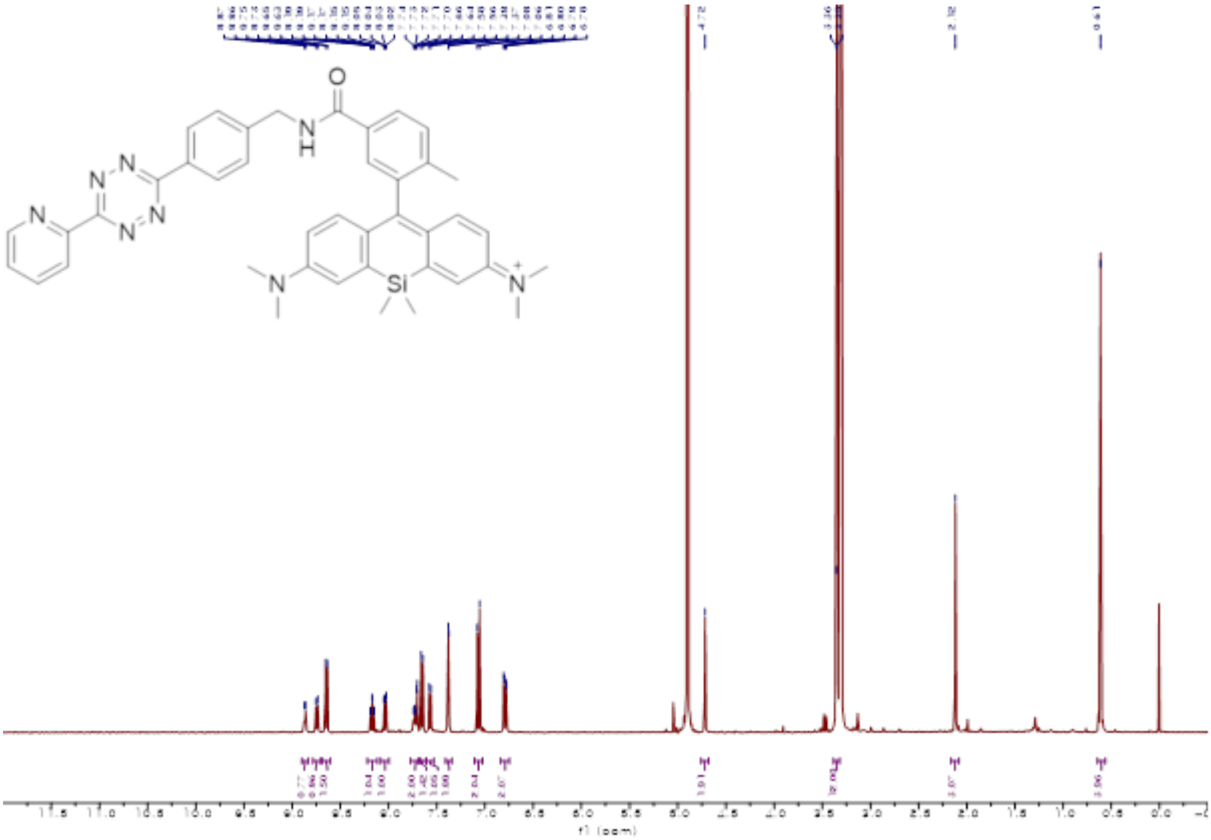

^{13}C NMR of **SiR-Tz3**

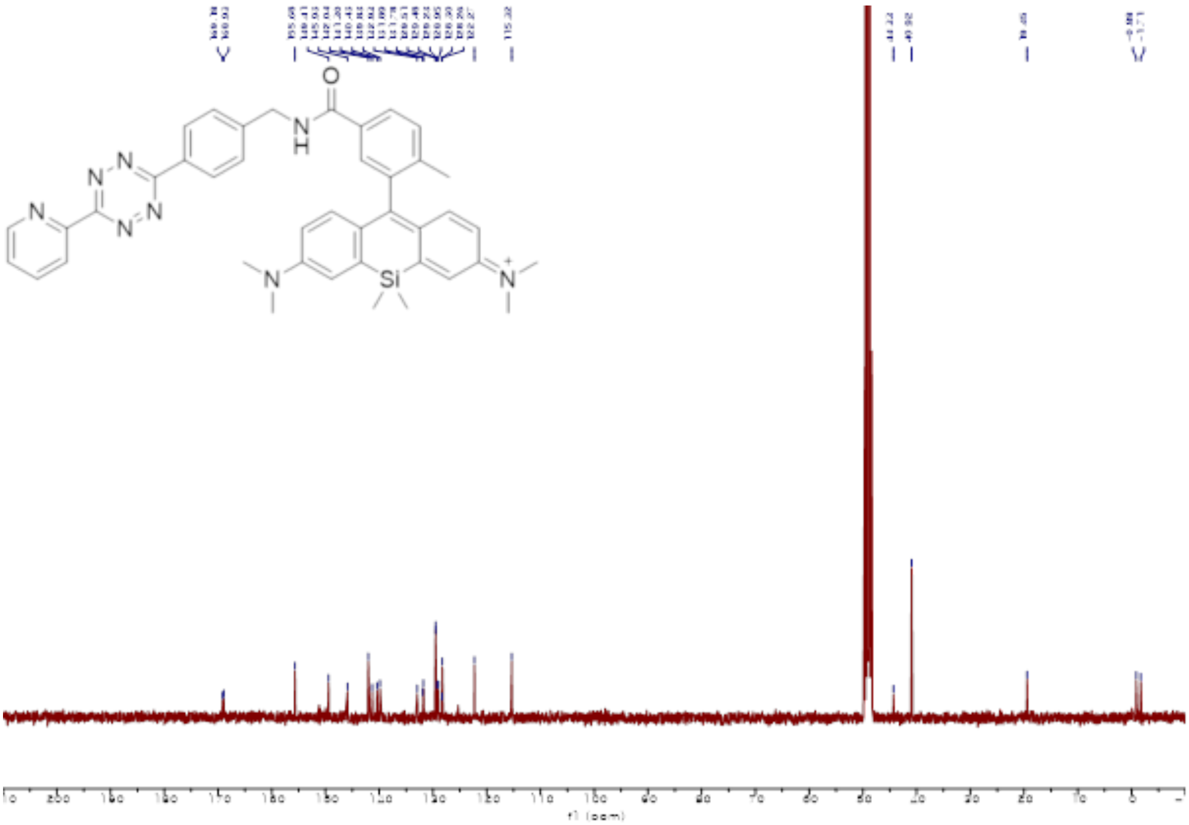

^{1}H NMR of **SiR-Tz4**

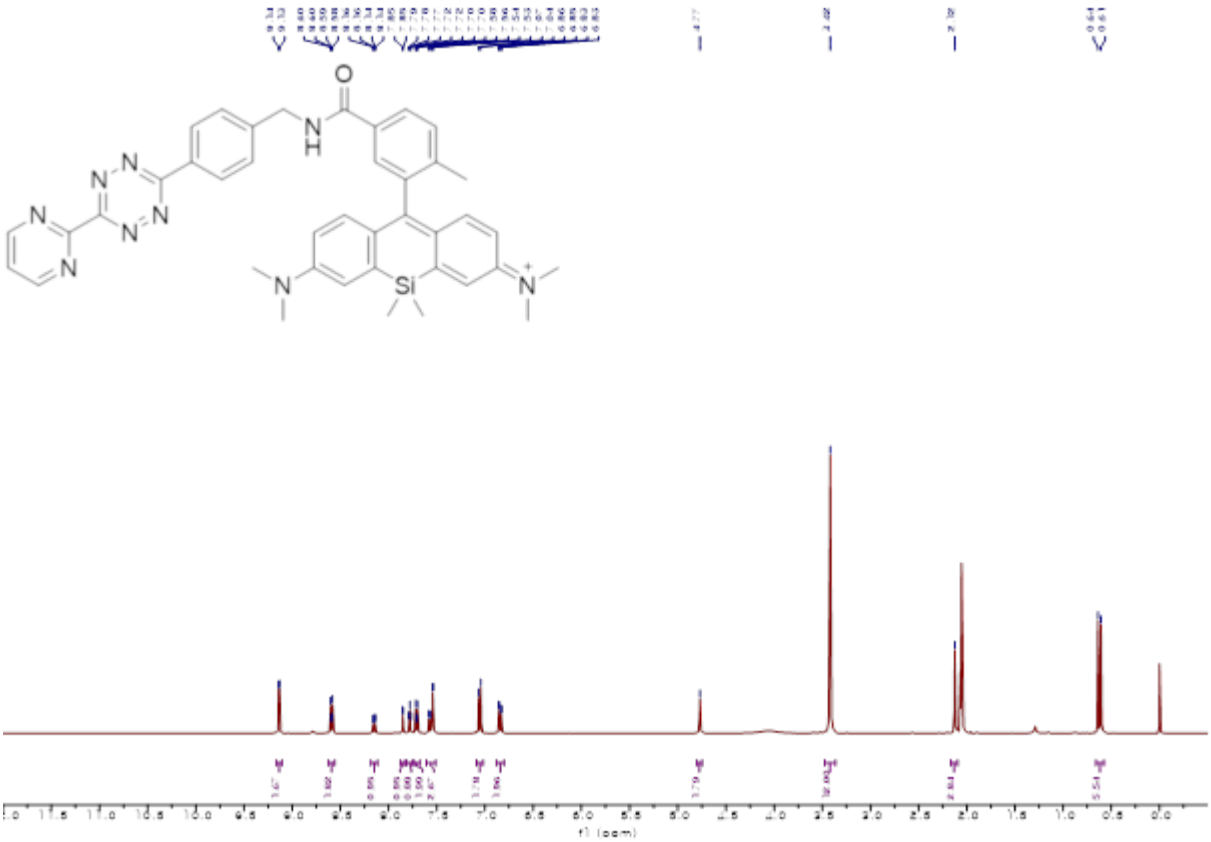

^{13}C NMR of **SiR-Tz4**

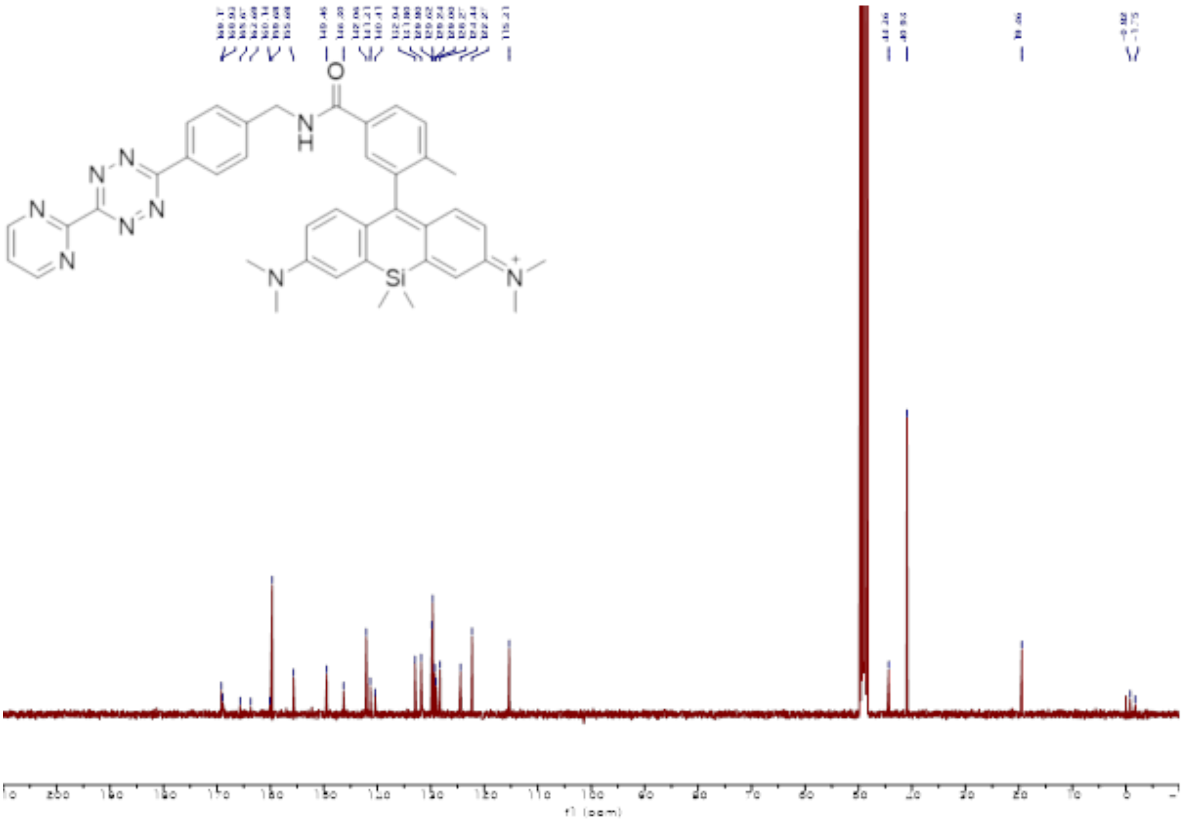

^{1}H NMR of **SiR-Tz5**

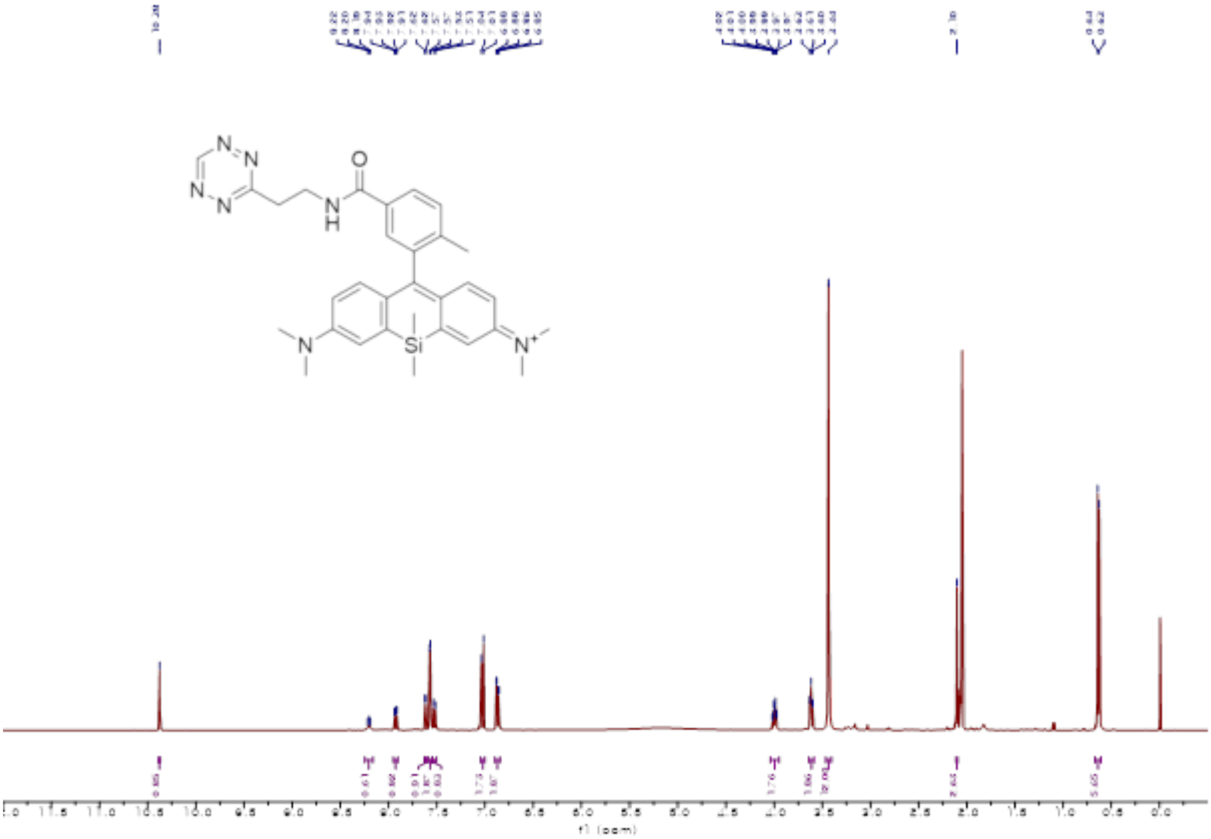

^{13}C NMR of **SiR-Tz5**

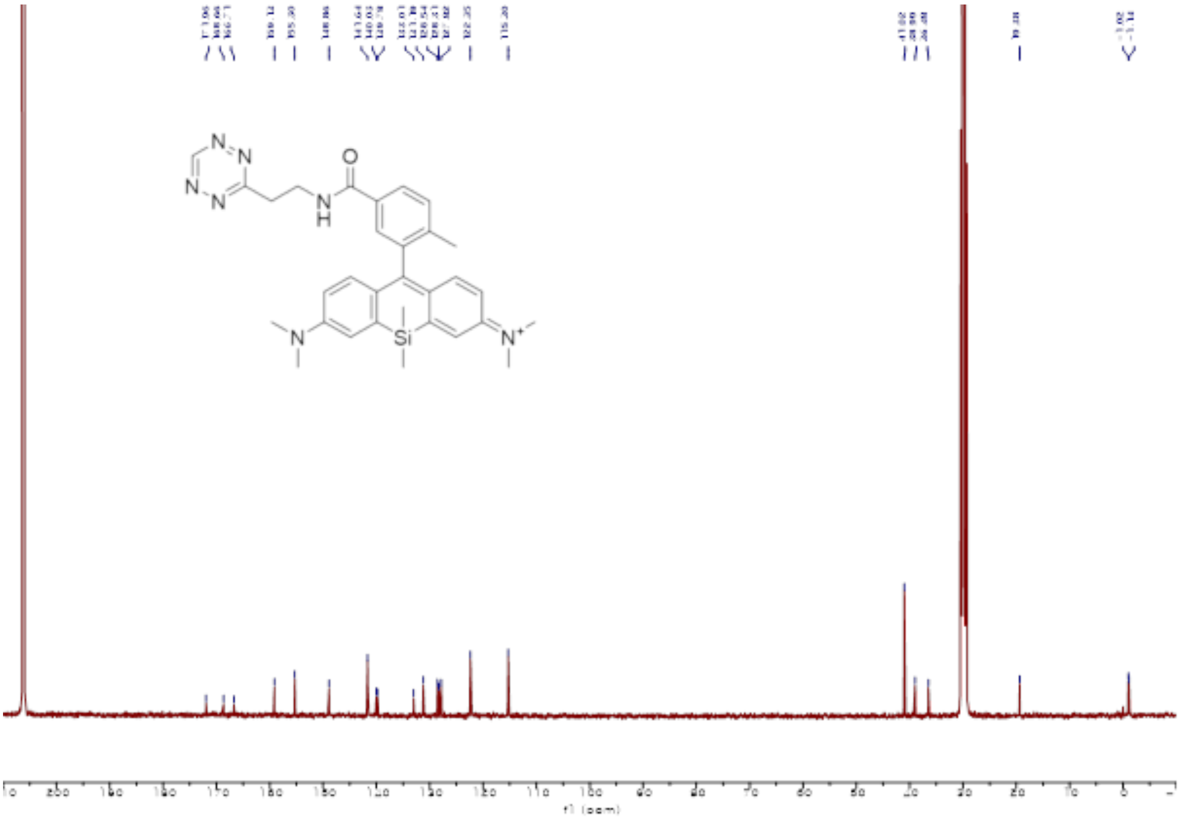

^{1}H NMR of **SiR-Tz6**

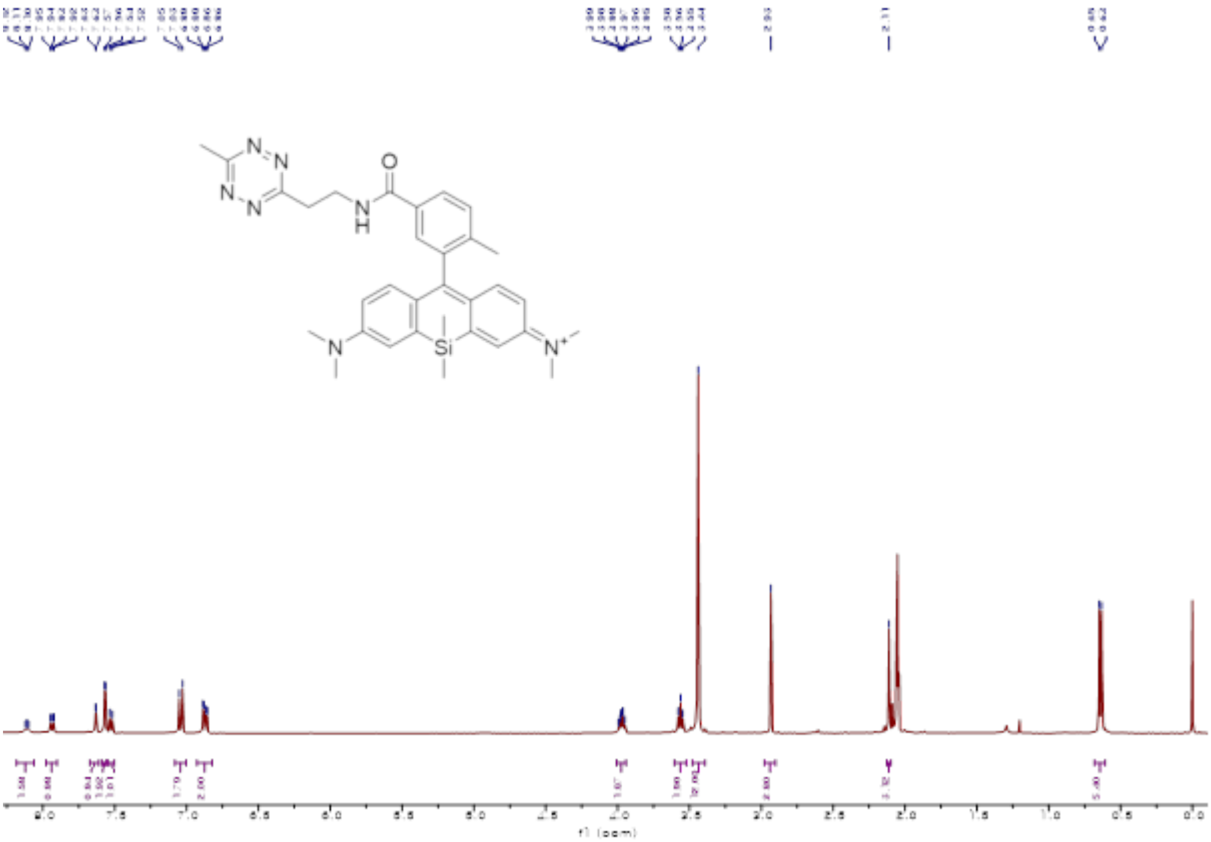

^{13}C NMR of **SiR-Tz6**

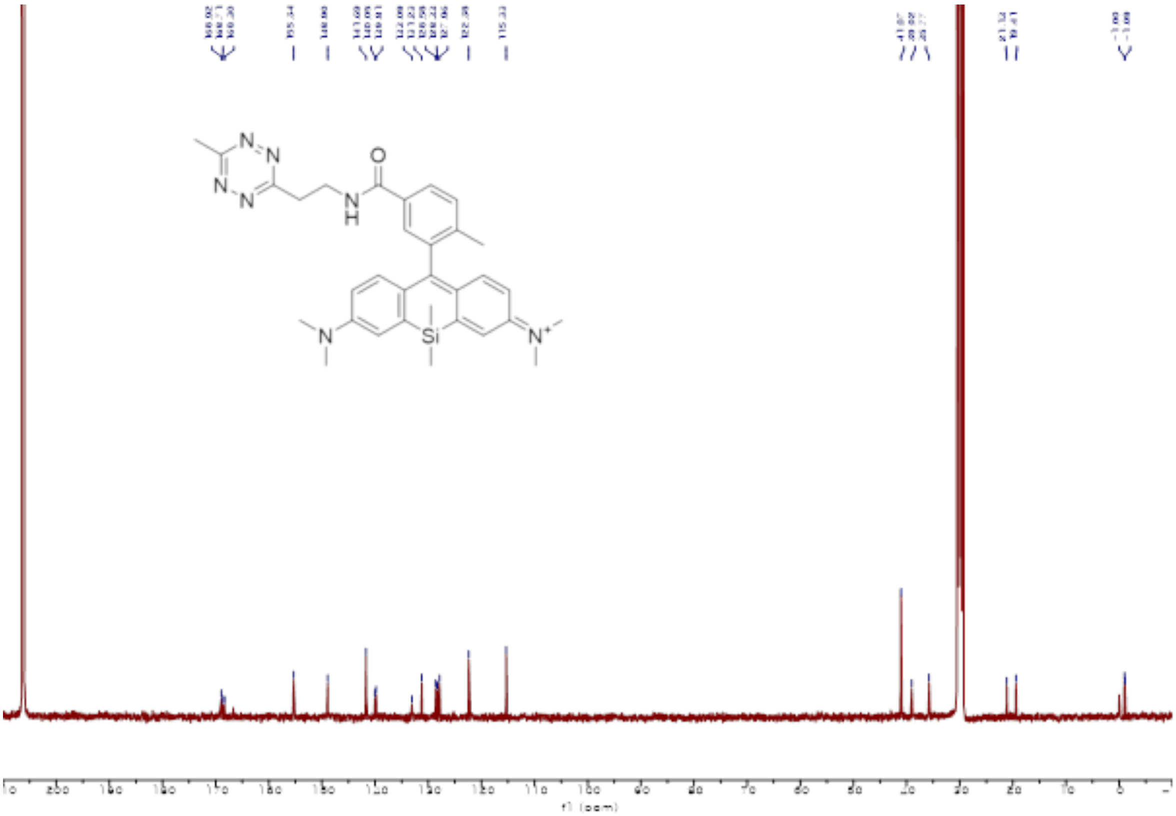

^{1}H NMR of **SiR-Tz7**

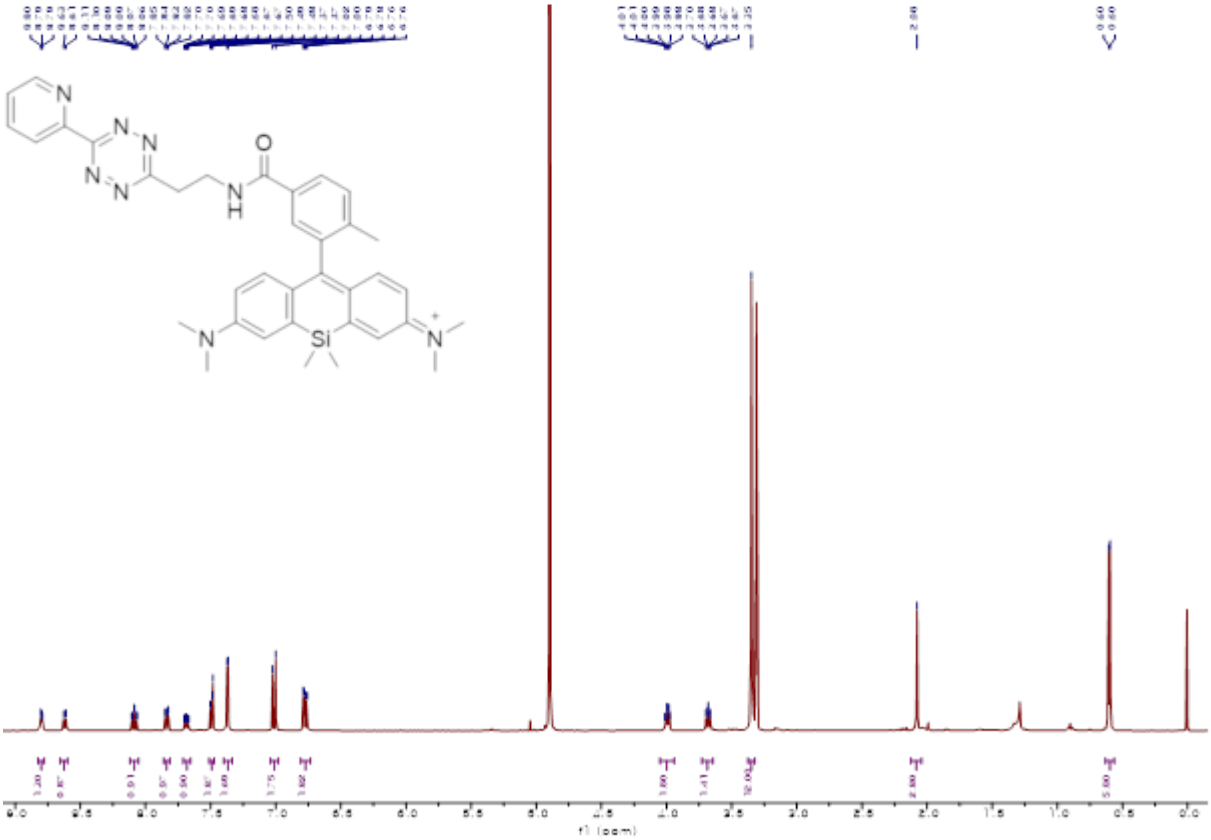

^{13}C NMR of **SiR-Tz7**

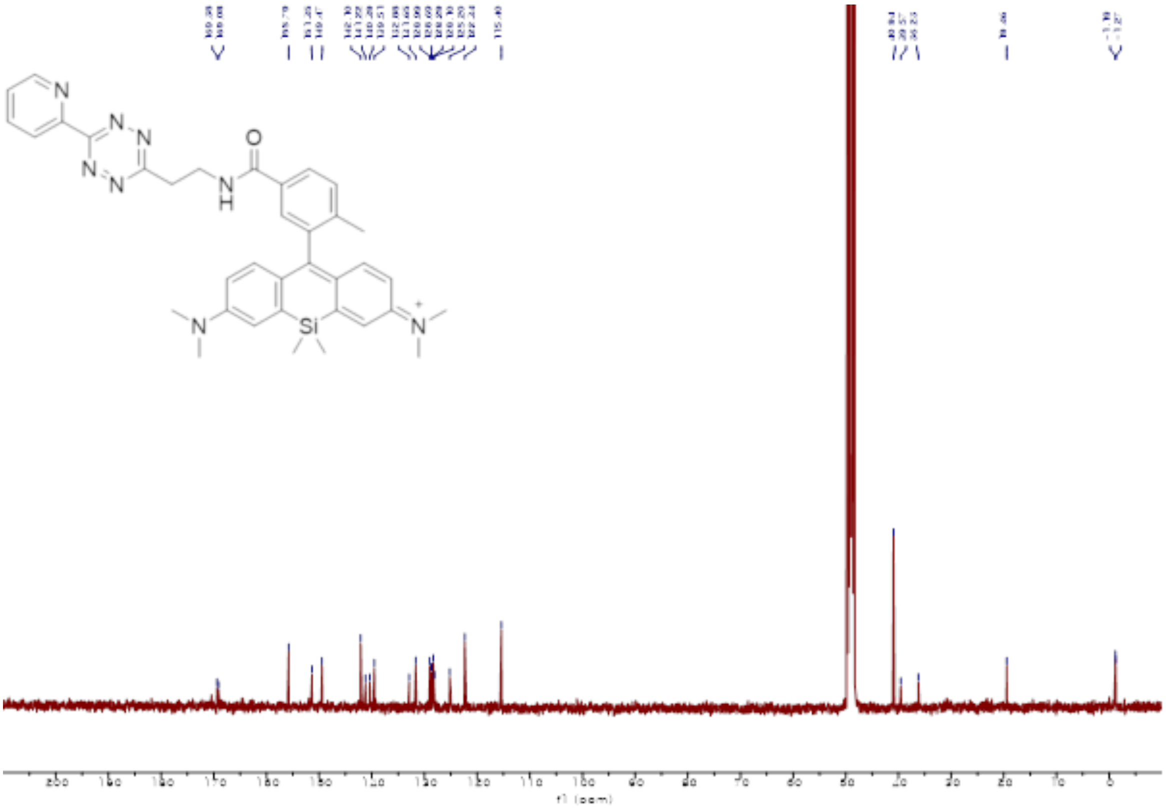

^{1}H NMR of **SiR-Tz8**

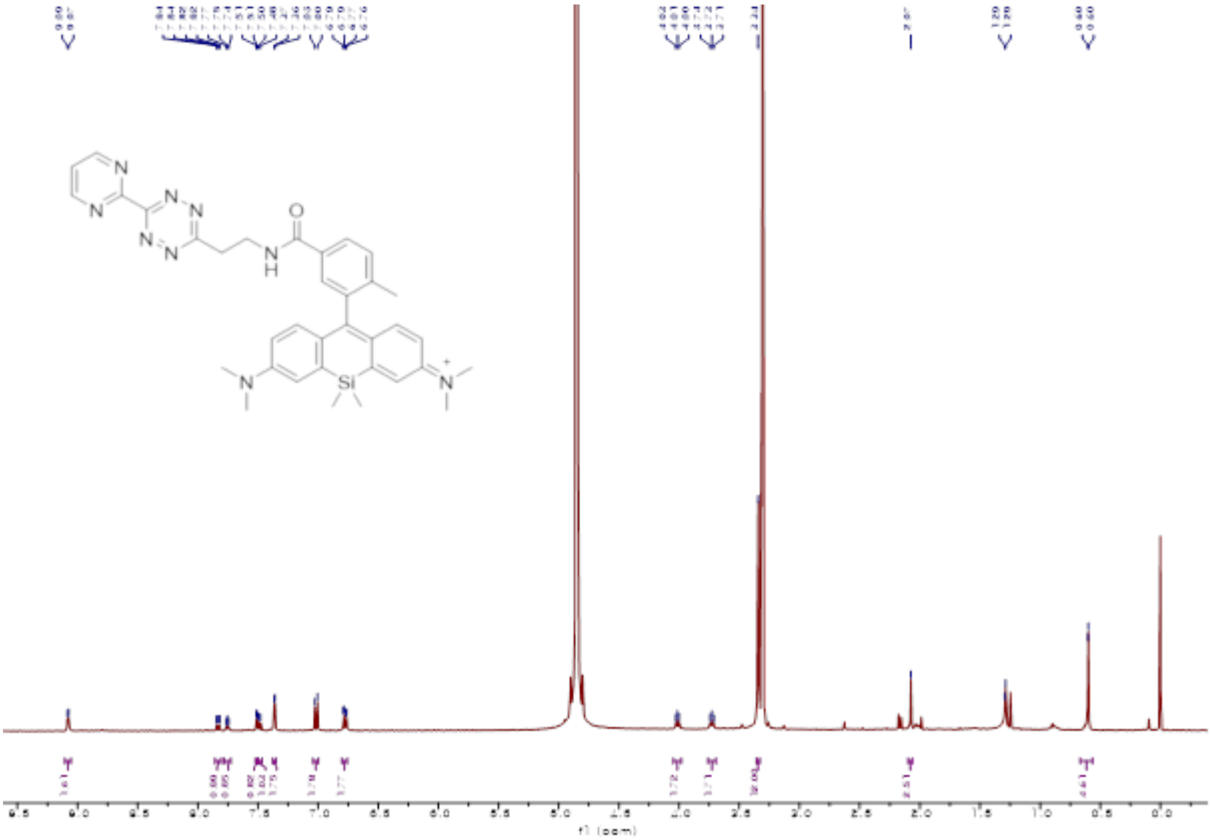

^{13}C NMR of **SiR-Tz8**

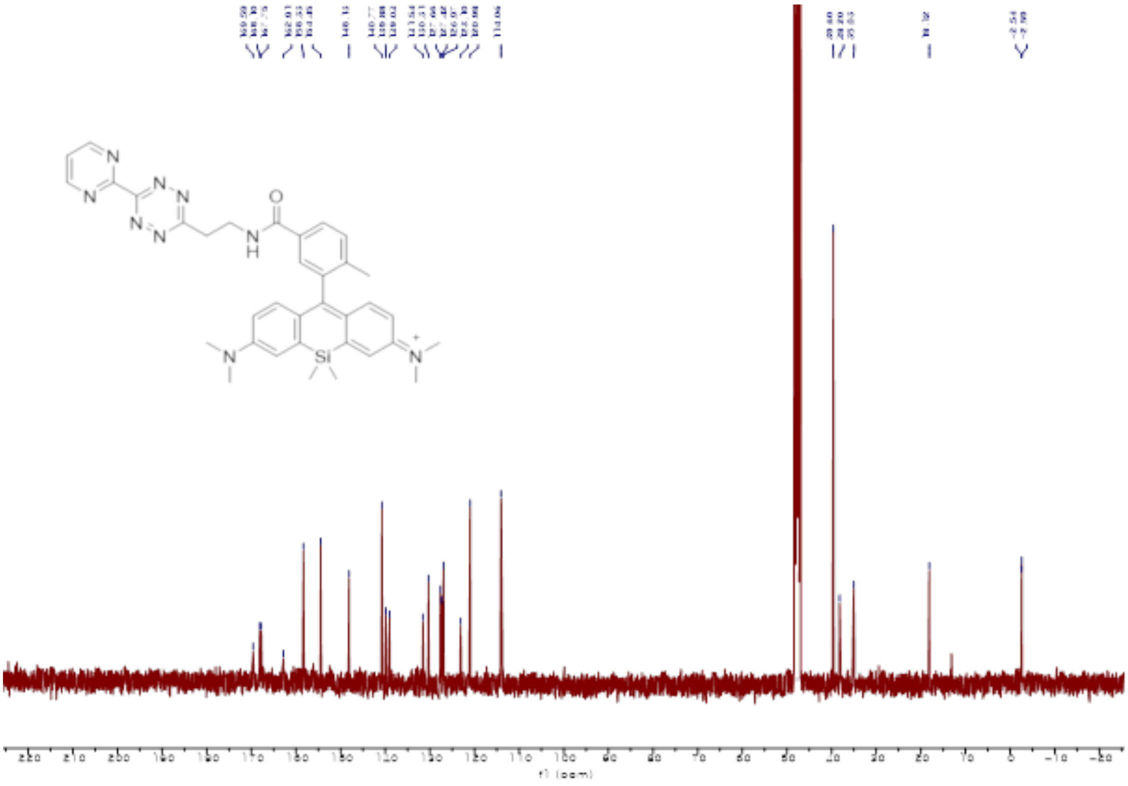

^{1}H NMR of **SiR-Tz9**

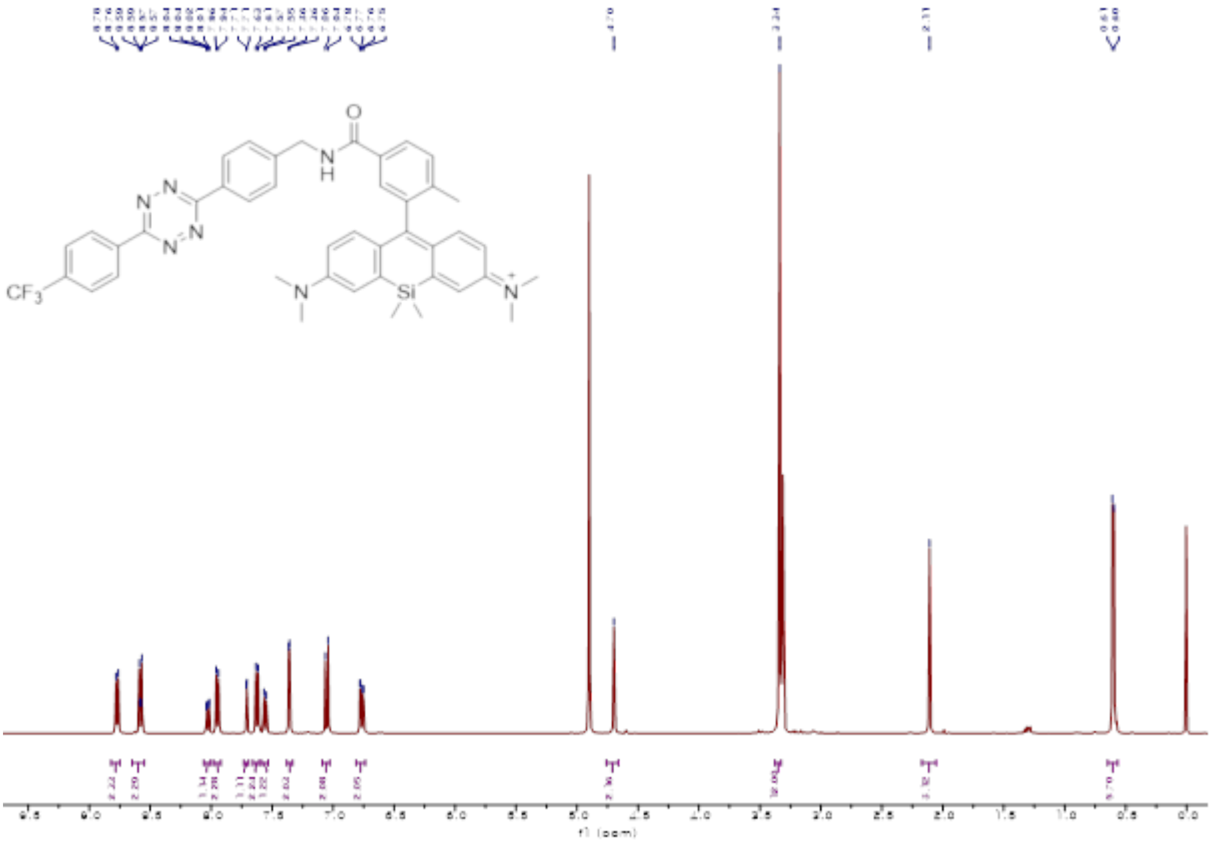

^{13}C NMR of **SiR-Tz9**

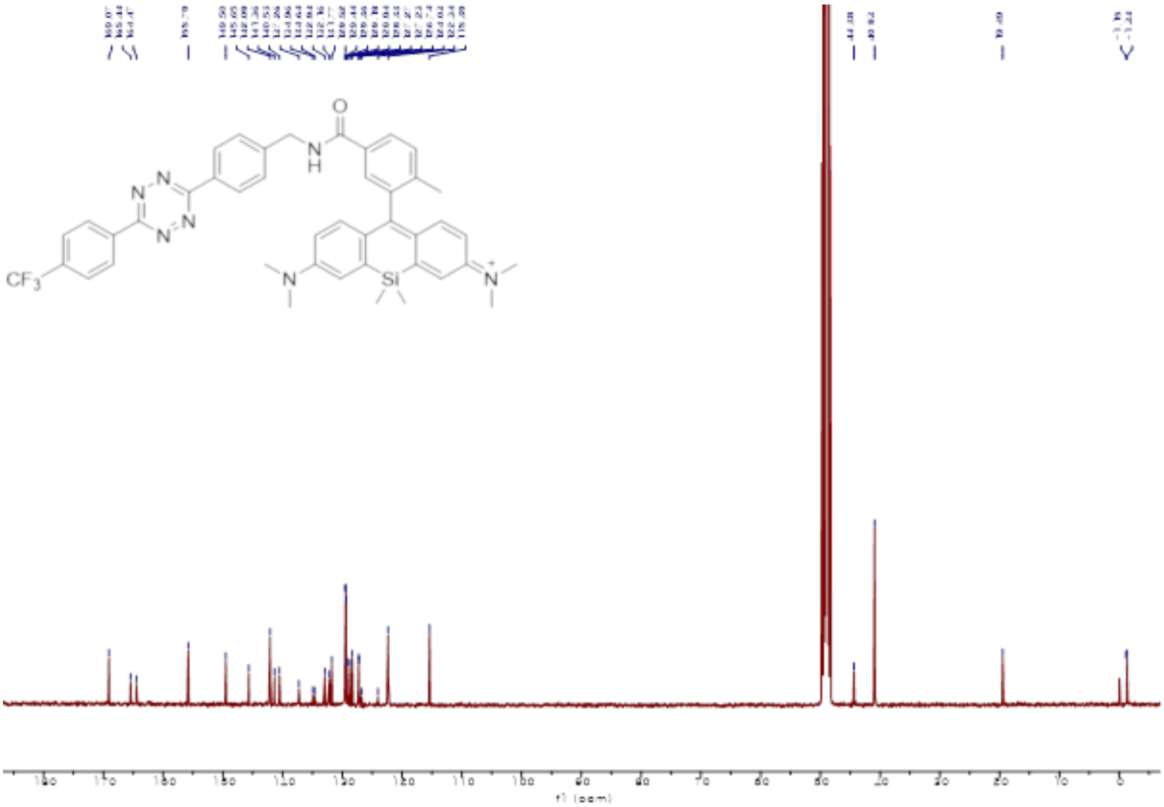

^{1}H NMR of **SiR-Tz10**

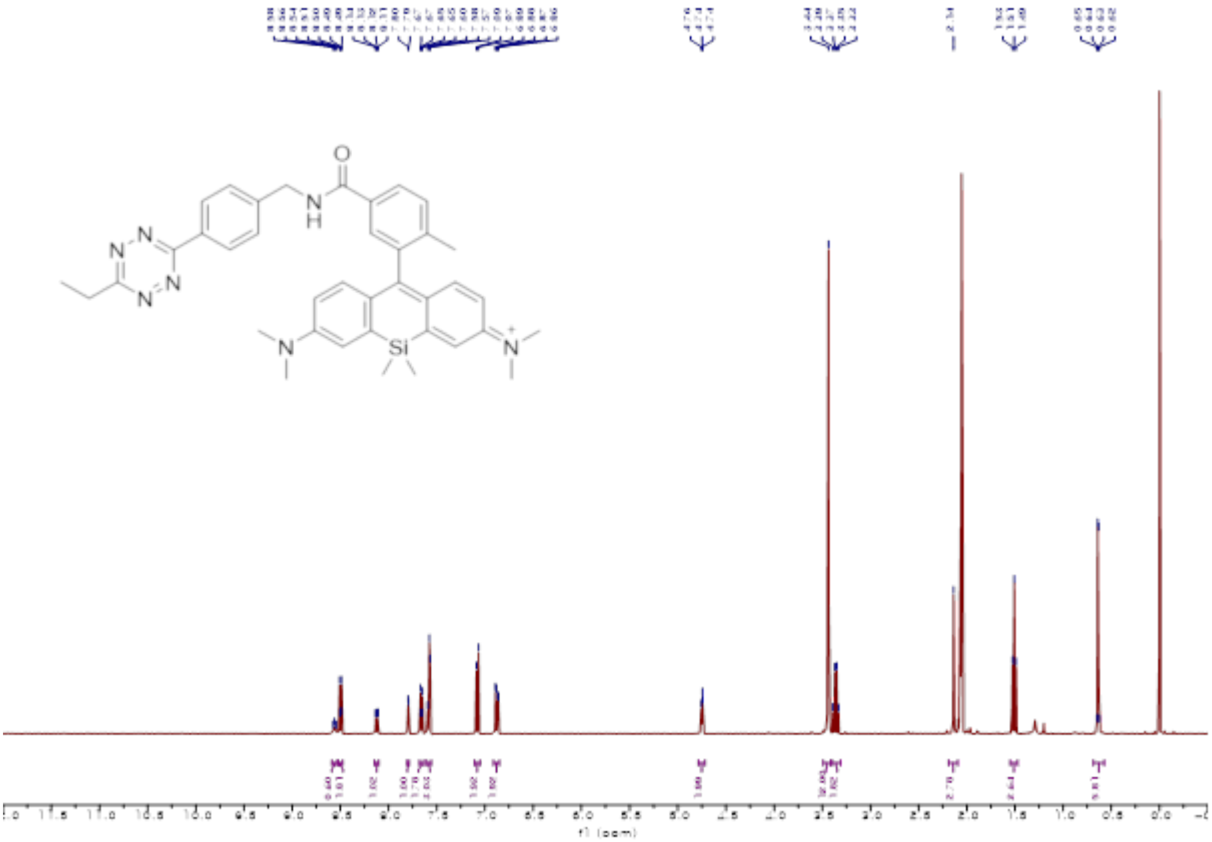

^{13}C NMR of **SiR-Tz10**

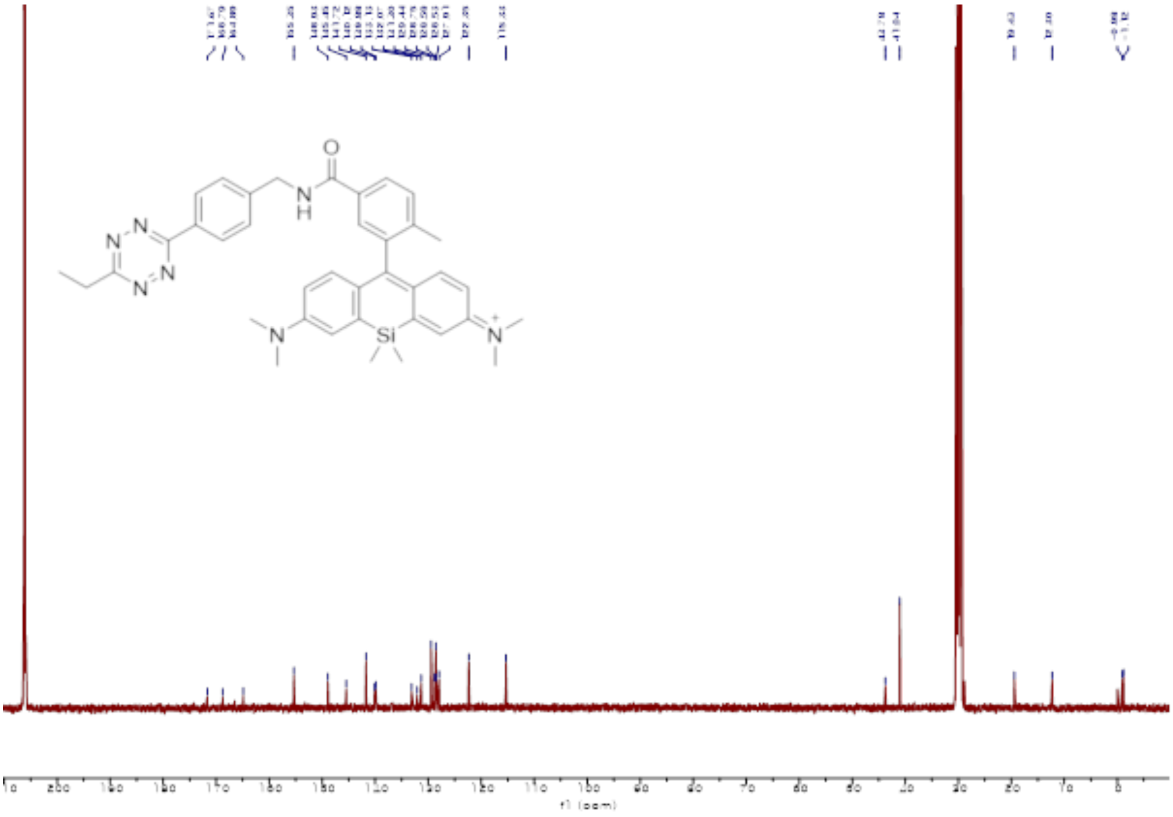

^{1}H NMR of **SiR-Tz11**

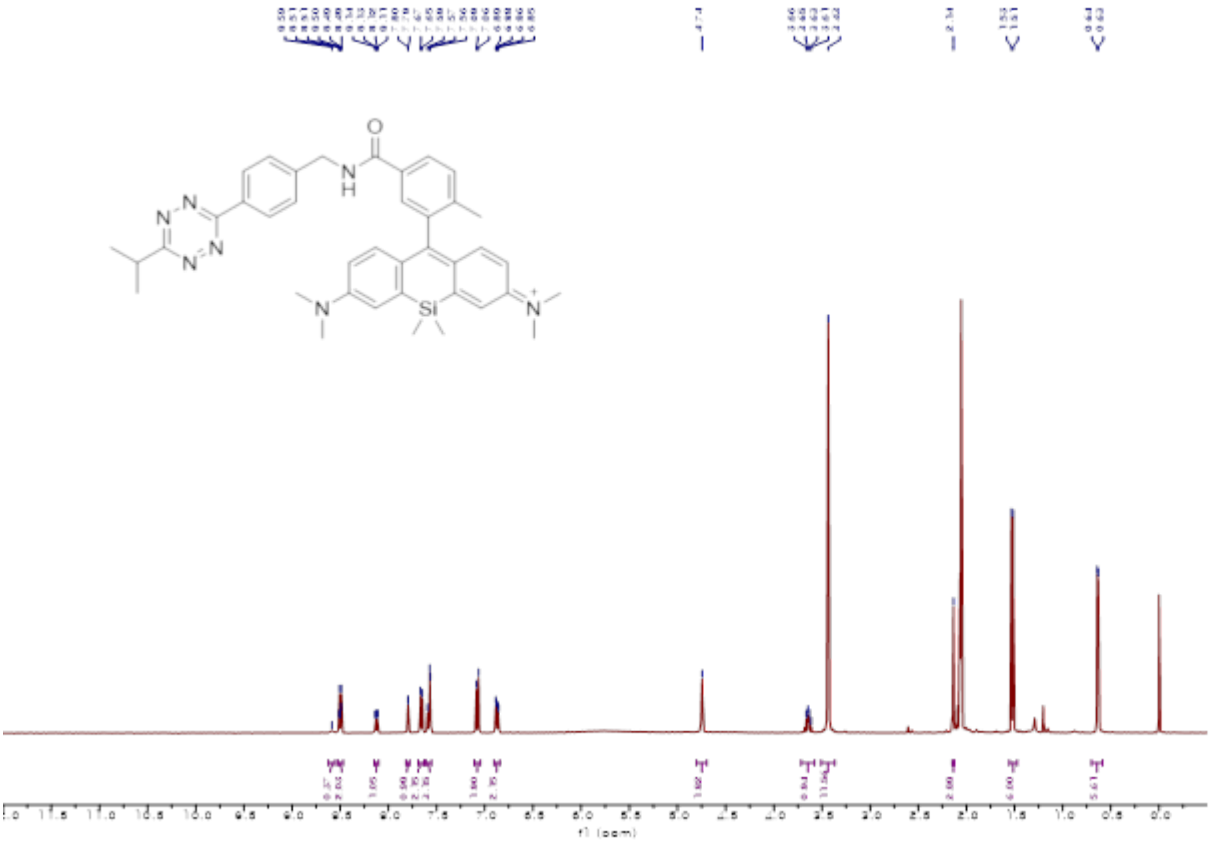

^{13}C NMR of **SiR-Tz11**

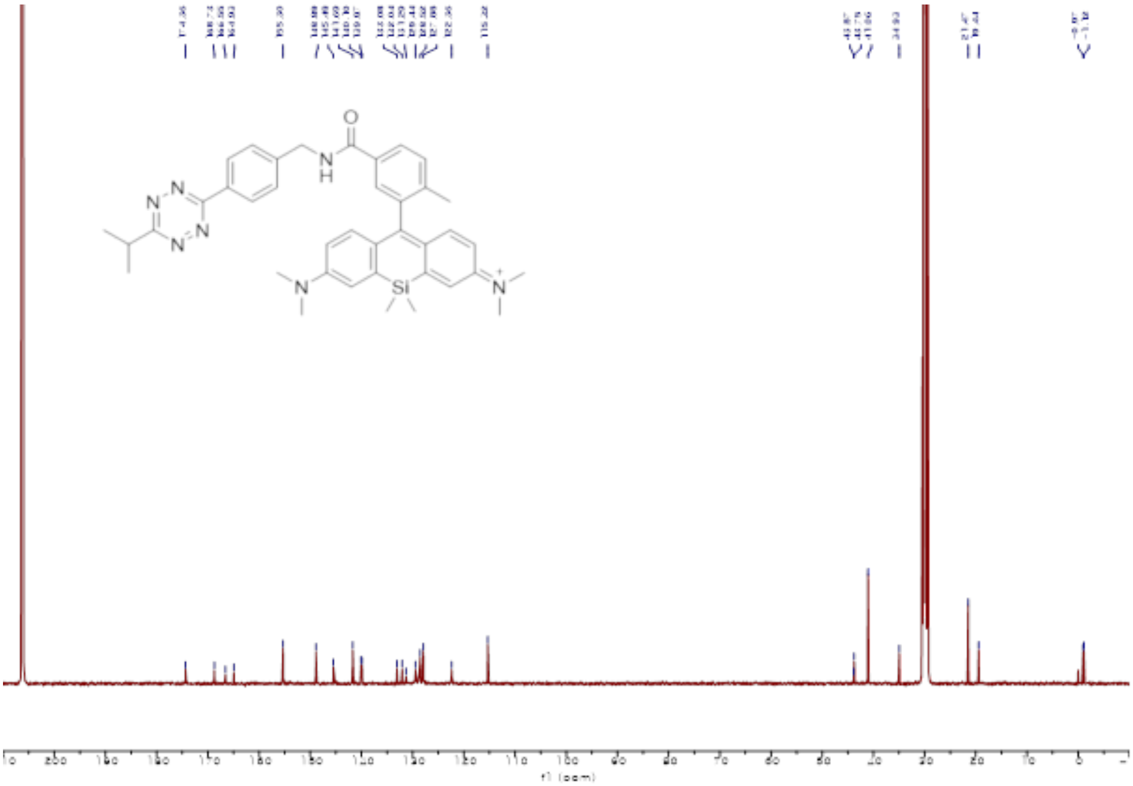

^{1}H NMR of **SiR-Tz12**

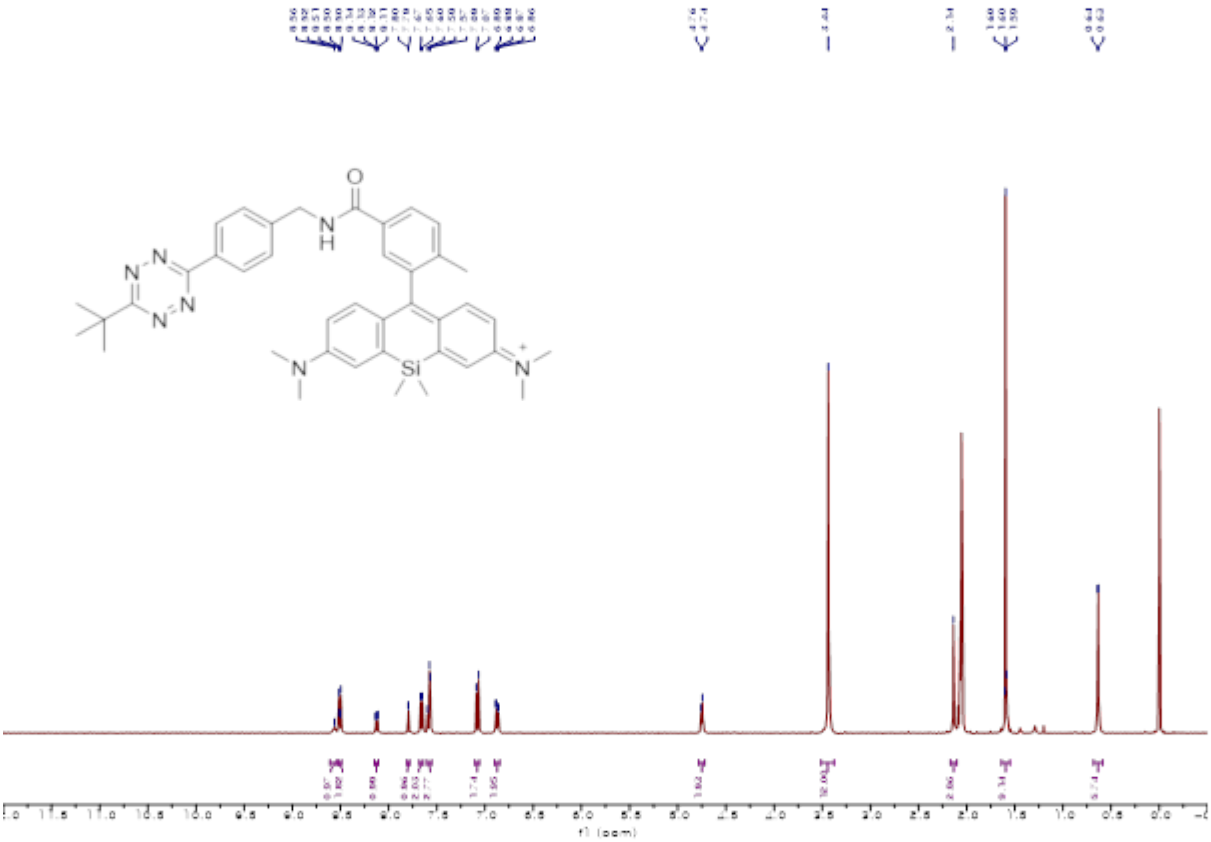

^{13}C NMR of **SiR-Tz12**

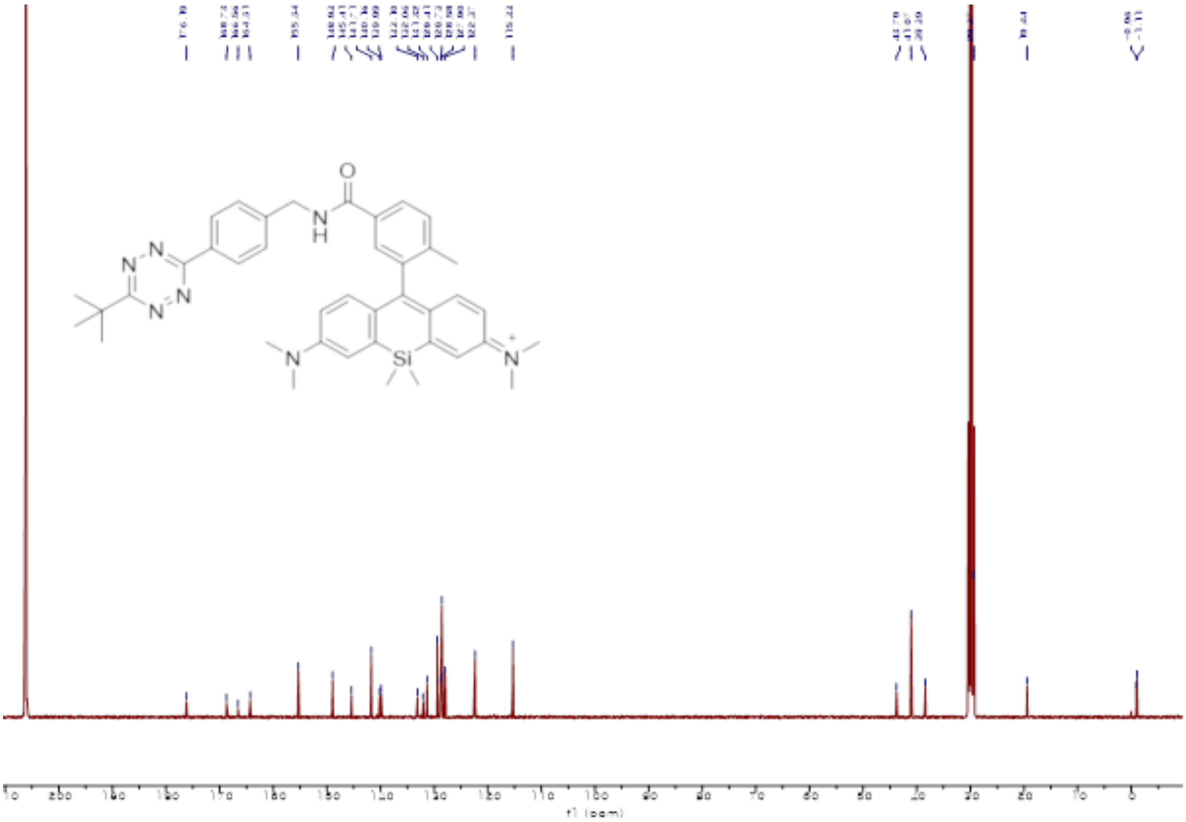

^{1}H NMR of **SiR-Tz13**

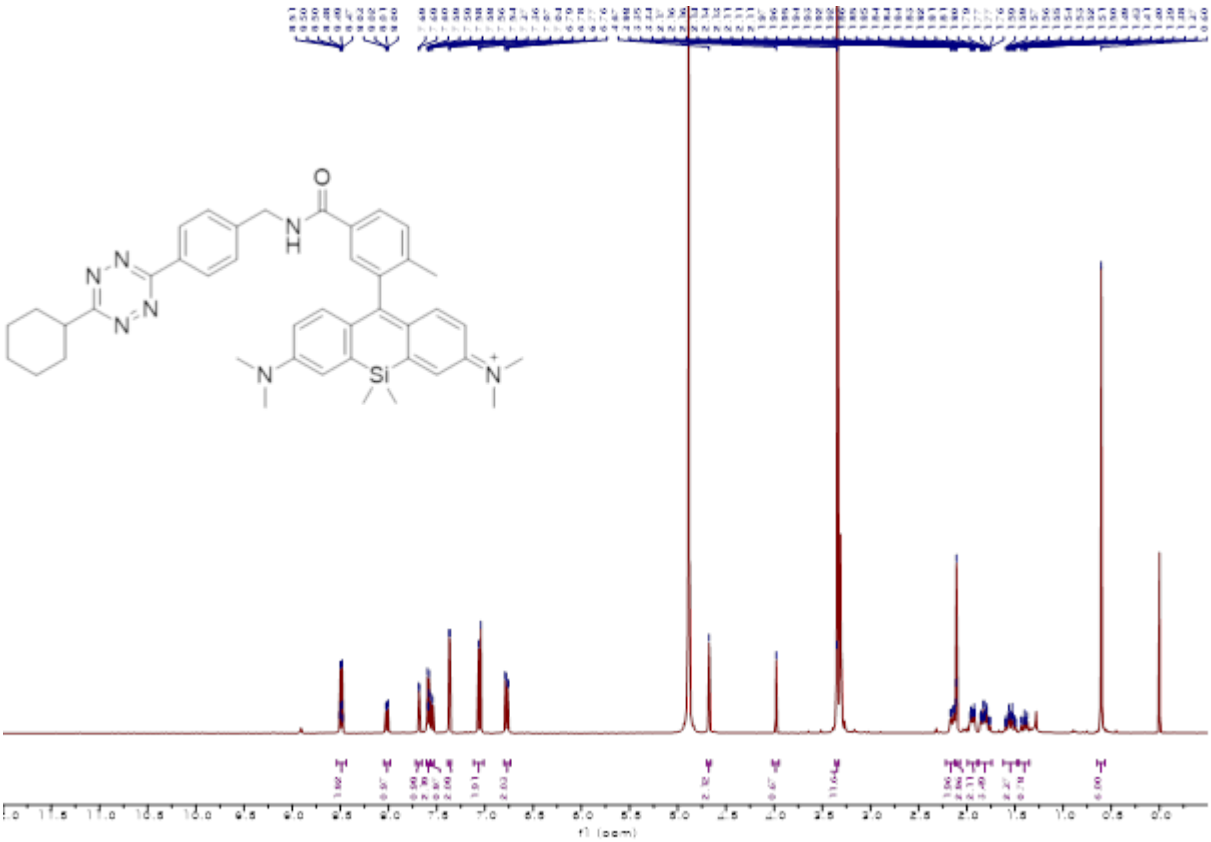

^{13}C NMR of **SiR-Tz13**

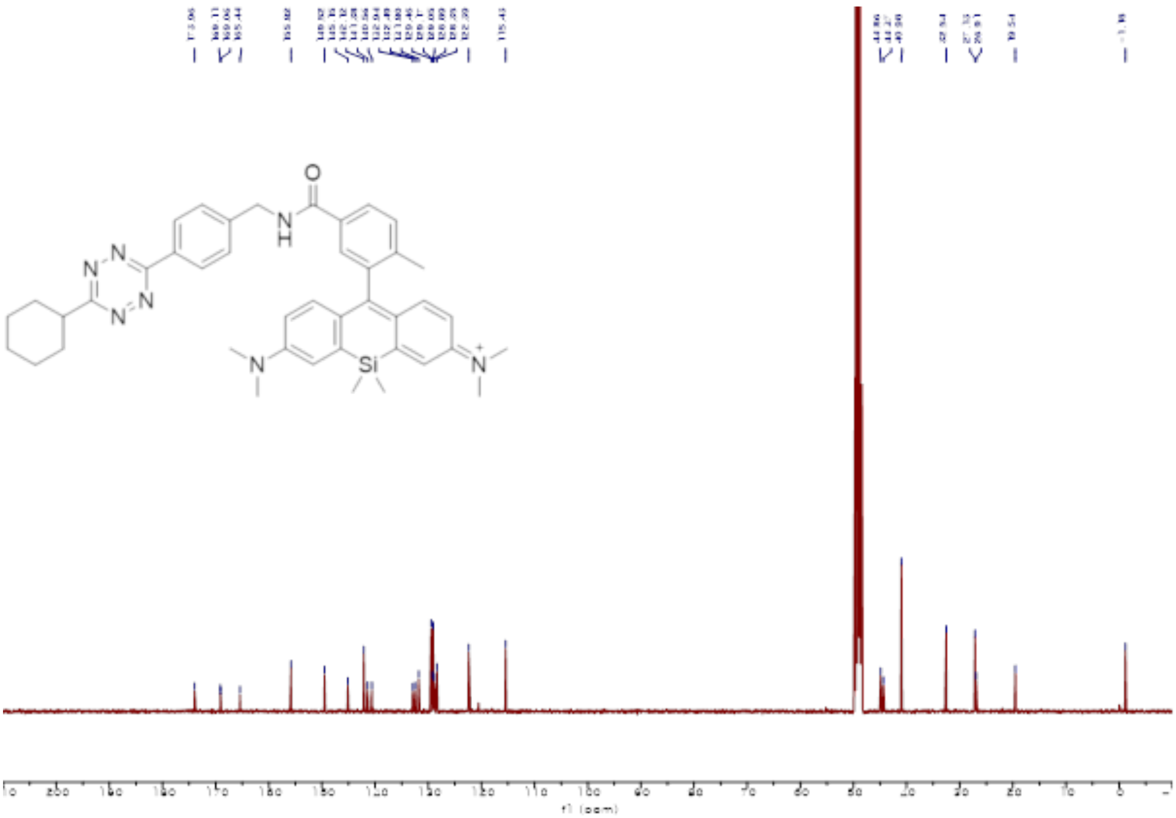

^{1}H NMR of **SiR-Tz14**

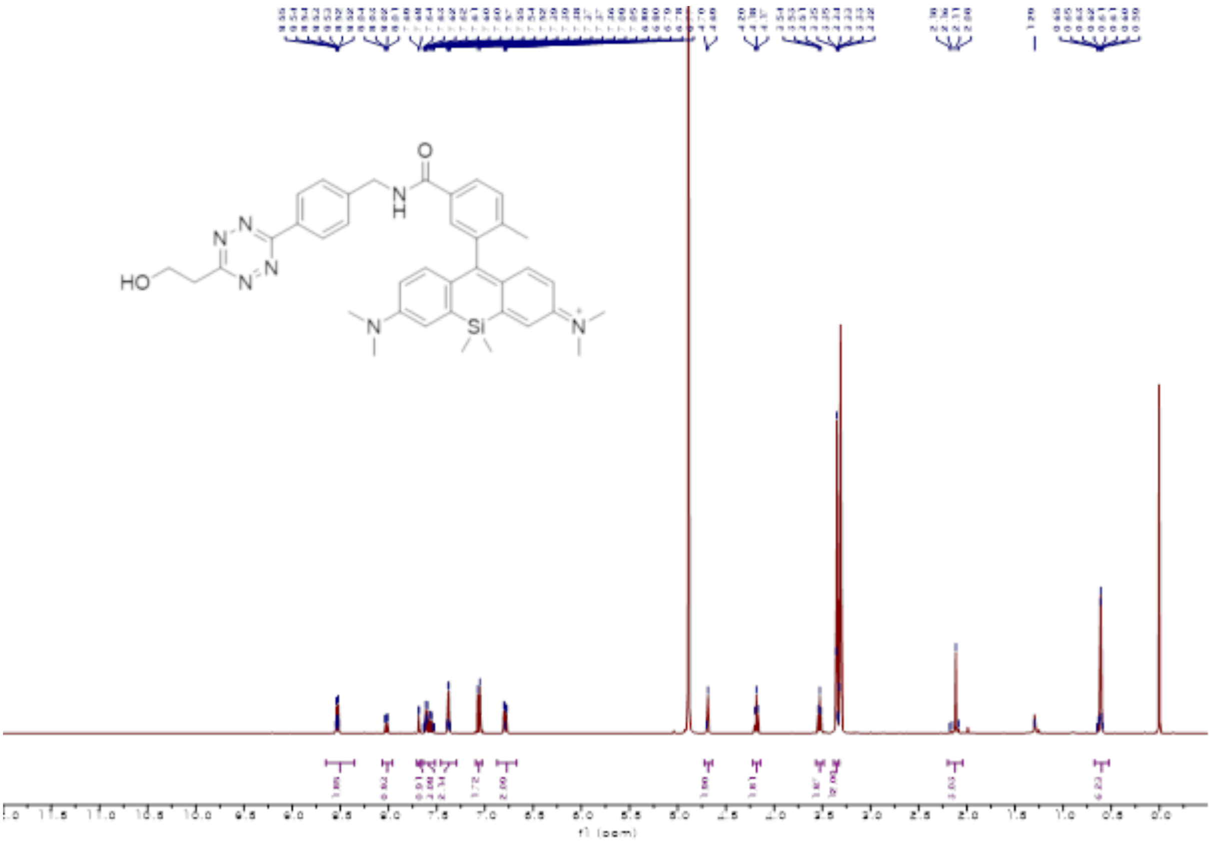

^{13}C NMR of **SiR-Tz14**

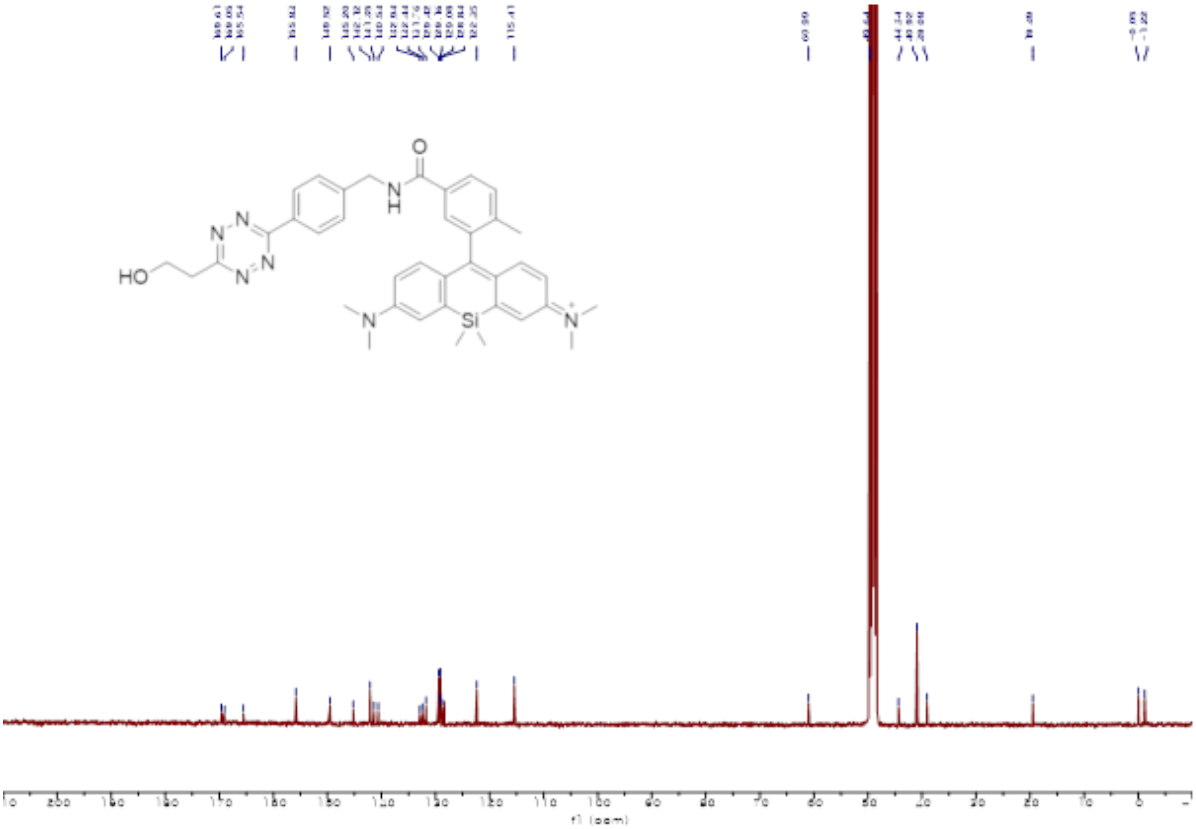

^{1}H NMR of **SiR-Tz15**

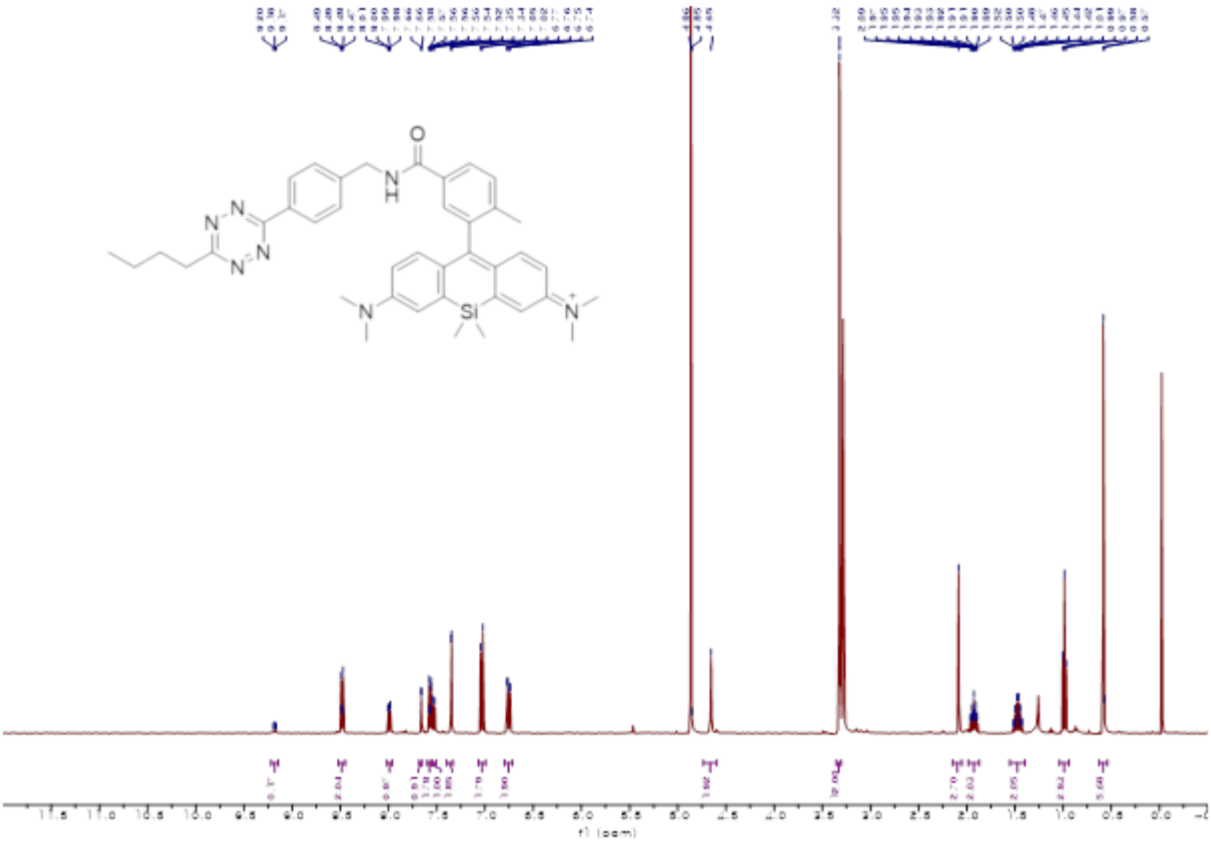

^{13}C NMR of **SiR-Tz15**

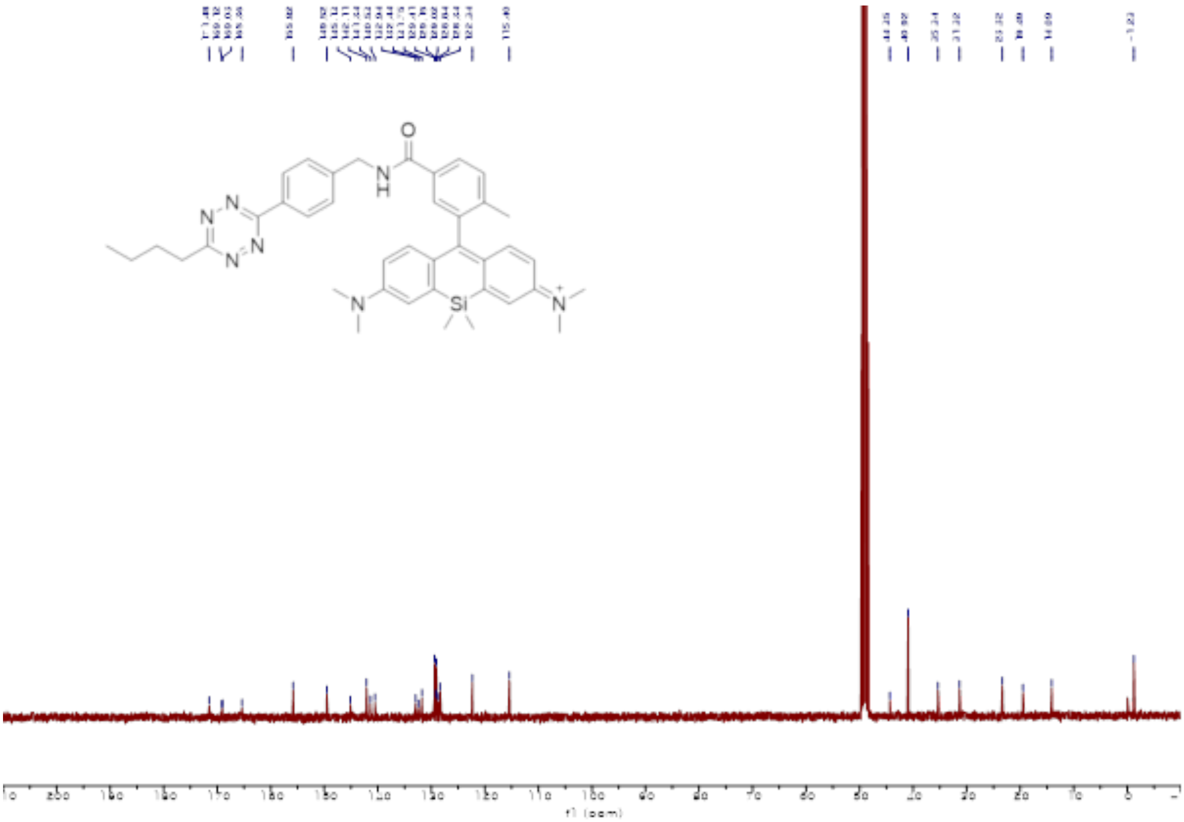

^{1}H NMR of **SiR-Tz16**

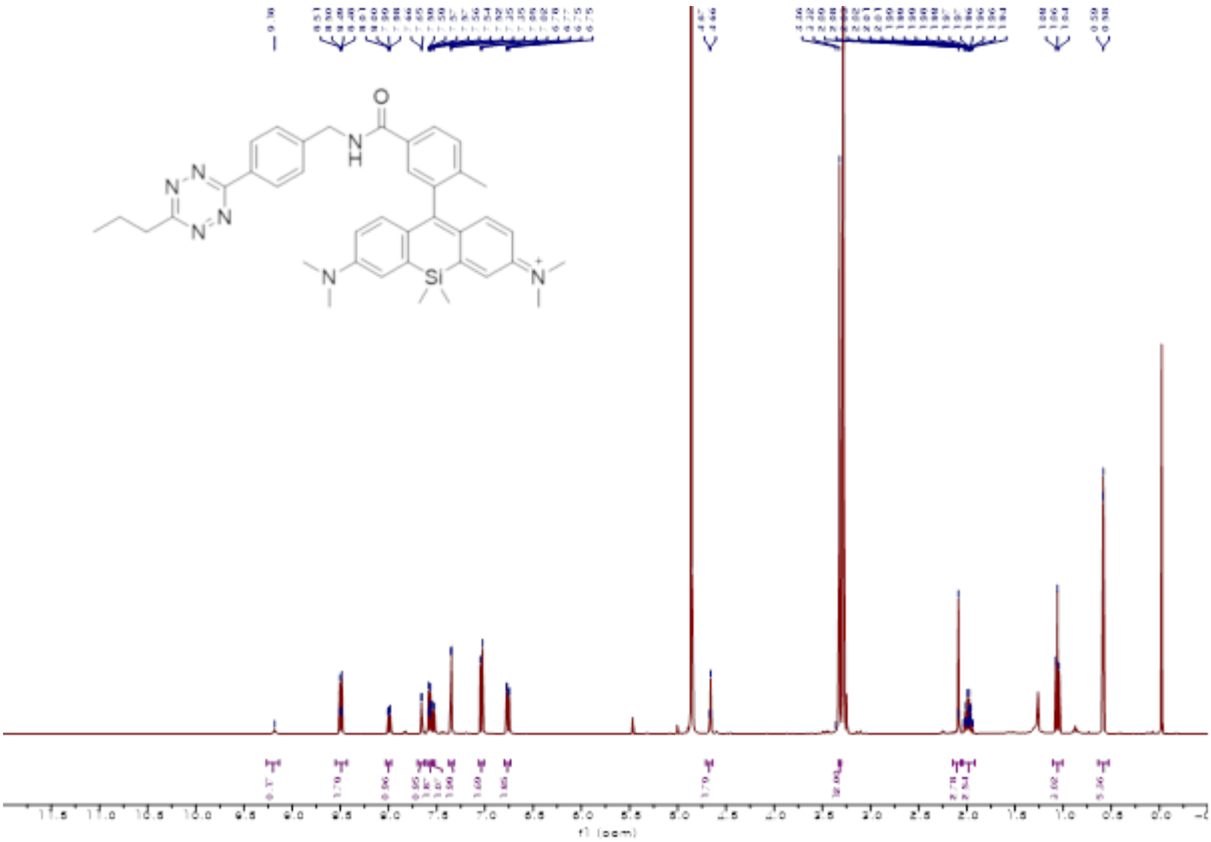

^{13}C NMR of **SiR-Tz16**

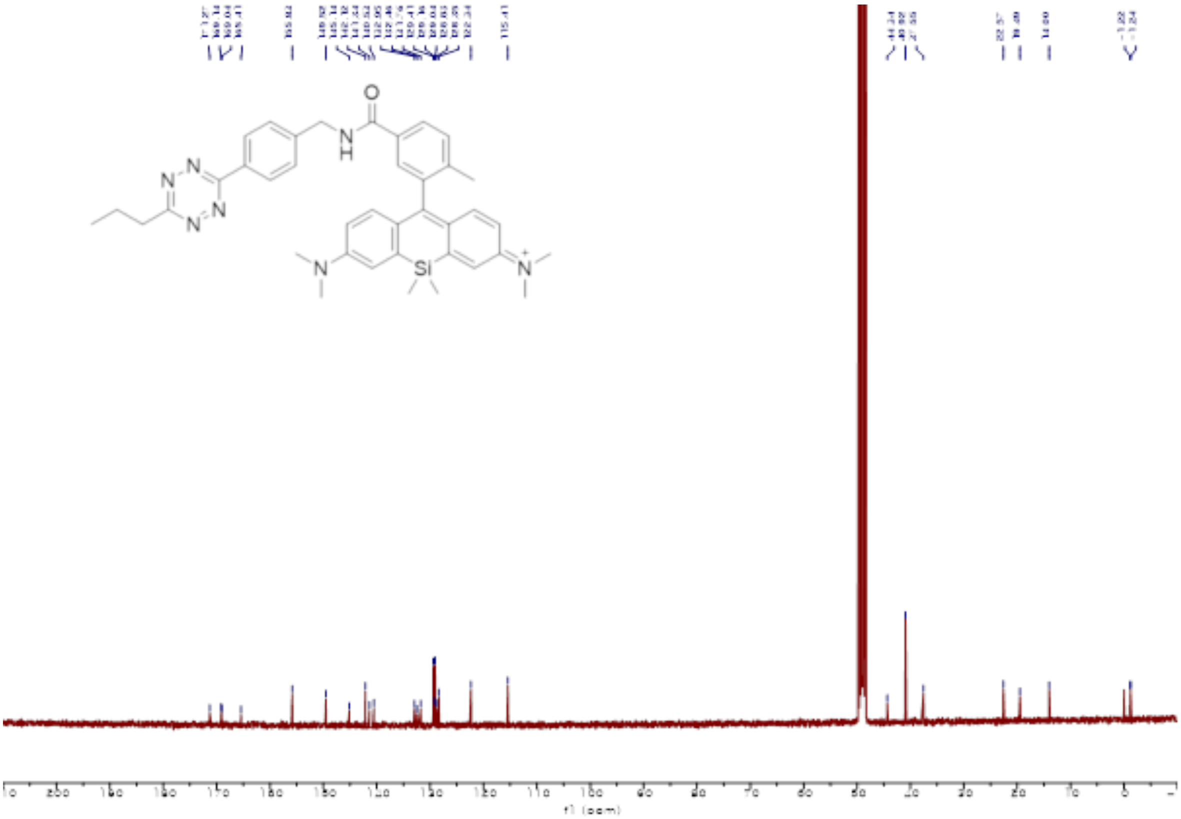

^{1}H NMR of **SiR-Tz17**

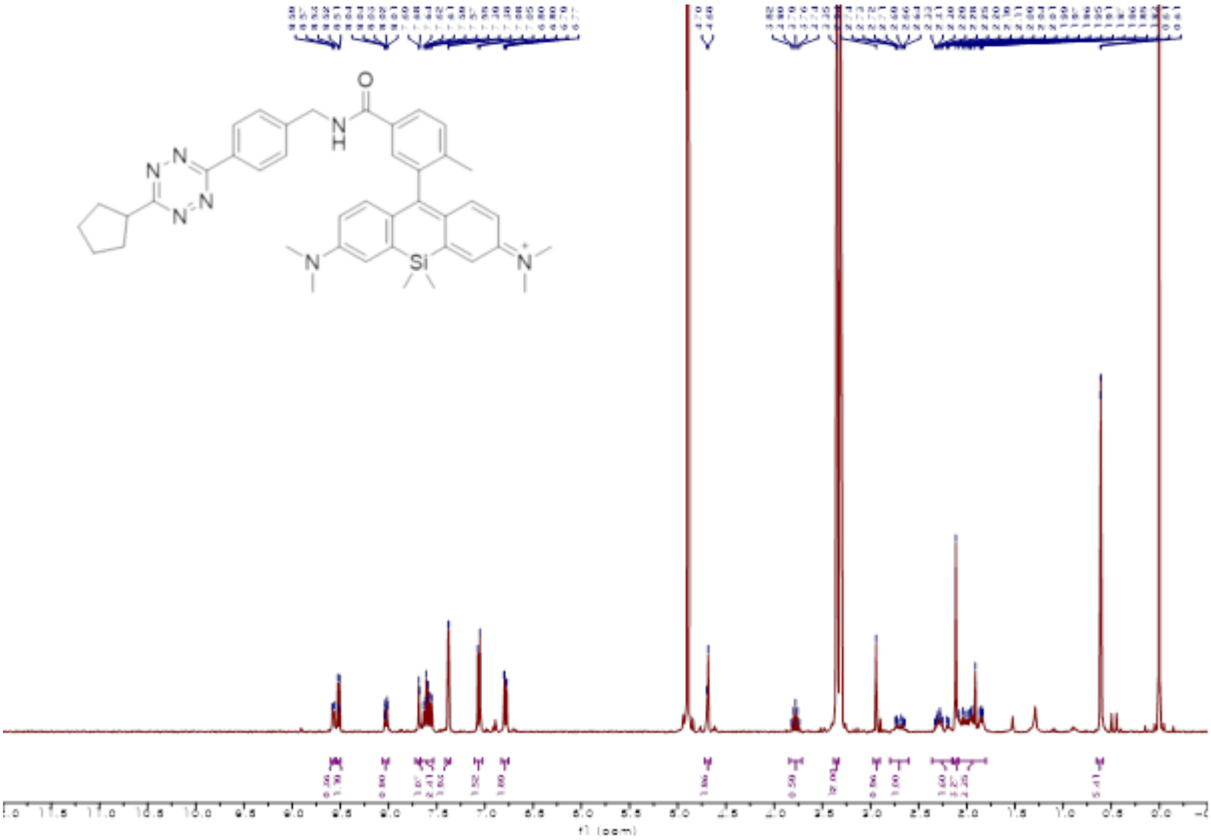

^{13}C NMR of **SiR-Tz17**

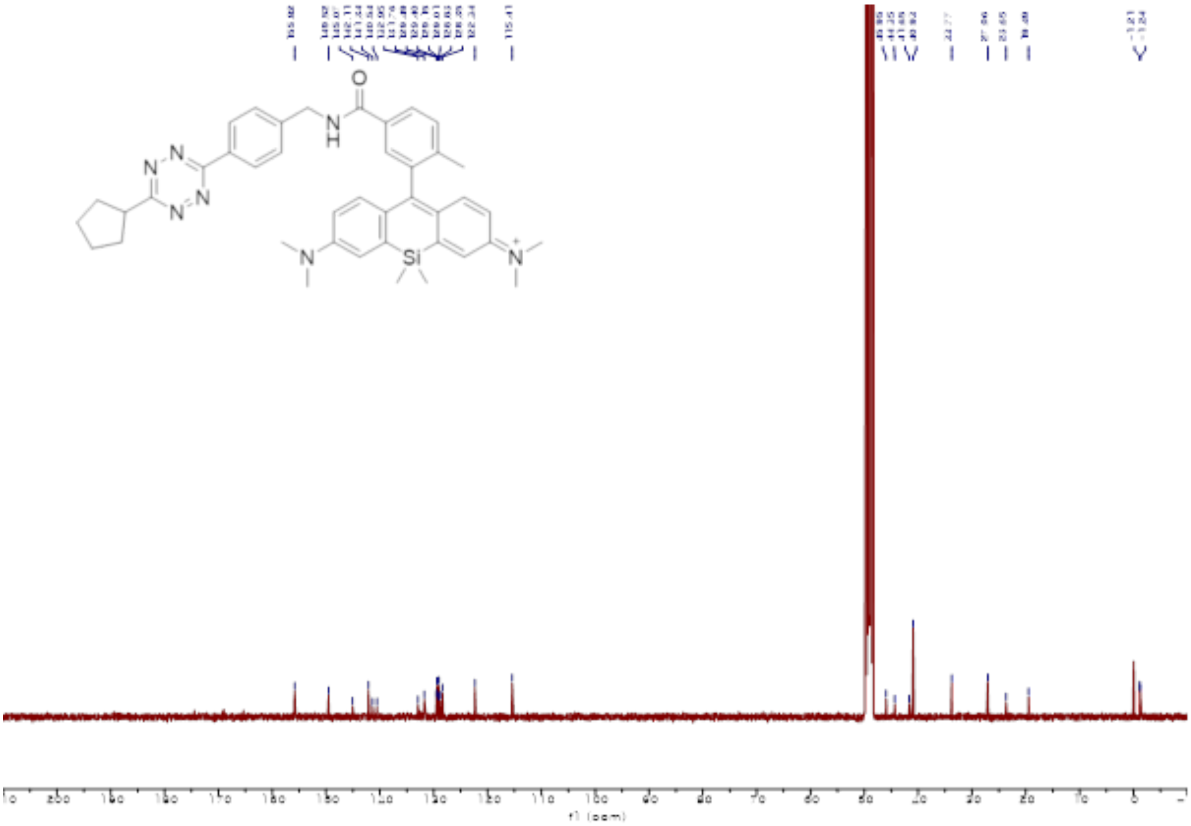

^{1}H NMR of **SiR-Tz18**

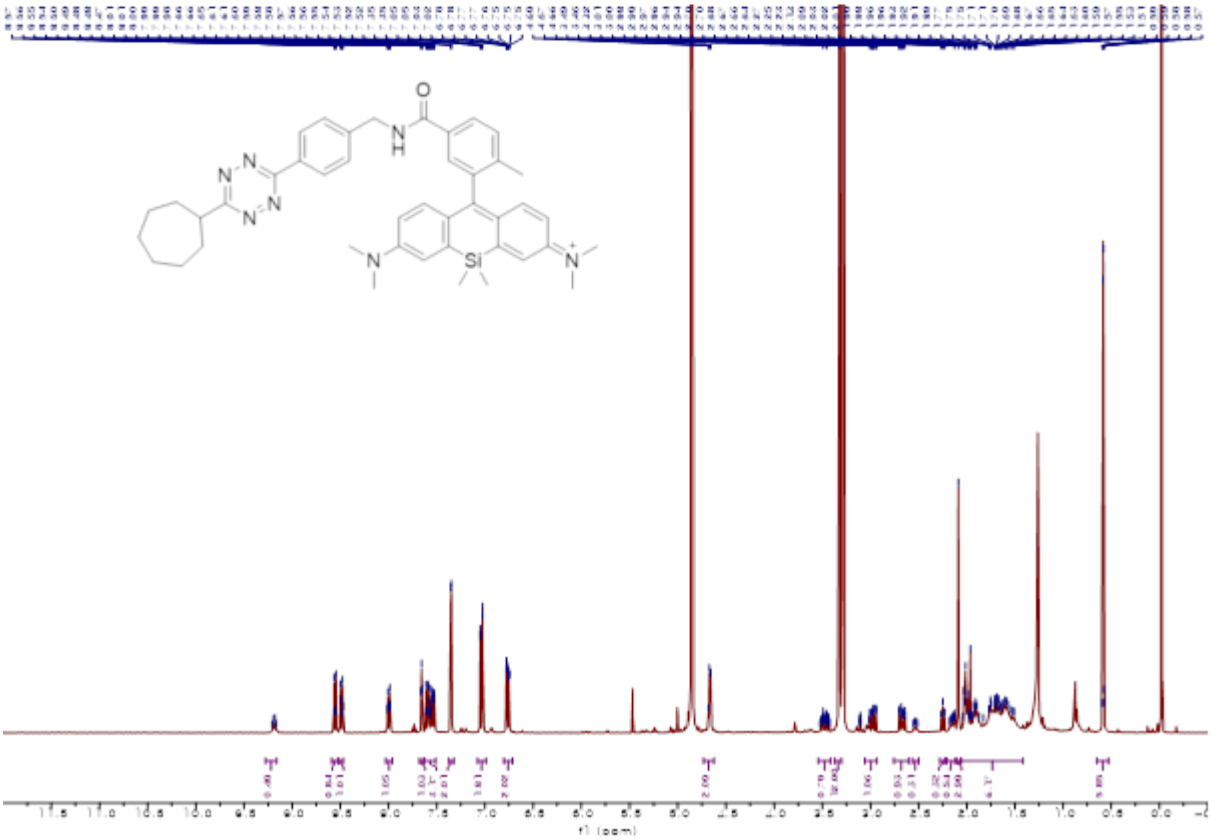

^{13}C NMR of **SiR-Tz18**

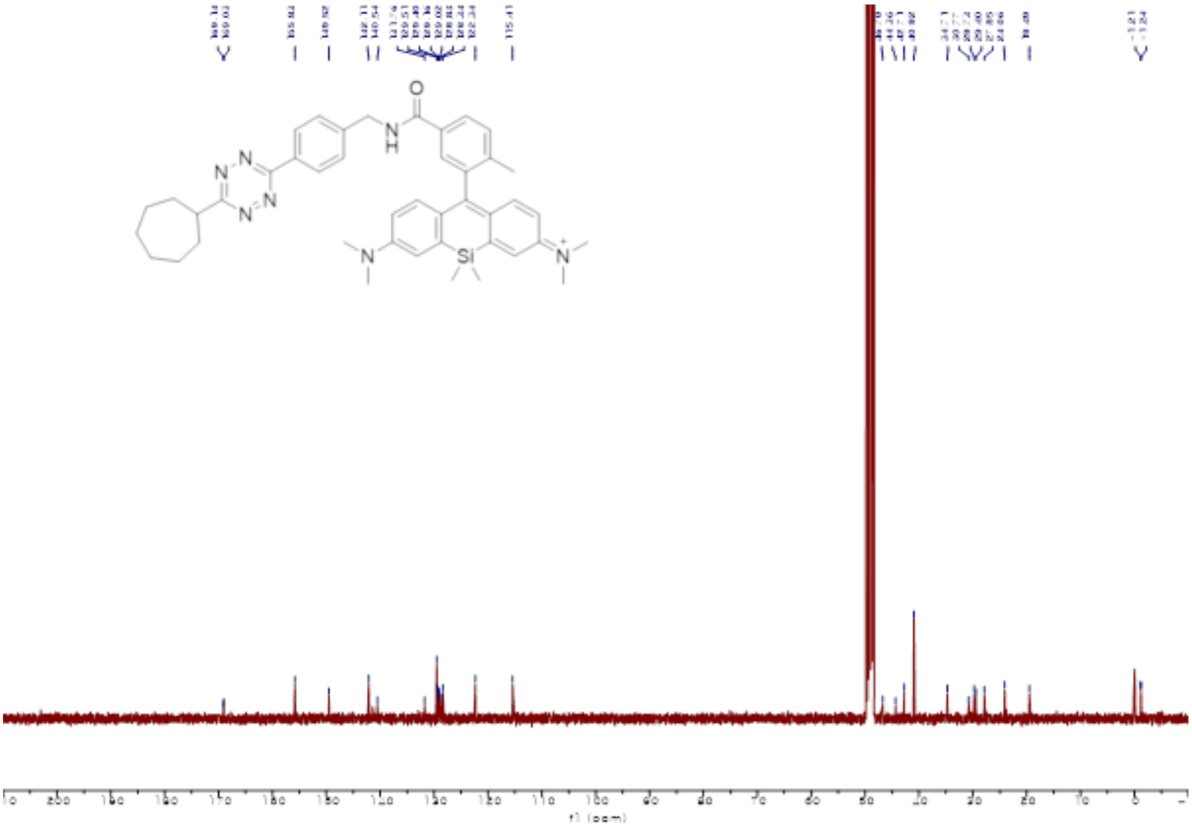

^{1}H NMR of **SiR-Tz19**

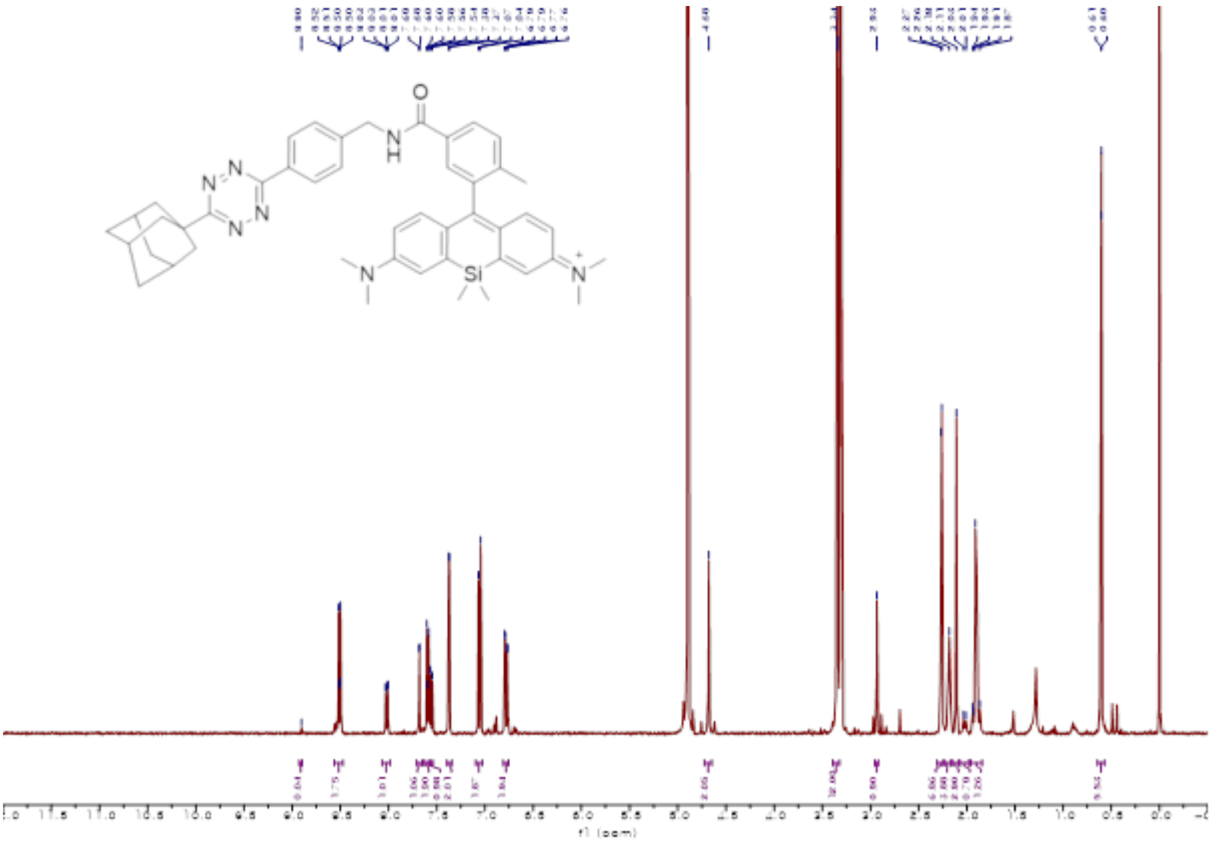

^{13}C NMR of **SiR-Tz19**

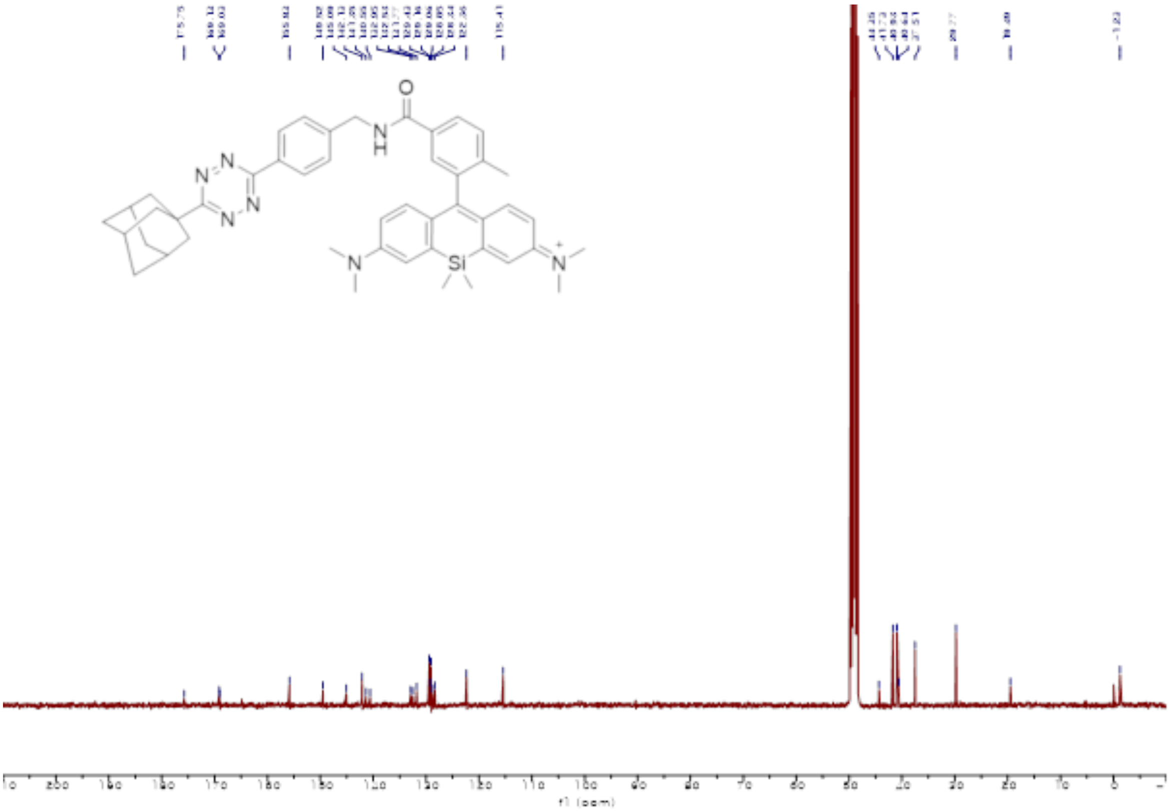

^{1}H NMR of **SiR-Tz20**

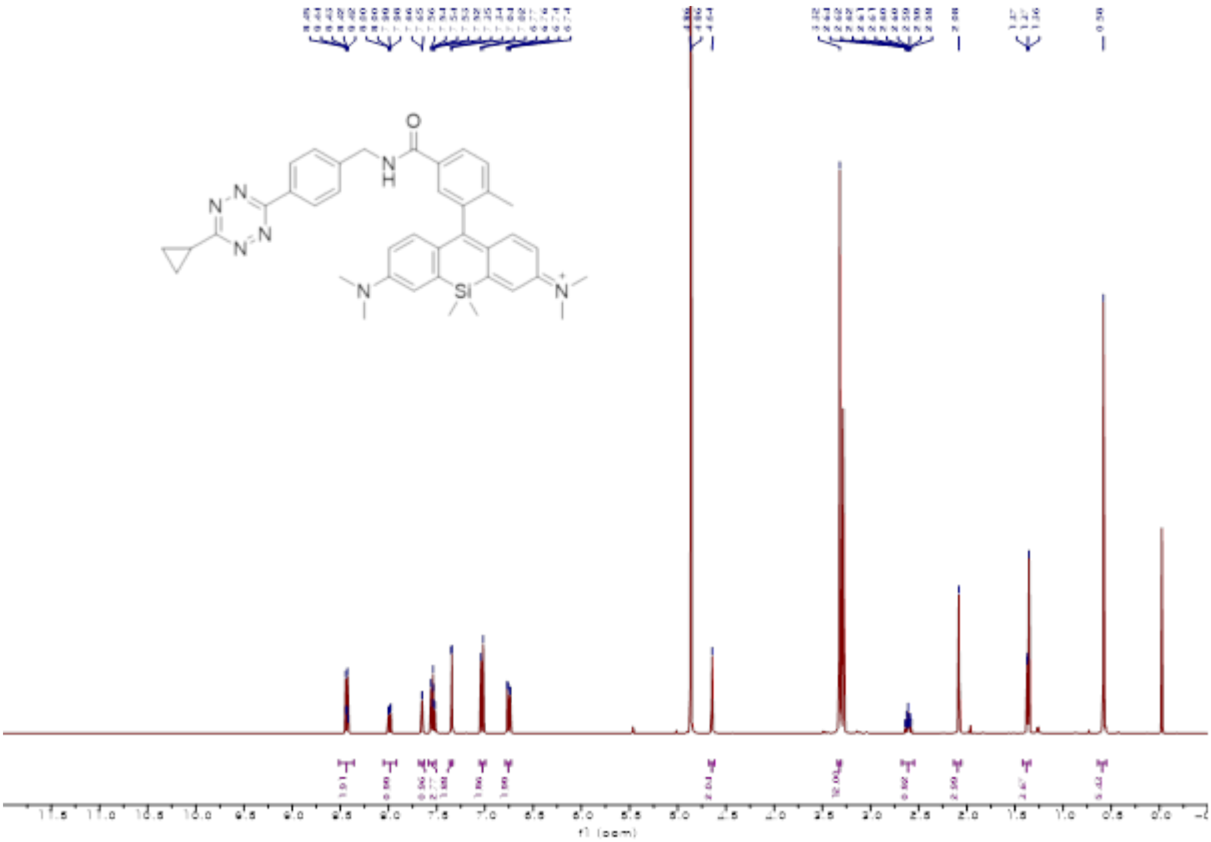

^{13}C NMR of **SiR-Tz20**

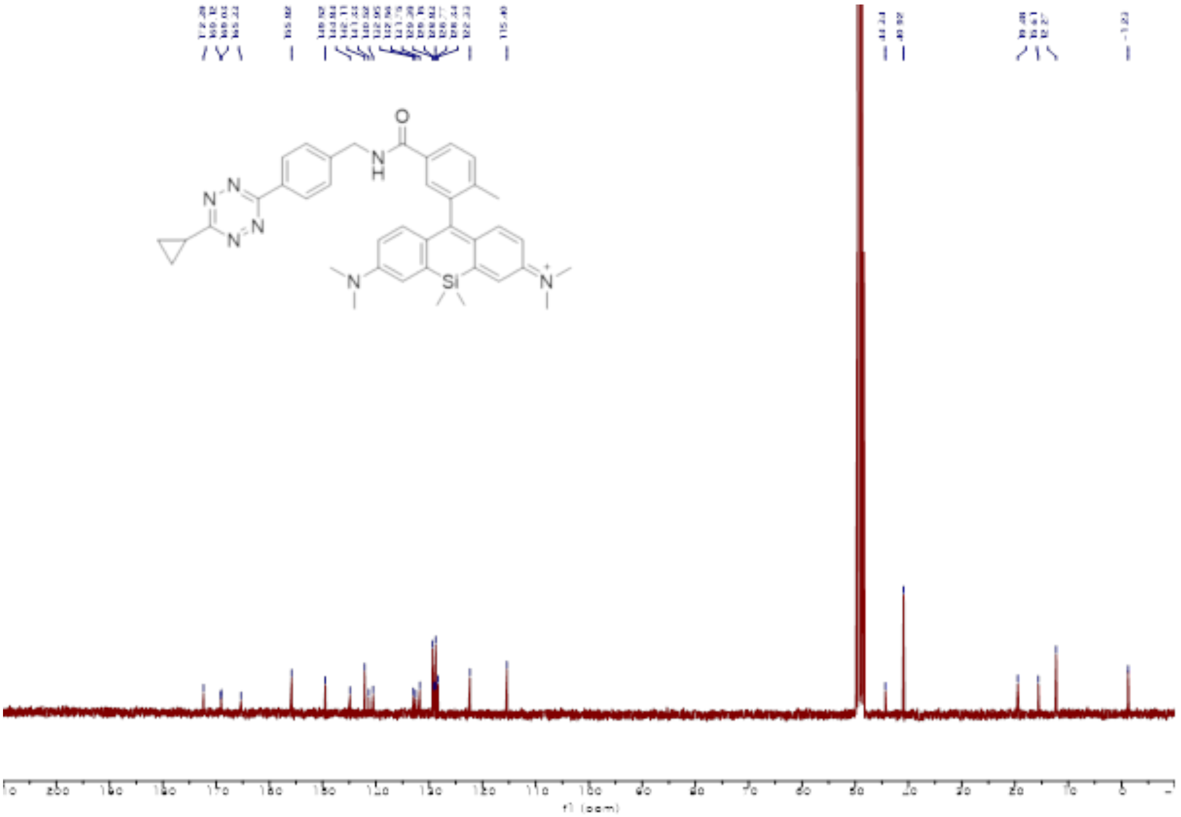

HR-MS(ESI) of **SiR-Tz20**

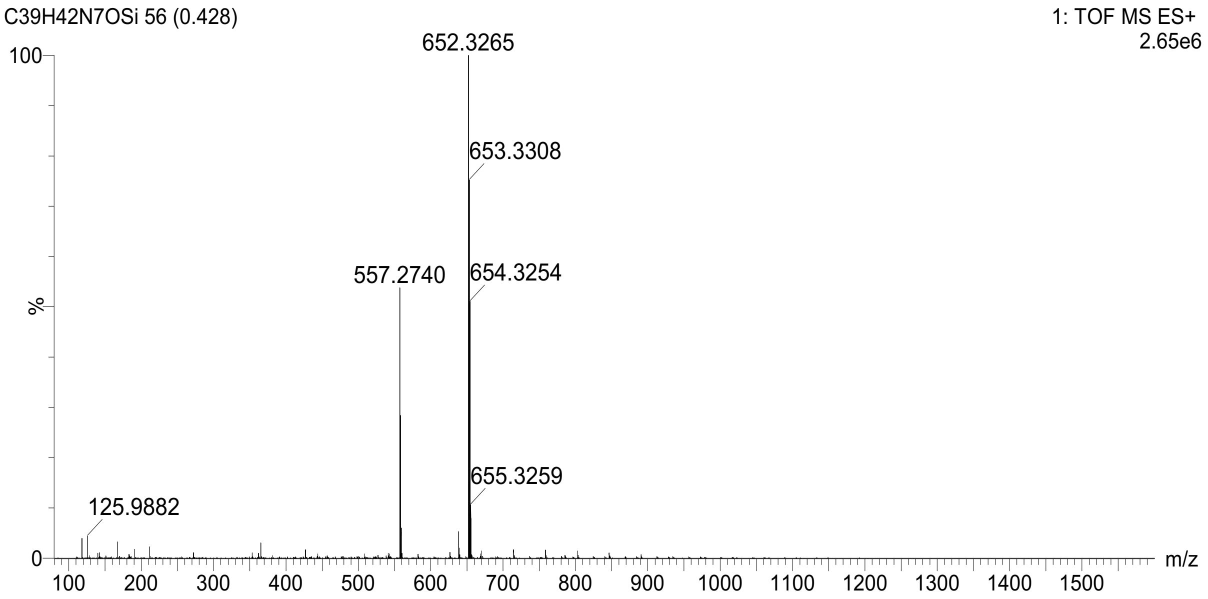

^{1}H NMR of **SiR-Tz21**

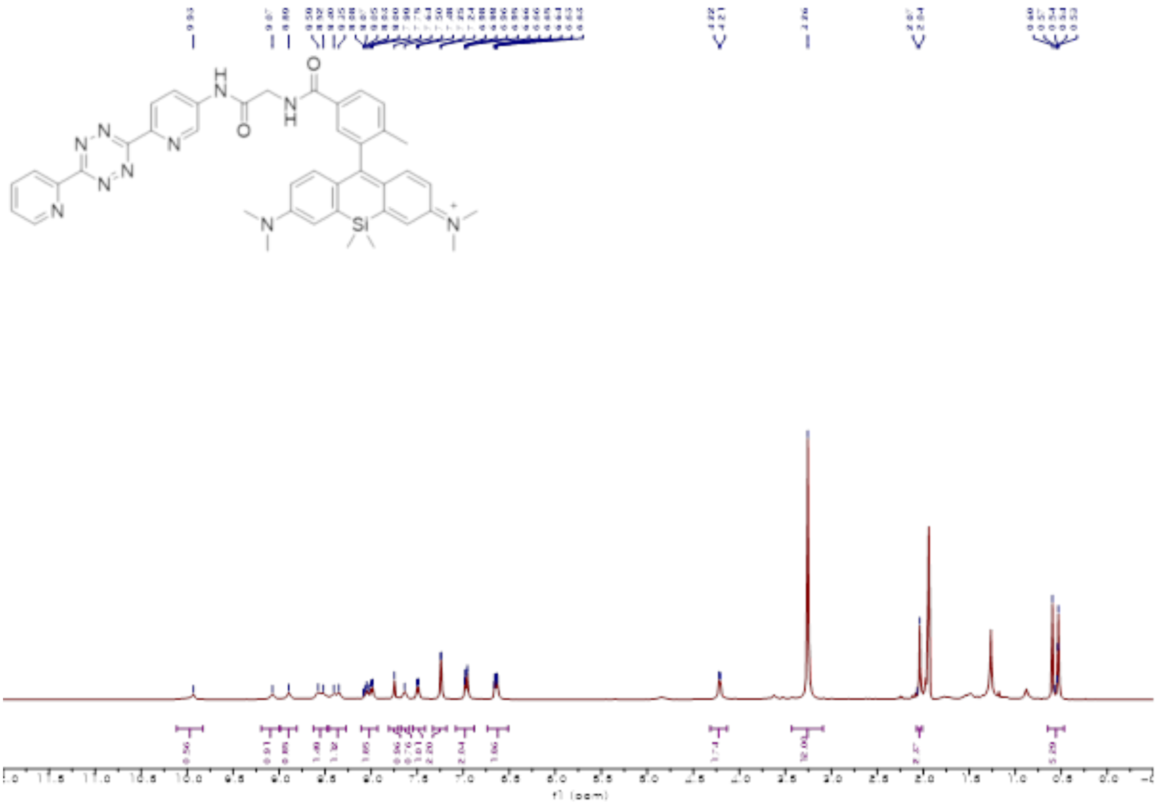

^{13}C NMR of **SiR-Tz21**

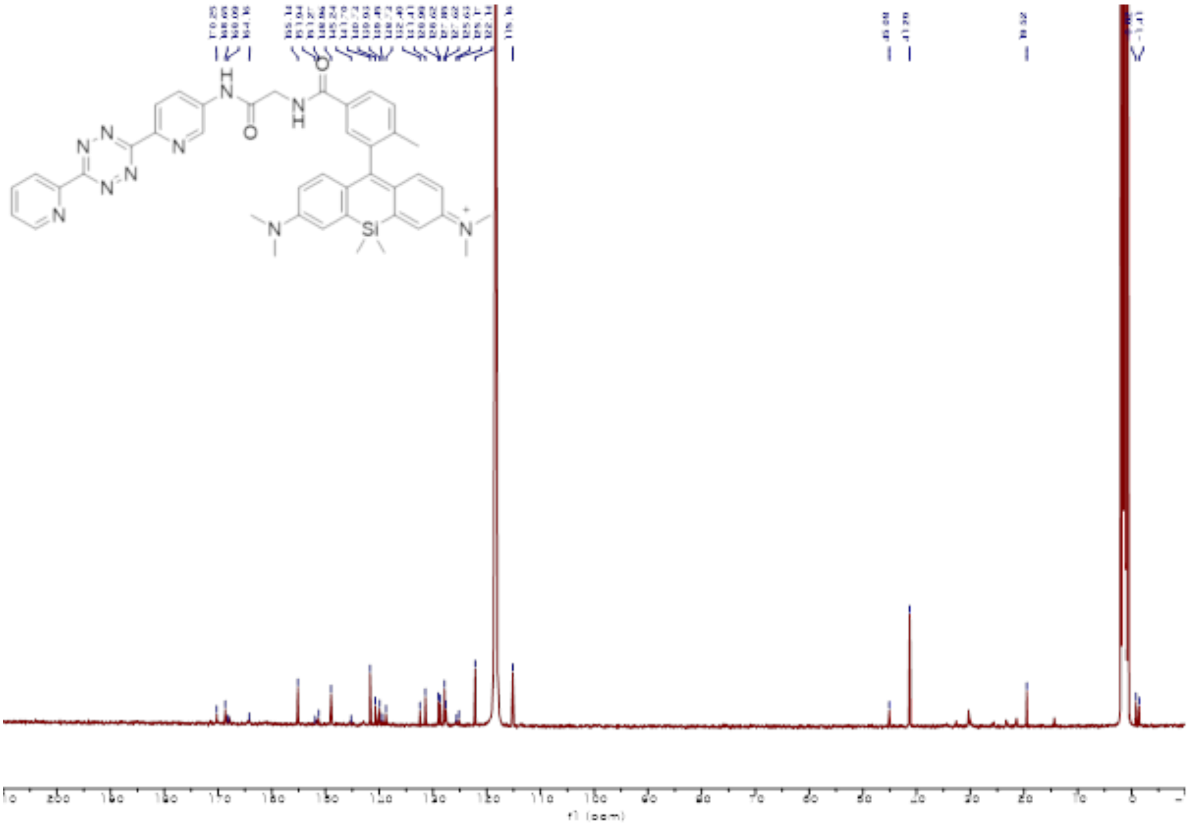

^{1}H NMR of **SiR-Tz22**

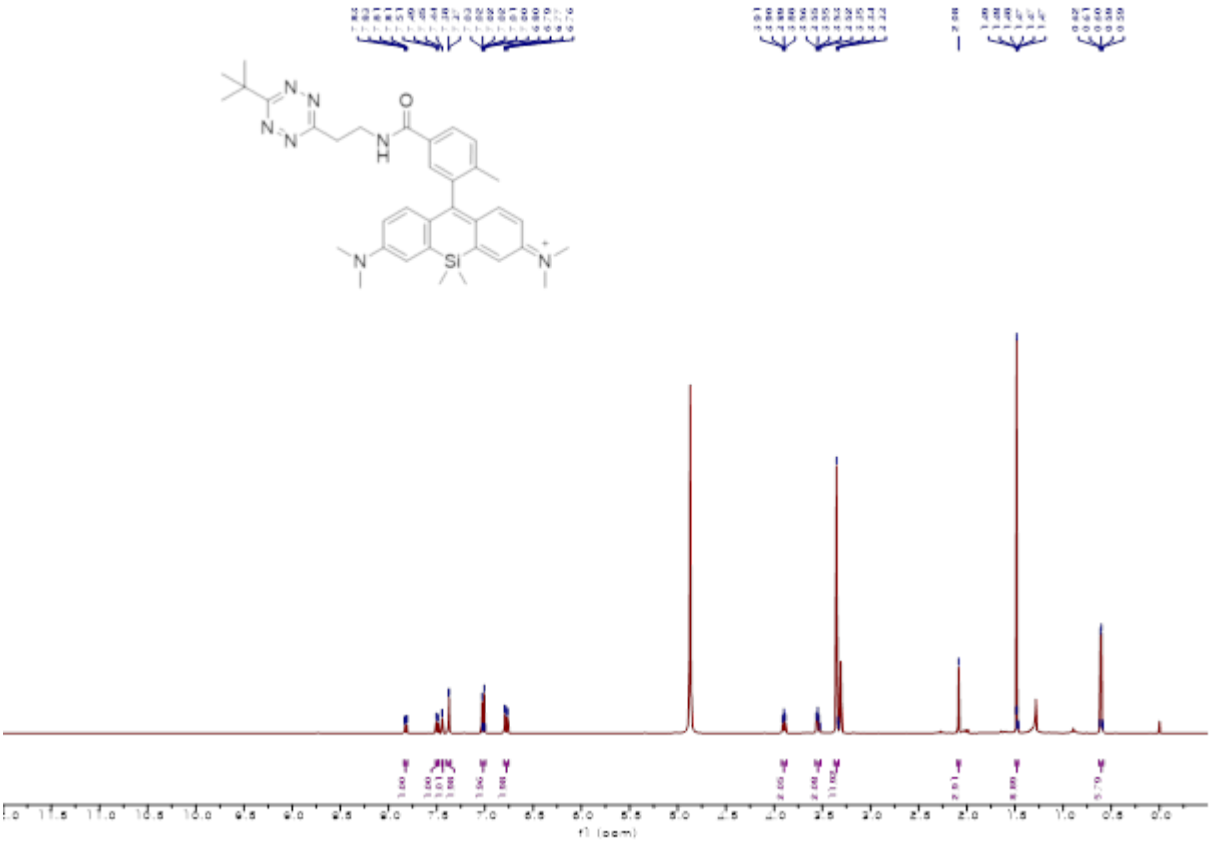

^{13}C NMR of **SiR-Tz22**

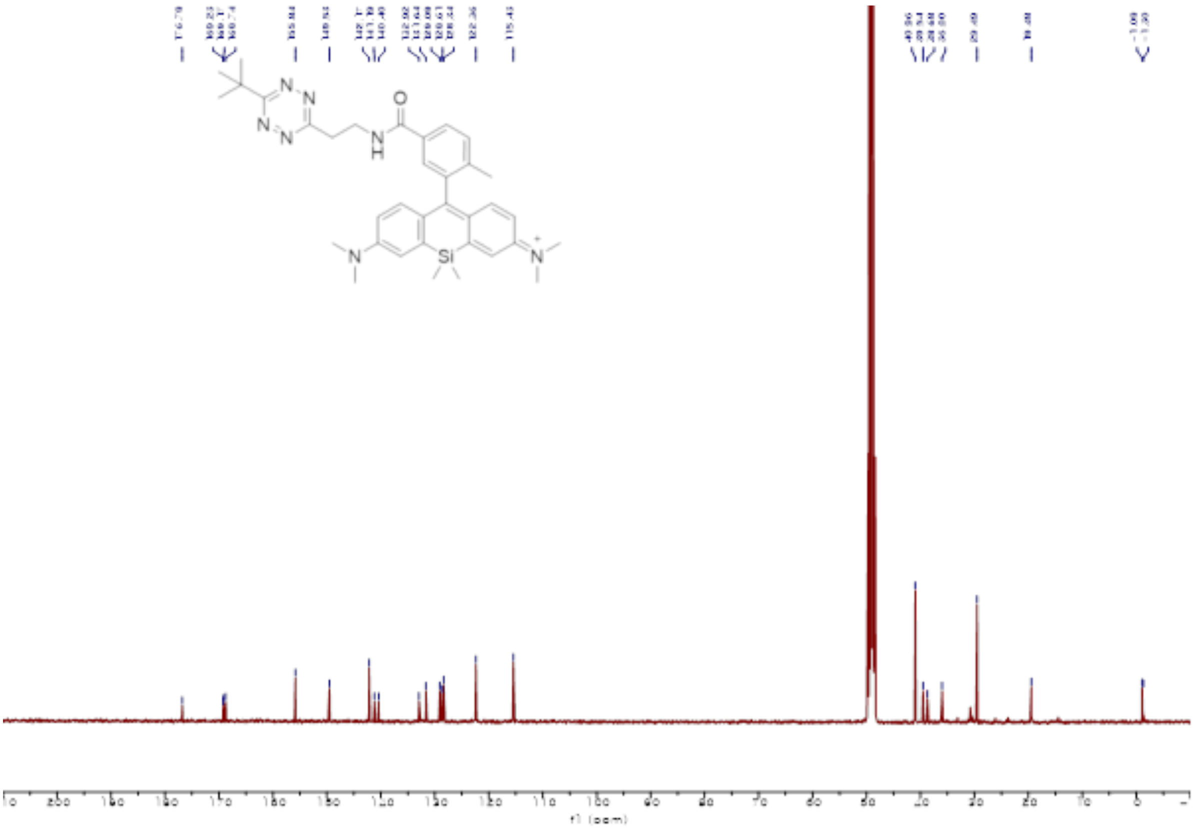

^{1}H NMR of **SiR-Tz23**

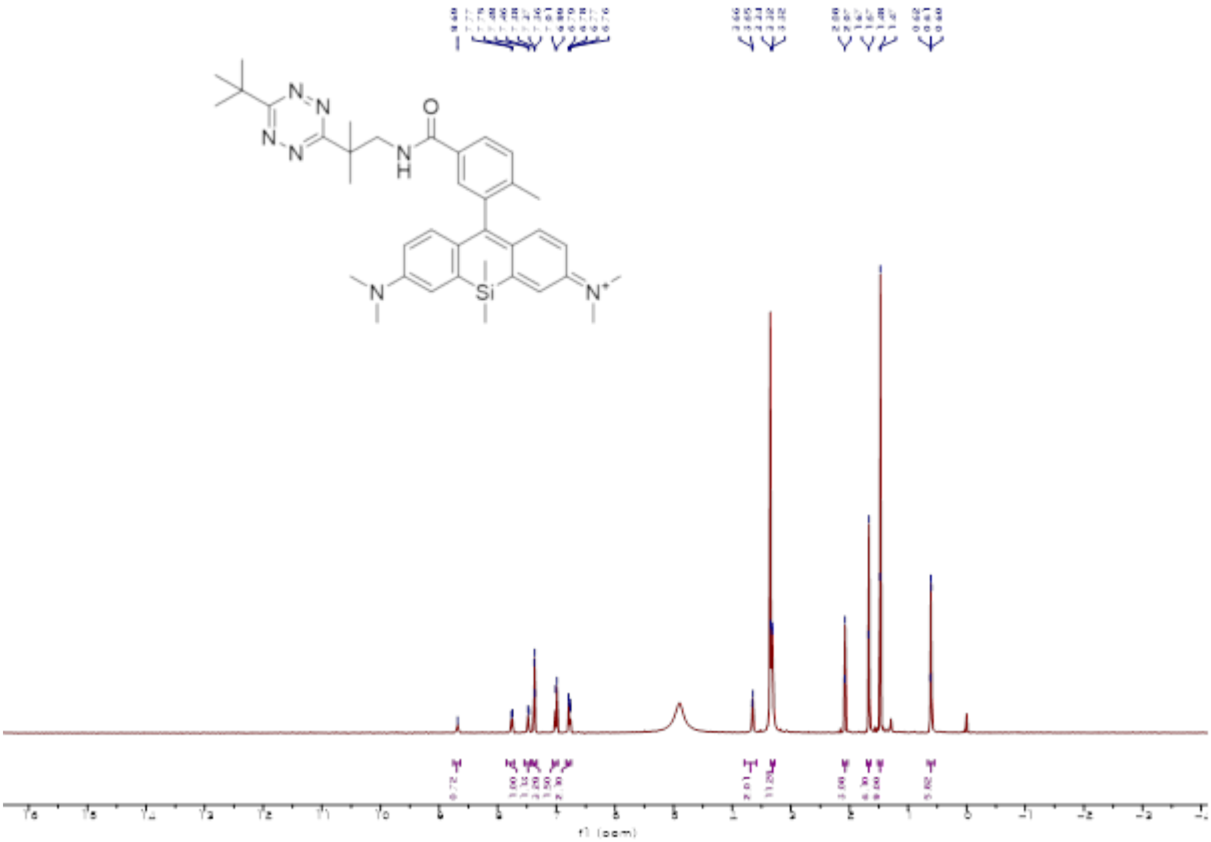

^{13}C NMR of **SiR-Tz23**

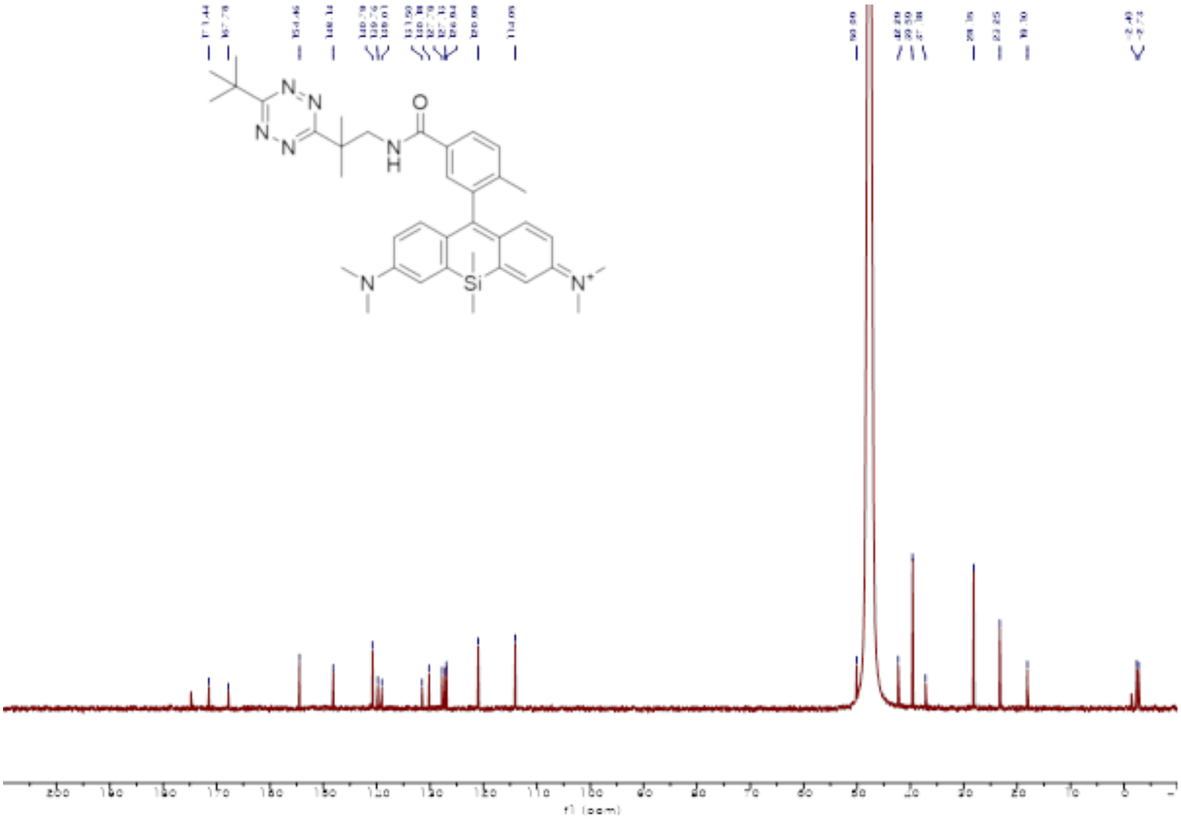

^{1}H NMR of **SiR-Bn**

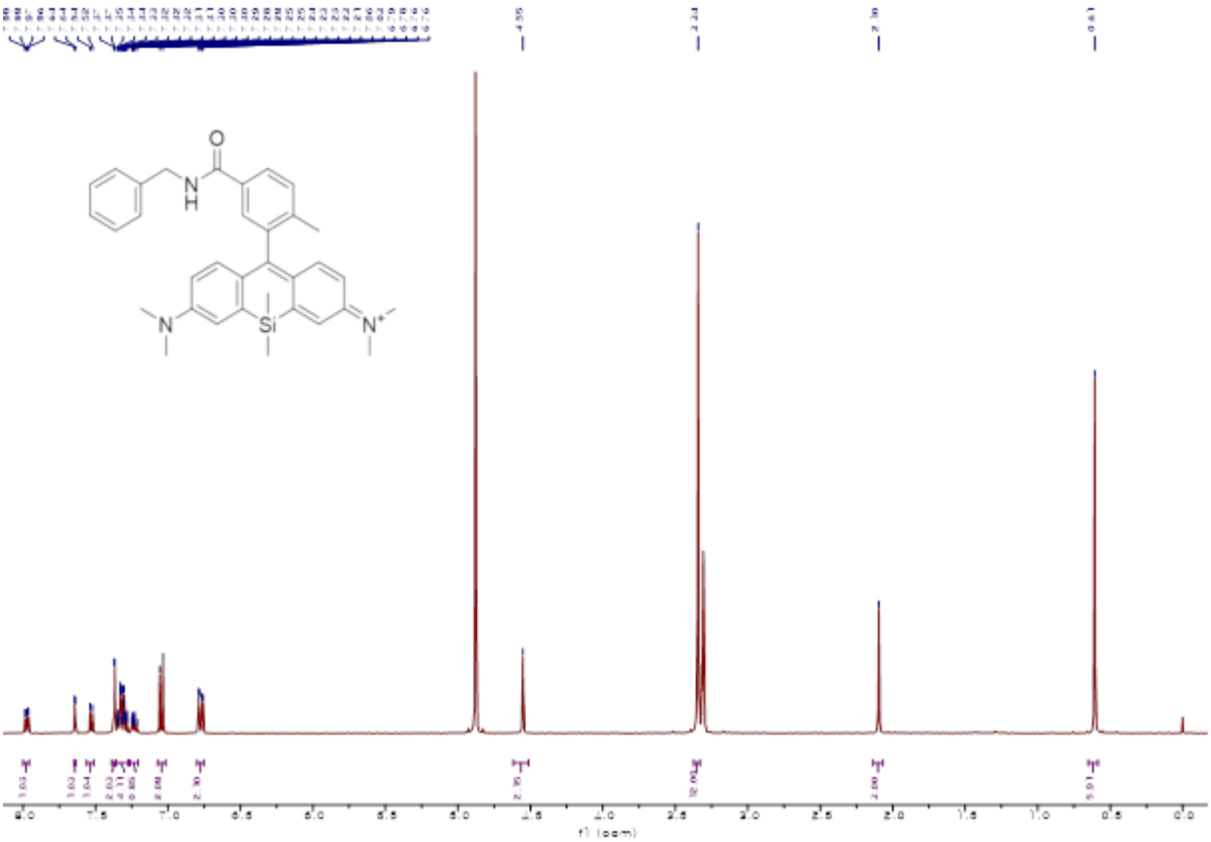

^{13}C NMR of **SiR-Bn**

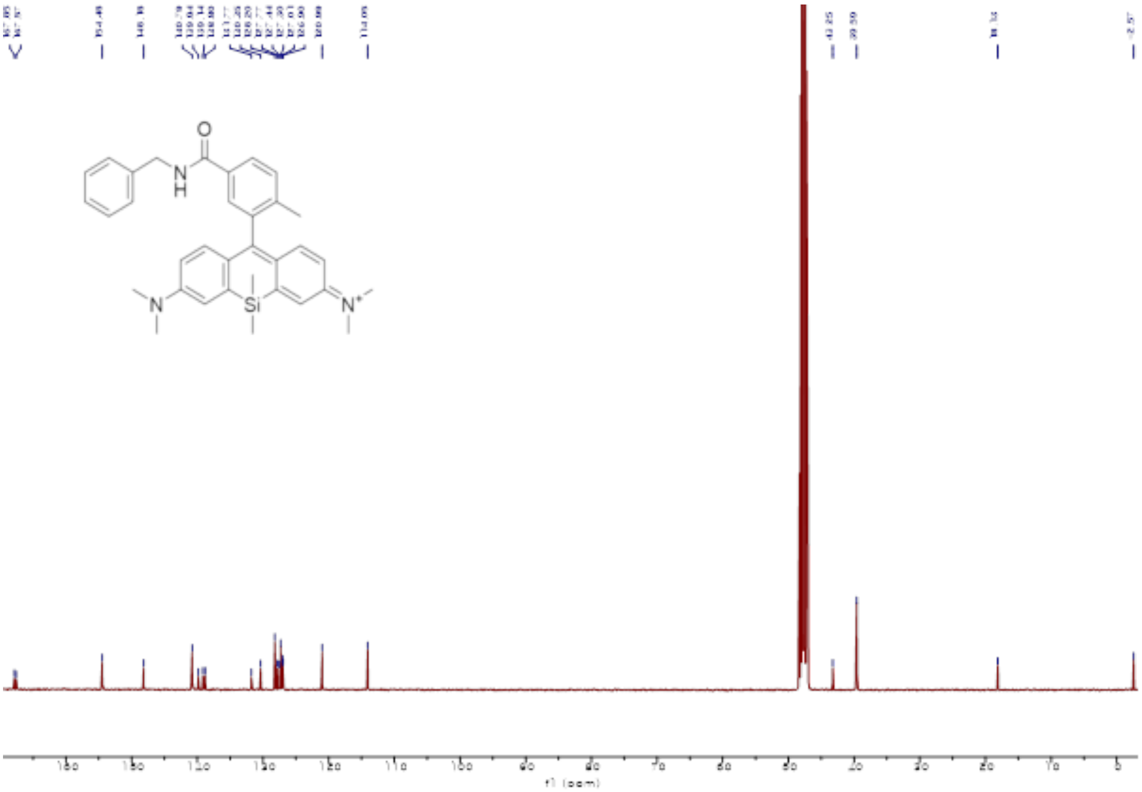

^{1}H and ^{13}C NMR of **I-2**

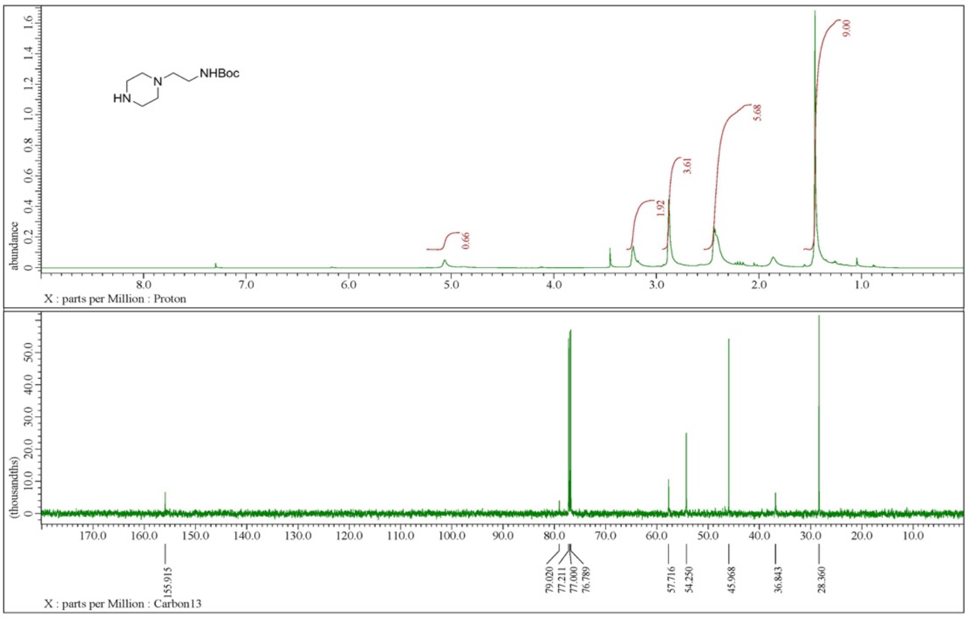

^{1}H and ^{13}C NMR of **I-3**

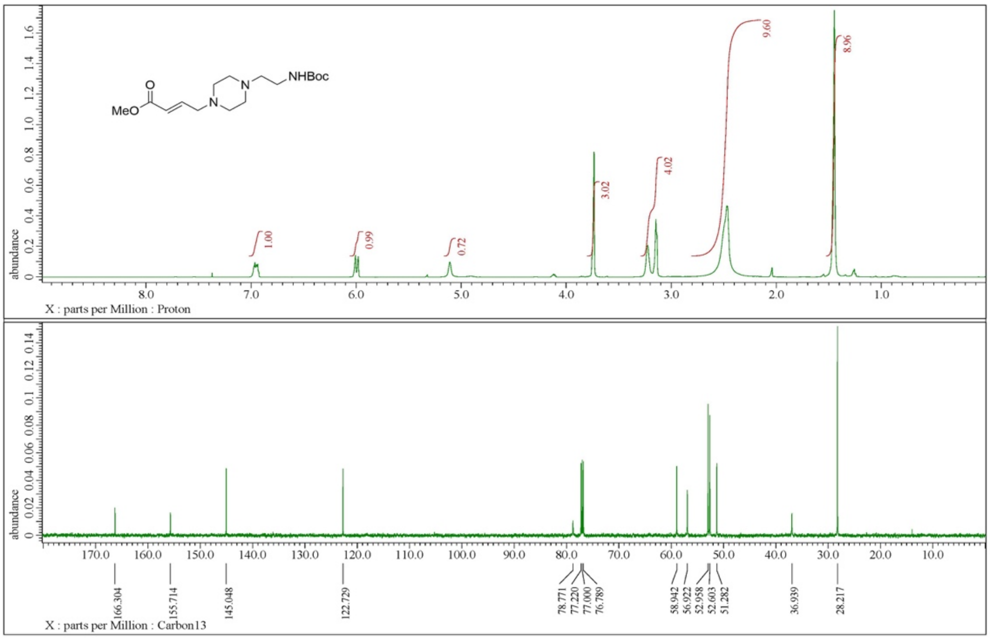

^{1}H and ^{13}C NMR of **I-4**

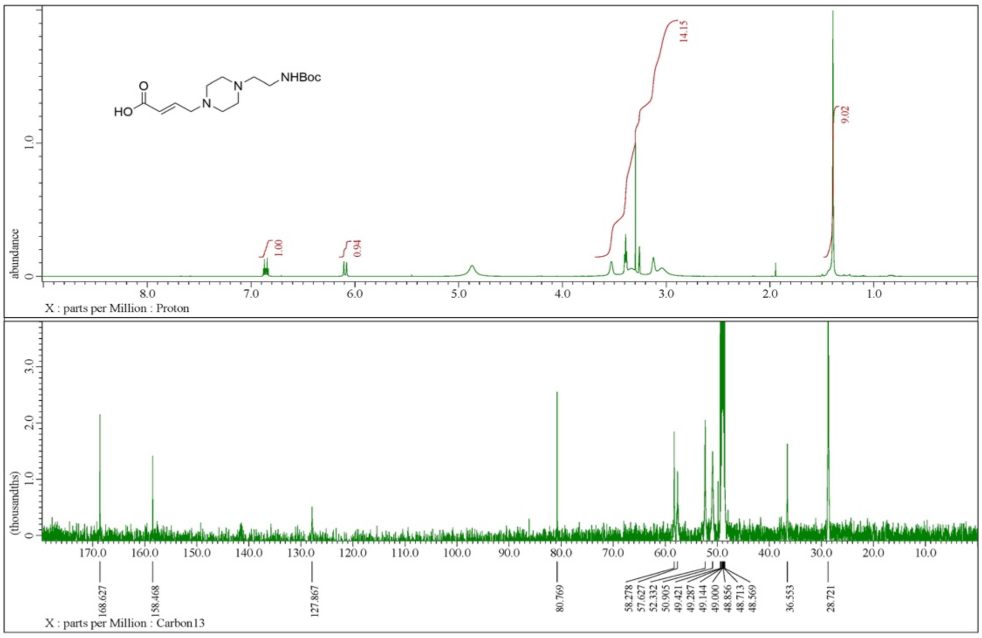

^{1}H and ^{13}C NMR of **I-5**

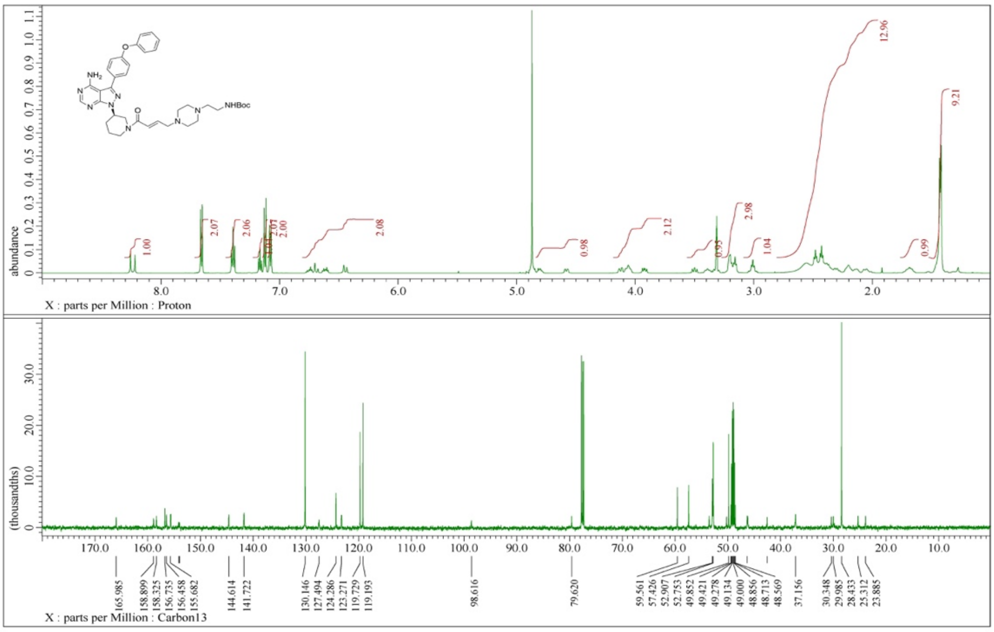

^{1}H and ^{13}C NMR of **I-6**

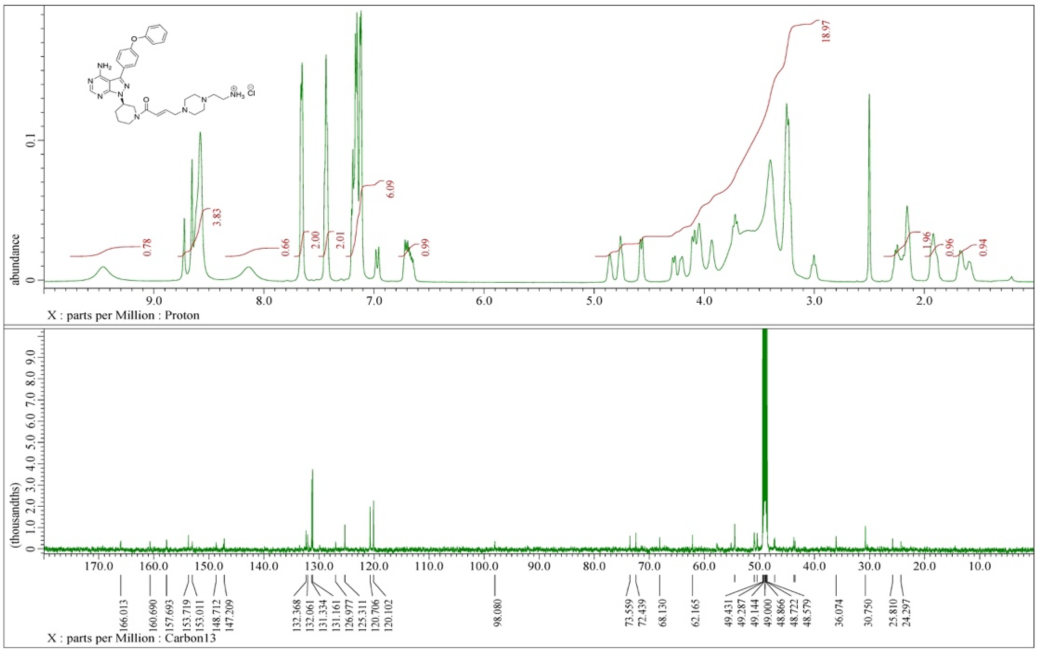

^{1}H and ^{13}C NMR of **IBR-TCO**

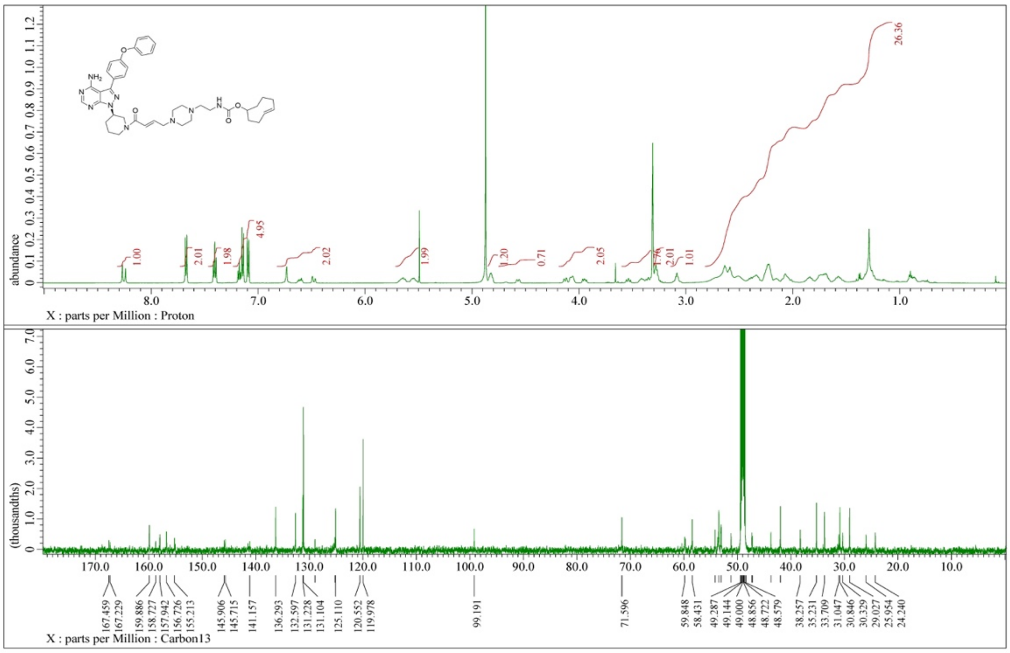

^{1}H NMR of **S-2**

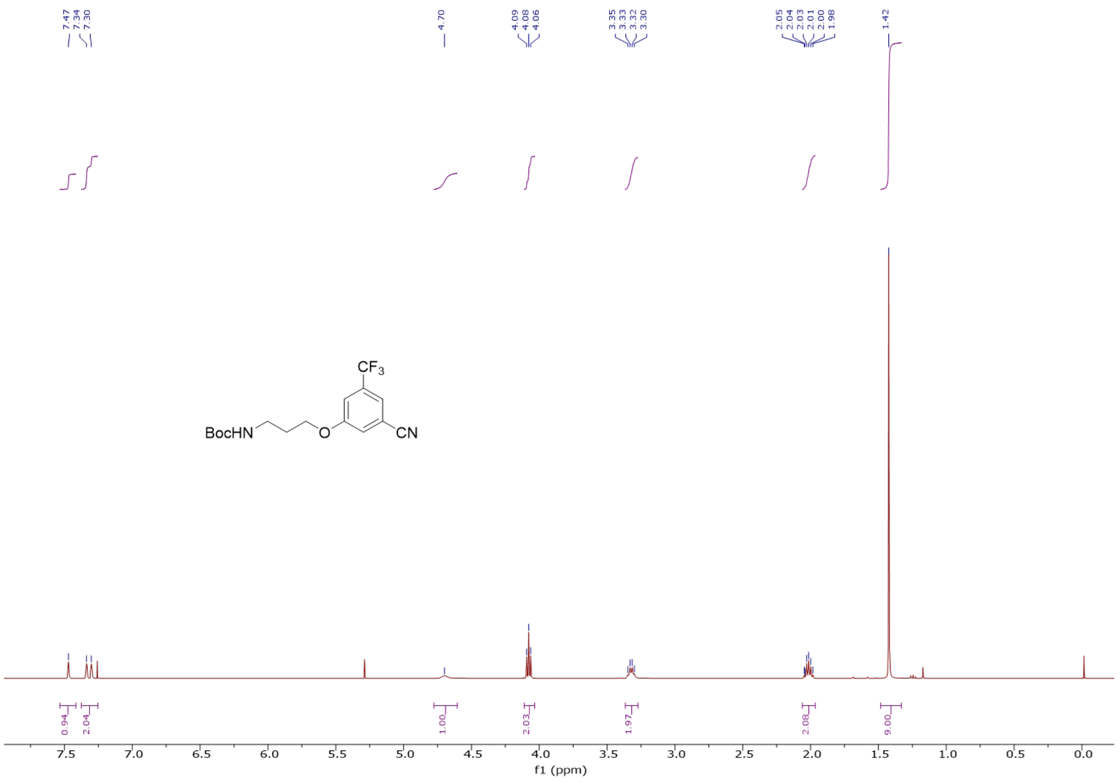

^{13}C NMR of **S-2**

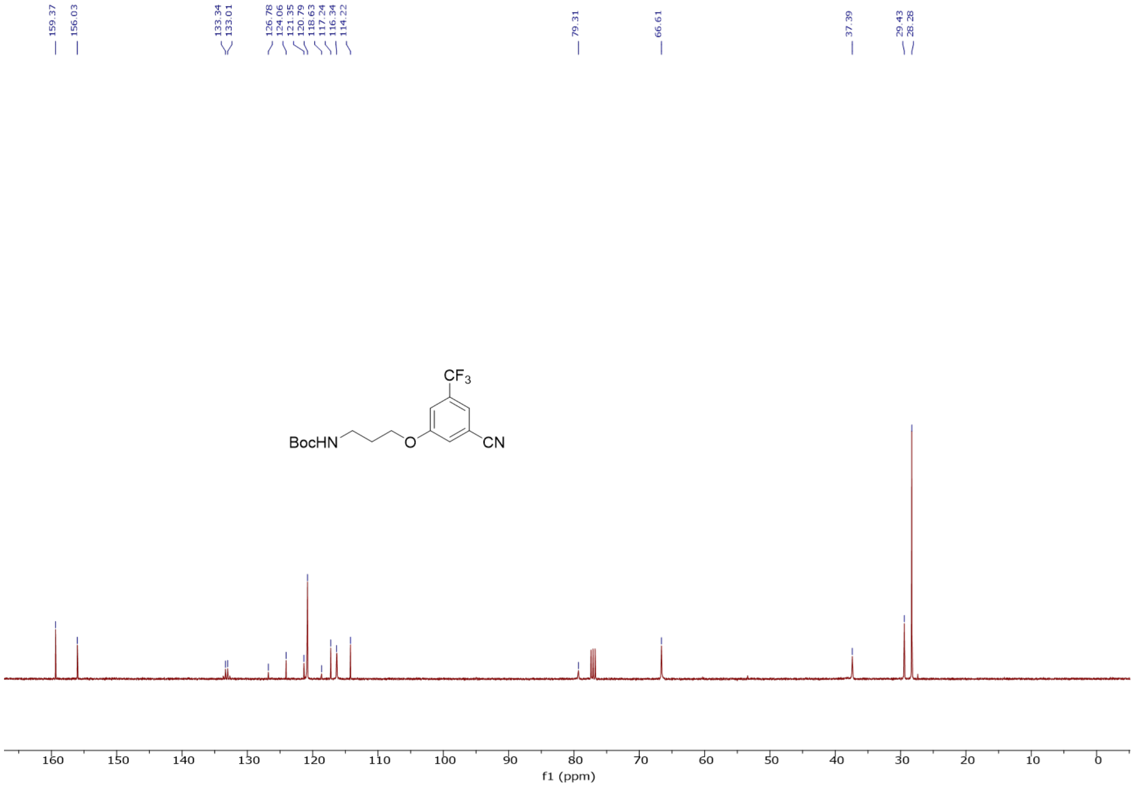

^{1}H NMR of **S-3**

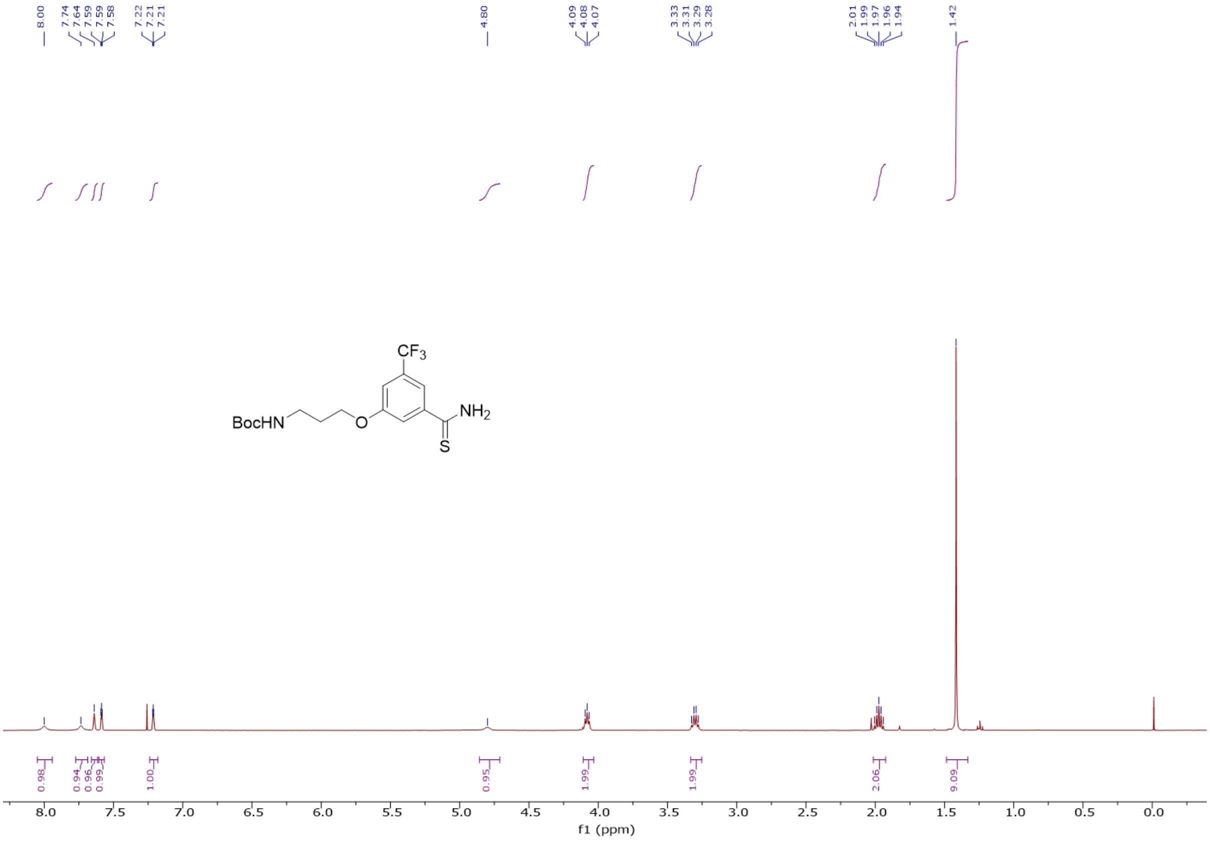

^{13}C NMR of **S-3**

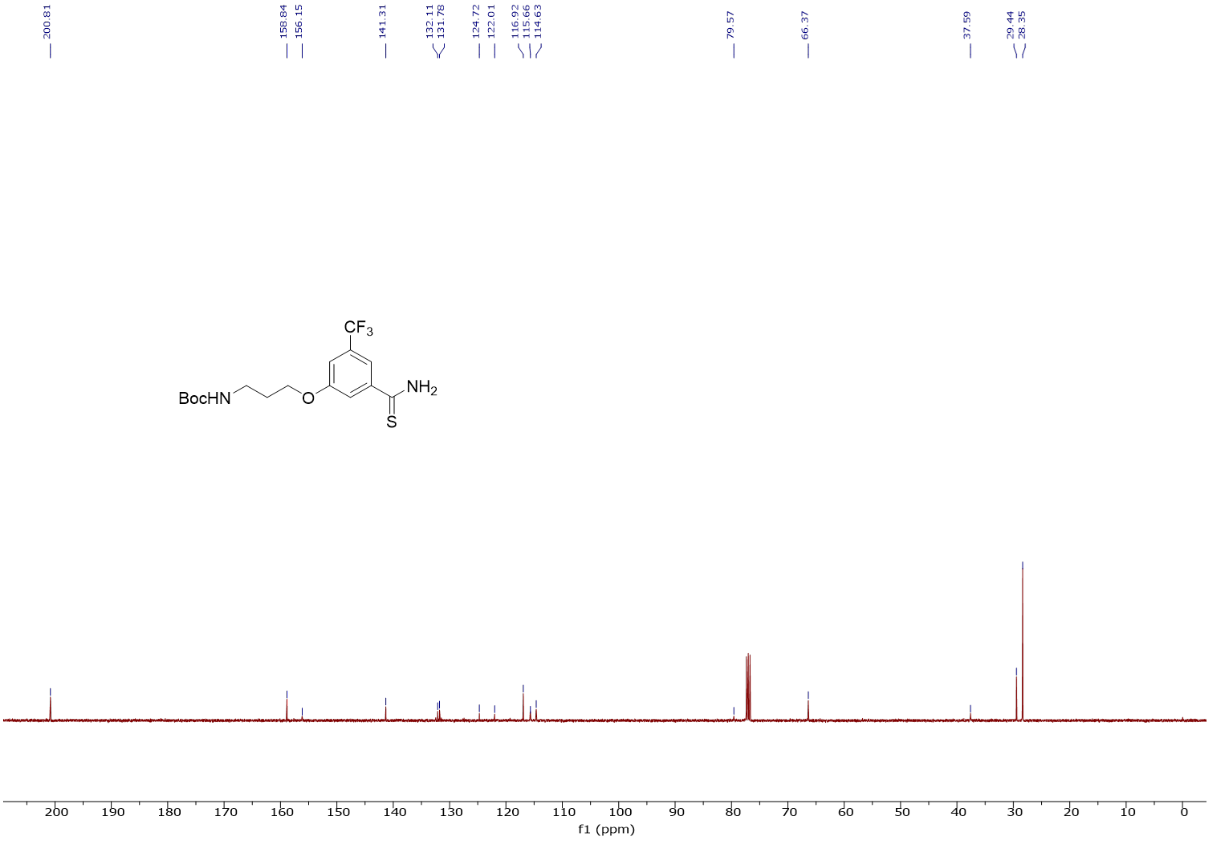

^{1}H NMR of **S-4**

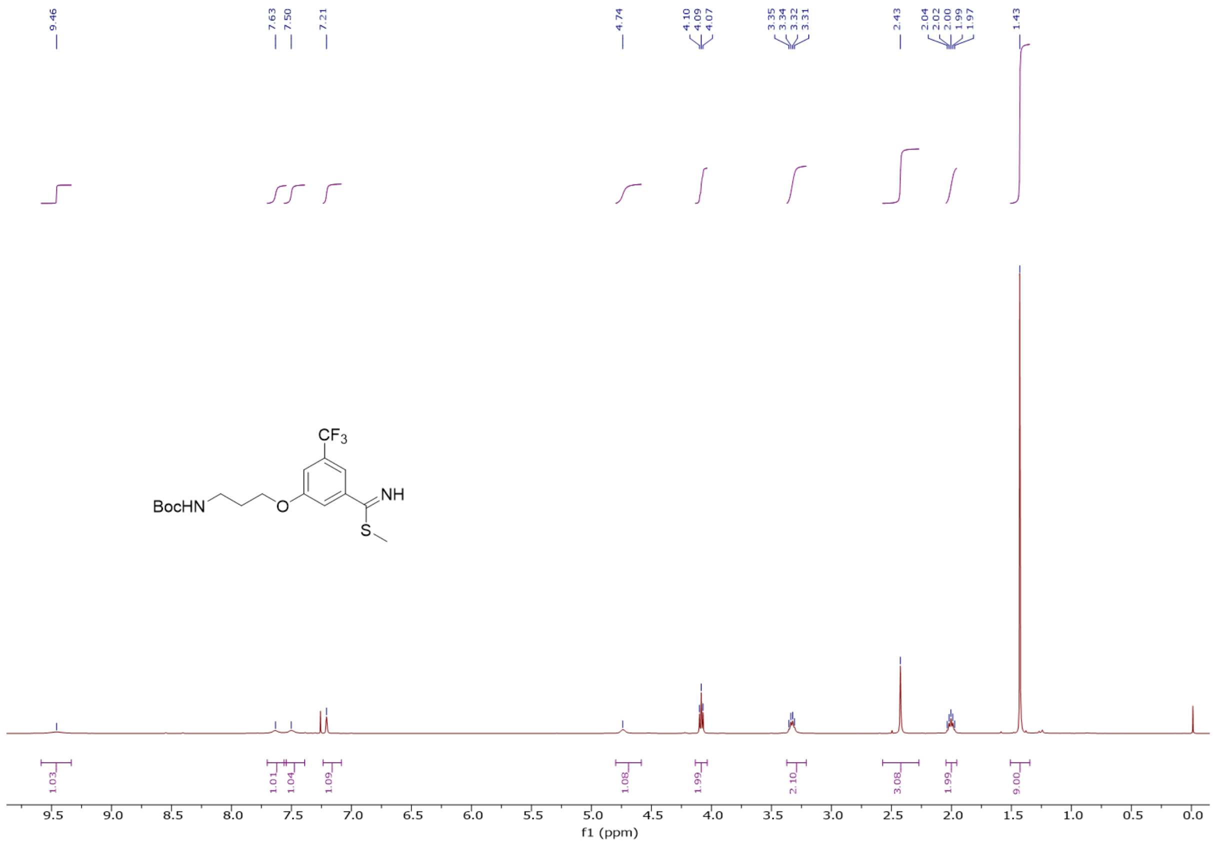

^{13}C NMR of **S-4**

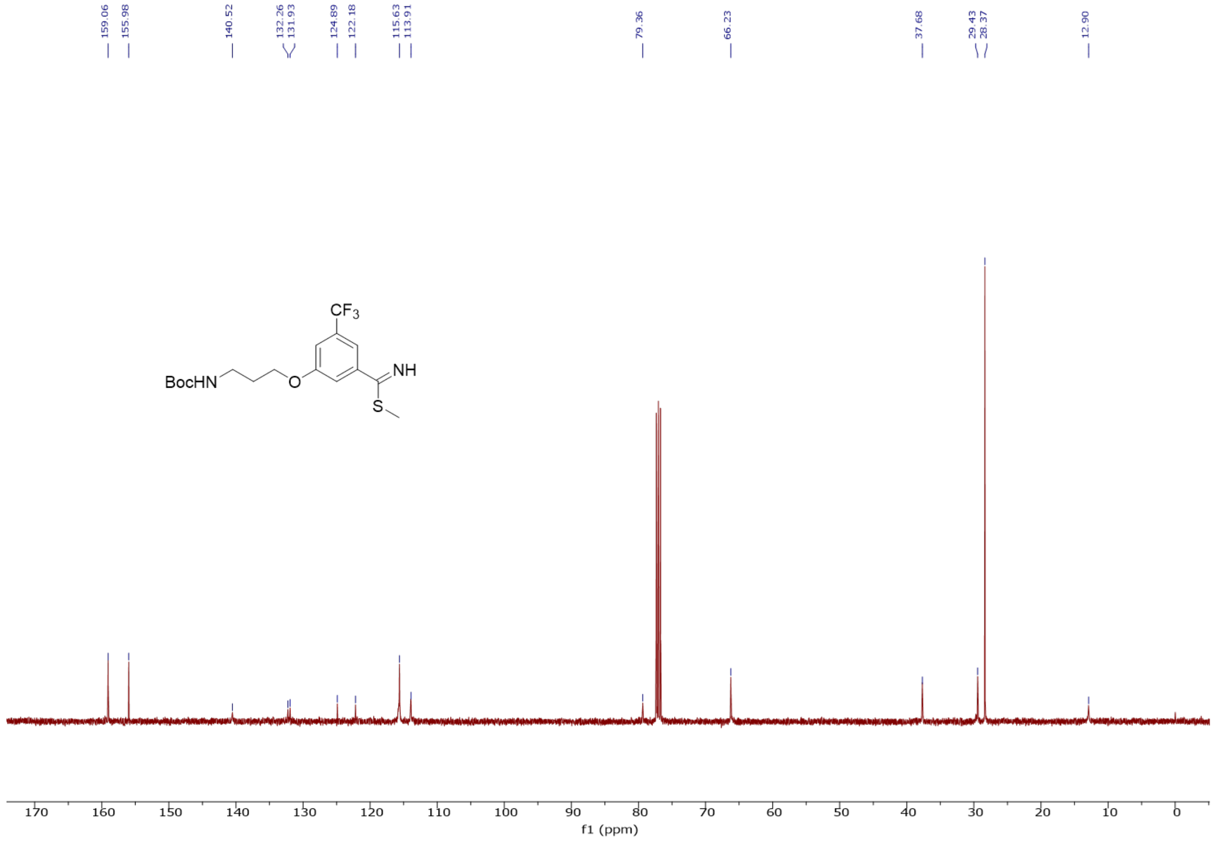

^{1}H NMR of **S-5**

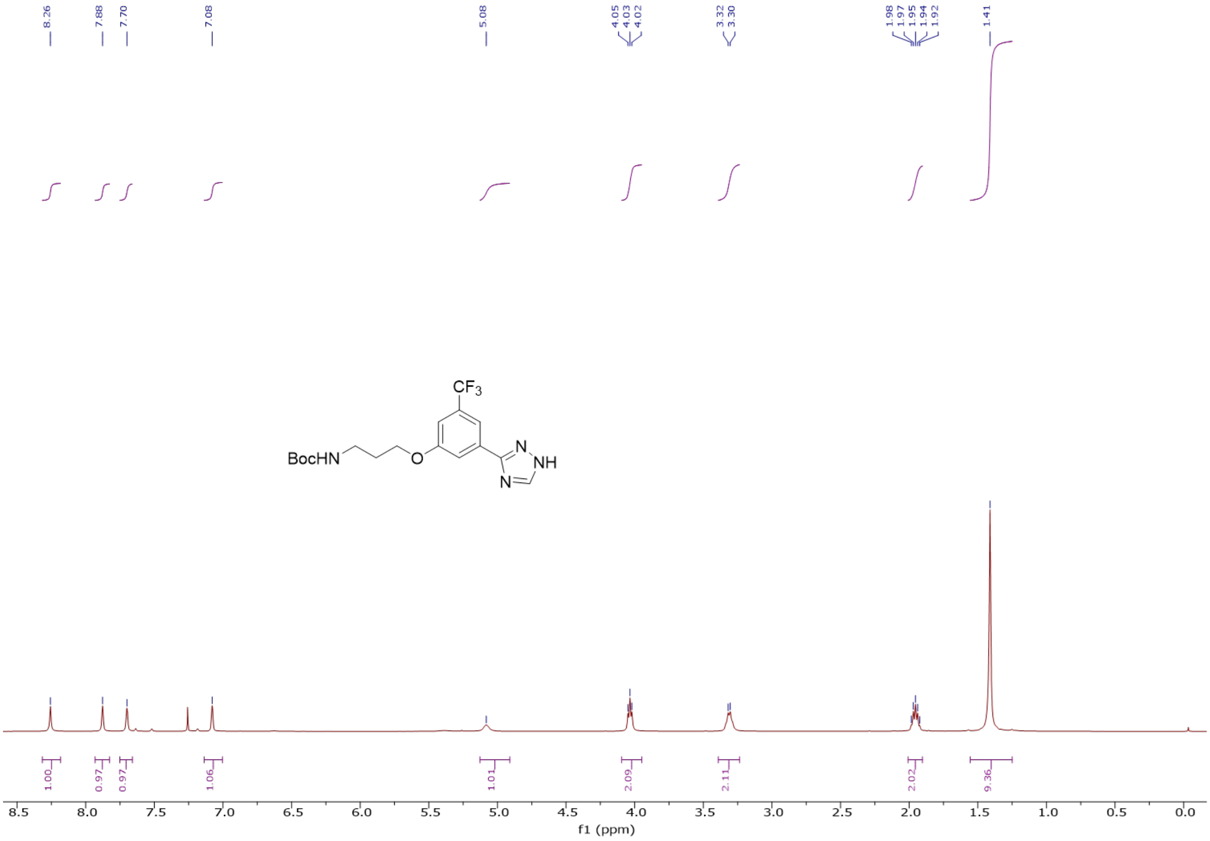

^{13}C NMR of **S-5**

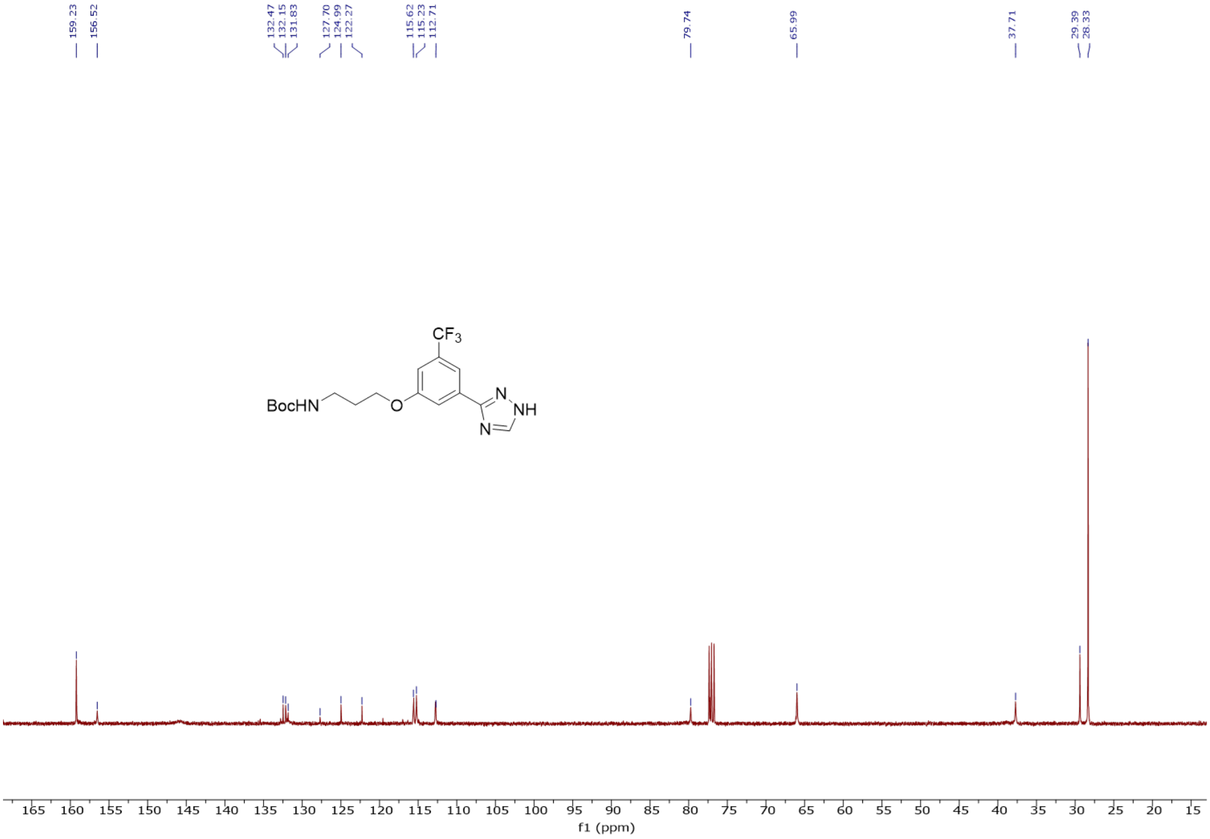

^{1}H NMR of **S-7**

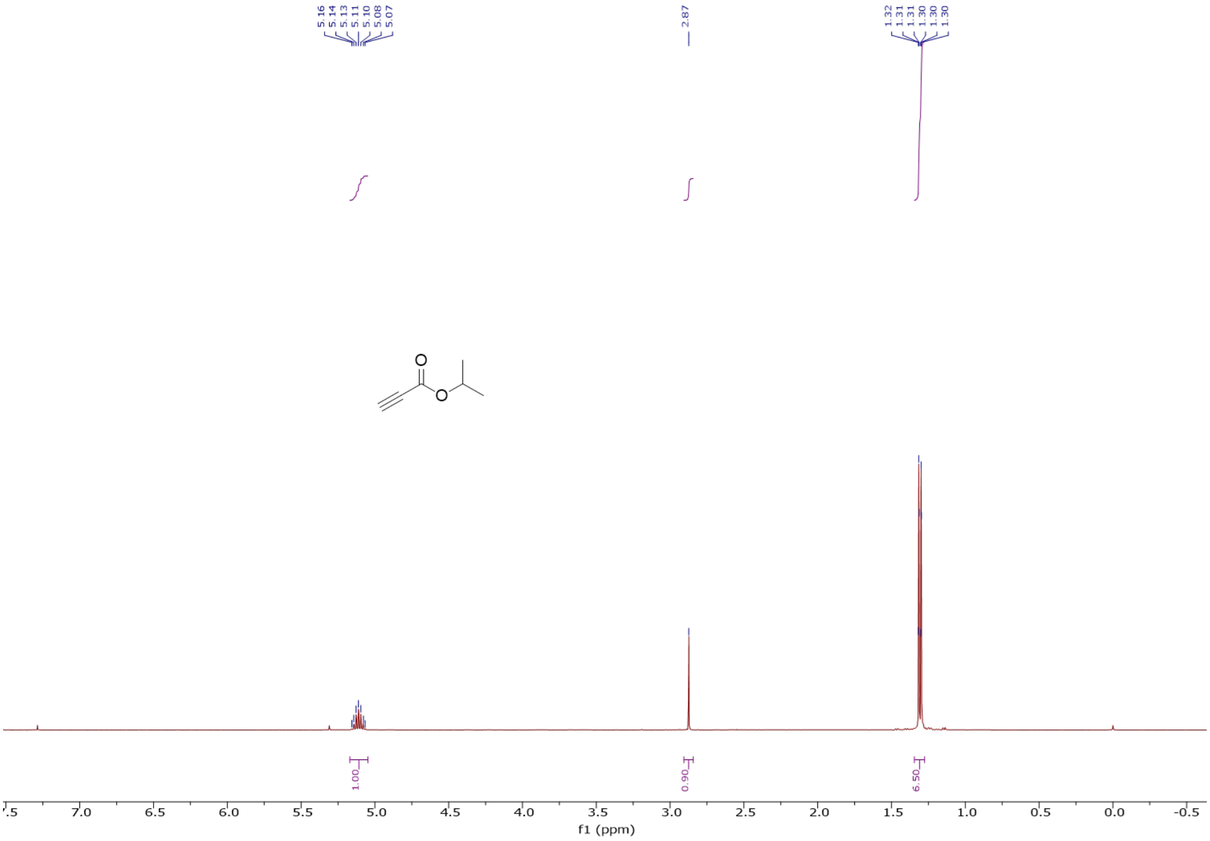

^{1}H NMR of **S-8**

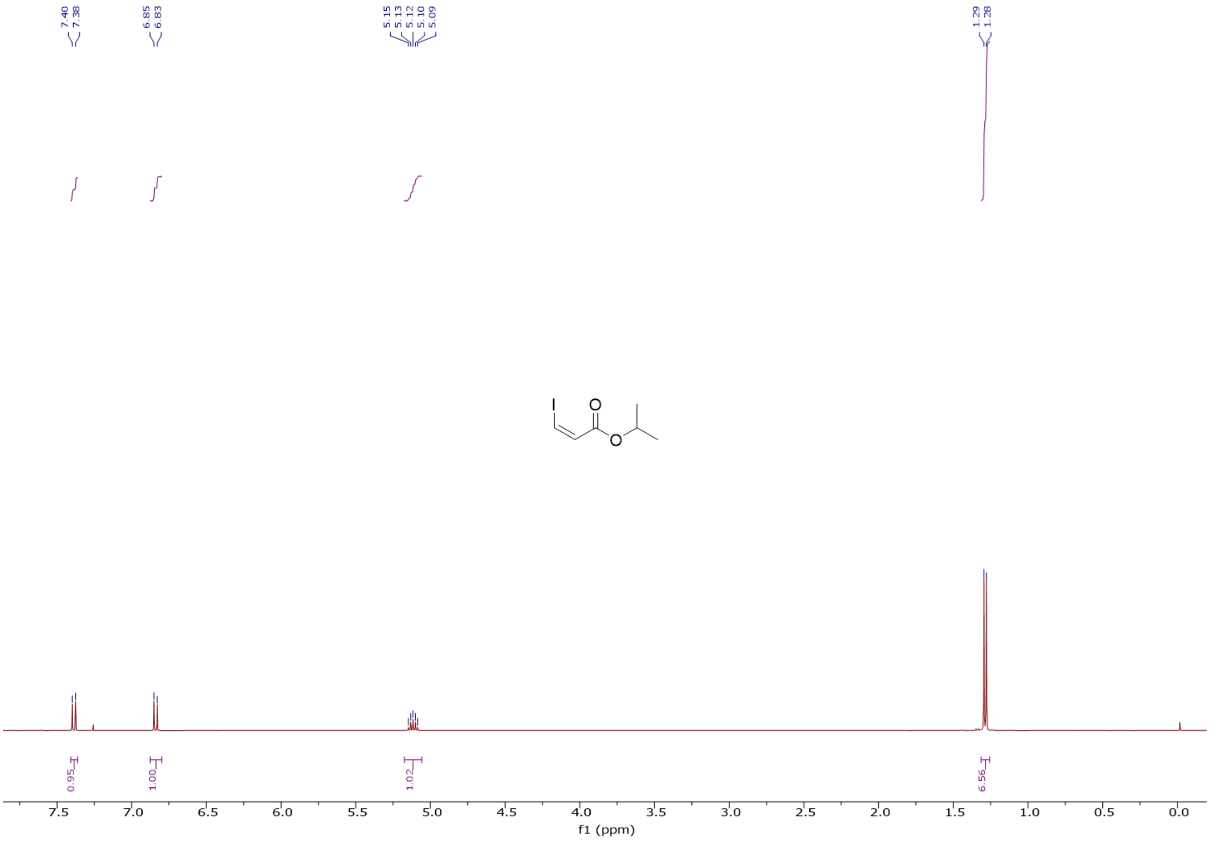

^{1}H NMR of **S-9**

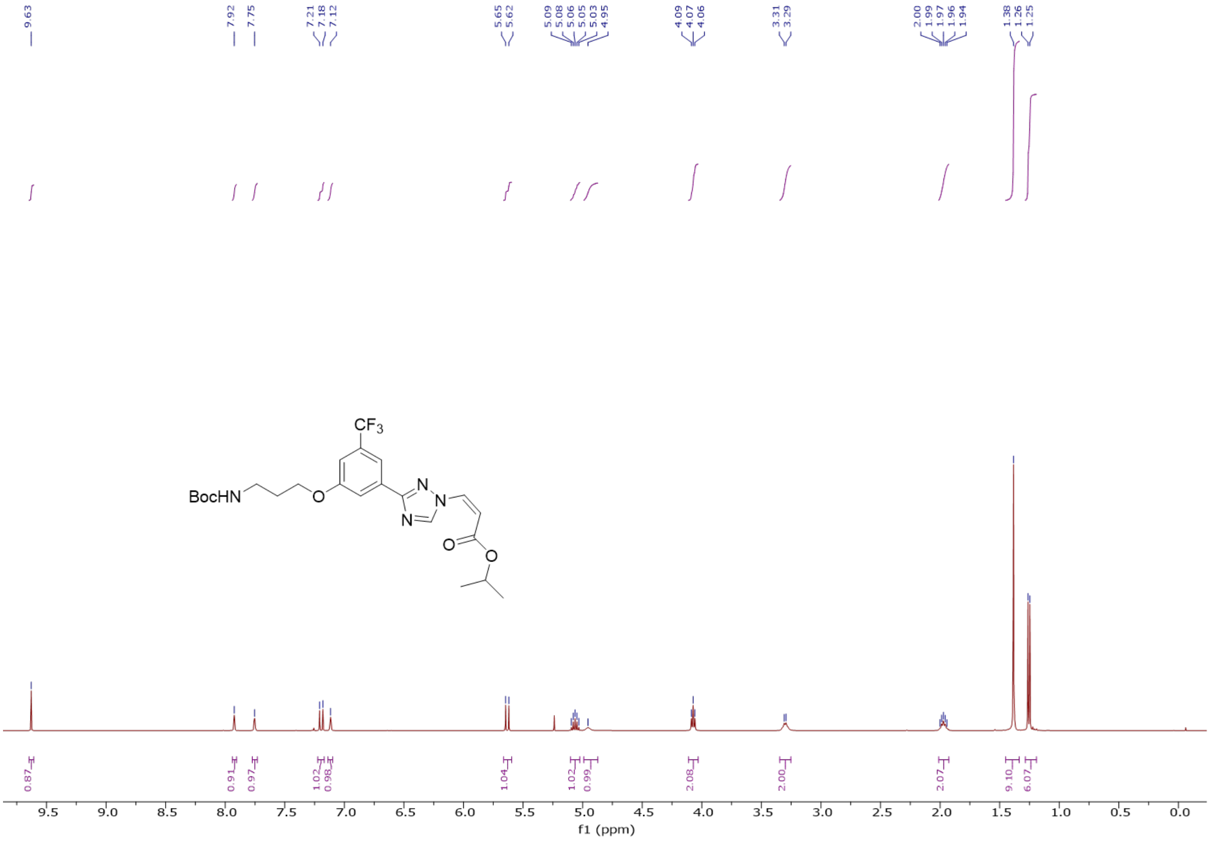

^{13}C NMR of **S-9**

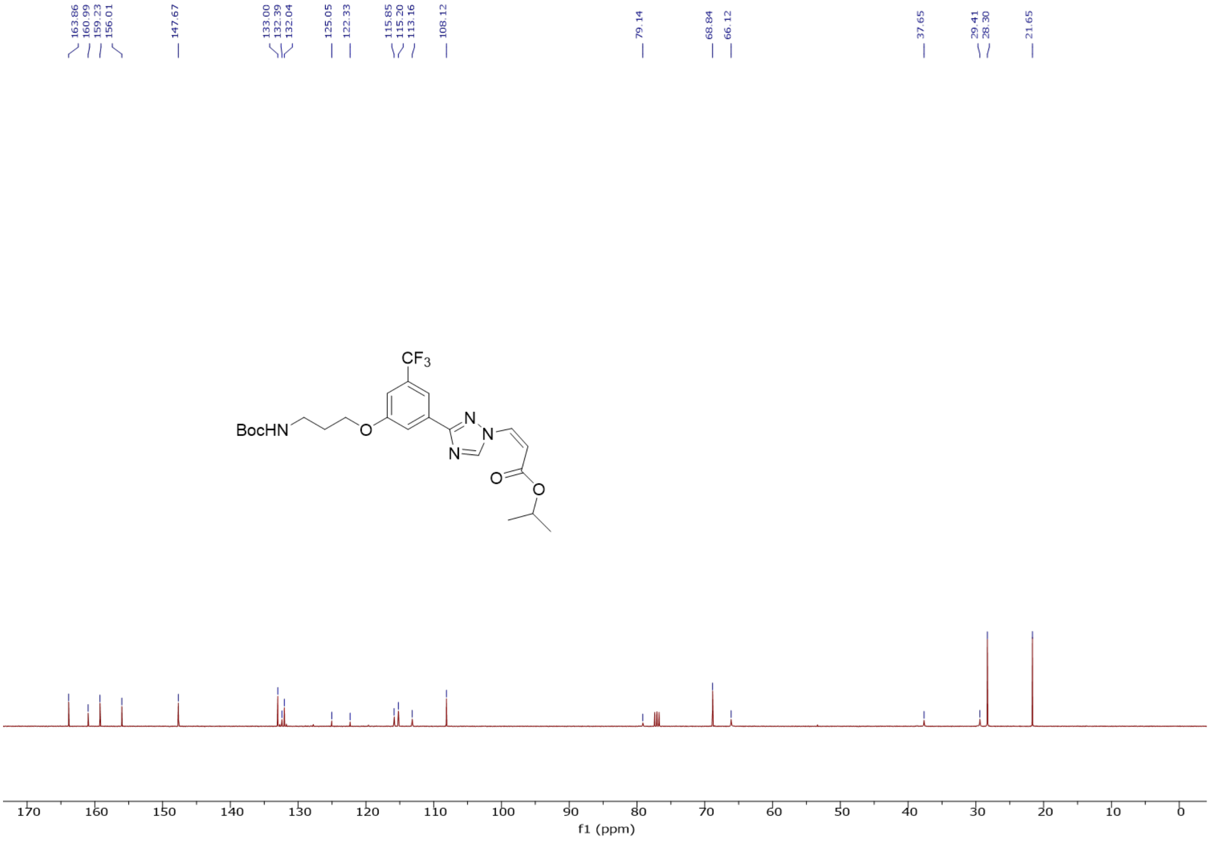

^{1}H NMR of **S-11**

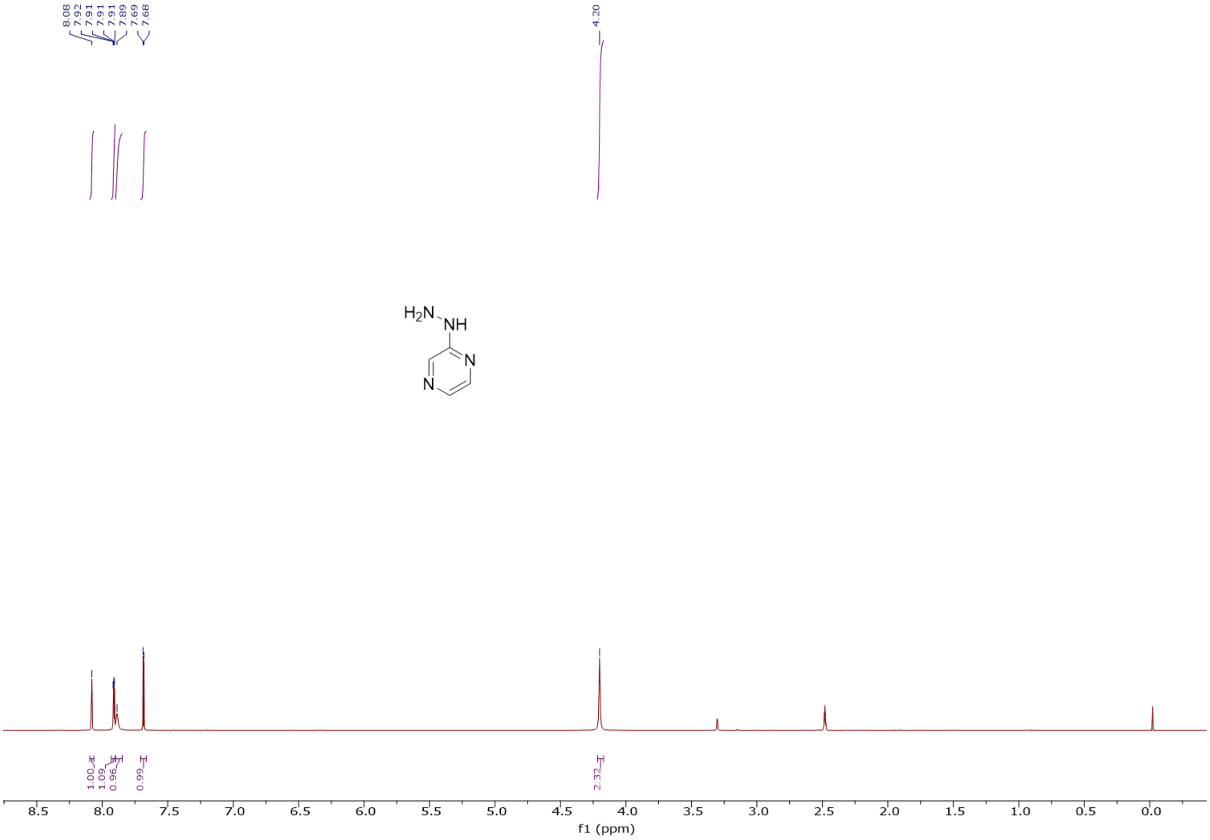
8.08
7.92
7.91
7.91
7.91
7.89
7.69
7.68
4.20
H2N
NH
N
N
1.00
1.09
0.96
0.99
2.32
8.5
8.0
7.5
7.0
6.5
6.0
5.5
5.0
4.5
4.0
3.5
3.0
2.5
2.0
1.5
1.0
0.5
0.0
f1 (ppm)

^{1}H NMR of **S-12**

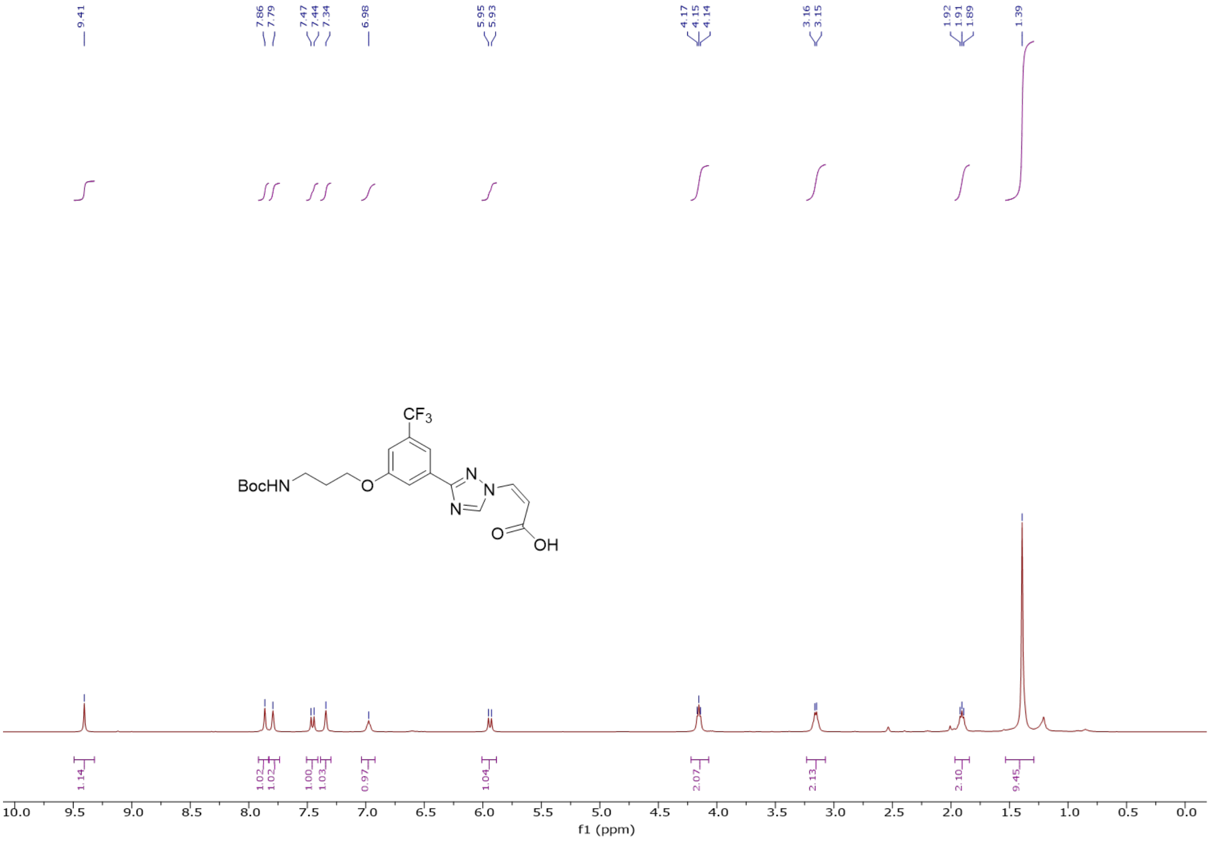

^{13}C NMR of **S-12**

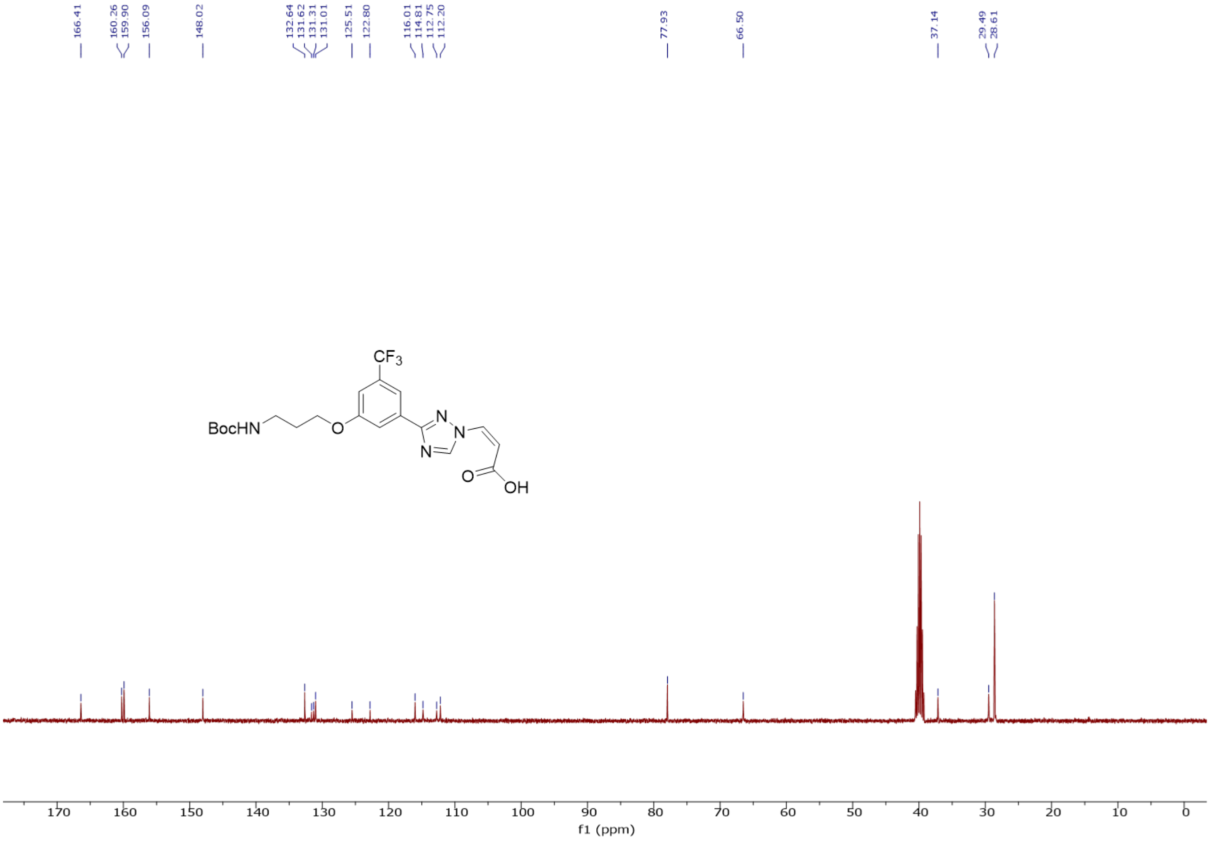

^{1}H NMR of **S-13**

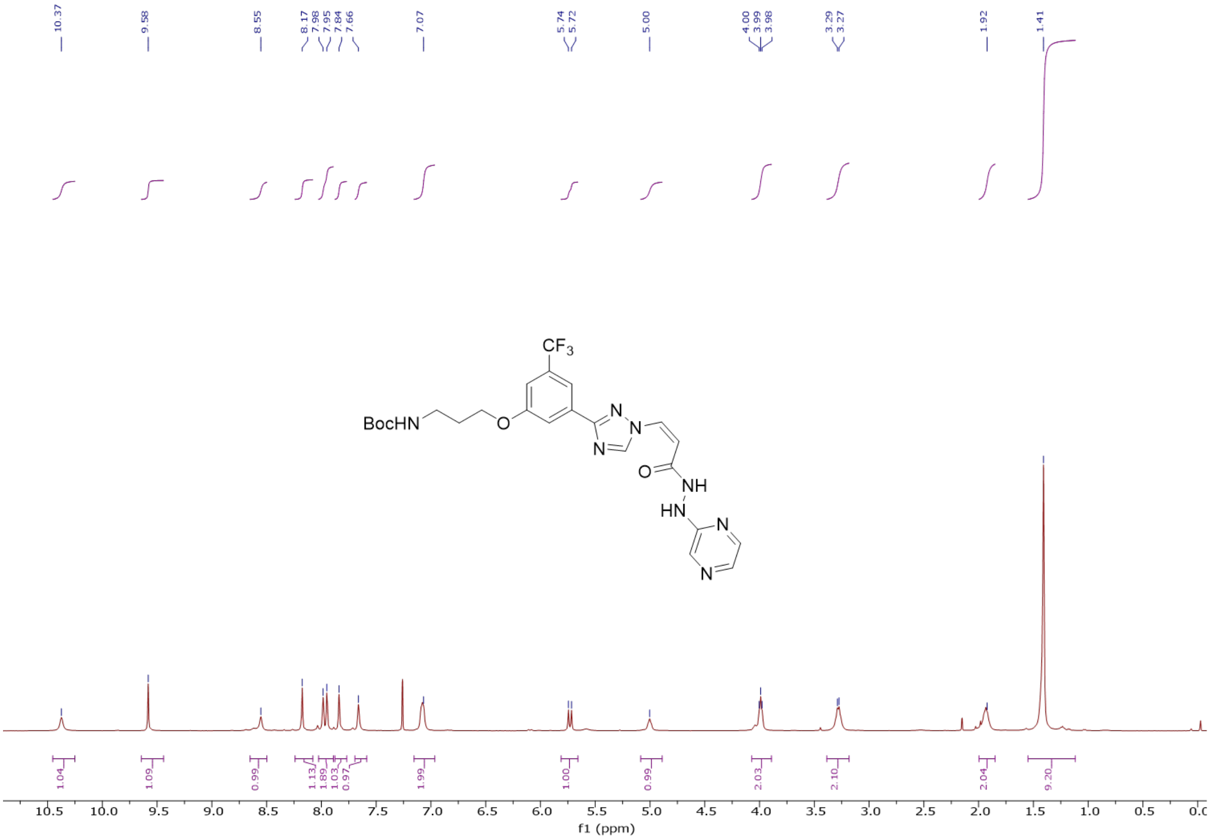

^{13}C NMR of **S-13**

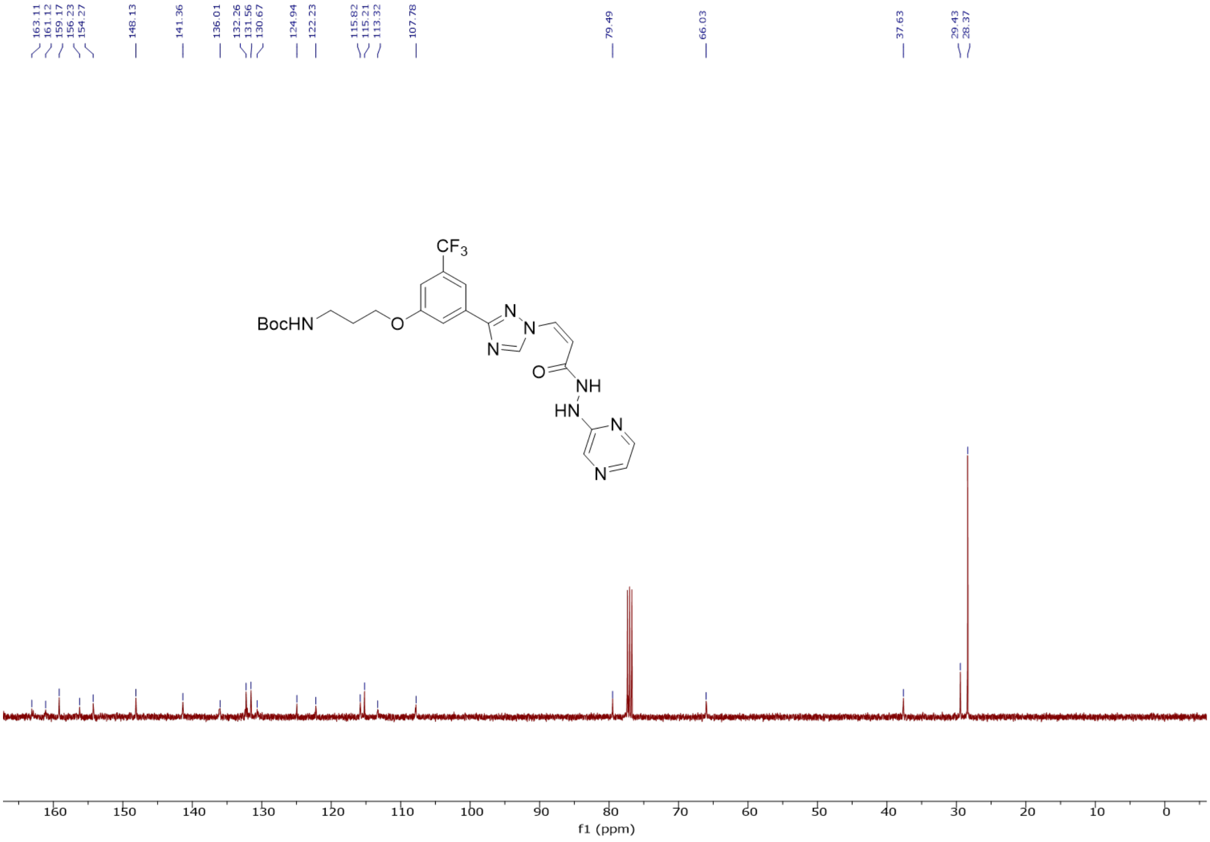

^{1}H NMR of **SEL-TCO**

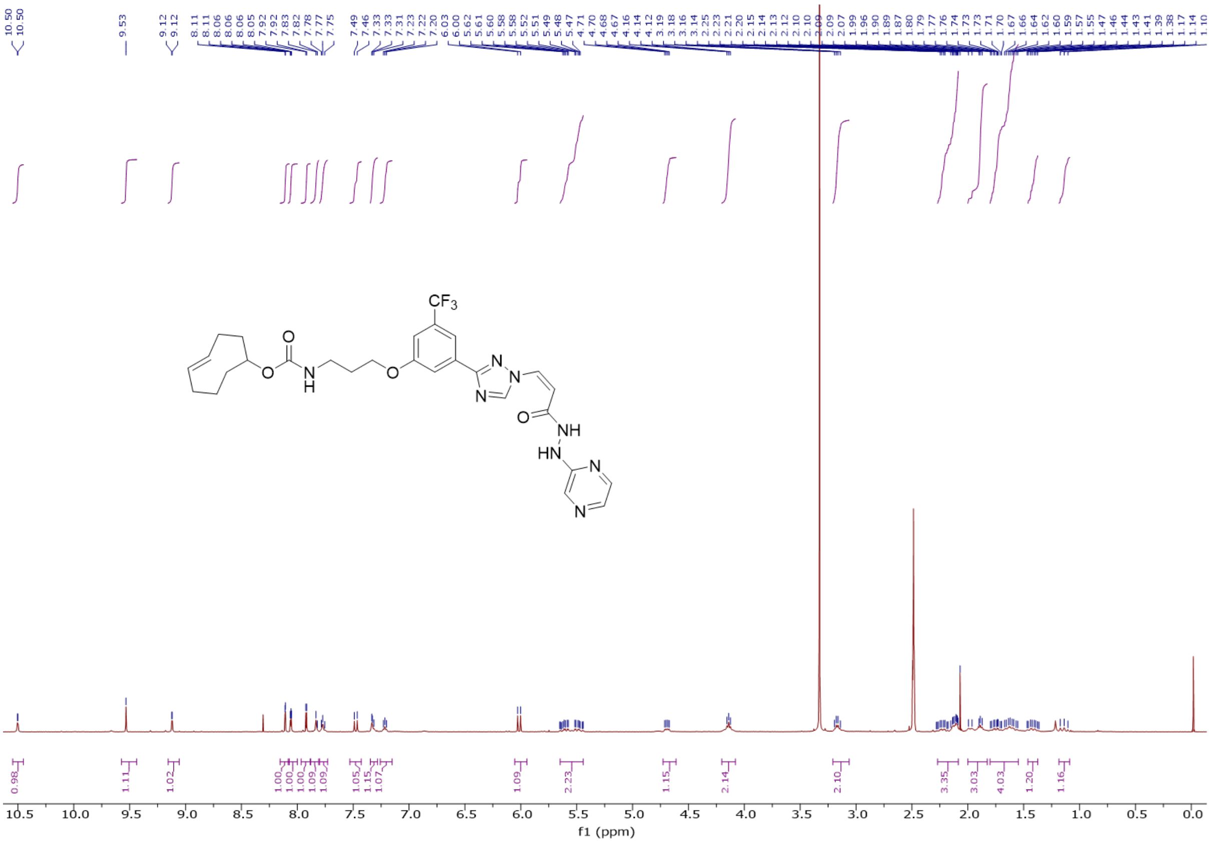

^{13}C NMR of **SEL-TCO**

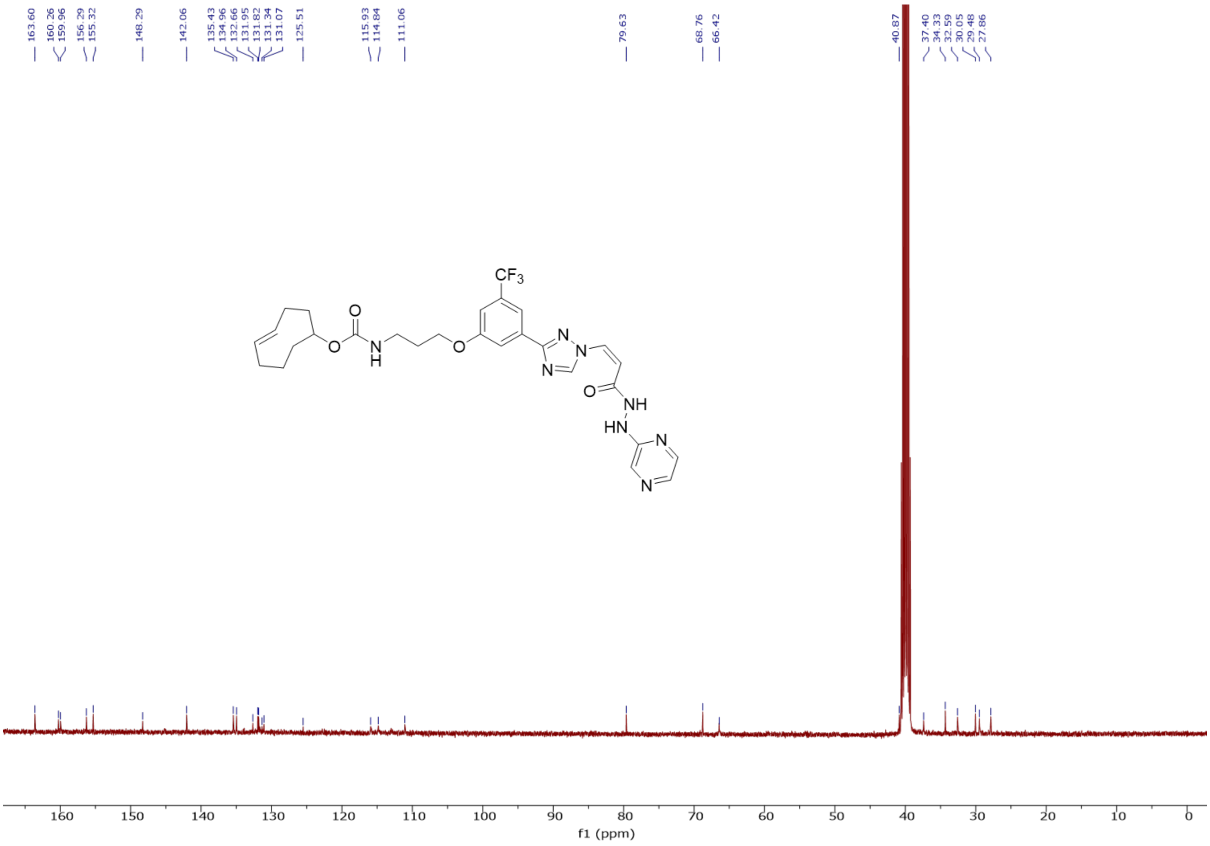

ESI-MS of **SEL-TCO**

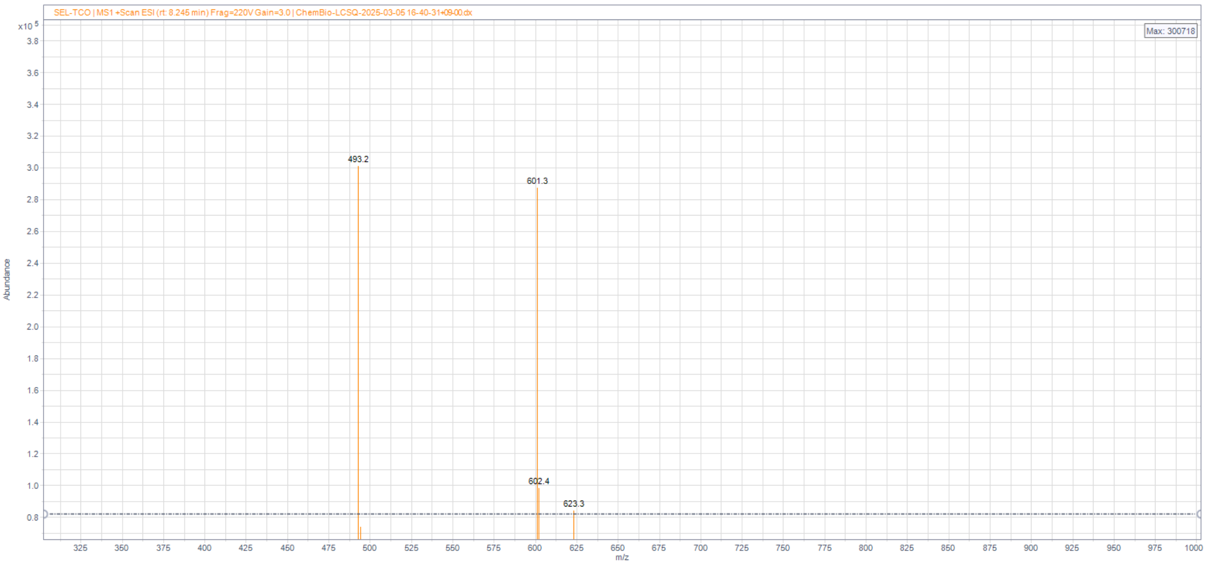

^{1}H NMR of **TP-2**

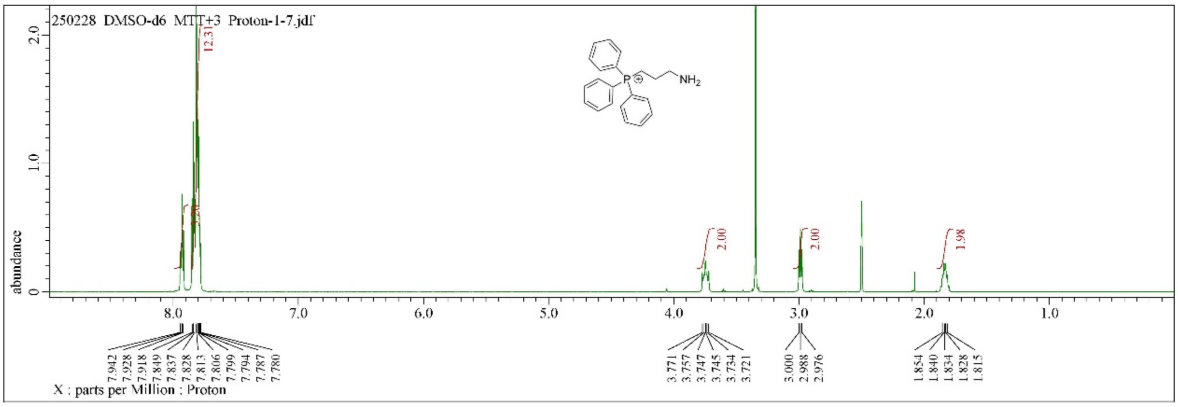

ESI-MS of **TP-2**

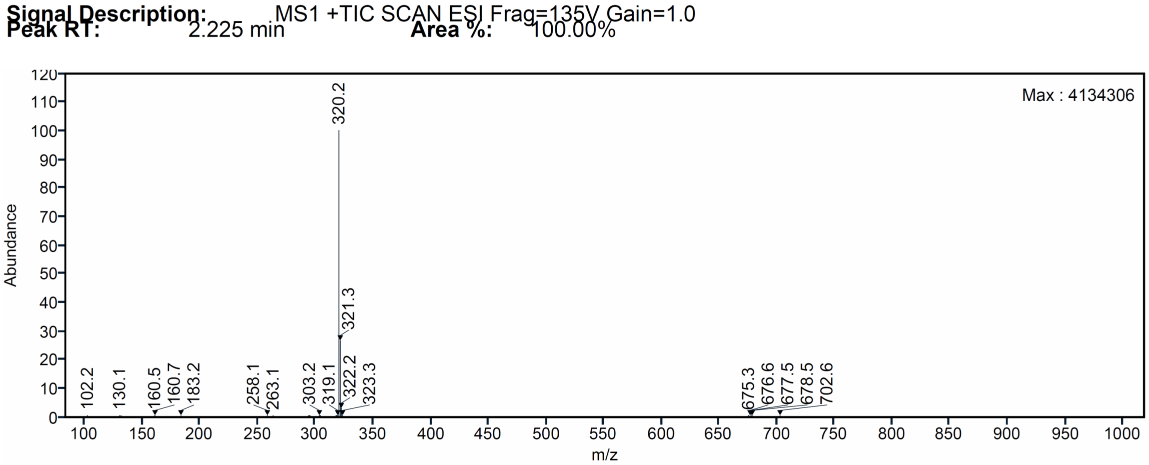

^{1}H NMR of **TP-3**

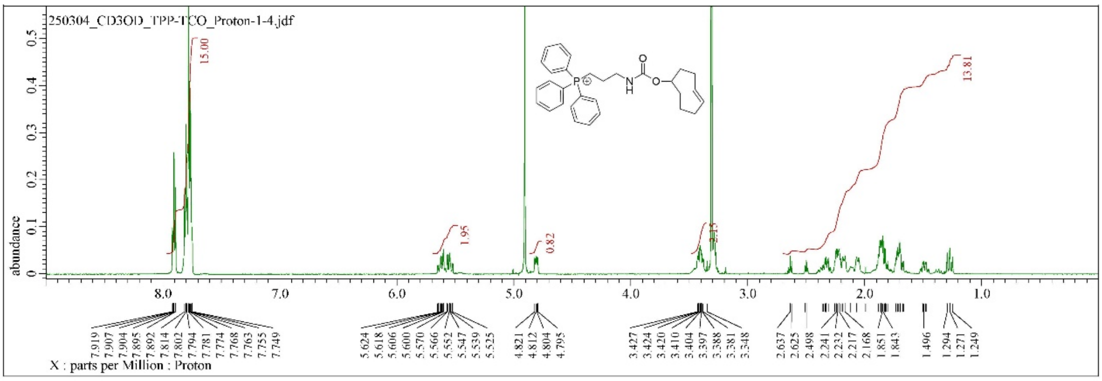

ESI-MS of **TP-3**

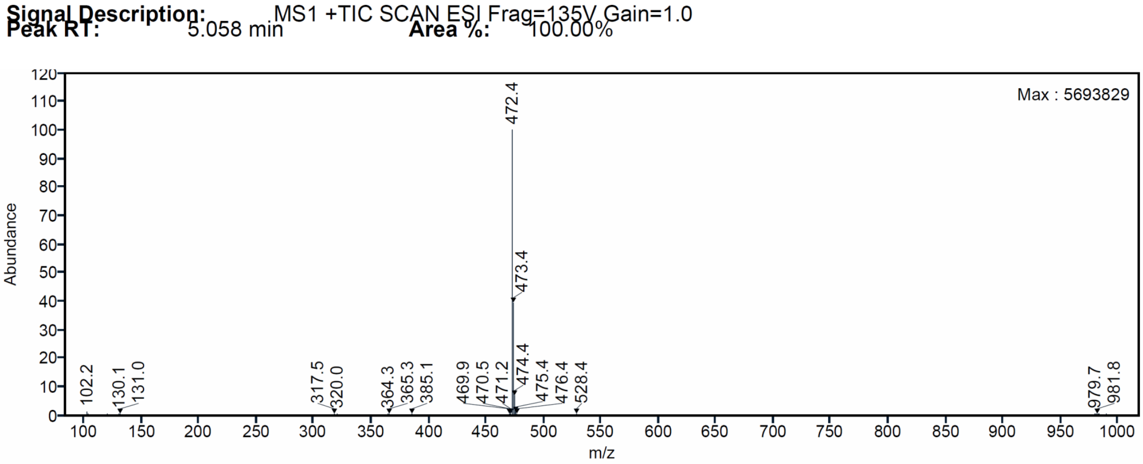

References

(1) Sung, J.; Rho, J. G.; Jeon, G. G.; Chu, Y.; Min, J. S.; Lee, S.; Kim, J. H.; Kim, W.; Kim, E. A New Infrared Probe Targeting Mitochondria via Regulation of Molecular Hydrophobicity. *Bioconjug Chem* **2019**, *30* (1), 210-217.

(2) Lang, K.; Davis, L.; Wallace, S.; Mahesh, M.; Cox, D. J.; Blackman, M. L.; Fox, J. M.; Chin, J. W. Genetic Encoding of bicyclononynes and trans-cyclooctenes for site-specific protein labeling in vitro and in live mammalian cells via rapid fluorogenic Diels-Alder reactions. *J Am Chem Soc* **2012**, *134* (25), 10317-10320.

(3) Sarris, A. J. C.; Hansen, T.; de Geus, M. A. R.; Maurits, E.; Doelman, W.; Overkleeft, H. S.; Codee, J. D. C.; Filippov, D. V.; van Kasteren, S. I. Fast and pH-Independent Elimination of trans-Cyclooctene by Using Aminoethyl-Functionalized Tetrazines. *Chemistry* **2018**, *24* (68), 18075-18081.

(4) Eising, S.; Lelivelt, F.; Bonger, K. M. Vinylboronic acids as fast reacting, synthetically accessible, and stable bioorthogonal reactants in the Carboni–Lindsey reaction. *Angewandte Chemie* **2016**, *128* (40), 12431-12435.
